# Effects of exercise on BMI z-score in overweight and obese children and adolescents: a systematic review with meta-analysis

**DOI:** 10.1186/1471-2431-14-225

**Published:** 2014-09-09

**Authors:** George A Kelley, Kristi S Kelley, Russell R Pate

**Affiliations:** Meta-Analytic Research Group, School of Public Health, Department of Biostatistics, Robert C Byrd Health Sciences Center, West Virginia University, Morgantown, WV 26506-9190 USA; Children’s Physical Activity Research Group, Department of Exercise Science, University of South Carolina, Columbia, SC USA

**Keywords:** Exercise, Physical activity, Overweight, Obesity, Adiposity, Body composition, Body mass index, Children, Adolescents, Meta-analysis, Systematic review

## Abstract

**Background:**

Overweight and obesity are major public health problems in children and adolescents. The purpose of this study was to conduct a systematic review with meta-analysis to determine the effects of exercise (aerobic, strength or both) on body mass index (BMI) z-score in overweight and obese children and adolescents.

**Methods:**

Studies were included if they were randomized controlled exercise intervention trials ≥ 4 weeks in overweight and obese children and adolescents 2 to 18 years of age, published in any language between 1990–2012 and in which data were available for BMI z-score. Studies were retrieved by searching eleven electronic databases, cross-referencing and expert review. Two authors (GAK, KSK) selected and abstracted data. Bias was assessed using the Cochrane Risk of Bias Assessment Instrument. Exercise minus control group changes were calculated from each study and weighted by the inverse of the variance. All results were pooled using a random-effects model with non-overlapping 95% confidence intervals (CI) considered statistically significant. Heterogeneity was assessed using Q and *I*^*2*^ while funnel plots and Egger’s regression test were used to assess for small-study effects. Influence and cumulative meta-analysis were performed as well as moderator and meta-regression analyses.

**Results:**

Of the 4,999 citations reviewed, 835 children and adolescents (456 exercise, 379 control) from 10 studies representing 21 groups (11 exercise, 10 control) were included. On average, exercise took place 4 x week for 43 minutes per session over 16 weeks. Overall, a statistically significant reduction equivalent to 3% was found for BMI z-score . No small-study effects were observed and results remained statistically significant when each study was deleted from the model once. Based on cumulative meta-analysis, results have been statistically significant since 2009. None of the moderator or meta-regression analyses were statistically significant. The number-needed-to treat was 107 with an estimated 116,822 million obese US children and adolescents and approximately 1 million overweight and obese children and adolescents worldwide potentially improving their BMI z-score by participating in exercise.

**Conclusions:**

Exercise improves BMI z-score in overweight and obese children and adolescents and should be recommended in this population group. However, a need exists for additional studies on this topic.

**Electronic supplementary material:**

The online version of this article (doi:10.1186/1471-2431-14-225) contains supplementary material, which is available to authorized users.

## Background

It has been suggested that exercise is a promising intervention in overweight and obese children and adolescents [1]. Potential benefits include, but are not limited to, improvements in (1) cardiovascular fitness, (2) muscular strength and (3) vascular function [1]. In addition, exercise may reduce body fat and increase lean body mass [1], thereby reducing the risk of overweight and obesity in adulthood [2] and the subsequent premature morbidity and mortality associated with such [3].

Body mass index (BMI) is the most common method used to assess overweight and obesity in children and adolescents. Previous systematic reviews, with or without meta-analysis, have generally focused on multiple lifestyle interventions, for example, diet and exercise, in the prevention and treatment of overweight and obesity in children and adolescents [4–29]. Consequently, the independent effects of an intervention such as exercise on BMI measures cannot be elucidated. From the investigative team’s perspective, this is important to know when attempting to develop effective interventions for treating overweight and obese children and adolescents. For the five systematic reviews with meta-analyses that have included a focus on exercise [4, 12, 19, 28, 29], four of five (80%) reported a non-significant change in BMI among male and female children and adolescents [4, 12, 19, 28]. However, all five suffer from one or more of the following potential limitations: (1) inclusion of a small number of studies with exercise as the only intervention [4, 12, 19], (2) inclusion of non-randomized trials [12, 29], and (3) inclusion of children and adolescents who were not overweight or obese [12, 28, 29]. Furthermore, using the Assessment of Multiple Systematic Reviews (AMSTAR) instrument for assessing the methodological quality of systematic reviews [30], the overall quality score (0% to 100% with higher scores representing better quality) was only 45% [29], 55% [4, 28], 64% [19] and 82% [12] for these five meta-analyses. Finally, none of the reviews included BMI z-score [4, 12, 19, 28, 29], an outcome that has been suggested to be more valid than other BMI measures in children and adolescents [31]. It is critically important to develop a better understanding of the overall magnitude of effect, as well as potential factors associated with, exercise-induced changes on BMI in overweight and obese children and adolescents. Given the former, the primary purpose of this study was to use the meta-analytic approach to examine the effects of exercise on BMI z-score in overweight and obese children and adolescents. A secondary purpose was to examine other selected variables that have been shown to be associated with cardiovascular as well as all-cause mortality; body weight, BMI in kg^.^ m^2^, BMI percentile, body fat (absolute and percent), fat-free mass, waist circumference, waist-to-hip ratio, resting systolic and diastolic blood pressure, total cholesterol (TC), high-density lipoprotein cholesterol (HDL-C), ratio of total cholesterol to high-density lipoprotein cholesterol (TC:HDL-C), low-density lipoprotein cholesterol (LDL-C), triglycerides (TG), non-high density lipoprotein cholesterol (non-HDL-C), fasting glucose, fasting insulin, glycosylated hemoglobin, physical activity levels, maximum oxygen consumption (ml^.^kg^-1.^min^-1^), muscular strength, energy intake and energy expenditure [32].

## Methods

This study was conducted and reported according to the general guidelines recommended by the Primary Reporting Items for Systematic Reviews and Meta-analyses (PRISMA) Statement [33]. A PRISMA checklist indicating where these items are reported in the original Word document can be found in Additional file 1.

### Study eligibility criteria

The *a priori* inclusion criteria for this meta-analysis were as follows: (1) randomized controlled trials with the unit of assignment at the participant level, (2) comparative control group (non-intervention, attention control, usual care, placebo), (3) exercise-only intervention group (no diet intervention) lasting ≥ 4 weeks, (4) overweight and obese children and adolescents 2 to 18 years of age, (5) studies published in full in any language and source (journal articles, dissertations, etc.) between January 1, 1990 and December 31, 2012, (6) data available for BMI z-score or data to calculate BMI-z-score. Studies were limited to randomized trials because it is the only way to control for confounders that are not known or measured as well as the observation that nonrandomized controlled trials tend to overestimate the effects of healthcare interventions [34, 35]. Four weeks was chosen as the lower cut point for intervention length based on previous research demonstrating improvements in adiposity over this period of time in 11-year old girls [36]. Participants were limited to overweight and obese children and adolescents, as defined by the original study authors, because it has been shown that this population is at an increased risk for premature morbidity and mortality throughout their lifetime [37]. The year 1990 was chosen as the start point for searching in order to increase the chances of receiving data from investigators. The review protocol for this study is available from the corresponding author upon request.

### Data sources

Studies up to December 31, 2012 were retrieved using the following 11 electronic databases: (1) Medline, (2) CINAHL, (3) Scopus, (4) Academic Search Complete, (5) Educational Research Complete, (6) Web of Science, (7) Sport Discus, (8) ERIC, (9) LILACS, (10) Cochrane Central Register of Controlled Trials (CENTRAL) and (11) Proquest. All electronic searches were conducted by the second author with assistance from a Health Sciences librarian at West Virginia University. While the search strategies used varied per the requirements of the different databases searched, keywords centered around the terms “exercise”, “overweight”, “obesity”, “children,” “adolescents” and “randomized”. The search strategies for all databases searched can be found in Additional file 2. After removing duplicates, the overall precision of the searches was calculated by dividing the number of studies included by the total number of studies screened [38]. The number needed to read (NNR) was then calculated as the inverse of the precision [38]. In addition to electronic database searches, cross-referencing for potentially eligible meta-analyses from retrieved reviews was also conducted. All studies were stored in Reference Manager, version 12.0.1 [39].

### Study selection

All studies were selected by the first two authors, independent of each other. Disagreements regarding the final list of studies to include were resolved by consensus. If consensus could not be reached, the third author acted as an arbitrator. After an initial list of included studies was developed, the third author, an expert in exercise and overweight and obesity in children and adolescents, reviewed the list for completeness. All included studies as well as a list of excluded studies, including reasons for exclusion, were stored in Reference Manager (version 12.0.1) [39].

### Data abstraction

Prior to data abstraction, a detailed codebook that could hold at least 242 items per study was developed by all three members of the research team in Microsoft Excel 2007 [40]. The major categories of variables that were coded included: (1) study characteristics, (2) subject characteristics, (3) exercise program characteristics, (4) primary outcomes and (5) secondary outcomes. The primary outcome for this study was BMI z-score. Secondary outcomes included body weight, BMI in kg^.^ m^2^, BMI percentile, body fat (absolute and percent), fat-free mass, waist circumference, waist-to-hip ratio, resting systolic and diastolic blood pressure, TC, HDL-C, TC:HDL-C, LDL-C, TG, non-HDL-C, fasting glucose, fasting insulin, glycosylated hemoglobin, physical activity levels, maximum oxygen consumption (ml^.^kg^-1.^min^-1^), muscular strength, energy intake and energy expenditure.

Based on abstracted data and similar to a previous study in children and adolescents [41], intensity of training was calculated as metabolic equivalents (METS) using the following categories: (1) low = 2.35, based on range of 1.8 to 2.9, (2) moderate = 4.45, based on a range of 3.0 to 5.9, (3) high = 7.5, based on a MET value greater than 5.9 [42]. In addition, the following calculations were made: (1) minutes of training per week (frequency × duration), (2) MET minutes per week (frequency × duration × METS), (3) total minutes over the entire intervention (length × frequency × duration), (4) total MET minutes over the entire intervention (length × frequency × duration × METS). Where possible, calculations were also adjusted for compliance, defined as the percentage of exercise sessions attended.

Missing primary outcome data were requested from the author(s). Multiple publication bias was avoided by only including data from the most recently published study. Data abstraction occurred using the same procedure as the selection of studies. Using Cohen’s kappa statistic [43], the overall agreement rate prior to correcting discrepant items was 0.93.

### Risk of bias

The Cochrane Collaboration risk of bias instrument was used to assess bias across six categories: (1) random sequence generation, (2) allocation concealment, (3) blinding of participants and personnel, (4) blinding of outcome assessment, (5) incomplete outcome data, (6) selective reporting and (7) whether or not participants were exercising regularly, as defined by the original study authors, prior to taking part in the study [44]. Each item was classified as having either a high, low, or unclear risk of bias [44]. Assessment for risk of bias was limited to the primary outcome of interest, changes in BMI z-score. Since it’s impossible to blind participants to group assignment in exercise intervention protocols, all studies were considered to be at a high risk of bias with respect to the category “blinding of participants and personnel”. Based on previous research, no study was excluded based on the results of the risk of bias assessment [45]. All assessments were performed by the first two authors, independent of each other. Both authors then met and reviewed every item for agreement. Disagreements were resolved by consensus.

### Statistical analysis

The *a priori* plan was to conduct a one-step individual participant data (IPD) meta-analysis [46]. However, because of (1) the inability to obtain IPD from all eligible studies, (2) the inability to resolve discrepancies between the IPD provided and data reported in the published studies, for example, final sample sizes and (3) the potential loss of power with fewer included studies at the IPD level, a *post hoc* decision was made to conduct an aggregate data meta-analysis, an approach similar to conducting a two-step meta-analysis with IPD [46].

### Calculation of effect sizes for primary and secondary outcomes from each study

The primary outcome for this study was effect size (ES) changes in BMI z-score. This was calculated by subtracting the change score difference in the exercise group from the change score difference in the control group. Variances were calculated from the pooled standard deviations of change scores in the intervention and control groups. If change score standard deviations were not available, these were calculated from reported 95% confidence intervals (CI) or pre and post standard deviation (SD) values according to procedures developed by Follmann et al. [47]. Each ES was then weighted by the inverse of its variance [48]. With the exception of fasting insulin, all other secondary outcomes were calculated using the same approach as for BMI z-score. For fasting insulin, the standardized mean difference ES, adjusted for small sample bias, was calculated from each study in order to create a common metric for the pooling of findings [48]. This was calculated as the difference in change scores between the exercise and control groups divided by the pooled SD of the change scores [48]. For all ES’s, the beneficial direction of effect was the natural direction of benefit, (for example, negative values for decreases in BMI z-score, positive values for increases in maximum oxygen consumption, etc.).

### Pooled estimates for primary and secondary outcomes

Random-effects, method-of-moments models that incorporate heterogeneity into the overall estimate were used to pool results for BMI z-score and secondary outcomes from each study [49]. Multiple groups from the same study were analyzed independently as well as collapsing multiple groups so that only one ES represented each outcome from each study [50]. Non-overlapping 95% CI were considered statistically significant. Secondary outcomes were only included if data for the primary outcome of interest, BMI z-score, were available. To enhance practical application, the number-needed-to treat (NNT) was calculated for any overall findings that were reported as statistically significant [51]. This was accomplished using the approach suggested by the Cochrane Collaboration and assuming a control group risk of 10% [52]. Based on the NNT for changes in BMI z-score, gross estimates of the number of obese children and adolescents in the US who could benefit from exercise, based on 12.5 million obese children and adolescents [53] as well as the number of overweight and obese children worldwide who could benefit from exercise, based on 110 million overweight or obese children [54, 55], were provided. It was assumed that none of the overweight and obese children and adolescents included in the original estimates were exercising regularly.

### Stability and validity of changes in primary and secondary outcomes

Heterogeneity of results between studies was examined using Q and *I*^*2*^ [56]. To determine treatment effects in a new trial, 95% prediction intervals (PI) were also calculated [57, 58]. Small-study effects (publication bias, etc.) were examined using the regression approach of Egger et al. [59, 60]. In order to examine the effects of each result from each study on the overall findings, results were analyzed with each study deleted from the model once. Cumulative meta-analysis, ranked by year, was used to examine the accumulation of evidence over time [61]. *Post hoc,* changes in BMI z-score were examined with two studies in which reductions in energy intake occurred deleted from the model [62, 63].

### Moderator analysis for BMI z-score

Between-group differences (Q_b_) in BMI z-score for categorical variables were examined using mixed effects ANOVA-like models for meta-analysis [64]. This consisted of a random effects model for combining studies within each subgroup and a fixed effect-model across subgroups [64]. Study-to-study variance (tau-squared) was considered to be unequal for all subgroups. This value was computed within subgroups but not pooled across subgroups. Planned categorical variables to examine *a priori* included: country in which the study was conducted (USA, other), type of control group (non-intervention, other), whether IPD was provided (yes, no), whether the study was funded (yes, no), power/sample size analysis provided (yes, no), adverse events (yes, no), risk of bias assessment (separate assessment of low, high or unclear risk according to sequence generation, allocation concealment, blinding of participants and personnel, blinding of outcome assessment, incomplete outcome data, selective reporting, whether subjects were inactive prior to enrollment), gender and race/ethnicity. Using the categories yes, no or some, analyses were planned for the following variables: prescribed drugs, changes in exercise and/or physical activity levels beyond the exercise intervention, hyperlipidemia, type 1 diabetes, type 2 diabetes, hypertension, heart problems, metabolic syndrome, cancer, asthma and pubertal stage. In addition, type of exercise (aerobic, strength, both, other), exercise supervision (yes, no), setting that exercise took place (facility, home, both), type of participation (self, group, both), type of analysis (analysis-by-protocol versus intention-to-treat) and intensity of exercise (low, moderate, high), were examined [65]. All moderator analyses were considered exploratory [66].

### Meta-regression for changes in BMI z-score and potential covariates

Simple mixed-effects, method of moments meta-regression was used to examine the potential association between changes in BMI z-score and continuous variables [64]. Because missing data for different variables from different studies was expected, only simple meta-regression was planned and performed. Potential predictor variables, established *a priori,* included year of publication, percentage of dropouts, age in years, baseline BMI z-score, as well as the following exercise intervention characteristics: length of training (weeks), frequency of training (days per week), duration of training (minutes per session), total minutes per week (unadjusted and adjusted for compliance), MET minutes per week (unadjusted and adjusted for compliance), total minutes for the entire intervention period (unadjusted and adjusted for compliance), and compliance, defined as the percentage of exercise sessions attended. Similar to moderator analyses, all meta-regression tests were considered exploratory [66].

## Results

### Study characteristics

**Figure 1:**
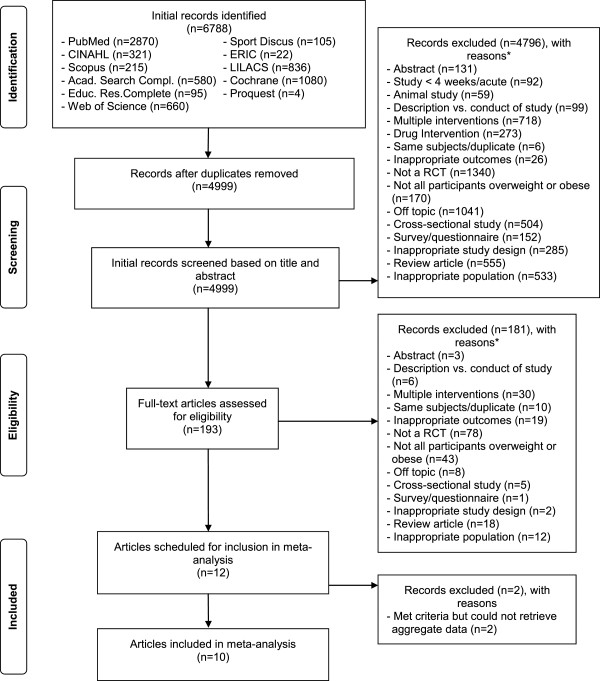
**Flow diagram for the selection of studies.** *, number of reasons exceeds the number of studies because some studies were excluded for more than one reason.

A general description of the characteristics of each study is shown in Table 1. Of the 4,999 citations reviewed, 10 studies representing 21 groups (11 exercise, 10 control) and final assessment of BMI z-score in 835 children and adolescents (456 exercise, 379 control), were included [62, 63, 67–74]. The precision of the searches was 0.0028 while the NNR was 357. A description of the search process, including the reasons for excluded studies, is shown in Figure 1 while a list of excluded studies, including the reasons for exclusion, is shown in Additional file 3. All studies were published in English-language journals between the years 2004 and 2012 [62, 63, 67–74]. Seven studies used a non-intervention control group [62, 63, 69–73] while the remaining three used some type of attention control [67, 68, 74]. For matching, seven studies did not match participants [62, 67, 69, 71–74] while the remaining three matched participants according to race and gender [68, 70] or age, gender and BMI [63]. For data analysis, four studies used the intention-to-treat approach [67, 68, 70, 74], another five appeared to use the per-protocol approach [63, 69, 71–73] and one used both [62]. Sample size justification was provided by five of the 10 studies [62, 67, 68, 70, 74] while all ten reported receiving some type of funding to conduct their study [62, 63, 67–74]. The dropout rate for the eight studies in which data were available [62, 63, 67, 68, 70, 71, 73, 74] ranged from 0% to 34% for the 9 exercise groups for which data were available  and 0% to 26% for the 8 control groups in which data were available for . Detailed data regarding the reasons for dropping out for each study are available upon request from the corresponding author. For the three studies that reported sufficient data on adverse events [68, 70, 74], two reported no serious adverse events [70, 74] while one reported a foot fracture in one participant as well as several minor injuries [68].

**Table 1:** Characteristics of included studies*

Study	Country	Participants	Exercise intervention
Daley et al., 2006 [67]	United Kingdom	47 Asian, Black and White male and female adolescents 11 to 16 yrs of age assigned to an exercise therapy group (n = 28) or an exercise placebo group (n = 23)	3 days/wk of aerobic exercise, 40-49% HRR, 30 min/session for 8 wks (24 sessions) followed by an at home program for 6 wks
Davis et al., 2012 [68]	United States	222 overweight Black, White and Hispanic male and female children ages 7–11 yrs assigned to a low dose , or high dose aerobic or control group	5 days/wk, running games, jump rope, modified basketball and soccer, average HR >150 bpm, 2–20 min sessions/day (high dose) or 1 – 20 min session/day (low dose) for 10–15 wks (school semester)
Farpour-Lambert et al., 2009 [62]	Switzerland	44 pre-pubertal obese male and female children assigned to an exercise or control group	3 days/wk, 30 min of aerobic exercise at 55-65% VO_2_max, 20 min strength training, 10 min stretching/cool down in addition to physical education for 3 months
Hagstromer et al., 2009 [69]	Sweden	31 obese male and female adolescents assigned to an exercise or control group	1 hr/wk of group activities (brisk walking, spinning, strength training (50-70% 1RM), swimming) for 13 wks
Kelly et al., 2004 [63]	United States	20 overweight male and female children and adolescents assigned to an exercise or control group	4 days/wk, stationary cycling, 30–50 min/session, 50-80% VO_2max_, for 8 wks
Maddison et al., 2011 [70]	New Zealand	322 Maori, Pacific, ZN Euro/other overweight and obese male and female children assigned to an active video game intervention or to a sedentary video game control group	60 min moderate to vigorous PA on most days of the wk by 1) supplementing periods of inactivity w/active video game play and 2) substituting periods of nonactive video games with active ones, for 12 wks
Meyer et al., 2006 [71]	Germany	67 obese male and female children assigned to an exercise or control group	3 days/wk, 60–90 min/session, supervised swimming, aerobic training, sports games, and walking, for 6 months
Murphy et al., 2009 [72]	United States	35 overweight male and female children 7 to 12 yrs of age assigned to either an exercise (n = 23) or delayed treatment control (n = 12) group	5 days/wk of home-based Dance, Dance Revolution (DDR),10-30 min/session while wearing a pedometer to count steps for 12 wks
Shaibi et al., 2006 [73]	United States	22 overweight, adolescent Latino males assigned to either a resistance training or control group	2 days/wk, resistance training,10 exercises, 1–3 sets, 8–15 reps, 62-97% 1RM, 1–2 min rest between sets, for 16 wks
Weintraub et al., 2008 [74]	United States	21 overweight Hispanic/Latino, Black or African American, Native Hawaiian or Pacific Islander male and female children, assigned to either a coed soccer or active placebo nutrition and health education group	3-4 days/wk, 75 min activity/session for soccer group; 25-session, weekly state-of-the-art information-based nutrition and health education intervention for active placebo group, for 6 months

Notes: *, Description of groups limited to those from each study that met the criteria for inclusion while sample sizes limited to those in which final BMI *z*-scores were available; yrs, year(s); min, minute(s); h, hour(s); wk, week(s); RM, repetition maximum; reps, repetitions; VO_2max_, maximum oxygen consumption; MHR, maximum heart rate; HRR, heart rate reserve; HR, heart rate; bpm, beats per minute; PE, physical education; PA, physical activity; ; mean ± standard deviation.

Initial physical characteristics of the exercise and control groups are shown in Tables 1 and 2. For prior exercise, three studies reported that none of the participants were exercising regularly prior to enrollment [62, 68, 71], one reported that some were exercising regularly [69], while another reported that participants exceeded the guidelines for physical activity at baseline [70]. During the intervention period and when compared to the control group, one study reported a reduction in total daily physical activity in the exercise group [69]. Participants included those with and without cardiovascular disease risk factors [62, 63, 67–74].

**Table 2:** **Initial physical characteristics of participants**

	Exercise				Control			
Variable	Groups/Participants(#)		Mdn	Range	Groups/Participants(#)		Mdn	Range
Age (years)	11/456	11.4 ± 2.1	11	9 - 15	10/379	11.8 ± 2.2	11	9 – 16
Height (cm)	6/242	155.2 ± 9.7	156	140 - 166	6/232	154.6 ± 12.0	156	136 – 168
Body weight (kg)	6/242	71.3 ± 15.2	69	51 - 90	6/232	73.8 ± 18.7	72	47 – 98
BMI (z-score)	11/456	2.3 ± 0.5	2	1 – 3	10/379	2.4 ± 0.6	2	1 – 3
BMI (kg/m^2^)	10/428	28.4 ± 2.9	27	25 - 33	9/356	29.7 ± 3.4	31	25 – 35
BMI (percentile)	6/201	97.8 ± 1.0	97	97 - 100	5/125	98.4 ± 1.1	98	98 – 100
Fat mass (kg)	5/217	28.4 ± 6.2	31	21 - 34	5/209	29.5 ± 6.0	30	21 – 37
Body fat (%)	9/416	38.2 ± 4.2	38	32 – 45	8/343	38.7 ± 4.7	40	31 – 45
Fat-free mass (kg)	5/79	43.5 ± 10.7	43	28 – 54	5/69	45.0 ± 13.7	43	26 - 62
Waist circumference (cm)	3/193	91.3 ± 6.0	88	87 - 98	3/184	95.5 ± 7.8	95	88 – 104
Waist-to-hip ratio	2/54	0.94 ± 0.01	0.94	0.93 – 0.94	2/46	0.95 ± 0.01	0.95	0.94 – 0.95
Systolic BP (mmHg)	6/112	117.6 ± 8.6	119	107 – 128	6/103	120.8 ± 10.7	123	101 – 133
Diastolic BP (mmHg)	5/79	65.8 ± 6.5	67	56 – 74	5/69	66.2 ± 9.0	68	52 - 77
TC (mg/dl)	4/65	156.6 ± 13.3	157	143 – 170	4/55	158.0 ± 3.9	157	143 – 170
HDL-C (mg/dl)	5/98	39.8 ± 4.8	39	34 – 46	5/89	39.1 ± 5.2	39	33 – 46
TC:HDL-C	3/43	4.2 ± 0.6	4	4 – 5	3/33	4.3 ± 0.5	4	4 – 5
LDL-C (mg/dl)	5/98	100.8 ± 12.1	105	86 – 112	5/89	104.5 ± 9.0	109	94 – 112
TG (mg/dl)	5/96	96.5 ± 27.5	98	53 – 125	5/89	97.3 ± 28.4	95	62 – 141
Non-HDL-C (mg/dl)	3/43	116.7 ± 14.0	109	108 – 133	3/33	121.0 ± 7.3	122	113 – 128
Fasting glucose (mg/dl)	6/207	89.6 ± 3.8	91	84 – 91	6/211	89.8 ± 3.5	90	85 – 93
VO_2max_ (ml^.^kg^-1.^min^-1^)	8/385	28.5 ± 4.3	26	23 – 37	7/310	28.1 ± 4.8	27	23 – 37
Energy intake (kcals)	2/18	2407 ± 840	2407	1813 - 3001	2/19	2326 ± 1007	2326	1614 – 3038

Notes: Groups/Participants (#), number of groups and participants in which data were available; , mean ± standard deviation; Mdn, Median; BMI, body mass index; BP, blood pressure; TC, total cholesterol; HDL-C, high-density lipoprotein cholesterol; TC:HDL-C, ratio of total cholesterol to high-density lipoprotein cholesterol; LDL-C, low-density lipoprotein cholesterol; TG, triglycerides; Non-HDL-C, non-high-density lipoprotein cholesterol; VO_2max_, maximum oxygen consumption; kcals, kilocalories.

Characteristics of the exercise programs for each group from each study are described in Table 1. As can be seen, the exercise interventions varied widely. Length of training for the 11 exercise groups ranged from 8 to 24 weeks , frequency from 2 to 7 times per week  and duration from 6 to 75 minutes per session . Intensity of training was classified as moderate for 7 groups and high for 4. Seven of the ten studies focused primarily on aerobic types of activities [63, 67, 68, 70–72, 74], one on strength training [73] and two on both [62, 69]. Eight groups from seven studies participated in supervised exercise [62, 63, 68, 69, 71, 73, 74], two in unsupervised exercise [70, 72] and one in both [67].

For the four studies [62, 68, 73, 74] and five groups in which data were available, compliance, defined as the percentage of exercise sessions attended, ranged from 42% to 96% . Total minutes per week of exercise ranged from 40 to 250  while MET minutes per week ranged from 180 to 1873 . When adjusted for compliance for the four studies and five groups in which compliance data were available [62, 68, 73, 74] total minutes per week of exercise ranged from 85 to 168  while MET minutes per week ranged from 554 to 1260 . Total minutes of training over the entire length of the interventions ranged from 780 to 6000  while total MET minutes ranged from 6648 to 18881 .

### Risk of bias assessment

**Figure 2:**
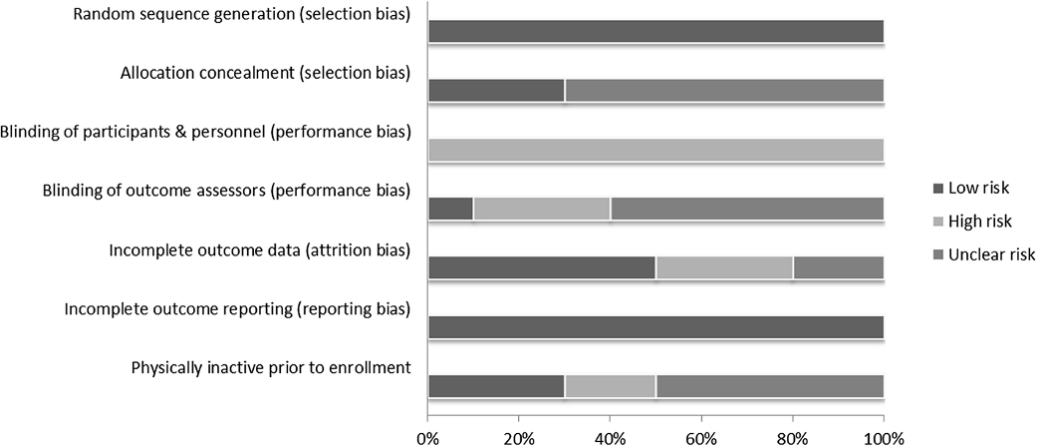
**Risk of bias.** Pooled risk of bias results using the Cochrane Risk of Bias Assessment Instrument.

Risk of bias results are shown in Figure 2 while results for each item from each study are shown in Additional file 4. As can be seen, there was a general lack of clear reporting for several potential risks of bias as well as an increased risk of bias for several variables.

### Primary outcome

#### BMI Z-score

**Figure 3:**
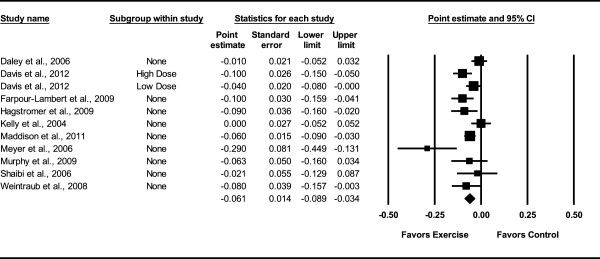
**Forest plot for changes in BMI z-score.** Forest plot for point estimate changes in BMI z-score. The black squares represent the mean difference while the left and right extremes of the squares represent the corresponding 95% confidence intervals. The middle of the black diamond represents the overall mean difference while the left and right extremes of the diamond represent the corresponding 95% confidence intervals.

**Figure 4:**
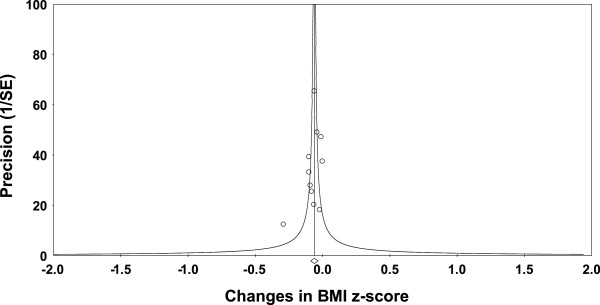
**Funnel plot for changes in BMI z-score.**

**Figure 5:**
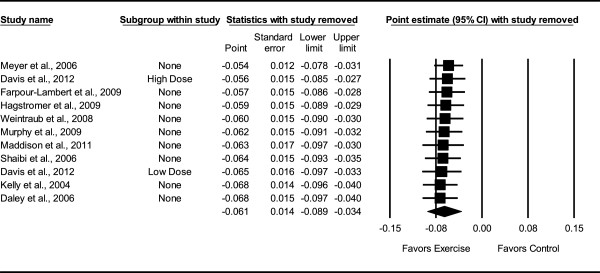
**Influence analysis for changes in BMI z-score.** Influence analysis for point estimate changes in BMI z-score with each corresponding study deleted from the model once. The black squares represent the mean difference while the left and right extremes of the squares represent the corresponding 95% confidence intervals. The middle of the black diamond represents the overall mean difference while the left and right extremes of the diamond represent the corresponding 95% confidence intervals. Results are ordered from smallest to largest reductions*.*

**Figure 6:**
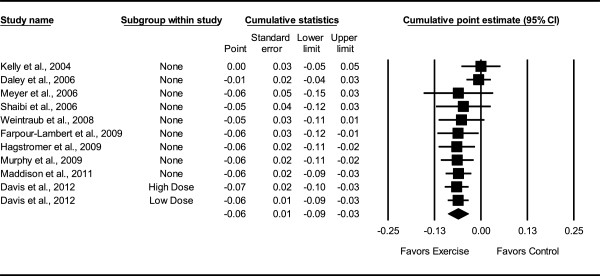
**Cumulative meta-analysis for changes in BMI z-score.** Cumulative meta-analysis, ordered by year, for point estimate changes in BMI z-score. The black squares represent the mean difference while the left and right extremes of the squares represent the corresponding 95% confidence intervals. The results of each corresponding study are pooled with all studies preceding it. The middle of the black diamond represents the overall mean difference while the left and right extremes of the diamond represent the corresponding 95% confidence intervals.

Overall, there was a statistically significant reduction in BMI z-score (Table 3 and Figure 3). This was equivalent to a relative exercise minus control group improvement of approximately 3%. Statistically significant but moderate heterogeneity was observed while 95% PIs were overlapping. No small-study effects were observed as indicated by a lack of funnel plot asymmetry (Figure 4) as well as overlapping 95% CI based on Egger’s regression intercept test (β_0_, -1.6, 95% CI, -4.1 to 1.0) [59]. Improvements in BMI z-score remained statistically significant when data were collapsed so that only one ES represented each study . With each group deleted from the model once, results remained statistically significant across all deletions (Figure 5). The difference between the largest and smallest values with each group deleted was 0.007 (11.5%). Cumulative meta-analysis, ranked by year, demonstrated that results have been statistically significant since 2009 (Figure 6). The NNT was 107 (95% CI, 209 to 73) with an estimated 116,822 (95% CI, 59,809 to 171,233) obese US children and adolescents and approximately 1 million (95% CI, 0.5 to 1.5) overweight and obese children and adolescents worldwide experiencing improvements in their BMI z-score if they began and maintained a regular exercise program. Results remained statistically significant when the two studies in which energy intake decreased were deleted from the model .

**Table 3:** **Changes in primary and secondary outcomes**

Variable	Studies (#)	ES (#)	Participants (#)		*Z*( *p*)	Q( *p*)	*I* ^*2*^ *(%)*	95% PI
Primary								
- BMI (z-score)	10	11	835	-0.06 (-0.09, -0.03)*	-4.32(<0.001)	24.9(0.01)*	59.8	-0.15, 0.02
Secondary								
- Body weight (kg)	6	6	474	-0.74 (-1.18, -0.30)*	-3.29(<0.001)	4.8(0.44)	0	-1.36, -0.12*
- BMI (kg^.^m^2^)	8	8	562	-0.47 (-0.86, -0.08)*	-2.38(0.02)	24.5(<0.001)*	71.4	-1.67, 0.73
- BMI (percentile)	4	4	104	-0.10 (-0.18, -0.02)*	-2.53(0.01)	1.1(0.78)	0	-0.27, 0.07
- Fat mass (kg)	5	5	426	-0.65 (-1.15, -0.16)*	-2.58(<0.01)	4.7(0.32)	14.9	-1.72, 0.42
- Body Fat (%)	9	9	759	-0.96 (-1.43, -0.50)*	-4.04(<0.001)	16.6(0.03)*	51.8	-2.27, 0.35
- Fat-free mass (kg)	5	5	148	-0.07 (-0.62, 0.48)	-0.26(0.79)	**10.6(** 0.03)*	62.3	--
- Waist circumference (cm)	3	3	377	0.27 (-2.08, 2.62)	0.23(0.82)	**4.7(** 0.09)*	57.5	--
- Waist-to-hip ratio	2	2	100	-0.03 (-0.09, 0.04)	-0.71(0.48)	**24.5** (<0.001)*	95.9	--
- Systolic BP (mmHg)	6	6	215	-2.66 (-7.73, 2.41)	-1.03(0.30)	**16.7(** 0.01)*	70.0	--
- Diastolic BP (mmHg)	5	5	148	-1.46 (-4.73, 1.80)	-0.88(0.38)	6.2(0.19)	35.0	--
- TC (mg/dl)	4	4	120	0.06 (-7.00, 7.12)	0.02(0.99)	2.4(0.50)	0	--
- HDL-C (mg/dl)	5	5	187	1.70 (-1.08, 4.49)	1.20(0.23)	**10.5(** 0.03)*	61.8	--
- TC:HDL-C	3	3	76	-0.22 (-0.47, 0.02)	-1.78(0.07)	0.5(0.79)	0	--
- LDL-C (mg/dl)	5	5	187	-1.53 (-8.78, 5.71)	-0.42(0.68)	**7.9(** 0.09)*	49.6	--
- TG (mg/dl)	5	5	185	**-12.46** **(-24.7, -** 0.22)*	-1.99(0.05)	1.7(0.78)	0	-32.3, 7.4
- Non-HDL-C (mg/dl)	3	3	76	0.40 (-7.67, 8.46)	0.10(0.92)	0.83(0.66)	0	--
- Fasting glucose (mg/dl)	6	6	418	-0.60 (-2.13, 0.93)	-0.77(0.44)	7.23(0.20)	30.8	--
- Fasting insulin (smd)	6	7	405	**-0.39** **(-0.57, -** 0.21)*	-4.25(<0.001)	5.93(0.43)	0	**-0.6, -** 0.15*
- VO_2max_ (ml^.^kg^-1.^min^-1^)	7	8	695	**1.92** **(0.67,** 3.17)*	3.02(<0.001)	**16.9(** 0.02)*	58.6	-1.60, 5.45
- Energy intake (kcals)	2	2	37	**-346 (-** **506, -** 188)*	-4.24(<0.001)	0.02(0.88)	0	--

Notes: #, number; ES, effect size; , mean and 95% confidence interval; Z(*p*), Z value and alpha value for Z; Q(*p*), Cochran’s Q statistic and alpha value for Q; *I* ^*2*^ (*%),* I-squared; 95% PI, 95% prediction intervals; BMI, body mass index; BP, blood pressure; TC, total cholesterol; HDL-C, high-density lipoprotein cholesterol; TC:HDL-C, ratio of total cholesterol to high-density lipoprotein cholesterol; LDL-C, low-density lipoprotein cholesterol; TG, triglycerides; Non-HDL-C, non-high-density lipoprotein cholesterol; smd, standardized mean difference; VO_2max_, maximum oxygen consumption; kcals, kilocalories;*, statistically significant; --, Not calculated; **Boldfaced** items indicate statistical significance.

Moderator analyses for changes in BMI z-score and in which sufficient data were available are shown in Additional file 5. As can be seen, no statistically significant between-group Q_b_ differences for any of the analyses were observed.

Meta-regression analyses for changes in BMI z-score and selected covariates in which sufficient data were available for are shown in Additional file 6. As can be seen, there was no statistically significant association between changes in BMI z-score and any of the covariates.

### Secondary outcomes

Changes in secondary outcomes are shown in Table 3. As can be seen, there were statistically significant reductions for body weight, BMI in kg/m^2^, BMI percentile, fat mass and percent body fat. These were equivalent to relative improvements of approximately 1%, 2%, 1%, 2% and 3%, respectively, for body weight, BMI in kg/m^2^, BMI percentile, fat mass and percent body fat. In addition, improvements were also observed for TG, fasting insulin, VO_2max_ in ml^.^kg^-1.^min^-1^, and energy intake. These were equivalent to relative improvements of approximately 13% and 7% respectively, for TG and VO_2max_ in ml^.^kg^-1.^min^-1^. There was also a statistically significant reduction of approximately 14% for energy intake. Based on the *I*^*2*^ statistic, no between-study heterogeneity was observed for body weight, BMI percentile, TG, fasting insulin and energy intake while a very low amount was observed for fat mass. Between-study heterogeneity was categorized as moderate for BMI in kg/m^2^, percent body fat and VO_2max_ in ml^.^kg^-1.^min^-1^. Statistically significant 95% PI were limited to improvements in body weight and fasting insulin. No small-study effects were observed for any of the secondary outcomes. In addition, all results remained statistically significant when ES were collapsed so that only one ES represented each study. No statistically significant differences were observed for fat-free mass, waist circumference, waist-to-hip ratio, resting systolic and diastolic blood pressure, TC, HDL-C ratio of TC to HDL-C, LDL-C, TG, non-HDL-C and fasting glucose. Insufficient data were available to calculate and pool changes in glycosylated hemoglobin, physical activity levels during the intervention period, muscular strength and energy expenditure.

## Discussion

### Overall findings

The primary purpose of this study was to use the aggregate data meta-analytic approach to determine the effects of exercise (aerobic, strength training or both) on BMI z-score in overweight and obese children and adolescents. The overall findings suggest that exercise improves BMI z-score in children and adolescents. This interpretation is supported by (1) non-overlapping 95% CI, (2) sensitivity of results with each study deleted from the model once, (3) cumulative meta-analysis, (4) absence of small-study effects, and (5) number of overweight and obese children in the US as well as worldwide who might improve their BMI z-score by initiating and maintaining a regular exercise program. In addition, the fact that statistically significant improvements were observed in both BMI z-score and percent body fat is encouraging given that BMI z-score is not the most sensitive measure of adiposity and changes may be observed in body composition variables such as percent body fat but not BMI.

While random-effects models that incorporate heterogeneity into the analysis were used, unidentified, moderate heterogeneity based on a fixed-effect model was observed for changes in BMI z-score. Consequently, it is possible that some groups may see greater or no improvements in BMI z-score. However, the existence of heterogeneity in meta-analysis is not only common [75], but also relevant, as there is no need to combine studies exactly alike since their findings, within statistical error, would be the same [76]. Caution may also be warranted with respect to the current findings given that PI for estimating the expected results of a new trial included zero for changes in BMI z-score. However, these values should not be confused with CI since PI are based on a random mean effect while CI are not [57].

No significant differences in BMI z-score were observed for any of the moderator or meta-regression analyses conducted. However, absence of evidence is not necessarily evidence of absence [77]. The lack of statistically significant and practically relevant findings may be especially important given the small number of studies and participants included in many of the covariate analyses. The former notwithstanding, the fact that no statistically significant differences in BMI z-score existed between those studies that supplied IPD versus those that did not suggests a lack of bias between the two.

The improvements in several body composition variables (body weight, BMI in kg^.^m^2^, percent body fat) observed in the current study are in agreement as well as disagreement with previous meta-analyses in which all or a majority of the participants were overweight and obese children and adolescents. For example, the statistically significant improvements observed for BMI in kg^.^m^2^ for the current meta-analysis were also reported in another meta-analysis (-0.35 kg^.^m^2^, 95% CI, -0.12 to -0.58) [29]. With respect to percent body fat and body weight, the improvements observed for percent body fat and body weight in the current study are in agreement with a previous meta-analysis with respect to percent body fat (SMD, -0.4, 95% CI, -0.7 to -0.1) but not body weight (-2.7 kg, 95% CI, -6.1 to 0.8) [4]. Potential reasons for this latter discrepancy may have to do with such things as differing inclusion criteria and possibly more importantly, differing sample sizes.

The results of this study compare favorably with the results of a recent meta-analysis on dietary interventions alone as well as combined lifestyle interventions in which the majority of the children and adolescents were overweight or obese [19]. Specifically, no statistically significant differences were observed for combined measures of adiposity for dietary interventions (SMD, -0.22, 95% CI, -0.56 to 0.11) while a statistically significant improvement was observed for combined lifestyle interventions targeting the family (SMD, -0.64, 95% CI, -0.88 to -0.39) but not children (SMD, -0.17, 95% CI, -0.40 to 0.05) [19].

The results of this study also compare favorably with pharmacologic interventions in overweight and obese children and adolescents. For example, statistically significant improvements have been observed in BMI in kg^.^m^2^ for sibutramine (-2.4 kg^.^m^2^, 95% CI, -1.8 to -3.1), and orlistat (-0.7 kg^.^m^2^, 95% CI, -0.3 to -1.2) [19]. While the findings for sibutramine are greater than the statistically significant findings in the current meta-analysis, as judged by non-overlapping CI between the two, no such between-intervention differences existed between exercise and orlistat [19]. In addition, the use of pharmacologic interventions in overweight and obese children and adolescents needs to be used with consideration for potential side-effects. Furthermore, there are numerous other potential benefits of exercise, some of which were observed in the current meta-analysis, for example, increases in cardiorespiratory fitness, which cannot be achieved with pharmacologic interventions such as sibutramine and orlistat.

### Implications for research

The results of the current systematic review with meta-analysis have at least seven implications for future research. First, based on the Cochrane Risk of Bias Instrument [44], future randomized controlled trials need to do a better job in reporting information on several potential sources of bias. This includes complete information on (1) allocation concealment, (2) blinding of outcome assessors, (3) attrition, including reasons, according to each group and (4) the physical activity levels of the participants prior to study enrollment. While all of the included studies were also considered to be at a high risk of bias for the blinding of participants and personnel category [62, 63, 67–74], it is important to realize that it is impossible to blind participants to the exercise intervention. Therefore, the best that one can probably do is blind participants to group assignment.

Second, only one study appeared to use both per-protocol and intention-to-treat approaches in the analysis of their data [62]. It is suggested that future studies include both. As a result, one may gain a better understanding of not only the efficacy (per-protocol analysis), but also the effectiveness (intention-to-treat analysis) of exercise for improving BMI z-score and other measures of adiposity in overweight and obese children and adolescents [78].

Third, given the paucity of data that were available for adverse events and the cost-effectiveness of the interventions employed, there is a need for future studies to collect and report this information. The inclusion of such is critical for those involved in deciding which interventions to recommend over others.

Fourth, it is suggested that investigators collect and report complete information on the exercise intervention(s) used. This includes data on the length, frequency, intensity and duration of exercise as well as the mode(s) used and compliance to the exercise protocol. Also, the setting in which exercise takes place (for example, home versus facility-based) and supervision status (supervised versus unsupervised) should be reported. For all groups studied, including control groups, data should also be collected and reported on the total physical activity levels of all groups during the study. The rationale for this suggestion is based on the possibility that physical activity levels beyond any intervention(s) may increase or decrease in the intervention and/or control groups. For example, one of the included studies reported that total daily physical activity in the exercise group, when compared to the control group, decreased during the study [69]. Changes such as these may negatively impact one or more of the outcomes under investigation.

Fifth, the PI reported for changes in BMI z-score may be beneficial for future researchers interested in conducting randomized controlled intervention trials addressing the effects of exercise on BMI z-score in overweight and obese children and adolescents. This is important for ensuring that appropriate power is obtained for the variable(s) of interest.

Sixth, since the dose–response effects of exercise on measures of adiposity in overweight and obese children and adolescents remain elusive, it is suggested that future randomized controlled trials address this issue. The determination of such is critical for the development of optimal exercise programs for reducing measures of adiposity in overweight and obese children and adolescents.

Seventh, the *a priori plan* of the current meta-analysis was to conduct a one-step IPD meta-analysis [46]. However, because of (1) the inability to obtain IPD from all eligible studies, (2) the inability to resolve discrepancies between the IPD provided and data reported in the published studies, for example, final sample sizes and (3) the potential loss of power with fewer included studies at the IPD level, a *post hoc* decision was made to conduct an aggregate data meta-analysis, an approach similar to conducting a two-step meta-analysis with IPD [46]. While some may consider IPD to be the gold standard [46, 79], primarily because of the potential to conduct covariate analyses at the participant level, this has to be weighed against the reality of trying to obtain valid IPD from all eligible studies as well as the fact that causal inferences based on covariate analyses, whether conducted using IPD or aggregate data, cannot be made given that experiments are never randomly assigned to covariates [66, 80]. In addition, the time and costs associated with conducting an IPD meta-analysis are substantially greater than conducting an aggregate data meta-analysis. For example, in 1997, Steinberg et al. estimated the cost for 12 ovarian cancer studies to be 5.3 times higher than conducting an IPD meta-analysis ($259,300 versus $48,665) [81]. However, it has been suggested that the real costs may be 8 times greater since the research team continued to work on the project after the money ran out [80]. Furthermore, support for the use of IPD is not always well grounded. For example, when examining for overall effects, the primary purpose of meta-analysis [76], studies claiming the superiority of IPD over aggregate data meta-analysis have been based on comparisons of a different number of studies between the two [82, 83]. In contrast, when identical or a nearly identical number of studies were included, the overall results were similar [81, 84, 85]. Finally, while the use of IPD has increased in recent years [86], the aggregate data approach is still the most common approach used when conducting a meta-analysis. Thus, while the research team agrees that an IPD meta-analysis may be the best approach in an ideal world, such an approach may not be appropriate in most real world situations in which applied meta-analysts currently reside. It is suggested that future investigators planning an IPD meta-analysis think very carefully about whether such an approach is not only feasible, but also what potential gain, if any, will be derived when compared to conducting an aggregate data meta-analysis.

### Implications for practice

The results of the current meta-analysis in overweight and obese children and adolescents have important implications for practice. For example, exercise appears to improve BMI z-score as well as several other body composition (body weight, BMI in kg/m^2^, BMI percentile, fat mass, percent body fat) and cardiovascular disease risk factors (TG, fasting insulin, VO_2_max in ml^.^kg^-1.^min^-1^) variables. While the exact dose–response effects of exercise could not be determined in the current meta-analysis and despite the lack of reporting for adverse events, it would appear plausible to suggest that overweight and obese children and adolescents follow the current guidelines for exercise in youth [87]. These include 60 or more minutes of exercise per day, most of which should be either moderate or vigorous intensity aerobic exercise (brisk walking, running, cycling, etc.), including at least 3 days per week of vigorous intensity exercise, for example, running versus walking [87]. Included in the 60 minutes per day should be muscle strengthening exercises (pushups, weight training, etc.) at least 3 days per week as well as bone strengthening exercises, for example jumping rope, at least 3 days per week [87]. These activities should be (1) enjoyable, (2) age appropriate, (3) gender appropriate and (4) varied. For previously inactive children and adolescents who are overweight or obese, a gradual increase in the volume of activity should be encouraged until these thresholds can be met. In addition, since changes in all measures of adiposity were 3% or less, the use of additional interventions such as a reduction in energy intake, along with exercise, may yield greater improvements.

### Potential strengths and limitations of current study

#### Strengths

There are least four *potential* strengths of the current meta-analysis. First, to the best of the investigative team’s knowledge, this is the first meta-analysis to focus on BMI z-score, a metric suggested to be superior to other BMI measures [31], as the primary outcome with respect to the effects of exercise in overweight and obese children and adolescents. Thus, this adds important information regarding the magnitude of benefit that exercise can provide for improving adiposity in overweight and obese children and adolescents. Second, the inclusion of the NNT provides practical information to aid decision-makers in deciding what treatments to recommend or prioritize over others when attempting to reduce adiposity in overweight and obese children and adolescents. Third, while gross estimates were provided and are prone to error, the absolute number of overweight and/or obese children and adolescents who could improve their BMI z-scores by participating in a regular exercise program can help aid decision-makers in allocating the resources necessary for accomplishing such. Fourth, the calculation and inclusion of PI can aid investigators when planning future randomized controlled trials on this topic.

### Potential limitations

The results of the current meta-analysis should be viewed with respect to the following nine *potential* limitations. First, the many different exercise modes and various intensities used in the interventions could have affected the current findings. Second, while no statistically significant association was observed between baseline BMI z-score and changes in BMI z-score, results may have nevertheless been affected by the fact that studies included overweight to morbidly obese children and adolescents [62, 63, 67–74]. Third, while we were unable to examine for such, the exercise response could have been affected by the fact that the studies included both healthy populations as well as those with cardiovascular disease risk factors [62, 63, 67–74]. Fourth, while no statistically significant association was observed between compliance or dropouts and changes in BMI z-score, it is still possible that the various levels of compliance and dropout rates could have affected the current findings.

Fifth, because studies are not randomly assigned to covariates in meta-analysis, they are considered to be observational in nature. Consequently, the results of the moderator and meta-regression analyses conducted in this or any other meta-analysis do not support causal inferences [66]. Sixth, because a large number of statistical tests were conducted and no adjustments were made for such, some statistically significant findings could have been nothing more than the play of chance. However, as suggested by Rothman [88], no adjustment was made for multiple tests because of the concern about missing possibly important findings. Seventh, while estimates regarding the number of overweight and/or obese children who could improve their BMI z-scores in the US and worldwide were provided, it’s important to understand that these were gross estimates. Most notably, it was assumed that none of the overweight and obese children and adolescents were exercising. Consequently, the estimates provided may be inflated. Eighth, the results for the secondary outcomes included in the current meta-analysis may be biased since they were only included if BMI z-score was included as an outcome. Ninth, like any meta-analysis, the results of the current investigation may be prone to both ecological fallacy and/or Simpson’s Paradox [80].

## Conclusions

Exercise improves BMI z-score in overweight and obese children and adolescents. However, based on risk of bias assessments as well as other observed factors, additional, well-designed randomized controlled trials on this topic are needed.

## Authors’ information

GAK has more than 20 years of successful experience in the design and conduct of all aspects of meta-analysis, including the effects of chronic exercise in overweight and obese children, adolescents and adults. KSK has more than 16 years of successful experience in conducting meta-analysis, including the effects of chronic exercise in overweight and obese children, adolescents and adults. RRP has been a leading authority for more than 30 years on the effects of exercise in overweight and obese children and adolescents.

## Electronic supplementary material

Additional file 1: **PRISMA checklist.** (DOC 64 KB)

Additional file 2: **Search strategies for databases searched.** (DOCX 53 KB)

Additional file 3: **Studies excluded, including reasons.** (DOCX 742 KB)

Additional file 4: **Cochrane risk of bias results for each item from each included study.** (DOCX 15 KB)

Additional file 5: **Table of categorical analyses results for BMI z-score.** (DOCX 44 KB)

Additional file 6: **Mixed effects meta-regression results for changes in BMI z-score.** (DOCX 36 KB)

## References

[1] Watts K, Jones TW, Davis EA, Green D (2005). Exercise training in obese children and adolescents: current concepts. Sports Med.

[2] Singh AS, Mulder C, Twisk JW, Van MW, Chinapaw MJ (2008). Tracking of childhood overweight into adulthood: a systematic review of the literature. Obes Rev.

[3] McGee DL (2005). Body mass index and mortality: a meta-analysis based on person-level data from twenty-six observational studies. Ann Epidemiol.

[4] Atlantis E, Barnes EH, Singh MA (2006). Efficacy of exercise for treating overweight in children and adolescents: a systematic review. Int J Obes (Lond).

[5] August GP, Caprio S, Fennoy I, Freemark M, Kaufman FR, Lustig RH, Silverstein JH, Speiser PW, Styne DM, Montori VM (2008). Prevention and treatment of pediatric obesity: an endocrine society clinical practice guideline based on expert opinion. J Clin Endocrinol Metab.

[6] Campbell K, Waters E, O’Meara S, Kelly S, Summerbell C (2002). Interventions for preventing obesity in children. Cochrane Database Syst Rev.

[7] Collins CE, Warren J, Neve M, McCoy P, Stokes BJ (2006). Measuring effectiveness of dietetic interventions in child obesity: a systematic review of randomized trials. Arch Pediatr Adolesc Med.

[8] Epstein LH, Goldfield GS (1999). Physical activity in the treatment of childhood overweight and obesity: current evidence and research issues. Med Sci Sports Exerc.

[9] Flodmark CE, Marcus C, Britton M (2006). Interventions to prevent obesity in children and adolescents: a systematic literature review. Int J Obes (Lond).

[10] Gilles A, Cassano M, Shepherd EJ, Higgins D, Hecker JE, Nangle DW (2008). Comparing active pediatric obesity treatments using meta-analysis. J Clin Child Adolesc Psychol.

[11] Gonzalez-Suarez C, Worley A, Grimmer-Somers K, Dones V (2009). School-based interventions on childhood obesity: a meta-analysis. Am J Prev Med.

[12] Harris KC, Kuramoto LK, Schulzer M, Retallack JE (2009). Effect of school-based physical activity interventions on body mass index in children: a meta-analysis. Can Med Assoc J.

[13] Hesketh KD, Campbell KJ (2010). Interventions to prevent obesity in 0–5 year olds: an updated systematic review of the literature. Obesity.

[14] Kamath CC, Vickers KS, Ehrlich A, McGovern L, Johnson J, Singhal V, Paulo R, Hettinger A, Erwin PJ, Montori VM (2008). Behavioral interventions to prevent childhood obesity: a systematic review and meta-analyses of randomized trials. J Clin Endocrinol Metab.

[15] Kanekar A, Sharma M (2008). Meta-analysis of school-based childhood obesity interventions in the U.K. and U.S. Int Q Commun Health Educ.

[16] Katz DL, O’Connell M, Njike VY, Yeh MC, Nawaz H (2008). Strategies for the prevention and control of obesity in the school setting: systematic review and meta-analysis. Int J Obes (Lond).

[17] Kelly KP, Kirschenbaum DS (2011). Immersion treatment of childhood and adolescent obesity: the first review of a promising intervention. Obes Rev.

[18] Kitzmann KM, Dalton WT, Stanley CM, Beech BM, Reeves TP, Buscemi J, Egli CJ, Gamble HL, Midgett EL (2010). Lifestyle interventions for youth who are overweight: a meta-analytic review. Health Psych.

[19] McGovern L, Johnson JN, Paulo R, Hettinger A, Singhal V, Kamath C, Erwin PJ, Montori VM (2008). Treatment of pediatric obesity: a systematic review and meta-analysis of randomized trials. J Clin Endocrinol Metab.

[20] Oude LH, Baur L, Jansen H, Shrewsbury VA, O’Malley C, Stolk RP, Summerbell CD (2009). Interventions for treating obesity in children. Cochrane Database Syst Rev.

[21] Reilly JJ, McDowell ZC (2003). Physical activity interventions in the prevention and treatment of paediatric obesity: systematic review and critical appraisal. Proc Nutr Soc.

[22] Seo DC, Sa J (2010). A meta-analysis of obesity interventions among U.S. Minority children. J Adolesc Health.

[23] Snethen JA, Broome ME, Cashin SE (2006). Effective weight loss for overweight children: a meta-analysis of intervention studies. J Pediatr Nurs.

[24] Stice E, Shaw H, Marti CN (2006). A meta-analytic review of obesity prevention programs for children and adolescents: the skinny on interventions that work. Psychol Bull.

[25] Summerbell CD, Waters E, Edmunds LD, Kelly S, Brown T, Campbell KJ (2005). Interventions for preventing obesity in children. Cochrane Database Syst Rev.

[26] Wilfley DE, Tibbs TL, Van Buren DJ, Reach KP, Walker MS, Epstein LH (2007). Lifestyle interventions in the treatment of childhood overweight: a meta-analytic review of randomized controlled trials. Health Psych.

[27] Young KM, Northern JJ, Lister KM, Drummond JA, O’Brien WH (2007). A meta-analysis of family-behavioral weight-loss treatments for children. Clin Psychol Rev.

[28] Guerra PH, Nobre MRC, da Silveira JAC, Taddei JADC (2013). The effect of school-based physical activity interventions on body mass index: a meta-analysis of randomized trials. Clinics.

[29] Lavelle HV, Mackay DF, Pell JP (2012). Systematic review and meta-analysis of school-based interventions to reduce body mass index. J Pub Health.

[30] Shea BJ, Hamel C, Wells GA, Bouter LM, Kristjansson E, Grimshaw J, Henry DA, Boers M (2009). AMSTAR is a reliable and valid measurement tool to assess the methodological quality of systematic reviews. J Clin Epidemiol.

[31] Inokuchi M, Matsuo N, Takayama JI, Hasegawa T (2011). BMI z-score is the optimal measure of annual adiposity change in elementary school children. Ann Hum Biol.

[32] Physical Activity Guidelines Advisory Committee (2008). Physical Activity Guidelines Advisory Report.

[33] Liberati A, Altman DG, Tetzlaff J, Mulrow C, Gotzsche PC, Ioannidis JP, Clarke M, Devereaux PJ, Kleijnen J, Moher D (2009). The PRISMA statement for reporting systematic reviews and meta-analyses of studies that evaluate health care interventions: explanation and elaboration. Ann Int Med.

[34] Sacks HS, Chalmers TC, Smith H (1982). Randomized versus historical controls for clinical trials. Am J Med.

[35] Schulz KF, Chalmers I, Hayes R, Altman DG (1995). Empirical evidence of bias: dimensions of methodological quality associated with estimates of treatment effects in controlled trials. JAMA.

[36] Jago R, Jonker ML, Missaghian M, Baranowski T (2006). Effect of 4 weeks of pilates on the body composition of young girls. Prev Med.

[37] Dietz WH (1998). Health consequences of obesity in youth: childhood predictors of adult disease. Pediatrics.

[38] Lee E, Dobbins M, DeCorby K, Mcrae L, Tirilis D, Husson H (2012). An optimal search filter for retrieving systematic reviews and meta-analyses. BMC Med Res Methodol.

[39] *Reference Manager. 12.0.3*. Philadelphia, PA: Thompson ResearchSoft; 2009.

[40] *Microsoft Excel*. Redmond, WA: Microsoft Corporation; 2007.

[41] Sun C, Pezic A, Tikellis G, Ponsonby AL, Wake M, Carlin JB, Cleland V, Dwyer T (2013). Effects of school-based interventions for direct delivery of physical activity on fitness and cardiometabolic markers in children and adolescents: a systematic review of randomized controlled trials. Obes Rev.

[42] Ainsworth BE, Haskell WL, Herrmann SD, Meckes N, Bassett DR, Tudor-Locke C, Greer JL, Vezina J, Whitt-Glover MC, Leon AS (2011). 2011 compendium of physical activities: a second update of codes and MET values. Med Sci Sports Exerc.

[43] Cohen J (1968). Weighted kappa: nominal scale agreement with provision for scaled disagreement or partial credit. Psychol Bull.

[44] Higgins JP, Altman DG, Gotzsche PC, Juni P, Moher D, Oxman AD, Savovic J, Schulz KF, Weeks L, Sterne JA (2011). The Cochrane Collaboration’s tool for assessing risk of bias in randomised trials. Br Med J.

[45] Ahn S, Becker BJ (2011). Incorporating quality scores in meta-analysis. J Educ Behav Stat.

[46] Riley RD, Lambert PC, Staessen JA, Wang J, Gueyffier F, Thijs L, Boutitie F (2008). Meta-analysis of continuous outcomes combining individual patient data and aggregate data. Stat Med.

[47] Follmann D, Elliot P, Suh I, Cutler J (1992). Variance imputation for overviews of clinical trials with continuous response. J Clin Epidemiol.

[48] Hedges LV, Olkin I (1985). Statistical Methods for Meta-Analysis.

[49] Dersimonian R, Laird N (1986). Meta-analysis in clinical trials. Control Clin Trials.

[50] Bergstrom I, Landgren B, Brinck J, Freyschuss B (2008). Physical training preserves bone mineral density in postmenopausal women with forearm fractures and low bone mineral density. Osteoporos Int.

[51] Kraemer HC, Kupfer DJ (2006). Size of treatment effects and their importance to clinical research and practice. Biol Psychiatry.

[52] Higgins JPT, Green S (2011). Cochrane handbook for systematic reviews of interventions version 5.1.0 [updated march 2011]. Cochrane Collaboration.

[53] Ogden CL, Carroll MD, Kit BK, Flegal KM (2014). Prevalence of childhood and adult obesity in the United States, 2011–2012. JAMA.

[54] Cali AMG, Caprio S (2008). Obesity in children and adolescents. J Clin Endocrinol Metab.

[55] Haslam DW, James WP (2005). Obesity. Lancet.

[56] Higgins JPT, Thompson SG, Deeks JJ, Altman DG (2003). Measuring inconsistency in meta-analyses. Br Med J.

[57] Higgins JP, Thompson SG, Spiegelhalter DJ (2009). A re-evaluation of random-effects meta-analysis. J R Stat Soc Ser A.

[58] Kelley GA, Kelley KS (2009). Impact of progressive resistance training on lipids and lipoproteins in adults: another look at a meta-analysis using prediction intervals. Prev Med.

[59] Egger M, Davey Smith G, Schneider M, Minder C (1997). Bias in meta-analysis detected by a simple graphical test. Br Med J.

[60] Sterne JA, Sutton AJ, Ioannidis JP, Terrin N, Jones DR, Lau J, Carpenter J, Rucker G, Harbord RM, Schmid CH, Tetzlaff J, Deeks JJ, Peters J, Macaskill P, Schwarzer G, Duval S, Altman DG, Moher D, Higgins JP (2011). Recommendations for examining and interpreting funnel plot asymmetry in meta-analyses of randomised controlled trials. Br Med J.

[61] Lau J, Schmid CH, Chalmers TC (1995). Cumulative meta-analysis of clinical trials builds evidence for exemplary medical care: the potsdam international consultation on meta-analysis. J Clin Epidemiol.

[62] Farpour-Lambert NJ, Aggoun Y, Marchand LM, Martin XE, Herrmann FR, Beghetti M (2009). Physical activity reduces systemic blood pressure and improves early markers of atherosclerosis in pre-pubertal obese children. J Am Coll Cardiol.

[63] Kelly AS, Wetzsteon RJ, Kaiser DR, Steinberger J, Bank AJ, Dengel DR (2004). Inflammation, insulin, and endothelial function in overweight children and adolescents: The role of exercise. J Pediatr.

[64] Borenstein M, Hedges L, Higgins J, Rothstein H (2009). Introduction to Meta-Analysis.

[65] American College of Sports Medicine (2006). ACSM’s Guidelines for Exercise Testing and Prescription.

[66] Littell JH, Corcoran J, Pillai V (2008). Systematic Reviews and Meta-Analysis.

[67] Daley AJ, Copeland RJ, Wright NP, Roalfe A, Wales JK (2006). Exercise therapy as a treatment for psychopathologic conditions in obese and morbidly obese adolescents: a randomized, controlled trial. Pediatrics.

[68] Davis CL, Pollock NK, Waller JL, Allison JD, Dennis BA, Bassali R, Melendez A, Boyle CA, Gower BA (2012). Exercise dose and diabetes risk in overweight and obese children: a randomized controlled trial. JAMA.

[69] Hagstromer M, Elmberg K, Marild S, Sjostrom M (2009). Participation in organized weekly physical exercise in obese adolescents reduced daily physical activity. Acta Paediatr.

[70] Maddison R, Foley L, Ni MC, Jiang Y, Jull A, Prapavessis H, Hohepa M, Rodgers A (2011). Effects of active video games on body composition: a randomized controlled trial. Am J Clin Nutr.

[71] Meyer AA, Kundt G, Lenschow U, Schuff-Werner P, Kienast W (2006). Improvement of early vascular changes and cardiovascular risk factors in obese children after a six-month exercise program. J Am Coll Cardiol.

[72] Murphy EC, Carson L, Neal W, Baylis C, Donley D, Yeater R (2009). Effects of an exercise intervention using Dance Dance Revolution on endothelial function and other risk factors in overweight children. Int J Pediatr Obes.

[73] Shaibi GQ, Cruz ML, Ball GD, Weigensberg MJ, Salem GJ, Crespo NC, Goran MI (2006). Effects of resistance training on insulin sensitivity in overweight Latino adolescent males. Med Sci Sports Exerc.

[74] Weintraub DL, Tirumalai EC, Haydel KF, Fujimoto M, Fulton JE, Robinson TN (2008). Team sports for overweight children: the stanford sports to prevent obesity randomized trial (SPORT). Arch Pediatr Adolesc Med.

[75] Engels EA, Schmid CH, Terrin N, Olkin I, Lau J (2000). Heterogeneity and statistical significance in meta-analysis: an empirical study of 125 meta-analyses. Stat Med.

[76] Glass GV, McGaw B, Smith ML (1981). Meta-Analysis in Social Research.

[77] Altman DG, Bland JM (1995). Absence of evidence is not evidence of absence. Br Med J.

[78] Katz MH (2011). Multivariable analysis: a practical guide for clinicians.

[79] Kovalchik SA (2012). Aggregate-data estimation of an individual patient data linear random effects meta-analysis with a patient covariate-treatment interaction term. Biostatistics.

[80] Cooper H, Patall EA (2009). The relative benefits of meta-analysis conducted with individual participant data versus aggregated data. Psychol Methods.

[81] Steinberg KK, Smith SJ, Stroup DF, Olkin I, Lee NC, Williamson GD, Thacker SB (1997). Comparison of effect size estimates from a meta-analysis of summary data from published studies and from a meta-analysis using individual patient data for ovarian cancer studies. Am J Epidemiol.

[82] Stewart LA, Parmar MKB (1993). Meta-analysis of the literature or of individual patient data: is there a difference?. Lancet.

[83] Jeng GT, Scott JR, Burmeister LF (1995). A comparison of meta-analytic results using literature vs individual patient data. Paternal cell immunization for recurrent miscarriage. JAMA.

[84] Olkin I, Sampson A (1998). Comparison of meta-analysis versus analysis of variance of individual patient data. Biometrics.

[85] Mathew T, Nordstrom K (1999). On the equivalence of meta-analysis using literature and using individual patient data. Biometrics.

[86] Riley RD, Lambert PC, Abo-Zaid G (2010). Meta-analysis of individual participant data: rationale, conduct, and reporting. Br Med J.

[87] Atlanta, Georgia, Centers for Disease Control and Prevention. Division of Nutrition,Physical Activity and Obesity (2014). How Much Physical Activity do Children Need? 11-9-2011.

[88] Rothman KJ (1990). No adjustments are needed for multiple comparisons. Epidemiol.

[CR89] The pre-publication history for this paper can be accessed here: http://www.biomedcentral.com/1471-2431/14/225/prepub

