# Supplementary material for: Effects of exercise on BMI z-score in overweight and obese children and adolescents: a systematic review with meta-analysis
Source: BMC Pediatr. 2014 Sep 9;14:225. doi: 10.1186/1471-2431-14-225 (PMC4180550; doi:10.1186/1471-2431-14-225)
Supplement: Supplementary file 2 — Additional file 2: Search strategies for databases searched. (DOCX 53 KB) [file 12887_2014_1161_MOESM2_ESM.docx]

Additional File 1. Search strategies for databases searched.

**1. MEDLINE Search**

Ovid Technologies, Inc. Email Service ------------------------------ Search for: 137 not 138 Results: 2403

Last Run: 8-22-12

Database: Ovid MEDLINE(R)

Search Strategy:

--------------------------------------------------------------------------------

1 test.mp. (968616)

2 exp obesity/ or obes*.mp. (183612)

3 (weight adj loss).mp. or weight loss/ (54507)

4 overweight/ (8564)

5 (fat or adipose or overweight).mp. (233422)

6 (body adj3 fat).mp. (25830)

7 body mass index/ (66964)

8 (bmi or (quetelet$ adj3 index)).mp. (59072)

9 (body adj3 mass).mp. (132339)

10 (body adj3 weight).mp. (255189)

11 (body adj3 composition).mp. (35723)

12 sendentary.mp. (3)

13 or/2-12 (658937)

14 exp child/ (1455321)

15 (kid or kids).mp. (4024)

16 adolescen*.mp. (1521392)

17 (youth or youths).mp. (33502)

18 pediatric*.mp. (190548)

19 paediatric*.mp. (35719)

20 teen*.mp. (19468)

21 tween*.mp. (5753)

22 child*.mp. (1762973)

23 or/14-22 (2588989)

24 exp exercise/ (97146)

25 (physical adj3 activity).mp. (46520)

26 (phys* adj education).mp. (13574)

27 physical exertion.mp. [mp=title, abstract, original title, name of substance word, subject heading word, protocol

supplementary concept, rare disease supplementary concept, unique identifier] (52261)

28 (physical adj3 fitness).mp. (22175)

29 (physical adj3 therap*).mp. (36268)

30 exp physical therapy modalities/ (113805)

31 exercis*.mp. (229212)

32 (aerobic* or walk* or jog* or bicycle* or dancing or dance*).mp. (133550)

33 (Strength adj3 train*).mp. (3435)

34 (weight adj3 lift*).mp. (4343)

35 ((strength or resistance or circuit or enduran* or aerob* or physic* or fit*) adj6 train$).mp. (39182)

36 (jump* adj3 rope*).mp. (39)

37 (soccer or rugby or baseball* or basketball* or swim* or hopscotch or football).mp. (39031)

38 video games/ (1526)

39 (computer adj2 games).mp. (397)

40 (Dance adj Dance adj Revolution).mp. (16)

41 (cross adj3 training).mp. (644)

42 or/24-41 (525850)

43 13 and 23 and 42 (16727)

44 43 and humans/ (16002)

45 Randomized Controlled Trials as Topic/ (82529)

46 randomized controlled trial/ (335145)

47 Random Allocation/ (75521)

48 Double Blind Method/ (116586)

49 Single Blind Method/ (16587)

50 clinical trial/ (473097)

51 clinical trial, phase i.pt. (12477)

52 clinical trial, phase ii.pt. (19921)

53 clinical trial, phase iii.pt. (7275)

54 clinical trial, phase iv.pt. (735)

55 controlled clinical trial.pt. (84929)

56 randomized controlled trial.pt. (335145)

57 multicenter study.pt. (148503)

58 clinical trial.pt. (473097)

59 exp Clinical Trials as topic/ (259800)

60 or/45-59 (930268)

61 (clinical adj trial$).ti,ab. (184312)

62 [or/61-66] (0)

63 [or/70-72] (0)

64 test.mp. (968616)

65 exp obesity/ or obes*.mp. (183612)

66 (weight adj loss).mp. or weight loss/ (54507)

67 overweight/ (8564)

68 (fat or adipose or overweight).mp. (233422)

69 (body adj3 fat).mp. (25830)

70 body mass index/ (66964)

71 (bmi or (quetelet$ adj3 index)).mp. (59072)

72 (body adj3 mass).mp. (132339)

73 (body adj3 weight).mp. (255189)

74 (body adj3 composition).mp. (35723)

75 sendentary.mp. (3)

76 or/65-75 (658937)

77 exp child/ (1455321)

78 (kid or kids).mp. (4024)

79 adolescen*.mp. (1521392)

80 (youth or youths).mp. (33502)

81 pediatric*.mp. (190548)

82 paediatric*.mp. (35719)

83 teen*.mp. (19468)

84 tween*.mp. (5753)

85 child*.mp. (1762973)

86 or/77-85 (2588989)

87 exp exercise/ (97146)

88 (physical adj3 activity).mp. (46520)

89 (phys* adj education).mp. (13574)

90 physical exertion.mp. [mp=title, abstract, original title, name of substance word, subject heading word, protocol

supplementary concept, rare disease supplementary concept, unique identifier] (52261)

91 (physical adj3 fitness).mp. (22175)

92 (physical adj3 therap*).mp. (36268)

93 exp physical therapy modalities/ (113805)

94 exercis*.mp. (229212)

95 (aerobic* or walk* or jog* or bicycle* or dancing or dance*).mp. (133550)

96 (Strength adj3 train*).mp. (3435)

97 (weight adj3 lift*).mp. (4343)

98 ((strength or resistance or circuit or enduran* or aerob* or physic* or fit*) adj6 train$).mp. (39182)

99 (jump* adj3 rope*).mp. (39)

100 (soccer or rugby or baseball* or basketball* or swim* or hopscotch or football).mp. (39031)

101 video games/ (1526)

102 (computer adj2 games).mp. (397)

103 (Dance adj Dance adj Revolution).mp. (16)

104 (cross adj3 training).mp. (644)

105 or/87-104 (525850)

106 76 and 86 and 105 (16727)

107 106 and humans/ (16002)

108 Randomized Controlled Trials as Topic/ (82529)

109 randomized controlled trial/ (335145)

110 Random Allocation/ (75521)

111 Double Blind Method/ (116586)

112 Single Blind Method/ (16587)

113 clinical trial/ (473097)

114 clinical trial, phase i.pt. (12477)

115 clinical trial, phase ii.pt. (19921)

116 clinical trial, phase iii.pt. (7275)

117 clinical trial, phase iv.pt. (735)

118 controlled clinical trial.pt. (84929)

119 randomized controlled trial.pt. (335145)

120 multicenter study.pt. (148503)

121 clinical trial.pt. (473097)

122 exp Clinical Trials as topic/ (259800)

123 or/108-122 (930268)

124 (clinical adj trial$).ti,ab. (184312)

125 ((singl$ or doubl$ or treb$ or tripl$) adj (blind$3 or mask$3)).ti,ab. (117936)

126 PLACEBOS/ (31243)

127 placebo$.ti,ab. (143628)

128 randomly allocated.ti,ab. (14691)

129 (allocated adj2 random$).ti,ab. (17093)

130 or/124-129 (372220)

131 123 or 130 (1052880)

132 107 and 131 (2516)

133 (case adj1 report).mp. (183828)

134 letter/ (774742)

135 historical article/ (285838)

136 or/133-135 (1233762)

137 132 not 136 (2510)

138 (college or universit*).ti,ab. (269256)

139 137 not 138 (2403)

**2. CINAHL Search**

Ovid Technologies, Inc. Email Service ------------------------------ Search for: 73 not 74 Results: 321

Last Run: 8-22-12

CINAHL

Database: Ovid Nursing Database <1946 to August Week 2 2012> Search Strategy:

--------------------------------------------------------------------------------

1 exp obesity/ or obes*.mp. (11523)

2 (weight adj loss).mp. or weight loss/ (3579)

3 overweight/ (0)

4 (fat or adipose or overweight).mp. (12195)

5 (body adj3 fat).mp. (2501)

6 body mass index/ (6402)

7 (bmi or (quetelet$ adj3 index)).mp. (2653)

8 (body adj3 mass).mp. (8267)

9 (body adj3 weight).mp. (7107)

10 (body adj3 composition).mp. (4521)

11 sendentary.mp. (1)

12 or/1-11 (30577)

13 exp child/ (111361)

14 (kid or kids).mp. (570)

15 adolescen*.mp. (55184)

16 (youth or youths).mp. (3214)

17 pediatric*.mp. (23451)

18 paediatric*.mp. (2320)

19 teen*.mp. (2315)

20 tween*.mp. (16)

21 child*.mp. (101105)

22 or/13-21 (155821)

23 exp exercise/ (39707)

24 (physical adj3 activity).mp. (9455)

25 (phys* adj education).mp. (2283)

26 physical exertion.mp. [mp=title, original title, abstract, rare disease supplementary concept, protocol

supplementary concept, name of substance word, subject heading word, mesh heading word] (2586)

27 (physical adj3 fitness).mp. (3324)

28 (physical adj3 therap*).mp. (5361)

29 exp physical therapy modalities/ (28086)

30 exercis*.mp. (35709)

31 (aerobic* or walk* or jog* or bicycle* or dancing or dance*).mp. (12127)

32 (Strength adj3 train*).mp. (1541)

33 (weight adj3 lift*).mp. (1263)

34 ((strength or resistance or circuit or enduran* or aerob* or physic* or fit*) adj6 train$).mp. (8745)

35 (jump* adj3 rope*).mp. (20)

36 (soccer or rugby or baseball* or basketball* or swim* or hopscotch or football).mp. (5140)

37 video games/ (172)

38 (computer adj2 games).mp. (32)

39 (Dance adj Dance adj Revolution).mp. (7)

40 (cross adj3 training).mp. (188)

41 or/23-40 (72532)

42 12 and 22 and 41 (2558)

43 42 and humans.mp. [mp=title, original title, abstract, rare disease supplementary concept, protocol supplementary

concept, name of substance word, subject heading word, mesh heading word] (1953)

44 Randomized Controlled Trials as Topic/ (0)

45 randomized controlled trial/ (12950)

46 Random Allocation/ (1545)

47 Double Blind Method/ (367)

48 Single Blind Method/ (0)

49 clinical trial/ (12950)

50 clinical trial, phase i.pt. (77)

51 clinical trial, phase ii.pt. (145)

52 clinical trial, phase iii.pt. (93)

53 clinical trial, phase iv.pt. (19)

54 controlled clinical trial.pt. (3169)

55 randomized controlled trial.pt. (13589)

56 multicenter study.pt. (6469)

57 clinical trial.pt. (13631)

58 exp Clinical Trials as topic/ (0)

59 or/44-58 (35153)

60 (clinical adj trial$).ti,ab. (5331)

61 ((singl$ or doubl$ or treb$ or tripl$) adj (blind$3 or mask$3)).ti,ab. (3312)

62 PLACEBOS/ (3573)

63 placebo$.ti,ab. (3920)

64 randomly allocated.ti,ab. (581)

65 (allocated adj2 random$).ti,ab. (615)

66 or/60-65 (10683)

67 59 or 66 (36709)

68 43 and 67 (344)

69 (case adj1 report).mp. (3720)

70 letter/ (4841)

71 historical article/ (0)

72 or/69-71 (8548)

73 68 not 72 (343)

74 (universiti* or college or colleges).ti,ab. (10585)

75 73 not 74 (321)

**3. Scopus Search**

**Run date: 08/27/12 (215 Hits)**

1. TITLE-ABS-KEY (obesity OR obese OR sedentary OR overweight OR FAT OR Adipose OR adiposity OR (over w/3 weight)
2. TITLE-ABS-KEY “weight loss” OR “body mass”
3. TITLE-ABS-KEY **“**body mass index” OR bmi
4. TITLE-ABS-KEY quetelet$ w/3 index
5. TITLE-ABS-KEY (fat OR mass OR weight OR composition) w/3 body
6. #1 OR #2 OR #3 OR #4 OR #5
7. TITLE-ABS-KEY (child OR children OR kid OR kids OR youth OR youths OR pediatric* OR paediatrics OR teen OR teens OR teenager* OR tween OR tweens)
8. #6 AND #7
9. TITLE-ABS-KEY (exercising OR exercised OR exercise OR exertion OR aerobic* OR walk* OR jogging OR jogger OR runner OR runs OR bicycle* OR dancing OR dancer OR dances OR soccer OR rugby OR baseball* OR basketball* OR swim* OR hopscotch OR football)
10. TITLE-ABS-KEY (activit* OR educat* OR fitness OR therapy OR therapies OR therapeutic*) w/3 physical
11. KEY “physical exertion” OR “ physical therapy modalities”
12. TITLE-ABS-KEY (weight w/3 lift*)
13. TITLE-ABS-KEY (strength OR resistance OR circuit OR enduran* OR aerob* OR physical OR fit OR fitness) w/6 train*
14. TITLE-ABS-KEY (jump* w/3 rope*)
15. TITLE-ABS-KEY “video games” OR “computer games”
16. TITLE-ABS-KEY “Dance Dance Revolution”
17. TITLE-ABS-KEY train* w3 cross
18. # 9 OR #10 OR #11 OR #12 OR #13 OR #14 OR #15 OR #16 OR #17
19. #8 AND #18
20. TITLE-ABS-KEY (Human OR HUMANS)
21. #19 AND #20
22. KEY ( Randomized Controlled Trials as Topic)
23. KEY (randomized controlled trial)
24. KEY (Random Allocation)
25. KEY (Double Blind Method)
26. KEY (Single Blind Method)
27. KEY (clinical trial)
28. KEY (clinical trial, phase i)
29. KEY (clinical trial, phase ii)
30. . KEY (clinical trial, phase iii)
31. KEY (clinical trial, phase iv)
32. KEY (controlled clinical trial)
33. KEY (multicenter study)
34. KEY (clinical trials)
35. KEY (Clinical Trials as topic)
36. #/22 OR #23 OR #24 OR #25 OR #26 OR #27 OR #28 OR #29 OR #30 OR #31 OR #32 OR #33 OR #34 OR #35
37. TITLE-ABS-KEY(placebo* OR (clinical w/o trial*))
38. (singl* OR doubl* OR treb* OR tripl*) w/0 blind*
39. (singl* OR doubl* OR treb* OR tripl*) w/0 Mask
40. TITLE-ABS-KEY (Randomly w/3 allocat*)
41. TITLE-ABS (allocated w/2 random*)
42. #37 OR #38 OR #39 OR #41
43. #36 OR #42
44. #43 AND #8
45. TITLE-ABS-KEY (case w/2 report)
46. DOCTYPE (le)
47. KEY (historical article)
48. 45 OR 46 OR 47
49. 44 AND NOT48
50. TITLE-ABS-KEY (college OR colleges OR university OR universities).
51. 49 AND NOT 50
52. Limit to 1990-present

**4. Academic Search Complete**

EBSCOhost: Print Search HistoryLoading...580 HITS

Academic Search Complete Search Strategy

Revised Date: 07/2011 Accessibility Information and TipsPrint Search History

Friday, August 31, 2012 10:43:53 AM

# Query Limiters/Expanders Last Run Via Results Action

S52 s8 AND s51 Limiters - Published Date from: 19900101-20121231

Search modes - Find all my search terms Interface - EBSCOhost

Search Screen - Advanced Search

Database - Academic Search Complete;SPORTDiscus with Full Text 722 Edit

S52

S51 S48 NOT S49 Limiters - Published Date from: 19900101-20121231

Search modes - Find all my search terms Interface - EBSCOhost

Search Screen - Basic Search

Database - Academic Search Complete Display Edit S51

S50 S48 NOT S49 Search modes - Find all my search terms Interface -

EBSCOhost

Search Screen - Basic Search

Database - Academic Search Complete Display Edit S50

S49 TX college OR colleges OR university OR universities Search modes -

Find all my search terms Interface - EBSCOhost

Search Screen - Basic Search

Database - Academic Search Complete Display Edit S49

S48 S43 NOT S47 Search modes - Find all my search terms Interface -

EBSCOhost

Search Screen - Basic Search

Database - Academic Search Complete Display Edit S48

S47 S44 or S45 or S46 Search modes - Find all my search terms Interface -

EBSCOhost

Search Screen - Basic Search

Database - Academic Search Complete Display Edit S47

S46 su "historical article" Search modes - SmartText Searching Interface -

EBSCOhost

Search Screen - Basic Search

Database - Academic Search Complete Display Edit S46

S45 PT letter Search modes - SmartText Searching Interface - EBSCOhost

Search Screen - Basic Search

Database - Academic Search Complete Display Edit S45

S44 TX case w/2 report Search modes - Find all my search terms Interface -

EBSCOhost

Search Screen - Basic Search

Database - Academic Search Complete Display Edit S44

S43 S41 AND (S6 and S20) Search modes - Find all my search terms Interface

- EBSCOhost

Search Screen - Basic Search

Database - Academic Search Complete Display Edit S43

S42 S35 or S41 Search modes - Find all my search terms Interface -

EBSCOhost

Search Screen - Basic Search

Database - Academic Search Complete Display Edit S42

S41 S36 or S37 or S38 or S39 or S40 Search modes - Find all my search

terms Interface - EBSCOhost

Search Screen - Basic Search

Database - Academic Search Complete Display Edit S41

S40 TX allocated n/2 random* Search modes - Find all my search terms

Interface - EBSCOhost

Search Screen - Basic Search

Database - Academic Search Complete Display Edit S40

S39 TX Randomly n/3 allocat* Search modes - Find all my search terms

Interface - EBSCOhost

Search Screen - Basic Search

Database - Academic Search Complete Display Edit S39

S38 (singl* w/1 mask*) OR (doubl* w/1 mask*) OR (treb* w/1 mask*) OR

(tripl* w/1 mask*) Search modes - Find all my search terms Interface -

EBSCOhost

Search Screen - Basic Search

Database - Academic Search Complete Display Edit S38

S37 (singl* w/1 blind*) OR (doubl* w/1 blind*) OR (treb* w/1 blind*) OR

(tripl* w/1 blind*) Search modes - Find all my search terms Interface -

EBSCOhost

Search Screen - Basic Search

Database - Academic Search Complete Display Edit S37

S36 TX placebo* OR (clinical w/1 trial*) Search modes - Find all my search

terms Interface - EBSCOhost

Search Screen - Basic Search

Database - Academic Search Complete Display Edit S36

S35 S21 or S22 or S23 or S24 or S25 or S26 or S28 or S29 or S30 or S31 or

S32 or S33 or S34 Search modes - Find all my search terms Interface -

EBSCOhost

Search Screen - Basic Search

Database - Academic Search Complete Display Edit S35

S34 SU “clinical Trials as topic” Search modes - SmartText Searching

Interface - EBSCOhost

Search Screen - Basic Search

Database - Academic Search Complete Display Edit S34

S33 su clinical trials Search modes - Find all my search terms Interface -

EBSCOhost

Search Screen - Basic Search

Database - Academic Search Complete Display Edit S33

S32 su "multicenter study" Search modes - Find all my search terms

Interface - EBSCOhost

Search Screen - Basic Search

Database - Academic Search Complete Display Edit S32

S31 SU “controlled clinical trial” Search modes - SmartText Searching

Interface - EBSCOhost

Search Screen - Basic Search

Database - Academic Search Complete Display Edit S31

S30 SU "clinical trial, phase iv" Search modes - SmartText Searching

Interface - EBSCOhost

Search Screen - Basic Search

Database - Academic Search Complete Display Edit S30

S29 SU "clinical trial, phase iii" Search modes - SmartText Searching

Interface - EBSCOhost

Search Screen - Basic Search

Database - Academic Search Complete Display Edit S29

S28 SU clinical trial, phase ii Search modes - SmartText Searching

Interface - EBSCOhost

Search Screen - Basic Search

Database - Academic Search Complete Display Edit S28

S27 SU clinical trial, phase i Search modes - SmartText Searching

Interface - EBSCOhost

Search Screen - Basic Search

Database - Academic Search Complete Display Edit S27

S26 SU clinical trial Search modes - Find all my search terms Interface -

EBSCOhost

Search Screen - Basic Search

Database - Academic Search Complete Display Edit S26

S25 su single blind method Search modes - Find all my search terms

Interface - EBSCOhost

Search Screen - Basic Search

Database - Academic Search Complete Display Edit S25

S24 SU Double Blind method Search modes - SmartText Searching Interface -

EBSCOhost

Search Screen - Basic Search

Database - Academic Search Complete Display Edit S24

S23 SU “Random Allocation” Search modes - SmartText Searching Interface -

EBSCOhost

Search Screen - Basic Search

Database - Academic Search Complete Display Edit S23

S22 SU randomized controlled trial Search modes - Find all my search terms

Interface - EBSCOhost

Search Screen - Basic Search

Database - Academic Search Complete Display Edit S22

S21 SU Randomized Controlled Trials as Topic Search modes - Find all my

search terms Interface - EBSCOhost

Search Screen - Basic Search

Database - Academic Search Complete Display Edit S21

S20 S18 and S19 Search modes - Find all my search terms Interface -

EBSCOhost

Search Screen - Basic Search

Database - Academic Search Complete Display Edit S20

S19 TX (Human OR HUMANS) OR SU (HUMAN OR HUMANS) Search modes - Find all

my search terms Interface - EBSCOhost

Search Screen - Basic Search

Database - Academic Search Complete Display Edit S19

S18 S9 OR S10 OR S11 OR S12 OR S13 OR S14 OR S15 OR S16 OR S17 Search

modes - Find all my search terms Interface - EBSCOhost

Search Screen - Basic Search

Database - Academic Search Complete Display Edit S18

S17 TX cross n/3 train* Search modes - Find all my search terms Interface

- EBSCOhost

Search Screen - Basic Search

Database - Academic Search Complete Display Edit S17

S16 TX “Dance Dance Revolution" Search modes - Find all my search terms

Interface - EBSCOhost

Search Screen - Basic Search

Database - Academic Search Complete Display Edit S16

S15 TX “video games” OR “computer games” Search modes - Find all my search

terms Interface - EBSCOhost

Search Screen - Basic Search

Database - Academic Search Complete Display Edit S15

S14 TX (jump* n/3 rope*) Search modes - Find all my search terms Interface

- EBSCOhost

Search Screen - Basic Search

Database - Academic Search Complete Display Edit S14

S13 TX (strength n/6 train*) OR (resistance n/6 train*) OR (circuit n/6

train*) OR (enduran* n/6 train*) OR (aerob* n/6 train*) OR (physical n/6

train*) OR (fit n/6 train*) OR (fitness n/6 train*) Search modes - Find

all my search terms Interface - EBSCOhost

Search Screen - Basic Search

Database - Academic Search Complete Display Edit S13

S12 TX (weight n/3 lift*) Search modes - Find all my search terms

Interface - EBSCOhost

Search Screen - Basic Search

Database - Academic Search Complete Display Edit S12

S11 SU “physical exertion” OR “ physical therapy modalities” Search modes

- Find all my search terms Interface - EBSCOhost

Search Screen - Basic Search

Database - Academic Search Complete Display Edit S11

S10 TX (activit* n/3 physical) OR (educat* n/3 physical) OR (fitness n/3

physical) OR (therapy n/3 physical) OR (therapies n/3 physical) OR

(therapeutic n/3 physical) Search modes - Find all my search terms

Interface - EBSCOhost

Search Screen - Basic Search

Database - Academic Search Complete Display Edit S10

S9 TX bic* OR walk* OR jogging OR jogger OR runner OR runs OR bicycle* OR

dancing OR dancer OR dances OR soccer OR rugby OR baseball* OR basketball*

OR swim* OR hopscotch OR football Search modes - Find all my search terms

Interface - EBSCOhost

Search Screen - Basic Search

Database - Academic Search Complete Display Edit S9

S8 (S6 and S7) Search modes - Find all my search terms Interface -

EBSCOhost

Search Screen - Basic Search

Database - Academic Search Complete Display Edit S8

S7 TX child OR children OR kid OR kids OR youth OR youths OR pediatric* OR

paediatrics OR teen OR teens OR teenager* OR tween OR tweens Search modes

- Find all my search terms Interface - EBSCOhost

Search Screen - Basic Search

Database - Academic Search Complete Display Edit S7

S6 S1 OR S2 OR S3 OR S4 OR S5 Search modes - Find all my search terms

Interface - EBSCOhost

Search Screen - Basic Search

Database - Academic Search Complete Display Edit S6

S5 TX (fat n/3 body) OR (mass n/3 body) OR (weight n/3 body) OR

(composition n/3 body) Search modes - Find all my search terms Interface -

EBSCOhost

Search Screen - Basic Search

Database - Academic Search Complete Display Edit S5

S4 TX quetelet* n/3 index Search modes - Find all my search terms

Interface - EBSCOhost

Search Screen - Basic Search

Database - Academic Search Complete Display Edit S4

S3 TX “body mass index” OR bmi Search modes - Find all my search terms

Interface - EBSCOhost

Search Screen - Basic Search

Database - Academic Search Complete Display Edit S3

S2 TX “weight loss” OR “body mass” Search modes - Find all my search terms

Interface - EBSCOhost

Search Screen - Basic Search

Database - Academic Search Complete Display Edit S2

S1 TX obesity OR obese OR sedentary OR overweight OR FAT OR Adipose OR

adiposity OR (over n/3 weight) Search modes - Find all my search terms

Interface - EBSCOhost

Search Screen - Basic Search

Database - Academic Search Complete Display Edit S1

**5. Education Research Complete**

**Education Research Complete**

**Run: September 4, 2012**

**95 hits**

EBSCOhost: Print Search History

Loading...

Print Search History

Tuesday, September 04, 2012 3:53:13 PM

#

Query

Limiters/Expanders

Last Run Via

Results

Action

S52

Limiters - Published Date from: 19900101-20121231

Search modes - Find all my search terms

Interface - EBSCOhost

Search Screen - Basic Search

Database - Academic Search Complete

Display

EditS52

S51

Limiters - Published Date from: 19900101-20121231

Search modes - Find all my search terms

Interface - EBSCOhost

Search Screen - Basic Search

Database - Academic Search Complete

Display

EditS51

S50

S48 NOT S49

Search modes - Find all my search terms

Interface - EBSCOhost

Search Screen - Basic Search

Database - Academic Search Complete

Display

EditS50

S49

TX college OR colleges OR university OR universities

Search modes - Find all my search terms

Interface - EBSCOhost

Search Screen - Basic Search

Database - Academic Search Complete

Display

EditS49

S48

S43 NOT S47

Search modes - Find all my search terms

Interface - EBSCOhost

Search Screen - Basic Search

Database - Academic Search Complete

Display

EditS48

S47

S44 OR S45 OR S46

Search modes - Find all my search terms

Interface - EBSCOhost

Search Screen - Basic Search

Database - Academic Search Complete

Display

EditS47

S46

SU historical article

Search modes - SmartText Searching

Interface - EBSCOhost

Search Screen - Basic Search

Database - Academic Search Complete

Display

EditS46

S45

TX letter OR PT letter

Search modes - SmartText Searching

Interface - EBSCOhost

Search Screen - Basic Search

Database - Academic Search Complete

Display

EditS45

S44

TX case w/2 report

Search modes - Find all my search terms

Interface - EBSCOhost

Search Screen - Basic Search

Database - Academic Search Complete

Display

EditS44

S43

S41 AND (S6 AND S20)

Search modes - Find all my search terms

Interface - EBSCOhost

Search Screen - Basic Search

Database - Academic Search Complete

Display

EditS43

S42

S35 OR S41

Search modes - Find all my search terms

Interface - EBSCOhost

Search Screen - Basic Search

Database - Academic Search Complete

Display

EditS42

S41

S36 OR S37 OR S38 OR S39 OR S40

Search modes - Find all my search terms

Interface - EBSCOhost

Search Screen - Basic Search

Database - Academic Search Complete

Display

EditS41

S40

TX allocated n/2 random*

Search modes - Find all my search terms

Interface - EBSCOhost

Search Screen - Basic Search

Database - Academic Search Complete

Display

EditS40

S39

TX Randomly n/3 allocat*

Search modes - Find all my search terms

Interface - EBSCOhost

Search Screen - Basic Search

Database - Academic Search Complete

Display

EditS39

S38

(singl* w/1 mask*) OR (doubl* w/1 mask*) OR (treb* w/1 mask*) OR (tripl* w/1

mask*)

Search modes - Find all my search terms

Interface - EBSCOhost

Search Screen - Basic Search

Database - Academic Search Complete

Display

EditS38

S37

(singl* w/1 blind*) OR (doubl* w/1 blind*) OR (treb* w/1 blind*) OR (tripl* w/1

blind*)

Search modes - Find all my search terms

Interface - EBSCOhost

Search Screen - Basic Search

Database - Academic Search Complete

Display

EditS37

S36

TX placebo* OR (clinical w/1 trial*)

Search modes - Find all my search terms

Interface - EBSCOhost

Search Screen - Basic Search

Database - Academic Search Complete

Display

EditS36

S35

S21 OR S22 OR S23 OR S24 OR S25 OR S26 OR S28 OR S29 OR S30 OR S31 OR S32 OR S33

OR S34

Search modes - Find all my search terms

Interface - EBSCOhost

Search Screen - Basic Search

Database - Academic Search Complete

Display

EditS35

S34

DE “clinical Trials as topic”

Search modes - SmartText Searching

Interface - EBSCOhost

Search Screen - Basic Search

Database - Academic Search Complete

Display

EditS34

S33

DE clinical trials

Search modes - Find all my search terms

Interface - EBSCOhost

Search Screen - Basic Search

Database - Academic Search Complete

Display

EditS33

S32

DE multicenter study

Search modes - Find all my search terms

Interface - EBSCOhost

Search Screen - Basic Search

Database - Academic Search Complete

Display

EditS32

S31

DE controlled clinical trial

Search modes - SmartText Searching

Interface - EBSCOhost

Search Screen - Basic Search

Database - Academic Search Complete

Display

EditS31

S30

DE "clinical trial, phase iv"

Search modes - SmartText Searching

Interface - EBSCOhost

Search Screen - Basic Search

Database - Academic Search Complete

Display

EditS30

S29

DE "clinical trial, phase iii"

Search modes - SmartText Searching

Interface - EBSCOhost

Search Screen - Basic Search

Database - Academic Search Complete

Display

EditS29

S28

DE "clinical trial, phase ii"

Search modes - SmartText Searching

Interface - EBSCOhost

Search Screen - Basic Search

Database - Academic Search Complete

Display

EditS28

S27

DE "clinical trial, phase i"

Search modes - SmartText Searching

Interface - EBSCOhost

Search Screen - Basic Search

Database - Academic Search Complete

Display

EditS27

S26

DE clinical trial

Search modes - Find all my search terms

Interface - EBSCOhost

Search Screen - Basic Search

Database - Academic Search Complete

Display

EditS26

S25

DE single blind method

Search modes - Find all my search terms

Interface - EBSCOhost

Search Screen - Basic Search

Database - Academic Search Complete

Display

EditS25

S24

DE Double Blind method

Search modes - SmartText Searching

Interface - EBSCOhost

Search Screen - Basic Search

Database - Academic Search Complete

Display

EditS24

S23

DE “Random Allocation”

Search modes - SmartText Searching

Interface - EBSCOhost

Search Screen - Basic Search

Database - Academic Search Complete

Display

EditS23

S22

DE randomized controlled trial

Search modes - Find all my search terms

Interface - EBSCOhost

Search Screen - Basic Search

Database - Academic Search Complete

Display

EditS22

S21

DE Randomized Controlled Trials as Topic

Search modes - Find all my search terms

Interface - EBSCOhost

Search Screen - Basic Search

Database - Academic Search Complete

Display

EditS21

S20

S18 AND S19

Search modes - Find all my search terms

Interface - EBSCOhost

Search Screen - Basic Search

Database - Academic Search Complete

Display

EditS20

S19

TX (Human OR HUMANS) OR SU (HUMAN OR HUMANS)

Search modes - Find all my search terms

Interface - EBSCOhost

Search Screen - Basic Search

Database - Academic Search Complete

Display

EditS19

S18

S9 OR S10 OR S11 OR S12 OR S13 OR S14 OR S15 OR S16 OR S17

Search modes - Find all my search terms

Interface - EBSCOhost

Search Screen - Basic Search

Database - Academic Search Complete

Display

EditS18

S17

TX cross n/3 train*

Search modes - Find all my search terms

Interface - EBSCOhost

Search Screen - Basic Search

Database - Academic Search Complete

Display

EditS17

S16

TX “Dance Dance Revolution"

Search modes - Find all my search terms

Interface - EBSCOhost

Search Screen - Basic Search

Database - Academic Search Complete

Display

EditS16

S15

TX “video games” OR “computer games”

Search modes - Find all my search terms

Interface - EBSCOhost

Search Screen - Basic Search

Database - Academic Search Complete

Display

EditS15

S14

TX (jump* n/3 rope*)

Search modes - Find all my search terms

Interface - EBSCOhost

Search Screen - Basic Search

Database - Academic Search Complete

Display

EditS14

S13

TX (strength n/6 train*) OR (resistance n/6 train*) OR (circuit n/6 train*) OR

(enduran* n/6 train*) OR (aerob* n/6 train*) OR (physical n/6 train*) OR (fit

n/6 train*) OR (fitness n/6 train*)

Search modes - Find all my search terms

Interface - EBSCOhost

Search Screen - Basic Search

Database - Academic Search Complete

Display

EditS13

S12

TX (weight n/3 lift*)

Search modes - Find all my search terms

Interface - EBSCOhost

Search Screen - Basic Search

Database - Academic Search Complete

Display

EditS12

S11

DE physical exertion OR physical therapy modalities

Search modes - Find all my search terms

Interface - EBSCOhost

Search Screen - Basic Search

Database - Academic Search Complete

Display

EditS11

S10

TX (activit* n/3 physical) OR (educat* n/3 physical) OR (fitness n/3 physical)

OR (therapy n/3 physical) OR (therapies n/3 physical) OR (therapeutic n/3

physical)

Search modes - Find all my search terms

Interface - EBSCOhost

Search Screen - Basic Search

Database - Academic Search Complete

Display

EditS10

S9

TX bic* OR walk* OR jogging OR jogger OR runner OR runs OR bicycle* OR dancing

OR dancer OR dances OR soccer OR rugby OR baseball* OR basketball* OR swim* OR

hopscotch OR football

Search modes - Find all my search terms

Interface - EBSCOhost

Search Screen - Basic Search

Database - Academic Search Complete

Display

EditS9

S8

S6 AND S7

Search modes - Find all my search terms

Interface - EBSCOhost

Search Screen - Basic Search

Database - Academic Search Complete

Display

EditS8

S7

TX child OR children OR kid OR kids OR youth OR youths OR pediatric* OR

paediatrics OR teen OR teens OR teenager* OR tween OR tweens

Search modes - Find all my search terms

Interface - EBSCOhost

Search Screen - Basic Search

Database - Academic Search Complete

Display

EditS7

S6

S1 OR S2 OR S3 OR S4 OR S5

Search modes - Find all my search terms

Interface - EBSCOhost

Search Screen - Basic Search

Database - Academic Search Complete

Display

EditS6

S5

TX (fat n/3 body) OR (mass n/3 body) OR (weight n/3 body) OR (composition n/3

body)

Search modes - Find all my search terms

Interface - EBSCOhost

Search Screen - Basic Search

Database - Academic Search Complete

Display

EditS5

S4

TX quetelet* n/3 index

Search modes - Find all my search terms

Interface - EBSCOhost

Search Screen - Basic Search

Database - Academic Search Complete

Display

EditS4

S3

TX “body mass index” OR bmi

Search modes - Find all my search terms

Interface - EBSCOhost

Search Screen - Basic Search

Database - Academic Search Complete

Display

EditS3

S2

TX “weight loss” OR “body mass”

Search modes - Find all my search terms

Interface - EBSCOhost

Search Screen - Basic Search

Database - Academic Search Complete

Display

EditS2

S1

TX obesity OR obese OR sedentary OR overweight OR FAT OR Adipose OR adiposity OR

(over n/3 weight)

Search modes - Find all my search terms

Interface - EBSCOhost

Search Screen - Basic Search

Database - Academic Search Complete

Display

EditS1

**6. Web of Science**

Web of Science® Search History

Run: September 10, 2012

660 citations

# 3 660 Topic=(obesity OR obese OR sedentary OR overweight OR FAT OR Adipose OR adiposity OR BMI OR weight) AND Topic=(child OR children OR kid OR kids OR youth OR youths OR pediatric OR paediatrics OR teen OR teens OR teenager OR tween OR tweens OR teenagers) AND Topic=(exercise OR sports OR walking OR jogging OR running OR bicycling OR dancing OR soccer OR rugby OR baseball OR basketball OR swiming OR hopscotch OR football OR training OR fitness OR lifting OR physical OR games) AND Topic=(human OR humans)

Refined by: Document Types=( ARTICLE OR REVIEW OR PROCEEDINGS PAPER ) AND [excluding] Document Types=( REVIEW OR PROCEEDINGS PAPER OR BOOK CHAPTER )

Databases=SCI-EXPANDED, SSCI, A&HCI, CPCI-S, CPCI-SSH Timespan=1990-01-01 - 2012-09-10

Lemmatization=On Select to combine sets. Select to delete this set.

# 2 869 Topic=(obesity OR obese OR sedentary OR overweight OR FAT OR Adipose OR adiposity OR BMI OR weight) AND Topic=(child OR children OR kid OR kids OR youth OR youths OR pediatric OR paediatrics OR teen OR teens OR teenager OR tween OR tweens OR teenagers) AND Topic=(exercise OR sports OR walking OR jogging OR running OR bicycling OR dancing OR soccer OR rugby OR baseball OR basketball OR swiming OR hopscotch OR football OR training OR fitness OR lifting OR physical OR games) AND Topic=(human OR humans)

Refined by: Document Types=( ARTICLE OR REVIEW OR PROCEEDINGS PAPER )

Databases=SCI-EXPANDED, SSCI, A&HCI, CPCI-S, CPCI-SSH Timespan=1990-01-01 - 2012-09-10

Lemmatization=On Select to combine sets. Select to delete this set.

# 1 881 Topic=(obesity OR obese OR sedentary OR overweight OR FAT OR Adipose OR adiposity OR BMI OR weight) AND Topic=(child OR children OR kid OR kids OR youth OR youths OR pediatric OR paediatrics OR teen OR teens OR teenager OR tween OR tweens OR teenagers) AND Topic=(exercise OR sports OR walking OR jogging OR running OR bicycling OR dancing OR soccer OR rugby OR baseball OR basketball OR swiming OR hopscotch OR football OR training OR fitness OR lifting OR physical OR games) AND Topic=(human OR humans)

Databases=SCI-EXPANDED, SSCI, A&HCI, CPCI-S, CPCI-SSH Timespan=1990-01-01 - 2012-09-10

Lemmatization=On

**7. Sport Discus**

Sport Discus (105 citations) Line S52

EBSCOhost: Print Search HistoryLoading...

Revised Date: 07/2011 Accessibility Information and TipsPrint Search History

Thursday, September 06, 2012 10:40:04 AM

# Query Limiters/Expanders Last Run Via Results Action

S156 S154 and S155 Search modes - Find all my search terms Interface -

EBSCOhost

Search Screen - Advanced Search

Database - SPORTDiscus with Full Text Display Edit S156

S155 Limiters - Published Date: 19900101-20121231

Search modes - Find all my search terms Interface - EBSCOhost

Search Screen - Advanced Search

Database - SPORTDiscus with Full Text Display Edit S155

S154 S152 NOT S153 Search modes - Find all my search terms Interface -

EBSCOhost

Search Screen - Advanced Search

Database - SPORTDiscus with Full Text Display Edit S154

S153 TX college OR colleges OR university OR universities Search modes -

Find all my search terms Interface - EBSCOhost

Search Screen - Advanced Search

Database - SPORTDiscus with Full Text Display Edit S153

S152 S147 NOT S151 Search modes - Find all my search terms Interface -

EBSCOhost

Search Screen - Advanced Search

Database - SPORTDiscus with Full Text Display Edit S152

S151 S148 OR S149 OR S150 Search modes - Find all my search terms

Interface - EBSCOhost

Search Screen - Advanced Search

Database - SPORTDiscus with Full Text Display Edit S151

S150 SU historical article Search modes - SmartText Searching Interface -

EBSCOhost

Search Screen - Advanced Search

Database - SPORTDiscus with Full Text Display Edit S150

S149 TX letter OR PT letter Search modes - SmartText Searching Interface -

EBSCOhost

Search Screen - Advanced Search

Database - SPORTDiscus with Full Text Display Edit S149

S148 TX case w/2 report Search modes - Find all my search terms Interface

- EBSCOhost

Search Screen - Advanced Search

Database - SPORTDiscus with Full Text Display Edit S148

S147 S145 AND (S110 AND S124) Search modes - Find all my search terms

Interface - EBSCOhost

Search Screen - Advanced Search

Database - SPORTDiscus with Full Text Display Edit S147

S146 S139 OR S145 Search modes - Find all my search terms Interface -

EBSCOhost

Search Screen - Advanced Search

Database - SPORTDiscus with Full Text Display Edit S146

S145 S140 OR S141 OR S142 OR S143 OR S144 Search modes - Find all my

search terms Interface - EBSCOhost

Search Screen - Advanced Search

Database - SPORTDiscus with Full Text Display Edit S145

S144 TX allocated n/2 random* Search modes - Find all my search terms

Interface - EBSCOhost

Search Screen - Advanced Search

Database - SPORTDiscus with Full Text Display Edit S144

S143 TX Randomly n/3 allocat* Search modes - Find all my search terms

Interface - EBSCOhost

Search Screen - Advanced Search

Database - SPORTDiscus with Full Text Display Edit S143

S142 (singl* w/1 mask*) OR (doubl* w/1 mask*) OR (treb* w/1 mask*) OR

(tripl* w/1 mask*) Search modes - Find all my search terms Interface -

EBSCOhost

Search Screen - Advanced Search

Database - SPORTDiscus with Full Text Display Edit S142

S141 (singl* w/1 blind*) OR (doubl* w/1 blind*) OR (treb* w/1 blind*) OR

(tripl* w/1 blind*) Search modes - Find all my search terms Interface -

EBSCOhost

Search Screen - Advanced Search

Database - SPORTDiscus with Full Text Display Edit S141

S140 TX placebo* OR (clinical w/1 trial*) Search modes - Find all my

search terms Interface - EBSCOhost

Search Screen - Advanced Search

Database - SPORTDiscus with Full Text Display Edit S140

S139 S125 OR S126 OR S127 OR S128 OR S129 OR S130 OR S132 OR S133 OR S134

OR S135 OR S136 OR S137 OR S138 Search modes - Find all my search terms

Interface - EBSCOhost

Search Screen - Advanced Search

Database - SPORTDiscus with Full Text Display Edit S139

S138 DE “clinical Trials as topic” Search modes - SmartText Searching

Interface - EBSCOhost

Search Screen - Advanced Search

Database - SPORTDiscus with Full Text Display Edit S138

S137 DE clinical trials Search modes - Find all my search terms Interface

- EBSCOhost

Search Screen - Advanced Search

Database - SPORTDiscus with Full Text Display Edit S137

S136 DE multicenter study Search modes - Find all my search terms

Interface - EBSCOhost

Search Screen - Advanced Search

Database - SPORTDiscus with Full Text Display Edit S136

S135 DE controlled clinical trial Search modes - SmartText Searching

Interface - EBSCOhost

Search Screen - Advanced Search

Database - SPORTDiscus with Full Text Display Edit S135

S134 DE "clinical trial, phase iv" Search modes - SmartText Searching

Interface - EBSCOhost

Search Screen - Advanced Search

Database - SPORTDiscus with Full Text Display Edit S134

S133 DE "clinical trial, phase iii" Search modes - SmartText Searching

Interface - EBSCOhost

Search Screen - Advanced Search

Database - SPORTDiscus with Full Text Display Edit S133

S132 DE "clinical trial, phase ii" Search modes - SmartText Searching

Interface - EBSCOhost

Search Screen - Advanced Search

Database - SPORTDiscus with Full Text Display Edit S132

S131 DE "clinical trial, phase i" Search modes - SmartText Searching

Interface - EBSCOhost

Search Screen - Advanced Search

Database - SPORTDiscus with Full Text Display Edit S131

S130 DE clinical trial Search modes - Find all my search terms Interface -

EBSCOhost

Search Screen - Advanced Search

Database - SPORTDiscus with Full Text Display Edit S130

S129 DE single blind method Search modes - Find all my search terms

Interface - EBSCOhost

Search Screen - Advanced Search

Database - SPORTDiscus with Full Text Display Edit S129

S128 DE Double Blind method Search modes - SmartText Searching Interface -

EBSCOhost

Search Screen - Advanced Search

Database - SPORTDiscus with Full Text Display Edit S128

S127 DE “Random Allocation” Search modes - SmartText Searching Interface -

EBSCOhost

Search Screen - Advanced Search

Database - SPORTDiscus with Full Text Display Edit S127

S126 DE randomized controlled trial Search modes - Find all my search

terms Interface - EBSCOhost

Search Screen - Advanced Search

Database - SPORTDiscus with Full Text Display Edit S126

S125 DE Randomized Controlled Trials as Topic Search modes - Find all my

search terms Interface - EBSCOhost

Search Screen - Advanced Search

Database - SPORTDiscus with Full Text Display Edit S125

S124 S122 AND S123 Search modes - Find all my search terms Interface -

EBSCOhost

Search Screen - Advanced Search

Database - SPORTDiscus with Full Text Display Edit S124

S123 TX (Human OR HUMANS) OR SU (HUMAN OR HUMANS) Search modes - Find all

my search terms Interface - EBSCOhost

Search Screen - Advanced Search

Database - SPORTDiscus with Full Text Display Edit S123

S122 S113 OR S114 OR S115 OR S116 OR S117 OR S118 OR S119 OR S120 OR S121

Search modes - Find all my search terms Interface - EBSCOhost

Search Screen - Advanced Search

Database - SPORTDiscus with Full Text Display Edit S122

S121 TX cross n/3 train* Search modes - Find all my search terms Interface

- EBSCOhost

Search Screen - Advanced Search

Database - SPORTDiscus with Full Text Display Edit S121

S120 TX “Dance Dance Revolution" Search modes - Find all my search terms

Interface - EBSCOhost

Search Screen - Advanced Search

Database - SPORTDiscus with Full Text Display Edit S120

S119 TX “video games” OR “computer games” Search modes - Find all my

search terms Interface - EBSCOhost

Search Screen - Advanced Search

Database - SPORTDiscus with Full Text Display Edit S119

S118 TX (jump* n/3 rope*) Search modes - Find all my search terms

Interface - EBSCOhost

Search Screen - Advanced Search

Database - SPORTDiscus with Full Text Display Edit S118

S117 TX (strength n/6 train*) OR (resistance n/6 train*) OR (circuit n/6

train*) OR (enduran* n/6 train*) OR (aerob* n/6 train*) OR (physical n/6

train*) OR (fit n/6 train*) OR (fitness n/6 train*) Search modes - Find

all my search terms Interface - EBSCOhost

Search Screen - Advanced Search

Database - SPORTDiscus with Full Text Display Edit S117

S116 TX (weight n/3 lift*) Search modes - Find all my search terms

Interface - EBSCOhost

Search Screen - Advanced Search

Database - SPORTDiscus with Full Text Display Edit S116

S115 DE physical exertion OR physical therapy modalities Search modes -

Find all my search terms Interface - EBSCOhost

Search Screen - Advanced Search

Database - SPORTDiscus with Full Text Display Edit S115

S114 TX (activit* n/3 physical) OR (educat* n/3 physical) OR (fitness n/3

physical) OR (therapy n/3 physical) OR (therapies n/3 physical) OR

(therapeutic n/3 physical) Search modes - Find all my search terms

Interface - EBSCOhost

Search Screen - Advanced Search

Database - SPORTDiscus with Full Text Display Edit S114

S113 TX bic* OR walk* OR jogging OR jogger OR runner OR runs OR bicycle*

OR dancing OR dancer OR dances OR soccer OR rugby OR baseball* OR

basketball* OR swim* OR hopscotch OR football Search modes - Find all my

search terms Interface - EBSCOhost

Search Screen - Advanced Search

Database - SPORTDiscus with Full Text Display Edit S113

S112 S110 AND S111 Search modes - Find all my search terms Interface -

EBSCOhost

Search Screen - Advanced Search

Database - SPORTDiscus with Full Text Display Edit S112

S111 TX child OR children OR kid OR kids OR youth OR youths OR pediatric*

OR paediatrics OR teen OR teens OR teenager* OR tween OR tweens Search

modes - Find all my search terms Interface - EBSCOhost

Search Screen - Advanced Search

Database - SPORTDiscus with Full Text Display Edit S111

S110 S105 OR S106 OR S107 OR S108 OR S109 Search modes - Find all my

search terms Interface - EBSCOhost

Search Screen - Advanced Search

Database - SPORTDiscus with Full Text Display Edit S110

S109 TX (fat n/3 body) OR (mass n/3 body) OR (weight n/3 body) OR

(composition n/3 body) Search modes - Find all my search terms Interface -

EBSCOhost

Search Screen - Advanced Search

Database - SPORTDiscus with Full Text Display Edit S109

S108 TX quetelet* n/3 index Search modes - Find all my search terms

Interface - EBSCOhost

Search Screen - Advanced Search

Database - SPORTDiscus with Full Text Display Edit S108

S107 TX “body mass index” OR bmi Search modes - Find all my search terms

Interface - EBSCOhost

Search Screen - Advanced Search

Database - SPORTDiscus with Full Text Display Edit S107

S106 TX “weight loss” OR “body mass” Search modes - Find all my search

terms Interface - EBSCOhost

Search Screen - Advanced Search

Database - SPORTDiscus with Full Text Display Edit S106

S105 TX obesity OR obese OR sedentary OR overweight OR FAT OR Adipose OR

adiposity OR (over n/3 weight) Search modes - Find all my search terms

Interface - EBSCOhost

Search Screen - Advanced Search

Database - SPORTDiscus with Full Text Display Edit S105

S104 S102 and S103 Search modes - Find all my search terms Interface -

EBSCOhost

Search Screen - Advanced Search

Database - SPORTDiscus with Full Text Display Edit S104

S103 Limiters - Published Date: 19900101-20121231

Search modes - Find all my search terms Interface - EBSCOhost

Search Screen - Advanced Search

Database - SPORTDiscus with Full Text Display Edit S103

S102 S100 NOT S101 Search modes - Find all my search terms Interface -

EBSCOhost

Search Screen - Advanced Search

Database - SPORTDiscus with Full Text Display Edit S102

S101 TX college OR colleges OR university OR universities Search modes -

Find all my search terms Interface - EBSCOhost

Search Screen - Advanced Search

Database - SPORTDiscus with Full Text Display Edit S101

S100 S95 NOT S99 Search modes - Find all my search terms Interface -

EBSCOhost

Search Screen - Advanced Search

Database - SPORTDiscus with Full Text Display Edit S100

S99 S96 OR S97 OR S98 Search modes - Find all my search terms Interface -

EBSCOhost

Search Screen - Advanced Search

Database - SPORTDiscus with Full Text Display Edit S99

S98 SU historical article Search modes - SmartText Searching Interface -

EBSCOhost

Search Screen - Advanced Search

Database - SPORTDiscus with Full Text Display Edit S98

S97 TX letter OR PT letter Search modes - SmartText Searching Interface -

EBSCOhost

Search Screen - Advanced Search

Database - SPORTDiscus with Full Text Display Edit S97

S96 TX case w/2 report Search modes - Find all my search terms Interface -

EBSCOhost

Search Screen - Advanced Search

Database - SPORTDiscus with Full Text Display Edit S96

S95 S93 AND (S58 AND S72) Search modes - Find all my search terms

Interface - EBSCOhost

Search Screen - Advanced Search

Database - SPORTDiscus with Full Text Display Edit S95

S94 S87 OR S93 Search modes - Find all my search terms Interface -

EBSCOhost

Search Screen - Advanced Search

Database - SPORTDiscus with Full Text Display Edit S94

S93 S88 OR S89 OR S90 OR S91 OR S92 Search modes - Find all my search

terms Interface - EBSCOhost

Search Screen - Advanced Search

Database - SPORTDiscus with Full Text Display Edit S93

S92 TX allocated n/2 random* Search modes - Find all my search terms

Interface - EBSCOhost

Search Screen - Advanced Search

Database - SPORTDiscus with Full Text Display Edit S92

S91 TX Randomly n/3 allocat* Search modes - Find all my search terms

Interface - EBSCOhost

Search Screen - Advanced Search

Database - SPORTDiscus with Full Text Display Edit S91

S90 (singl* w/1 mask*) OR (doubl* w/1 mask*) OR (treb* w/1 mask*) OR

(tripl* w/1 mask*) Search modes - Find all my search terms Interface -

EBSCOhost

Search Screen - Advanced Search

Database - SPORTDiscus with Full Text Display Edit S90

S89 (singl* w/1 blind*) OR (doubl* w/1 blind*) OR (treb* w/1 blind*) OR

(tripl* w/1 blind*) Search modes - Find all my search terms Interface -

EBSCOhost

Search Screen - Advanced Search

Database - SPORTDiscus with Full Text Display Edit S89

S88 TX placebo* OR (clinical w/1 trial*) Search modes - Find all my search

terms Interface - EBSCOhost

Search Screen - Advanced Search

Database - SPORTDiscus with Full Text Display Edit S88

S87 S73 OR S74 OR S75 OR S76 OR S77 OR S78 OR S80 OR S81 OR S82 OR S83 OR

S84 OR S85 OR S86 Search modes - Find all my search terms Interface -

EBSCOhost

Search Screen - Advanced Search

Database - SPORTDiscus with Full Text Display Edit S87

S86 DE “clinical Trials as topic” Search modes - SmartText Searching

Interface - EBSCOhost

Search Screen - Advanced Search

Database - SPORTDiscus with Full Text Display Edit S86

S85 DE clinical trials Search modes - Find all my search terms Interface -

EBSCOhost

Search Screen - Advanced Search

Database - SPORTDiscus with Full Text Display Edit S85

S84 DE multicenter study Search modes - Find all my search terms Interface

- EBSCOhost

Search Screen - Advanced Search

Database - SPORTDiscus with Full Text Display Edit S84

S83 DE controlled clinical trial Search modes - SmartText Searching

Interface - EBSCOhost

Search Screen - Advanced Search

Database - SPORTDiscus with Full Text Display Edit S83

S82 DE "clinical trial, phase iv" Search modes - SmartText Searching

Interface - EBSCOhost

Search Screen - Advanced Search

Database - SPORTDiscus with Full Text Display Edit S82

S81 DE "clinical trial, phase iii" Search modes - SmartText Searching

Interface - EBSCOhost

Search Screen - Advanced Search

Database - SPORTDiscus with Full Text Display Edit S81

S80 DE "clinical trial, phase ii" Search modes - SmartText Searching

Interface - EBSCOhost

Search Screen - Advanced Search

Database - SPORTDiscus with Full Text Display Edit S80

S79 DE "clinical trial, phase i" Search modes - SmartText Searching

Interface - EBSCOhost

Search Screen - Advanced Search

Database - SPORTDiscus with Full Text Display Edit S79

S78 DE clinical trial Search modes - Find all my search terms Interface -

EBSCOhost

Search Screen - Advanced Search

Database - SPORTDiscus with Full Text Display Edit S78

S77 DE single blind method Search modes - Find all my search terms

Interface - EBSCOhost

Search Screen - Advanced Search

Database - SPORTDiscus with Full Text Display Edit S77

S76 DE Double Blind method Search modes - SmartText Searching Interface -

EBSCOhost

Search Screen - Advanced Search

Database - SPORTDiscus with Full Text Display Edit S76

S75 DE “Random Allocation” Search modes - SmartText Searching Interface -

EBSCOhost

Search Screen - Advanced Search

Database - SPORTDiscus with Full Text Display Edit S75

S74 DE randomized controlled trial Search modes - Find all my search terms

Interface - EBSCOhost

Search Screen - Advanced Search

Database - SPORTDiscus with Full Text Display Edit S74

S73 DE Randomized Controlled Trials as Topic Search modes - Find all my

search terms Interface - EBSCOhost

Search Screen - Advanced Search

Database - SPORTDiscus with Full Text Display Edit S73

S72 S70 AND S71 Search modes - Find all my search terms Interface -

EBSCOhost

Search Screen - Advanced Search

Database - SPORTDiscus with Full Text Display Edit S72

S71 TX (Human OR HUMANS) OR SU (HUMAN OR HUMANS) Search modes - Find all

my search terms Interface - EBSCOhost

Search Screen - Advanced Search

Database - SPORTDiscus with Full Text Display Edit S71

S70 S61 OR S62 OR S63 OR S64 OR S65 OR S66 OR S67 OR S68 OR S69 Search

modes - Find all my search terms Interface - EBSCOhost

Search Screen - Advanced Search

Database - SPORTDiscus with Full Text Display Edit S70

S69 TX cross n/3 train* Search modes - Find all my search terms Interface

- EBSCOhost

Search Screen - Advanced Search

Database - SPORTDiscus with Full Text Display Edit S69

S68 TX “Dance Dance Revolution" Search modes - Find all my search terms

Interface - EBSCOhost

Search Screen - Advanced Search

Database - SPORTDiscus with Full Text Display Edit S68

S67 TX “video games” OR “computer games” Search modes - Find all my search

terms Interface - EBSCOhost

Search Screen - Advanced Search

Database - SPORTDiscus with Full Text Display Edit S67

S66 TX (jump* n/3 rope*) Search modes - Find all my search terms Interface

- EBSCOhost

Search Screen - Advanced Search

Database - SPORTDiscus with Full Text Display Edit S66

S65 TX (strength n/6 train*) OR (resistance n/6 train*) OR (circuit n/6

train*) OR (enduran* n/6 train*) OR (aerob* n/6 train*) OR (physical n/6

train*) OR (fit n/6 train*) OR (fitness n/6 train*) Search modes - Find

all my search terms Interface - EBSCOhost

Search Screen - Advanced Search

Database - SPORTDiscus with Full Text Display Edit S65

S64 TX (weight n/3 lift*) Search modes - Find all my search terms

Interface - EBSCOhost

Search Screen - Advanced Search

Database - SPORTDiscus with Full Text Display Edit S64

S63 DE physical exertion OR physical therapy modalities Search modes -

Find all my search terms Interface - EBSCOhost

Search Screen - Advanced Search

Database - SPORTDiscus with Full Text Display Edit S63

S62 TX (activit* n/3 physical) OR (educat* n/3 physical) OR (fitness n/3

physical) OR (therapy n/3 physical) OR (therapies n/3 physical) OR

(therapeutic n/3 physical) Search modes - Find all my search terms

Interface - EBSCOhost

Search Screen - Advanced Search

Database - SPORTDiscus with Full Text Display Edit S62

S61 TX bic* OR walk* OR jogging OR jogger OR runner OR runs OR bicycle* OR

dancing OR dancer OR dances OR soccer OR rugby OR baseball* OR basketball*

OR swim* OR hopscotch OR football Search modes - Find all my search terms

Interface - EBSCOhost

Search Screen - Advanced Search

Database - SPORTDiscus with Full Text Display Edit S61

S60 S58 AND S59 Search modes - Find all my search terms Interface -

EBSCOhost

Search Screen - Advanced Search

Database - SPORTDiscus with Full Text Display Edit S60

S59 TX child OR children OR kid OR kids OR youth OR youths OR pediatric*

OR paediatrics OR teen OR teens OR teenager* OR tween OR tweens Search

modes - Find all my search terms Interface - EBSCOhost

Search Screen - Advanced Search

Database - SPORTDiscus with Full Text Display Edit S59

S58 S53 OR S54 OR S55 OR S56 OR S57 Search modes - Find all my search

terms Interface - EBSCOhost

Search Screen - Advanced Search

Database - SPORTDiscus with Full Text Display Edit S58

S57 TX (fat n/3 body) OR (mass n/3 body) OR (weight n/3 body) OR

(composition n/3 body) Search modes - Find all my search terms Interface -

EBSCOhost

Search Screen - Advanced Search

Database - SPORTDiscus with Full Text Display Edit S57

S56 TX quetelet* n/3 index Search modes - Find all my search terms

Interface - EBSCOhost

Search Screen - Advanced Search

Database - SPORTDiscus with Full Text Display Edit S56

S55 TX “body mass index” OR bmi Search modes - Find all my search terms

Interface - EBSCOhost

Search Screen - Advanced Search

Database - SPORTDiscus with Full Text Display Edit S55

S54 TX “weight loss” OR “body mass” Search modes - Find all my search

terms Interface - EBSCOhost

Search Screen - Advanced Search

Database - SPORTDiscus with Full Text Display Edit S54

S53 TX obesity OR obese OR sedentary OR overweight OR FAT OR Adipose OR

adiposity OR (over n/3 weight) Search modes - Find all my search terms

Interface - EBSCOhost

Search Screen - Advanced Search

Database - SPORTDiscus with Full Text Display Edit S53

S52 s8 AND s51 Limiters - Published Date: 19900101-20121231

Search modes - Find all my search terms Interface - EBSCOhost

Search Screen - Advanced Search

Database - SPORTDiscus with Full Text 105 Edit S52

S51 S48 NOT S49 Limiters - Published Date from: 19900101-20121231

Search modes - Find all my search terms Interface - EBSCOhost

Search Screen - Basic Search

Database - Academic Search Complete Display Edit S51

S50 S48 NOT S49 Search modes - Find all my search terms Interface -

EBSCOhost

Search Screen - Basic Search

Database - Academic Search Complete Display Edit S50

S49 TX college OR colleges OR university OR universities Search modes -

Find all my search terms Interface - EBSCOhost

Search Screen - Basic Search

Database - Academic Search Complete Display Edit S49

S48 S43 NOT S47 Search modes - Find all my search terms Interface -

EBSCOhost

Search Screen - Basic Search

Database - Academic Search Complete Display Edit S48

S47 S44 OR S45 OR S46 Search modes - Find all my search terms Interface -

EBSCOhost

Search Screen - Basic Search

Database - Academic Search Complete Display Edit S47

S46 SU historical article Search modes - SmartText Searching Interface -

EBSCOhost

Search Screen - Basic Search

Database - Academic Search Complete Display Edit S46

S45 TX letter OR PT letter Search modes - SmartText Searching Interface -

EBSCOhost

Search Screen - Basic Search

Database - Academic Search Complete Display Edit S45

S44 TX case w/2 report Search modes - Find all my search terms Interface -

EBSCOhost

Search Screen - Basic Search

Database - Academic Search Complete Display Edit S44

S43 S41 AND (S6 AND S20) Search modes - Find all my search terms Interface

- EBSCOhost

Search Screen - Basic Search

Database - Academic Search Complete Display Edit S43

S42 S35 OR S41 Search modes - Find all my search terms Interface -

EBSCOhost

Search Screen - Basic Search

Database - Academic Search Complete Display Edit S42

S41 S36 OR S37 OR S38 OR S39 OR S40 Search modes - Find all my search

terms Interface - EBSCOhost

Search Screen - Basic Search

Database - Academic Search Complete Display Edit S41

S40 TX allocated n/2 random* Search modes - Find all my search terms

Interface - EBSCOhost

Search Screen - Basic Search

Database - Academic Search Complete Display Edit S40

S39 TX Randomly n/3 allocat* Search modes - Find all my search terms

Interface - EBSCOhost

Search Screen - Basic Search

Database - Academic Search Complete Display Edit S39

S38 (singl* w/1 mask*) OR (doubl* w/1 mask*) OR (treb* w/1 mask*) OR

(tripl* w/1 mask*) Search modes - Find all my search terms Interface -

EBSCOhost

Search Screen - Basic Search

Database - Academic Search Complete Display Edit S38

S37 (singl* w/1 blind*) OR (doubl* w/1 blind*) OR (treb* w/1 blind*) OR

(tripl* w/1 blind*) Search modes - Find all my search terms Interface -

EBSCOhost

Search Screen - Basic Search

Database - Academic Search Complete Display Edit S37

S36 TX placebo* OR (clinical w/1 trial*) Search modes - Find all my search

terms Interface - EBSCOhost

Search Screen - Basic Search

Database - Academic Search Complete Display Edit S36

S35 S21 OR S22 OR S23 OR S24 OR S25 OR S26 OR S28 OR S29 OR S30 OR S31 OR

S32 OR S33 OR S34 Search modes - Find all my search terms Interface -

EBSCOhost

Search Screen - Basic Search

Database - Academic Search Complete Display Edit S35

S34 DE “clinical Trials as topic” Search modes - SmartText Searching

Interface - EBSCOhost

Search Screen - Basic Search

Database - Academic Search Complete Display Edit S34

S33 DE clinical trials Search modes - Find all my search terms Interface -

EBSCOhost

Search Screen - Basic Search

Database - Academic Search Complete Display Edit S33

S32 DE multicenter study Search modes - Find all my search terms Interface

- EBSCOhost

Search Screen - Basic Search

Database - Academic Search Complete Display Edit S32

S31 DE controlled clinical trial Search modes - SmartText Searching

Interface - EBSCOhost

Search Screen - Basic Search

Database - Academic Search Complete Display Edit S31

S30 DE "clinical trial, phase iv" Search modes - SmartText Searching

Interface - EBSCOhost

Search Screen - Basic Search

Database - Academic Search Complete Display Edit S30

S29 DE "clinical trial, phase iii" Search modes - SmartText Searching

Interface - EBSCOhost

Search Screen - Basic Search

Database - Academic Search Complete Display Edit S29

S28 DE "clinical trial, phase ii" Search modes - SmartText Searching

Interface - EBSCOhost

Search Screen - Basic Search

Database - Academic Search Complete Display Edit S28

S27 DE "clinical trial, phase i" Search modes - SmartText Searching

Interface - EBSCOhost

Search Screen - Basic Search

Database - Academic Search Complete Display Edit S27

S26 DE clinical trial Search modes - Find all my search terms Interface -

EBSCOhost

Search Screen - Basic Search

Database - Academic Search Complete Display Edit S26

S25 DE single blind method Search modes - Find all my search terms

Interface - EBSCOhost

Search Screen - Basic Search

Database - Academic Search Complete Display Edit S25

S24 DE Double Blind method Search modes - SmartText Searching Interface -

EBSCOhost

Search Screen - Basic Search

Database - Academic Search Complete Display Edit S24

S23 DE “Random Allocation” Search modes - SmartText Searching Interface -

EBSCOhost

Search Screen - Basic Search

Database - Academic Search Complete Display Edit S23

S22 DE randomized controlled trial Search modes - Find all my search terms

Interface - EBSCOhost

Search Screen - Basic Search

Database - Academic Search Complete Display Edit S22

S21 DE Randomized Controlled Trials as Topic Search modes - Find all my

search terms Interface - EBSCOhost

Search Screen - Basic Search

Database - Academic Search Complete Display Edit S21

S20 S18 AND S19 Search modes - Find all my search terms Interface -

EBSCOhost

Search Screen - Advanced Search

Database - ERIC 1848 Edit S20

S19 TX (Human OR HUMANS) OR SU (HUMAN OR HUMANS) Search modes - Find all

my search terms Interface - EBSCOhost

Search Screen - Basic Search

Database - Academic Search Complete Display Edit S19

S18 S9 OR S10 OR S11 OR S12 OR S13 OR S14 OR S15 OR S16 OR S17 Search

modes - Find all my search terms Interface - EBSCOhost

Search Screen - Basic Search

Database - Academic Search Complete Display Edit S18

S17 TX cross n/3 train* Search modes - Find all my search terms Interface

- EBSCOhost

Search Screen - Basic Search

Database - Academic Search Complete Display Edit S17

S16 TX “Dance Dance Revolution" Search modes - Find all my search terms

Interface - EBSCOhost

Search Screen - Basic Search

Database - Academic Search Complete Display Edit S16

S15 TX “video games” OR “computer games” Search modes - Find all my search

terms Interface - EBSCOhost

Search Screen - Basic Search

Database - Academic Search Complete Display Edit S15

S14 TX (jump* n/3 rope*) Search modes - Find all my search terms Interface

- EBSCOhost

Search Screen - Basic Search

Database - Academic Search Complete Display Edit S14

S13 TX (strength n/6 train*) OR (resistance n/6 train*) OR (circuit n/6

train*) OR (enduran* n/6 train*) OR (aerob* n/6 train*) OR (physical n/6

train*) OR (fit n/6 train*) OR (fitness n/6 train*) Search modes - Find

all my search terms Interface - EBSCOhost

Search Screen - Basic Search

Database - Academic Search Complete Display Edit S13

S12 TX (weight n/3 lift*) Search modes - Find all my search terms

Interface - EBSCOhost

Search Screen - Basic Search

Database - Academic Search Complete Display Edit S12

S11 DE physical exertion OR physical therapy modalities Search modes -

Find all my search terms Interface - EBSCOhost

Search Screen - Basic Search

Database - Academic Search Complete Display Edit S11

S10 TX (activit* n/3 physical) OR (educat* n/3 physical) OR (fitness n/3

physical) OR (therapy n/3 physical) OR (therapies n/3 physical) OR

(therapeutic n/3 physical) Search modes - Find all my search terms

Interface - EBSCOhost

Search Screen - Basic Search

Database - Academic Search Complete Display Edit S10

S9 TX bic* OR walk* OR jogging OR jogger OR runner OR runs OR bicycle* OR

dancing OR dancer OR dances OR soccer OR rugby OR baseball* OR basketball*

OR swim* OR hopscotch OR football Search modes - Find all my search terms

Interface - EBSCOhost

Search Screen - Basic Search

Database - Academic Search Complete Display Edit S9

S8 S6 AND S7 Search modes - Find all my search terms Interface - EBSCOhost

Search Screen - Basic Search

Database - Academic Search Complete Display Edit S8

S7 TX child OR children OR kid OR kids OR youth OR youths OR pediatric* OR

paediatrics OR teen OR teens OR teenager* OR tween OR tweens Search modes

- Find all my search terms Interface - EBSCOhost

Search Screen - Basic Search

Database - Academic Search Complete Display Edit S7

S6 S1 OR S2 OR S3 OR S4 OR S5 Search modes - Find all my search terms

Interface - EBSCOhost

Search Screen - Basic Search

Database - Academic Search Complete Display Edit S6

S5 TX (fat n/3 body) OR (mass n/3 body) OR (weight n/3 body) OR

(composition n/3 body) Search modes - Find all my search terms Interface -

EBSCOhost

Search Screen - Basic Search

Database - Academic Search Complete Display Edit S5

S4 TX quetelet* n/3 index Search modes - Find all my search terms

Interface - EBSCOhost

Search Screen - Basic Search

Database - Academic Search Complete Display Edit S4

S3 TX “body mass index” OR bmi Search modes - Find all my search terms

Interface - EBSCOhost

Search Screen - Basic Search

Database - Academic Search Complete Display Edit S3

S2 TX “weight loss” OR “body mass” Search modes - Find all my search terms

Interface - EBSCOhost

Search Screen - Basic Search

Database - Academic Search Complete Display Edit S2

S1 TX obesity OR obese OR sedentary OR overweight OR FAT OR Adipose OR

adiposity OR (over n/3 weight) Search modes - Find all my search terms

Interface - EBSCOhost

Search Screen - Basic Search

Database - Academic Search Complete Display Edit S1

**8. ERIC**

ERIC Search Strategy (22 hits)

EBSCOhost: Print Search HistoryLoading...

Revised Date: 07/2011 Accessibility Information and TipsPrint Search History

Monday, September 7, 2012 8:29 AM

# Query Limiters/Expanders Last Run Via Results Action

S52 s8 AND s51 Limiters - Published Date from: 19900101-20121231

Search modes - Find all my search terms Interface - EBSCOhost

Search Screen - Basic Search

Database - Academic Search Complete Display Edit S52

S51 S48 NOT S49 Limiters - Published Date from: 19900101-20121231

Search modes - Find all my search terms Interface - EBSCOhost

Search Screen - Basic Search

Database - Academic Search Complete Display Edit S51

S50 S48 NOT S49 Search modes - Find all my search terms Interface -

EBSCOhost

Search Screen - Basic Search

Database - Academic Search Complete Display Edit S50

S49 TX college OR colleges OR university OR universities Search modes -

Find all my search terms Interface - EBSCOhost

Search Screen - Basic Search

Database - Academic Search Complete Display Edit S49

S48 S43 NOT S47 Search modes - Find all my search terms Interface -

EBSCOhost

Search Screen - Basic Search

Database - Academic Search Complete Display Edit S48

S47 S44 OR S45 OR S46 Search modes - Find all my search terms Interface -

EBSCOhost

Search Screen - Basic Search

Database - Academic Search Complete Display Edit S47

S46 SU historical article Search modes - SmartText Searching Interface -

EBSCOhost

Search Screen - Basic Search

Database - Academic Search Complete Display Edit S46

S45 TX letter OR PT letter Search modes - SmartText Searching Interface -

EBSCOhost

Search Screen - Basic Search

Database - Academic Search Complete Display Edit S45

S44 TX case w/2 report Search modes - Find all my search terms Interface -

EBSCOhost

Search Screen - Basic Search

Database - Academic Search Complete Display Edit S44

S43 S41 AND (S6 AND S20) Search modes - Find all my search terms Interface

- EBSCOhost

Search Screen - Basic Search

Database - Academic Search Complete Display Edit S43

S42 S35 OR S41 Search modes - Find all my search terms Interface -

EBSCOhost

Search Screen - Basic Search

Database - Academic Search Complete Display Edit S42

S41 S36 OR S37 OR S38 OR S39 OR S40 Search modes - Find all my search

terms Interface - EBSCOhost

Search Screen - Basic Search

Database - Academic Search Complete Display Edit S41

S40 TX allocated n/2 random* Search modes - Find all my search terms

Interface - EBSCOhost

Search Screen - Basic Search

Database - Academic Search Complete Display Edit S40

S39 TX Randomly n/3 allocat* Search modes - Find all my search terms

Interface - EBSCOhost

Search Screen - Basic Search

Database - Academic Search Complete Display Edit S39

S38 (singl* w/1 mask*) OR (doubl* w/1 mask*) OR (treb* w/1 mask*) OR

(tripl* w/1 mask*) Search modes - Find all my search terms Interface -

EBSCOhost

Search Screen - Basic Search

Database - Academic Search Complete Display Edit S38

S37 (singl* w/1 blind*) OR (doubl* w/1 blind*) OR (treb* w/1 blind*) OR

(tripl* w/1 blind*) Search modes - Find all my search terms Interface -

EBSCOhost

Search Screen - Basic Search

Database - Academic Search Complete Display Edit S37

S36 TX placebo* OR (clinical w/1 trial*) Search modes - Find all my search

terms Interface - EBSCOhost

Search Screen - Basic Search

Database - Academic Search Complete Display Edit S36

S35 S21 OR S22 OR S23 OR S24 OR S25 OR S26 OR S28 OR S29 OR S30 OR S31 OR

S32 OR S33 OR S34 Search modes - Find all my search terms Interface -

EBSCOhost

Search Screen - Basic Search

Database - Academic Search Complete Display Edit S35

S34 DE “clinical Trials as topic” Search modes - SmartText Searching

Interface - EBSCOhost

Search Screen - Basic Search

Database - Academic Search Complete Display Edit S34

S33 DE clinical trials Search modes - Find all my search terms Interface -

EBSCOhost

Search Screen - Basic Search

Database - Academic Search Complete Display Edit S33

S32 DE multicenter study Search modes - Find all my search terms Interface

- EBSCOhost

Search Screen - Basic Search

Database - Academic Search Complete Display Edit S32

S31 DE controlled clinical trial Search modes - SmartText Searching

Interface - EBSCOhost

Search Screen - Basic Search

Database - Academic Search Complete Display Edit S31

S30 DE "clinical trial, phase iv" Search modes - SmartText Searching

Interface - EBSCOhost

Search Screen - Basic Search

Database - Academic Search Complete Display Edit S30

S29 DE "clinical trial, phase iii" Search modes - SmartText Searching

Interface - EBSCOhost

Search Screen - Basic Search

Database - Academic Search Complete Display Edit S29

S28 DE "clinical trial, phase ii" Search modes - SmartText Searching

Interface - EBSCOhost

Search Screen - Basic Search

Database - Academic Search Complete Display Edit S28

S27 DE "clinical trial, phase i" Search modes - SmartText Searching

Interface - EBSCOhost

Search Screen - Basic Search

Database - Academic Search Complete Display Edit S27

S26 DE clinical trial Search modes - Find all my search terms Interface -

EBSCOhost

Search Screen - Basic Search

Database - Academic Search Complete Display Edit S26

S25 DE single blind method Search modes - Find all my search terms

Interface - EBSCOhost

Search Screen - Basic Search

Database - Academic Search Complete Display Edit S25

S24 DE Double Blind method Search modes - SmartText Searching Interface -

EBSCOhost

Search Screen - Basic Search

Database - Academic Search Complete Display Edit S24

S23 DE “Random Allocation” Search modes - SmartText Searching Interface -

EBSCOhost

Search Screen - Basic Search

Database - Academic Search Complete Display Edit S23

S22 DE randomized controlled trial Search modes - Find all my search terms

Interface - EBSCOhost

Search Screen - Basic Search

Database - Academic Search Complete Display Edit S22

S21 DE Randomized Controlled Trials as Topic Search modes - Find all my

search terms Interface - EBSCOhost

Search Screen - Basic Search

Database - Academic Search Complete Display Edit S21

S20 S18 AND S19 Search modes - Find all my search terms Interface -

EBSCOhost

Search Screen - Basic Search

Database - Academic Search Complete Display Edit S20

S19 TX (Human OR HUMANS) OR SU (HUMAN OR HUMANS) Search modes - Find all

my search terms Interface - EBSCOhost

Search Screen - Basic Search

Database - Academic Search Complete Display Edit S19

S18 S9 OR S10 OR S11 OR S12 OR S13 OR S14 OR S15 OR S16 OR S17 Search

modes - Find all my search terms Interface - EBSCOhost

Search Screen - Basic Search

Database - Academic Search Complete Display Edit S18

S17 TX cross n/3 train* Search modes - Find all my search terms Interface

- EBSCOhost

Search Screen - Basic Search

Database - Academic Search Complete Display Edit S17

S16 TX “Dance Dance Revolution" Search modes - Find all my search terms

Interface - EBSCOhost

Search Screen - Basic Search

Database - Academic Search Complete Display Edit S16

S15 TX “video games” OR “computer games” Search modes - Find all my search

terms Interface - EBSCOhost

Search Screen - Basic Search

Database - Academic Search Complete Display Edit S15

S14 TX (jump* n/3 rope*) Search modes - Find all my search terms Interface

- EBSCOhost

Search Screen - Basic Search

Database - Academic Search Complete Display Edit S14

S13 TX (strength n/6 train*) OR (resistance n/6 train*) OR (circuit n/6

train*) OR (enduran* n/6 train*) OR (aerob* n/6 train*) OR (physical n/6

train*) OR (fit n/6 train*) OR (fitness n/6 train*) Search modes - Find

all my search terms Interface - EBSCOhost

Search Screen - Basic Search

Database - Academic Search Complete Display Edit S13

S12 TX (weight n/3 lift*) Search modes - Find all my search terms

Interface - EBSCOhost

Search Screen - Basic Search

Database - Academic Search Complete Display Edit S12

S11 DE physical exertion OR physical therapy modalities Search modes -

Find all my search terms Interface - EBSCOhost

Search Screen - Basic Search

Database - Academic Search Complete Display Edit S11

S10 TX (activit* n/3 physical) OR (educat* n/3 physical) OR (fitness n/3

physical) OR (therapy n/3 physical) OR (therapies n/3 physical) OR

(therapeutic n/3 physical) Search modes - Find all my search terms

Interface - EBSCOhost

Search Screen - Basic Search

Database - Academic Search Complete Display Edit S10

S9 TX bic* OR walk* OR jogging OR jogger OR runner OR runs OR bicycle* OR

dancing OR dancer OR dances OR soccer OR rugby OR baseball* OR basketball*

OR swim* OR hopscotch OR football Search modes - Find all my search terms

Interface - EBSCOhost

Search Screen - Basic Search

Database - Academic Search Complete Display Edit S9

S8 S6 AND S7 Search modes - Find all my search terms Interface - EBSCOhost

Search Screen - Basic Search

Database - Academic Search Complete Display Edit S8

S7 TX child OR children OR kid OR kids OR youth OR youths OR pediatric* OR

paediatrics OR teen OR teens OR teenager* OR tween OR tweens Search modes

- Find all my search terms Interface - EBSCOhost

Search Screen - Basic Search

Database - Academic Search Complete Display Edit S7

S6 S1 OR S2 OR S3 OR S4 OR S5 Search modes - Find all my search terms

Interface - EBSCOhost

Search Screen - Basic Search

Database - Academic Search Complete Display Edit S6

S5 TX (fat n/3 body) OR (mass n/3 body) OR (weight n/3 body) OR

(composition n/3 body) Search modes - Find all my search terms Interface -

EBSCOhost

Search Screen - Basic Search

Database - Academic Search Complete Display Edit S5

S4 TX quetelet* n/3 index Search modes - Find all my search terms

Interface - EBSCOhost

Search Screen - Basic Search

Database - Academic Search Complete Display Edit S4

S3 TX “body mass index” OR bmi Search modes - Find all my search terms

Interface - EBSCOhost

Search Screen - Basic Search

Database - Academic Search Complete Display Edit S3

S2 TX “weight loss” OR “body mass” Search modes - Find all my search terms

Interface - EBSCOhost

Search Screen - Basic Search

Database - Academic Search Complete Display Edit S2

S1 TX obesity OR obese OR sedentary OR overweight OR FAT OR Adipose OR

adiposity OR (over n/3 weight) Search modes - Find all my search terms

Interface - EBSCOhost

Search Screen - Basic Search

Database - Academic Search Complete Display Edit S1

**9. LILACS**

LILACS Database Search Strategy

RUN: September 9, 2012

Hits: 836

obesity OR obese OR sedentary OR overweight OR FAT OR Adipose OR adiposity OR BMI OR weight [Words] and child OR children OR kid OR kids OR youth OR youths OR pediatric OR paediatrics OR teen OR teens OR teenager OR tween OR tweens OR teenagers [Words] and exercise OR sports OR walking OR jogging OR running OR bicycling OR dancing OR soccer OR rugby OR baseball OR basketball OR swiming OR hopscotch OR football OR training OR fitness OR lifting OR physical OR games [Words]

**10. Cochrane**

Cochrane Library Search Strategy

Kids Obesity:

Run 9-10-12 (1080 hits)

ID Search Hits Edit Delete

#1 (obesity OR obese OR sedentary OR overweight OR FAT OR Adipose OR adiposity OR BMI OR weight):ti,ab,kw and (child OR children OR kid OR kids OR youth ORyouths OR pediatric OR paediatrics OR teen OR teens OR teenager OR tween OR tweens OR teenagers):ti,ab,kw and (exercise OR sports OR walking OR jogging OR running OR bicycling OR dancing OR soccer OR rugby OR baseball OR basketball OR swiming OR hopscotch OR football OR training OR fitness OR lifting OR physical OR games):ti,ab,kw and (human OR humans):kw, from 1990 to 2012 in Trials 1080 edit delete modified: Save as new strategy

**11. Proquest**

**Run: September 11, 2012**

**Search Strategy (4 hits)**

| **Set#** | **Searched for** | **Databases** | **Results** |  |
| --- | --- | --- | --- | --- |
| S6 | SUBJECT.exact("Obesity") AND SUBJECT.exact("Clinical trials") AND SUBJECT.exact("Children & youth") | ProQuest Dissertations & Theses: Health & Medicine | 2 | |
| S5 | su(obesity OR body mass index OR overweight OR weight) AND (child OR children OR kid OR kids OR youth OR youths OR pediatric OR paediatrics OR teen OR teens OR teenager OR tween OR teens OR teenagers) AND (ng OR running OR bicycling OR dancing OR soccer OR rugby OR baseball OR basketball OR swimming OR hopscotch OR football OR training OR fitness OR lifting OR physical OR games) AND su(clinical trials) AND pd(>19891231) | ProQuest Dissertations & Theses: Health & Medicine | 4 | |
| S4 | su(obesity OR body mass index OR overweight) AND (child OR children OR kid OR kids OR youth OR youths OR pediatric OR paediatrics OR teen OR teens OR teenager OR tween OR teens OR teenagers) AND (ng OR running OR bicycling OR dancing OR soccer OR rugby OR baseball OR basketball OR swimming OR hopscotch OR football OR training OR fitness OR lifting OR physical OR games) AND su(clinical trials) AND pd(>19891231) | ProQuest Dissertations & Theses: Health & Medicine | 2 | |
| S3 | su(obesity OR body mass index) AND (child OR children OR kid OR kids OR youth OR youths OR pediatric OR paediatrics OR teen OR teens OR teenager OR tween OR teens OR teenagers) AND (ng OR running OR bicycling OR dancing OR soccer OR rugby OR baseball OR basketball OR swimming OR hopscotch OR football OR training OR fitness OR lifting OR physical OR games) AND su(clinical trials) AND pd(>19891231) | ProQuest Dissertations & Theses: Health & Medicine | 2 | |
| S2 | su(obesity) AND (child OR children OR kid OR kids OR youth OR youths OR pediatric OR paediatrics OR teen OR teens OR teenager OR tween OR teens OR teenagers) AND (ng OR running OR bicycling OR dancing OR soccer OR rugby OR baseball OR basketball OR swimming OR hopscotch OR football OR training OR fitness OR lifting OR physical OR games) AND su(clinical trials) AND pd(>19891231) | ProQuest Dissertations & Theses: Health & Medicine | 2 | |
| S1 | su(obesity) AND (child OR children OR kid OR kids OR youth OR youths OR pediatric OR paediatrics OR teen OR teens OR teenager OR tween OR tweens OR teenagers) AND (ng OR running OR bicycling OR dancing OR soccer OR rugby OR baseball OR basketball OR swiming OR hopscotch OR football OR training OR fitness OR lifting OR physical OR games) AND su(clinical trials) AND pd(>19891231) | ProQuest Dissertations & Theses: Health & Medicine | 2 | |
