# Supplementary material for: Effects of exercise on BMI z-score in overweight and obese children and adolescents: a systematic review with meta-analysis
Source: BMC Pediatr. 2014 Sep 9;14:225. doi: 10.1186/1471-2431-14-225 (PMC4180550; doi:10.1186/1471-2431-14-225)
Supplement: Supplementary file 3 — Additional file 3: Studies excluded, including reasons. (DOCX 742 KB) [file 12887_2014_1161_MOESM3_ESM.docx]

Additional File 2. Studies excluded, including reasons.

(1) Weight gain associated with intensive therapy in the diabetes control and complications trial. The DCCT Research Group. Diabetes Care 1988 July;11(7):567-73. Not an exercise intervention study
Ref ID: 2329

(2) Noticeboard. Lancet 1992 February 15;339(8790):421. Not an exercise intervention study
Ref ID: 3341

(3) CURRENT AWARENESS IN GERIATRIC PSYCHIATRY. International Journal of Geriatric Psychiatry 1993 July;8(7):619-26. Not an exercise intervention study
Ref ID: 3342

(4) Current awareness in human psychopharmacology. Human Psychopharmacology: Clinical & Experimental 1994 November;9(6):451-8. Review article
Ref ID: 3343

(5) Progestogen-only contraceptives during lactation: I. Infant growth. World Health Organization Task force for Epidemiological Research on Reproductive Health; Special Programme of Research, Development and Research Training in Human Reproduction. Contraception 1994;50:35-53. Not a randomized controlled trial (RCT)
Ref ID: 1029

(6) Research Digest. Pediatric Exercise Science 1995 February;7(1):5-11. Abstract
Ref ID: 3881

(7) RESÃšMENES. (Spanish). RESUMED 10[1], 9. 1997. Abstract,
Ref ID: 3344

(8) AMERICAN JOURNAL OF CLINICAL NUTRITION. Journal of Human Nutrition & Dietetics 11[4], 347-361. 1998. Abstract,
Ref ID: 3346

(9) Radiation Oncology. Clinical & Investigative Medicine 1998 December 2;21:S84-S99. Not an exercise intervention study
Ref ID: 3345

(10) [A survey on the physical growth of children under 7 years in the urban and suburban rural areas of nine cities of China in 1995. Coordinating Group of Nine Cities on Physical Growth and Development of Children]. Zhonghua yi xue za zhi 1998;78:187-91. Not a randomized controlled trial (RCT), Not an exercise intervention study
Ref ID: 4648

(11) Proceedings of the Interplanetary Society Conference. Port Ludlow, Washington, USA. 30 May-2 June 1997. Dedicated to Paul B. Beeson, M.D. Journal of Infectious Diseases 1999 March 15;179:iv-S390. Did not meet all selection criteria. Off Topic
Ref ID: 544

(12) Poster presentations. Mycoses 1999 March;42(3):165-223. Abstract
Ref ID: 545

(13) Proceedings of the 1st Workshop on Insulin-dependent Diabetes Mellitus in Children and Adolescents. Amman, Jordan, 26-28 October 1997. Acta Paediatrica Supplement 1999 January 3;88(s427):1-51. Abstract
Ref ID: 3350

(14) Swedish Orthopedic Society. Acta Orthopaedica Scandinavica 1999 October 2;70:47. Abstract
Ref ID: 3348

(15) 3rd Baltic bone and cartilage conference. Acta Orthopaedica Scandinavica 1999 October 2;70:1. Abstract
Ref ID: 3347

(16) In defence of the Milquetoast phenotype. Lancet 1999 May 29;353(9167):1884-5. Editorial or letter or comment
Ref ID: 3349

(17) CORRESPONDENCE. Lancet 2000 July 15;356(9225):247. Editorial or letter or comment
Ref ID: 3354

(18) Consensus conference on osteoporosis: Prevention, diagnosis and treatment. Rev Esp Enferm Metab Oseas 2000;9(6):231-9. Review article
Ref ID: 3185

(19) Current Awareness. Human Psychopharmacology: Clinical & Experimental 2000 April;15(3):221-6. Abstract
Ref ID: 520

(20) New Millennium Research to Practice - Conference Abstracts: 11th World Congress of the International Association for the Scientific Study of Intellectual Disabilities (IASSID), 1-6 August 2000 Seattle, Washington, USA. Journal of Intellectual Disability Research 44[3/4], 189-528. 2000. Abstract,
Ref ID: 3793

(21) Occupational and Environmental Allergy. Allergy 55, 152-170. 8-2-2000. Abstract,
Ref ID: 504

(22) Current literature. Journal of Human Nutrition & Dietetics 13[5], 373-386. 2000. Abstract,
Ref ID: 3351

(23) Subject Index for Volume 3. Nutrition in Clinical Care 2000 November;3(6):407. Abstract
Ref ID: 499

(24) Other topics. Allergy 55, 239-282. 8-2-2000. Abstract,
Ref ID: 3352

(25) British HIV Association (BHIVA) guidelines for the treatment of HIV-infected adults with antiretroviral therapy. HIV Medicine 2000 April;1(2):76-101. Not an exercise intervention study
Ref ID: 3355

(26) Proceedings of the South-Eastern Organ Procurement Foundation meeting. 16 September 1999, Memphis, Tennessee, USA. Clinical Transplantation 2000 August 2;14:365-438. Not an exercise intervention study
Ref ID: 3353

(27) Poster Group 1 - Paediatrics. Allergy 56, 173-195. 11-2-2001. Wiley-Blackwell. Abstract,
Ref ID: 480

(28) Nutritional management of HIV in the era of highly active antiretroviral therapy: a review of treatment strategies. Australian Journal of Nutrition & Dietetics 2001 December;58(4):224. Review article
Ref ID: 3356

(29) Keyword index to volume 25. International Journal of Obesity & Related Metabolic Disorders 2001 December;25(12):1865. Abstract
Ref ID: 478

(30) Posters Group 2 - Environmental and Occupational Health. Allergy 56, 235-250. 11-2-2001. Wiley-Blackwell. Abstract,
Ref ID: 3357

(31) Track 5 Physical activity, exercise and sports P130-P151. International Journal of Obesity & Related Metabolic Disorders 2001 May 3;25:S68. Abstract
Ref ID: 490

(32) NASPE ABSTRACTS. Pacing & Clinical Electrophysiology 2001 April 15;24(4P2):539-737. Abstract
Ref ID: 3882

(33) Clinical Practice Guideline: Treatment of the School-Aged Child With Attention-Deficit/Hyperactivity Disorder. Pediatrics 2001 October;108(4):1033. Review article
Ref ID: 3360

(34) PEDIATRICS electronic pages. Pediatrics 2001 November;108(5):1193. Abstract
Ref ID: 3358

(35) What's new in the journals? Diabetic Medicine 2001 October 15;18:10-3. Abstract
Ref ID: 3359

(36) Guidelines for Referral to Pediatric Surgical Specialists. Pediatrics 2002 July;110(1):187. Survey or questionnaire
Ref ID: 446

(37) Poster Discussion Session 12: Asthma mechanisms, treatment and quality of life. Allergy 2002 July 2;57:317-23. Abstract
Ref ID: 3365

(38) The XXXIV Nordic Meeting of Gastroenterology. Scandinavian Journal of Gastroenterology Supplement 2002 June 2;37:2-38. Abstract
Ref ID: 3367

(39) Abstracts 401-450. Journal of Sleep Research 2002 June 2;11:201-24. Abstract
Ref ID: 3366

(40) Abstracts of the 14th Annual Meeting of the German Society of Andrology (DGA) Jena, Germany, 5â€“7 September 2002. Andrologia 34[4], 263-287. 2002. Abstract,
Ref ID: 3364

(41) Abstracts from IMCAS, Paris, January 2002. Journal of Cosmetic & Laser Therapy 4[3/4], 93. 2002. Abstract,
Ref ID: 3361

(42) Study makes strong case for early diabetes intervention. Disease Management Advisor 2002 May;8(5):65-8. Review article
Ref ID: 1819

(43) Abstracts of the Paediatrics and Child Health Division, RACP Annual Scientific Meeting, May 2002. Journal of Paediatrics & Child Health 2002 October;38(5):A1-A18. Abstract
Ref ID: 3363

(44) Neurochemical imaging (MR spectroscopy) in neuropsychiatric disorders â€“ perspectives and limitations. European Archives of Psychiatry & Clinical Neuroscience 2002 October 2;252(5):i1. Not an exercise intervention study
Ref ID: 3362

(45) Education Fuels Budget Debate. Techniques: Connecting Education & Careers 2002 May;77(5):6. Editorial or letter or comment
Ref ID: 3794

(46) Women's Health LiteratureWatch. Journal of Women's Health & Gender-Based Medicine 2002 January;11(1):89-93. Review article
Ref ID: 473

(47) Policy Statements Adopted by the Governing Council of the American Public Health Association, October 24, 2001. American Journal of Public Health 2002 March;92(3):451-83. Review article
Ref ID: 3796

(48) New Technology Program Grants from the Department of Education. Techniques: Connecting Education & Careers 2002 May;77(5):6. Review article
Ref ID: 3795

(49) Conference Abstracts. Fertility Weekly , 20-27. 2-4-2002. Abstract,
Ref ID: 3371

(50) Friday, 19 April 2002. - Plenary lecture. Acta Psychiatrica Scandinavica 105, 32-52. 4-2-2002. Wiley-Blackwell. Abstract,
Ref ID: 3368

(51) Author Index. Clinical Endocrinology 2002 June;56(6):829-38. Abstract
Ref ID: 450

(52) American Transplant Congress. Day-at-a-Glance, Tuesday, April 30, 2002. American Journal of Transplantation 2002 April 2;2:102-29. Abstract
Ref ID: 3370

(53) American Transplant Congress. Day-at-a-Glance, Sunday, April 28,2002. American Journal of Transplantation 2002 April 2;2:49-71. Abstract
Ref ID: 3369

(54) CHRONIC DAILY HEADACHE. Headache: The Journal of Head & Face Pain 43[10], 1128. 2003. Review article,
Ref ID: 3381

(55) PEDIATRIC HEADACHE. Headache: The Journal of Head & Face Pain 43[10], 1126. 2003. Not an exercise intervention study,
Ref ID: 3380

(56) MIGRAINE: PATHOPHYSIOLOGY. Headache: The Journal of Head & Face Pain 43[10], 1125. 2003. Not an exercise intervention study,
Ref ID: 3379

(57) Abstracts of the 24th Annual Scientific Meeting of the High Blood Pressure Research Council of Australia Inc. Clinical & Experimental Pharmacology & Physiology 30[7], A31-A68. 2003. Abstract,
Ref ID: 3382

(58) Correct answers to multiple choice questions appearing in the European Urology Update Series 2002. BJU International 2003 January 15;91(2):155-73. Not an exercise intervention study
Ref ID: 3385

(59) Physicians Poster Session: Supportive Care and Regulatory Issues. Bone Marrow Transplantation 2003 March 2;31(5):S216. Abstract
Ref ID: 3384

(60) 16th Annual Meeting of the European Musculo-Skeletal Oncology Society & 4th Symposium of the EMSOS Nurse Group, 7-9 May 2003, Budapest: Abstracts. Sarcoma 7[2], 93. 2003. Abstract,
Ref ID: 3383

(61) Abstracts of the Annual Meeting of the Thoracic Society of Australia and New Zealand. 4-9 April 2003, Adelaide, Australia. Respirology 2003 November 3;8:A1-A67. Abstract
Ref ID: 3375

(62) Safety Assessment of Salicylic Acid, Butyloctyl Salicylate, Calcium Salicylate, C12-15 Alkyl Salicylate, Capryloyl Salicylic Acid, Hexyldodecyl Salicylate, Isocetyl Salicylate, Isodecyl Salicylate, Magnesium Salicylate, MEA-Salicylate, Ethylhexyl.. International Journal of Toxicology (Taylor & Francis) 2003 November 15;22:1-108. Review article
Ref ID: 3373

(63) Author Index to Volume 64. Kidney International 64[6], 2351-2374. 2003. Nature Publishing Group. Abstract,
Ref ID: 3372

(64) Sister Societies. Journal of the European Academy of Dermatology & Venereology 17, 443. 11-2-2003. Abstract,
Ref ID: 3376

(65) MIGRAINE: TREATMENT. Headache: The Journal of Head & Face Pain 43[10], 1122. 2003. Review article,
Ref ID: 3378

(66) Saturday 5 April â€“ Respiratory Nurses SIG Oral Presentations (1030â€“1200). Respirology 8, A1-A65. 11-3-2003. Wiley-Blackwell. Abstract,
Ref ID: 3374

(67) Abstracts. Alternative Medicine Review 8[4], 453-466. 2003. Abstract,
Ref ID: 3377

(68) Saturday 20 March â€“ Respiratory Nurses SIG Oral Session (1030-1230). Respirology 2004 March 3;9:A1-A75. Abstract
Ref ID: 3403

(69) CITATIONS AND CLINICIANS' NOTES: MICRONUTRIENTS. Current Medical Literature: Clinical Nutrition 13[2], 31-32. 2004. Abstract,
Ref ID: 3402

(70) Physical Health and Profound, Multiple Disabilities. Journal of Intellectual Disability Research 48[4/5], 340-367. 2004. Abstract,
Ref ID: 3797

(71) American Transplant Journal Day at a Glance. American Journal of Transplantation 4, 51-157. 5-2-2004. Wiley-Blackwell. Abstract,
Ref ID: 3395

(72) Oral rehydration therapy is the mainstay of treatment for infectious diarrhoea in children. Drugs & Therapy Perspectives 2004 January;20(1):7-11. Abstract
Ref ID: 3407

(73) Track 7C: Physical activity. International Journal of Obesity & Related Metabolic Disorders 28, S205-S212. 5-2-2004. Abstract,
Ref ID: 3401

(74) CITATIONS AND CLINICIANS' NOTES: MlCRONUTRIENTS. Current Medical Literature: Clinical Nutrition 2004 July;13(3):50-3. Not an exercise intervention study
Ref ID: 3393

(75) CITATIONS AND CLINICIANS' NOTES: HYPERTENSION -- COMPLICATIONS. Current Medical Literature: Nephrology & Hypertension 2004 June;10(2):43-6. Not an exercise intervention study
Ref ID: 3394

(76) Abstracts cont. Clinical Microbiology & Infection 10, 179-273. 5-2-2004. Wiley-Blackwell. Abstract,
Ref ID: 3398

(77) Track 6a: Prevention and etiology - prevention and methods. International Journal of Obesity & Related Metabolic Disorders 28, S162-S166. 5-2-2004. Abstract,
Ref ID: 3400

(78) Track 5a: Management - obesity management. International Journal of Obesity & Related Metabolic Disorders 28, S133-S151. 5-2-2004. Abstract,
Ref ID: 3399

(79) Part One - The primary headaches. Cephalalgia (Wiley-Blackwell) 24, 23-136. 5-2-2004. Wiley-Blackwell. Not an exercise intervention study,
Ref ID: 3396

(80) Apulo-Lucania Sections Meeting of the Italian Neurological Society and the Italian Society of Hospital Neurosciences: Foggia, Italy November 13â€“15, 2003. Neurological Sciences 2004 February;24(6):428-41. Abstract
Ref ID: 3405

(81) B. Renal Disease and Failure. Kidney 2004 November;13(6):263-9. Abstract
Ref ID: 3390

(82) 18th Meeting of the Austrian Society of Transplantation, Transfusion and Genetics. European Surgery: ACA Acta Chirurgica Austriaca 2004 November 3;36:1-42. Abstract
Ref ID: 3389

(83) Abstracts of the 40th Annual Scientific Meeting of the Australian and New Zealand Society of Nephrology. Adelaide, Australia, 1-3 September 2004. Nephrology 2004 August 2;9:A1-A46. Abstract
Ref ID: 3392

(84) Endoscopy. Journal of Gastroenterology & Hepatology 19, A223-A273. 10-2-2004. Wiley-Blackwell. Survey or questionnaire,
Ref ID: 3391

(85) No effect of rosiglitazone for treatment of HIV-1 lipoatrophy: randomised, double-blind, placebo-controlled trial. Lancet 2004 February 7;363(9407):429-38. Drug intervention study
Ref ID: 3404

(86) Correct answers to multiple choice questions appearing in the European Urology Update Series 2003. BJU International 93[2], 228-246. 1-15-2004. Not an exercise intervention study,
Ref ID: 3406

(87) 14th European congress of clinical microbiology and infectious diseases, prague, czech republic, 1-4 may 2004. Clinical Microbiology & Infection 10, 1-86. 5-2-2004. Wiley-Blackwell. Abstract,
Ref ID: 3397

(88) Index. Alimentary Pharmacology & Therapeutics 2004 December;20(11/12):1391-406. Abstract
Ref ID: 3386

(89) Accumulated Table of Contents. Kidney International 66[6], 2516-2535. 2004. Nature Publishing Group. Abstract,
Ref ID: 3388

(90) Author Index to Volume 66. Kidney International 66[6], 2493-2515. 2004. Nature Publishing Group. Abstract,
Ref ID: 3387

(91) Abstracts of the 26th Annual Scientific Meeting of the High Blood Pressure Research Council of Australia, 24-26 November 2004. Clinical & Experimental Pharmacology & Physiology 2005 July;32(7):A1-A27. Abstract
Ref ID: 3420

(92) Late effects and quality of life. Bone Marrow Transplantation 2005 March 17;35:183-94. Not an exercise intervention study
Ref ID: 3421

(93) L-Carnitine. Alternative Medicine Review 2005 March;10(1):42-50. Review article
Ref ID: 3422

(94) Correct answers to multiple choice questions appearing in the European Urology Update Series 2004. BJU International 95[2], 250-269. 1-15-2005. Wiley-Blackwell. Not an exercise intervention study,
Ref ID: 3423

(95) CONGRES ANNUEL DE RECHERCHE DERMATOLOGIQUE (CARD) FRENCH-SPEAKING CONGRESS OF DERMATOLOGICAL RESEARCH BREST (FRANCE), MAY 27â€“28, 2005. Journal of Investigative Dermatology 2005 Dec;A13-A24.
Ref ID: 3410¬

Abstract

(96) CHRONIC OBSTRUCTIVE PULMONARY DISEASE. Current Medical Literature: Respiratory Medicine 19[4], 86-93. 2005. Abstract,
Ref ID: 3411

(97) MACRONUTRIENTS. 14 ed. Remedica Medical Education & Publishing; 2005. p. 12-5.¬

Not an exercise intervention study
Ref ID: 3426

(98) PODIUM AND MODERATED POSTER SESSION ABSTRACTS. Journal of Sexual Medicine 2, 3-38. 1-2-2005. Wiley-Blackwell. Abstract,
Ref ID: 3424

(99) UNMODERATED POSTER SESSION ABSTRACTS. Journal of Sexual Medicine 2, 39-87. 1-2-2005. Wiley-Blackwell. Abstract,
Ref ID: 3425

(100) Colon, Rectum and Small Bowel. Journal of Gastroenterology & Hepatology 2005 September 2;20:A13-A40. Abstract
Ref ID: 3415

(101) Proceedings of the 2nd International Conference on New Insights in Progression of Chronic Kidney Disease, Barcelona, Spain, May 8-10, 2005. Kidney International Supplement 2005 December 2;(99):S-1. Abstract
Ref ID: 3408

(102) Nutrition and growth in kidney disease. Nephrology 2005 December 2;10:S177-S230. Review article
Ref ID: 3409

(103) 19th Meeting of the Austrian Society of Transplantation, Transfusion, and Genetics, October 26â€“28, 2005. European Surgery: ACA Acta Chirurgica Austriaca 2005 October 3;37:1-46. Abstract
Ref ID: 3412

(104) Diet, Lifestyle, and Disease. Current Medical Literature: Clinical Nutrition 2005 October;14(4):76-84. Abstract
Ref ID: 3413

(105) Oral Abstracts. Australian & New Zealand Journal of Obstetrics & Gynaecology 45, A1-A19. 9-2-2005. Wiley-Blackwell. Abstract,
Ref ID: 3414

(106) Free Communications, Poster Presentations: Student Poster Award Finalists. Journal of Athletic Training 40, S-95. 4-2-2005. Abstract,
Ref ID: 3799

(107) Folic Acid. Alternative Medicine Review 2005 September;10(3):222-9. Diet Intervention or Supplement Study
Ref ID: 3417

(108) C. Hypertension and Systemic Disease. Kidney 2005 September;14(5):224-30. Not an exercise intervention study
Ref ID: 3418

(109) Clinical Science. Epilepsia (Series 4) 2005 August;46(8):1158-61. Animal study, Diet Intervention Study
Ref ID: 3419

(110) SATELLITE SYMPOSIA. Journal of the European Academy of Dermatology & Venereology 2005 September 2;19:1-17. Abstract
Ref ID: 3416

(111) 1st IASSID Asian-Pacific Conference Taipei, Taiwan June 12â€“15, 2005 Abstracts. Journal of Policy & Practice in Intellectual Disabilities 2005 September;2(3/4):176-220. Abstract
Ref ID: 3798

(112) Intense diet, behavior, and physical activity intervention effective for obese children. Journal of Family Practice 2005;54:579. No exercise only group, Diet Intervention Study
Ref ID: 4649

(113) Abstracts from the 24th Annual Scientific Meeting. Immunology & Cell Biology 2006 June;84(3):A1-A34. Abstract
Ref ID: 3434

(114) 47th Annual Meeting of the Austrian Society of Surgery, Vienna, June 15â€“17, 2006. European Surgery: ACA Acta Chirurgica Austriaca 2006 June 2;38:1-121. Abstract
Ref ID: 3433

(115) Abstracts of the 10th Meeting of the Italian Peripheral Nerve Study Group. Journal of the Peripheral Nervous System 2006 June;11(2):179-208. Abstract
Ref ID: 3435

(116) Speaker Abstracts. Isokinetics & Exercise Science 2006 June;14(2):111-52. Abstract
Ref ID: 3883

(117) Proceedings of the Dutch Society of Clinical Pharmacology and Biopharmacy meeting of October 4th 2005. British Journal of Clinical Pharmacology 61[4], 475-486. 2006. Wiley-Blackwell. Abstract,
Ref ID: 3436

(118) Clinical Renal Pharmacology and Therapeutics. Kidney 15[6], 283-292. 2006. Abstract,
Ref ID: 3429

(119) 5-Methyltetrahydrofolate. Alternative Medicine Review 2006 December;11(4):330-7. Review article
Ref ID: 3428

(120) Oral Communications. Diabetic Medicine 2006 December 2;23:612-9. Not an exercise intervention study
Ref ID: 3427

(121) SSS abstracts. British Journal of Surgery 93[9], 1153-1176. 2006. Abstract,
Ref ID: 3432

(122) Parallel sessions: oral presentations. Liver International 2006 September 2;26:11-22. Abstract
Ref ID: 3431

(123) Hepatology. Journal of Gastroenterology & Hepatology 2006 October 3;21:A300-A334. Abstract
Ref ID: 3430

(124) Prevention of Boar Taint in Pig Production: Abstracts of the 19th Symposium of the Nordic Committee for Veterinary Scientific Cooperation Gardermoen, Norway. 21-22 November 2005. Acta Veterinaria Scandinavica 2006 January 2;48:1-3. Abstract, Animal study
Ref ID: 3437

(125) EAST-EUROPEAN SYMPOSIUM â€œCENTRAL AND PERIPHERAL SYNAPTIC TRANSMISSIONâ€ October 5â€“9, 2005, Varna, Bulgaria. Autonomic & Autacoid Pharmacology 26[1], 31-120. 2006. Wiley-Blackwell. Abstract,
Ref ID: 3438

(126) Circadian Rhythms. Sleep & Biological Rhythms 2007 August 2;5:A22-A31. Abstract
Ref ID: 3445

(127) Recent Abstracts. Alternative Medicine Review 12[2], 174-188. 2007. Abstract,
Ref ID: 3446

(128) Swiss Society of Surgery. British Journal of Surgery 94[6], 761-784. 2007. Abstract,
Ref ID: 3447

(129) An Access-Dictionary of Internationalist High Tech Latinate English. Third Education Group Review 3, 1-123. 2007. Third Education Group. Not an exercise intervention study,
Ref ID: 3800

(130) Immune reconstitution inflammatory syndrome (IRIS): review ofcommon infectious manifestations and treatment options. AIDS Research & Therapy 2007 January;4:9-18. Review article
Ref ID: 3450

(131) Poster abstracts. Transfusion Alternatives in Transfusion Medicine 2007 March 2;9:31-59. Abstract
Ref ID: 3448

(132) 43rd Workshop for Paediatric Research. European Journal of Pediatrics 2007 March;166(3):273-90. Abstract
Ref ID: 3449

(133) 21st Meeting of the Austrian Society of Transplantation, Transfusion and Genetics. St. Wolfgang, October 17â€“20, 2007. European Surgery: ACA Acta Chirurgica Austriaca 39, 1-43. 10-3-2007. Abstract,
Ref ID: 3440

(134) IMMUNE ENHANCEMENT AND CANCER PREVENTION OF A NEW STRAIN OF PROBIOTIC LACTIC ACID BACTERIUM (BIFIDOBACTERIUM LACTIS HN019). Journal of the Society for Integrative Oncology 2007;5(4):181. Diet Intervention or Supplement Study
Ref ID: 3442

(135) Nutrition. Journal of Gastroenterology & Hepatology 2007 October 3;22:A403-A412. Abstract
Ref ID: 3441

(136) Effective dietary interventions for overweight and obese children. Australian Nursing Journal 2007 June;14(11):31-4. Review article
Ref ID: 1210

(137) 41st Annual Meeting of the Germanâ€“Speaking Mycological Society (DMykG). Mycoses 2007 September;50(5):334-82. Abstract
Ref ID: 3444

(138) Hot Papers in the Literature. Journal of Women's Health (15409996) 2007 September;16(7):1076-84. Abstract
Ref ID: 3443

(139) Subject index. BJU International 2007 December;100(6):1451-63. Abstract
Ref ID: 3439

(140) Ocular and Aural Disorders. Current Medical Literature: Pediatrics 21[4], 138-141. 2008. Abstract,
Ref ID: 3451

(141) International Stroke Conference 2008. Clinician Reviews 2008 April;18(4):47-51. Abstract
Ref ID: 3461

(142) 49th Annual Meeting of the Austrian Society of Surgery, Innsbruck, May 21â€“23, 2008 (Guest Editors: Beate Neuhauser, Dietmar Ã–fner, Elfriede Ruttmann-Ulmer, and Anton Schwabegger). European Surgery: ACA Acta Chirurgica Austriaca 40, 1-131. 4-5-2008. Abstract,
Ref ID: 3460

(143) Oral program. Nutrition & Dietetics 2008 May 2;65:A1-A24. Abstract
Ref ID: 3459

(144) Management -- Complications and Comorbidities. Current Medical Literature: Diabetes 2008 March;25(1):29-33. Abstract
Ref ID: 3462

(145) Vitamin C in plasma is inversely related to blood pressure and change in blood pressure during the previous year in young Black and White women. Nutrition Journal 2008 January;7:35-43. Cross-sectional study
Ref ID: 3465

(146) The prince and the pauper. A tale of anticancer targeted agents. Molecular Cancer 2008 January;7:82-114. Review article
Ref ID: 3464

(147) A. Diagnosis, Pathology, and Pathophysiology. Kidney 2008 March;17(2):74-9. Not an exercise intervention study
Ref ID: 3463

(148) CONTRIBUTIONS FROM INTERNATIONAL CONGRESS. High Blood Pressure & Cardiovascular Prevention 15[3], 171-222. 2008. Abstract,
Ref ID: 3454

(149) European Tissue Repair Society: Joint Meeting with the Tissue Viability Unit of Malta. Wound Repair & Regeneration 2008 November;16(6):A66-A82. Abstract
Ref ID: 3453

(150) Keyword index. Neurogastroenterology & Motility 2008 November 2;20:154-9. Abstract
Ref ID: 3452

(151) Oral presentations. Clinical Microbiology & Infection 2008 July 2;14:S1-S119. Abstract
Ref ID: 3455

(152) GH treatment. Current Medical Literature: Growth, Growth Hormone, & Metabolism 2008 June;2(2):59-61. Abstract
Ref ID: 3458

(153) Lectures. Acta Neuropsychiatrica 2008 June 2;20:1-50. Abstract
Ref ID: 3457

(154) Posters. Clinical Microbiology & Infection 2008 July 2;14:S121-S666. Abstract
Ref ID: 3456

(155) Congres Annuel De Recherche Dermatologique (CARD) French-Speaking Congress of Dermatological Research Toulouse (France), 12â€“13 September 2008. Journal of Investigative Dermatology 129[3], 792-809. 2009. Abstract,
Ref ID: 3468

(156) Unmoderated Posters June 28, 2009, 1200-June 30, 2009, 1030. Canadian Urological Association Journal 3, S61-S82. 6-2-2009. Abstract,
Ref ID: 3467

(157) 23rd Meeting of the Austrian Society of Transplantation, Transfusion and Genetics. Seefeld, October 21â€“23, 2009. Guest Editor: Walter Mark, Innsbruck, Austria. European Surgery: ACA Acta Chirurgica Austriaca 41, 1-38. 10-2-2009. Abstract,
Ref ID: 3466

(158) 6th Meeting of the American Academy of Veterinary Nutrition, Seattle, WA, USA June 6, 2007 Abstracts. Journal of Animal Physiology and Animal Nutrition 2009;93(2):141-5. Abstract, Animal study
Ref ID: 5107

(159) TEMAS EN CARTEL. (Spanish). Revista Cubana de AlimentaciÃ³n y NutriciÃ³n 2010 February;20:S24-S129. Abstract
Ref ID: 3479

(160) 43. Jahrestagung Physiologie u. Pathologie der Fortpflanzung, gleichzeitig 35. VeterinÃ¤r-Humanmedizinische Gemeinschaftstagung MÃ¼nchen, 24. â€“ 26. Februar 2010. Reproduction in Domestic Animals 2010 February 2;45:1-61. Abstract, Animal study
Ref ID: 3478

(161) Keynote Abstracts. Journal of Intellectual Disability Research 2010 October;54(10):881-4. Abstract
Ref ID: 3473

(162) Insulin sensitivity is improved with vitamin D supplementation in South Asian women who are vitamin D deficient and insulin resistant - a randomised, placebo-controlled trial. Australasian Medical Journal 2[1], 55-56. 2010. Abstract,
Ref ID: 3480

(163) COMUNICACIONES PÃ“STER. (Spanish).: Sociedad Espanola de Nutricion Parenteral y Enteral; 2010 p. 477-94.¬

Abstract
Ref ID: 3476

(164) Design of a family-based lifestyle intervention for youth with type 2 diabetes: the TODAY study. Int J Obes (Lond) 2010 February;34(2):217-26. Description versus conduct of study
Ref ID: 91

(165) Laxative Abuse. Drugs 2010 August;70(12):1487-503. Review article
Ref ID: 3474

(166) Dislipidemia y virus de inmunodeficiencia adquirida/sida. (Spanish). Revista Cubana de EndocrinologÃ­a 2010 May;21(2):202-22. Not an exercise intervention study
Ref ID: 3477

(167) Workshop Abstracts. Basic & Clinical Pharmacology & Toxicology 2010 July 2;107:71-111. Abstract
Ref ID: 3475

(168) Other complementary therapies. Focus Altern Complement Ther 2010;15(2):175-9. Review article
Ref ID: 3186

(169) Clinical Practice Clinical Practice Clinical Practice. Journal of Gastroenterology & Hepatology 2010 October 2;25:A18-A43. Abstract
Ref ID: 3472

(170) Oseltamivir in Seasonal, Avian H5N1 and Pandemic 2009 A/H1N1 Influenza. Clinical Pharmacokinetics 2010 November;49(11):741-65. Not an exercise intervention study
Ref ID: 3471

(171) Recently Published Abstracts. Alternative Medicine Review 15[4], 369-380. 2010. Abstract,
Ref ID: 3469

(172) Contents / Sommaire. Applied Physiology, Nutrition & Metabolism 2010 December;35(6):C-1. Abstract
Ref ID: 3470

(173) Nutrition and cancer: from prevention to nutritional support, 8th October 2010, Milan. Ecancermedicalscience 2010 January;4:1-26. Review article
Ref ID: 3482

(174) Proceedings of the VI National Congress of Pharmacology October 2009 â€“ Posters. Autonomic & Autacoid Pharmacology 2010 January;30(1):1-65. Abstract
Ref ID: 3481

(175) Assessment of a two-year school-based physical activity intervention among 7-9-year-old children. International Journal of Behavioral Nutrition & Physical Activity 2011 January;8(1):138-50. Diet & Exercise intervention, Not All Participants were Overweight and/or Obese
Ref ID: 3491

(176) Regular consumption of vitamin D-fortified yogurt drink (Doogh) improved endothelial biomarkers in subjects with type 2 diabetes: a randomized double-blind clinical trial. BMC Medicine 2011 January;9(1):125-34. Diet Intervention or Supplement Study
Ref ID: 3488

(177) The mPED randomized controlled clinical trial: applying mobile persuasive technologies to increase physical activity in sedentary women protocol. BMC Public Health 2011 January;11(1):933-40. Study limited to adults
Ref ID: 3489

(178) Molecular targets and oxidative stress biomarkers in hepatocellular carcinoma: an overview. Journal of Translational Medicine 2011 January;9(1):171-84. Not an exercise intervention study
Ref ID: 3492

(179) Postprandial hyperlipidemia, endothelial dysfunction and cardiovascular risk: focus on incretins. Cardiovascular Diabetology 2011 January;10(1):61-71. Review article
Ref ID: 3490

(180) A higher response of plasma neuropeptide Y, growth hormone, leptin levels and extracellular glycerol levels in subcutaneous abdominal adipose tissue to Acipimox during exercise in patients with bulimia nervosa: single-blind, randomized, microdialysis.. Nutrition & Metabolism 2011 January;8(1):81-94. Study limited to adults
Ref ID: 3494

(181) Causal assessment of dietary acid load and bone disease: a systematic review & meta-analysis applying Hill's epidemiologic criteria for causality. Nutrition Journal 2011 January;10(1):41-63. Review article
Ref ID: 3495

(182) Concurrent Session 1: Fatty Acids. Australasian Medical Journal 4[12], 791-794. 2011. Abstract,
Ref ID: 3483

(183) What is New in Preventive Medicine? International Journal of Preventive Medicine 2[3], 190-200. 2011. Abstract,
Ref ID: 3484

(184) Management of Patients with Psoriasis in Japan. Advances in Psoriasis & Inflammatory Skin Diseases 2011 June;2(4):126-31. Review article
Ref ID: 3485

(185) Four variants in transferrin and HFE genes as potential markers of iron deficiency anaemia risk: an association study in menstruating women. Nutrition & Metabolism 2011 January;8(1):69-76. Cross-sectional study
Ref ID: 3493

(186) Aromatase inhibitors in men: effects and therapeutic options. Reproductive Biology & Endocrinology 2011 January;9(1):93-9. Drug intervention study
Ref ID: 3496

(187) Dietitians Association of Australia 29. Nutrition & Dietetics 2011 May 2;68:1-22. Abstract
Ref ID: 3486

(188) A randomized study of alglucosidase alfa in late-onset Pompes disease. Current Medical Literature: Lysosomal Storage Disease 2011 March;9(1):32-3. Drug intervention study
Ref ID: 3487

(189) A randomized controlled trial on the effectiveness of strength training on clinical and muscle cellular outcomes in patients with prostate cancer during androgen deprivation therapy: rationale and design. BMC Cancer 2012 January;12(1):123-32. Description versus conduct of study
Ref ID: 3498

(190) Phenylketonuria: nutritional advances and challenges. Nutrition & Metabolism 2012 January;9(1):7-13. Diet Intervention Study
Ref ID: 3500

(191) Metabolomic analysis of rat serum in streptozotocin-induced diabetes and after treatment with oral triethylenetetramine (TETA). Genome Medicine 2012 March;4(3):35-49. Animal study
Ref ID: 3497

(192) Can a standard dose of eicosapentaenoic acid (EPA) supplementation reduce the symptoms of delayed onset of muscle soreness? Journal of the International Society of Sports Nutrition 2012 January;9(1):2-11. Diet Intervention or Supplement Study
Ref ID: 3884

(193) National Student Conference of the Canadian Society for Epidemiology and Biostatistics, Saskatoon, Canada, May 13-14, 2012 Abstracts. American Journal of Epidemiology 2012;176(1):80. Abstract
Ref ID: 5108

(194) Effects of a vildagliptin/metformin combination on markers of atherosclerosis, thrombosis, and inflammation in diabetic patients with coronary artery disease. Cardiovascular Diabetology 2012 January;11(1):60-5. Drug intervention study
Ref ID: 3499

(195) -Anon. Habit-reversal behavioral therapy effective for children with Tourette disorder. Journal of the National Medical Association 2010;102:968-9. Not an exercise intervention study
Ref ID: 4650

(196) -Escoto-Ponce-de-León-MC, Mancilla-Díaz JM, Camacho-Ruiz EJ. A pilot study of the clinical and statistical significance of a program to reduce eating disorder risk factors in children. Eating and weight disorders : EWD 2008;13:111-8. Not an exercise intervention study
Ref ID: 4651

(197) Aasvee K, Jauhiainen M, Kurvinen E, Jordania R, Sundvall J, Ehnholm C. Lipoprotein(a), apolipoprotein A-I and B serum levels in young families from Tallinn, Estonia. Relationships with other cardiovascular risk factors and nationality. Scandinavian Journal of Clinical & Laboratory Investigation 1999;59(3):179-89. Cross-sectional study
Ref ID: 5109

(198) Aatola H, Koivistoinen T, Hutri-Kahonen N, Juonala M, Mikkila V, Lehtimaki T, Viikari JS, Raitakari OT, Kahonen M. Lifetime fruit and vegetable consumption and arterial pulse wave velocity in adulthood: the Cardiovascular Risk in Young Finns Study. Circulation 2010 December 14;122(24):2521-8. Cohort Study
Ref ID: 2330

(199) Abdel-Rahman SA, Shaheen AAM. Efficacy of weight bearing exercises on balance in children with Down syndrome. Egyptian Journal of Neurology, Psychiatry and Neurosurgery 2010;47:37-42. Inappropriate Outcomes
Ref ID: 4654

(200) Abell TL, Van CE, Abrahamsson H, Huizinga JD, Konturek JW, Galmiche JP, VoelIer G, Filez L, Everts B, Waterfall WE, Domschke W, Bruley d, V, Familoni BO, Bourgeois IM, Janssens J, Tougas G. Gastric electrical stimulation in intractable symptomatic gastroparesis. Digestion 2002;66(4):204-12. Not an exercise intervention study
Ref ID: 1784

(201) Abete I, Parra D, Crujeiras AB, Goyenechea E, Martinez JA. Specific insulin sensitivity and leptin responses to a nutritional treatment of obesity via a combination of energy restriction and fatty fish intake. Journal of Human Nutrition & Dietetics 2008 December;21(6):591-600. Diet Intervention Study
Ref ID: 3501

(202) Abood DA, Black DR, Coster DC. Evaluation of a school-based teen obesity prevention minimal intervention. J Nutr Educ Behav 2008 May;40(3):168-74. Not an exercise intervention study
Ref ID: 186

(203) Abou-Khalil BW. Lacosamide: What Can Be Expected from the Next New Antiepileptic Drug? Epilepsy Currents 2009 September;9(5):133-4. Drug intervention study
Ref ID: 3502

(204) Aboul-Seoud MA, Aboul-Seoud AL. Estimation of body fat from skinfold thickness. Computer Methods and Programs in Biomedicine 2001;65(3):201-6. Not an exercise intervention study
Ref ID: 5110

(205) Abrams EJ, Matheson PB, Thomas PA, Thea DM, Krasinski K, Lambert G, Shaffer N, Bamji M, Hutson D, Grimm K, Kaul A, Bateman D, Rogers M, Beatrice S, Chiasson MA, Debernardo E, Lawrence K, Mcveigh K, Odonnell R, Oleszko W, Punsalang A, Alford T, Betre A, Cappelli M, Courtland R et al. Neonatal Predictors of Infection Status and Early Death Among 332 Infants at Risk of Hiv-1 Infection Monitored Prospectively from Birth. Pediatrics 1995;96(3):451-8. Not an exercise intervention study
Ref ID: 5111

(206) Abreu RNDCd, Costa FLPd, Brito EMd, Vasconcelos SMM, Escudeiro SdS, Moreira TMM, Monteiro MGS. Pessoas em recuperação do alcoolismo: avaliação dos fatores de risco cardiovasculares^ipt
Personas en la recuperación del alcoholismo: evaluación de los factores de riesgo cardiovascular^ies
People in alcoholism recovery: assessment of cardiovascular risk factors^ien. SMAD, Rev Eletrônica Saúde Mental Álcool Drog 2009 August;5(2):1-14. Not an exercise intervention study
Ref ID: 3919

(207) Abt G, Zhou S, Weatherby R. The effect of a high-carbohydrate diet on the skill performance of midfield soccer players after intermittent treadmill exercise. Journal of Science & Medicine in Sport 1998 December;1(4):203-12. Diet Intervention Study
Ref ID: 2068

(208) Abubakar A, Van d, V, Van BA, Mbonani L, Kalu R, Newton C, Holding P. Socioeconomic status, anthropometric status, and psychomotor development of Kenyan children from resource-limited settings: a path-analytic study. Early human development 2008;84:613-21. Cross-sectional study
Ref ID: 4655

(209) Abubakr A, Wambacq I. Long-term outcome of vagus nerve stimulation therapy in patients with refractory epilepsy. Journal of Clinical Neuroscience 2008 February;15(2):127-9. Retrospective study
Ref ID: 1014

(210) Aburto NJ, Ramirez-Zea M, Neufeld LM, Flores-Ayala R. The effect of nutritional supplementation on physical activity and exploratory behavior of Mexican infants aged 8-12 months. Eur J Clin Nutr 2010 June;64(6):644-51. Not an exercise intervention study, Subjects less than 2 years old
Ref ID: 53

(211) Ackerman IL, Karn CA, Denne SC, Ensing GJ, Leitch CA. Total rut not resting energy expenditure is increased in infants with ventricular septal defects. Pediatrics 1998;102(5):1172-7. Subjects less than 2 years old
Ref ID: 5112

(212) Acosta Garcia E. Vigencia del Síndrome Metabólico^ies
Vigency of Metabolic Syndrome^ien
Vigencia da Sindrome Metabólica^ipt. Acta bioquím clín latinoam 2011 September;45(3):423-30. Review article
Ref ID: 3920

(213) Adair LS, Kuzawa CW, Borja J. Maternal energy stores and diet composition during pregnancy program adolescent blood pressure. Circulation 2001;104(9):1034-9. Not an exercise intervention study
Ref ID: 5113

(214) Adam S, Westenhofer J, Rudolphi B, Kraaibeek HK. Effects of a combined inpatient-outpatient treatment of obese children and adolescents. Obesity Facts 2009;2(5):286-93. Not a randomized controlled trial (RCT)
Ref ID: 621

(215) Adamo KB, Rutherford JA, Goldfield GS. Effects of interactive video game cycling on overweight and obese adolescent health. Appl Physiol Nutr Metab 2010 December;35(6):805-15. No comparative control group
Ref ID: 3

(216) Adamo KB, Sheel AW, Onywera V, Waudo J, Boit M, Tremblay MS. Child obesity and fitness levels among Kenyan and Canadian children from urban and rural environments: a KIDS-CAN Research Alliance Study. International Journal of Pediatric Obesity 2011 June;6(2-2):e225-e232. Not an exercise intervention study
Ref ID: 2332

(217) Adams TD, Avelar E, Cloward T, Crosby RD, Farney RJ, Gress R, Halverson RC, Hopkins PN, Kolotkin RL, Lamonte MJ, Litwin S, Nuttall RT, Pendleton R, Rosamond W, Simper SC, Smith SC, Strong M, Walker JM, Wiebke G, Yanowitz FG, Hunt SC. Design and rationale of the Utah obesity study. A study to assess morbidity following gastric bypass surgery. Contemporary Clinical Trials 2005 October;26(5):534-51. Description versus conduct of study
Ref ID: 1474

(218) Adegboye AR, Anderssen SA, Froberg K, Sardinha LB, Heitmann BL, Steene-Johannessen J, Kolle E, Andersen LB. Recommended aerobic fitness level for metabolic health in children and adolescents: a study of diagnostic accuracy. British Journal of Sports Medicine 2011 July;45(9):722-8. Cross-sectional study
Ref ID: 2333

(219) Adiputra IN. The improvement effect of Modern Balinese Baris Dancing Exercise on body composition, blood pressure and heart rate. Journal of Human Ergology 1994 December;23(2):93-9. Study limited to adults
Ref ID: 2219

(220) Adiputra N, Alex P, Sutjana DP, Tirtayasa K, Manuaba A. Balinese dance exercises improve the maximum aerobic capacity. Journal of Human Ergology 1996 June;25(1):25-9. Study limited to adults
Ref ID: 2148

(221) Adjemian D, Bustos P, Amigo H. Nivel socioeconómico y estado nutricional: un estudio en escolares^ies. Arch latinoam nutr 2007 June;57(2):125-9. Cross-sectional study
Ref ID: 3921

(222) Adkins S, Sherwood NE, Story M, Davis M. Physical activity among African-American girls: the role of parents and the home environment. Obesity Research 2004 September;12:Suppl-45S. Cross-sectional study
Ref ID: 1579

(223) Adu-Afarwuah S, Lartey A, Brown KH, Zlotkin S, Briend A, Dewey KG. Randomized comparison of 3 types of micronutrient supplements for home fortification of complementary foods in Ghana: effects on growth and motor development. American Journal of Clinical Nutrition 86(2):412-20, 2007 Aug 2007;(2):412-20. Diet Intervention or Supplement Study, Not a randomized controlled trial (RCT)
Ref ID: 2872

(224) Aerts D, Chinazzo H, Santos JAd, Oserow NR. Percepção da imagem corporal de adolescentes escolares brancas e não brancas de escolas públicas do Município de Gravataí, Estado do Rio Grande do Sul, Brasil^ipt
Body image perception of white and non-white female adolescents of city's Public Schools in the Municipality of Gravataí, State of Rio Grande do Sul, Brazil^ien. Epidemiol serv saúde 2011 September;20(3):363-72. Cross-sectional study
Ref ID: 3922

(225) AESGP. Herbal medicinal products in the European Union. Pharmaceuticals Policy & Law 1999 June;2(1):55. Not an exercise intervention study
Ref ID: 3503

(226) Africa EK, Van Deventer KJ, Barnard JG. Health risk behaviours of a selected group of adolescent girls. South African Journal for Research in Sport Physical Education and Recreation 2008;30(2):1-14. Survey or questionnaire
Ref ID: 5114

(227) Agah M, Yahyavi P, Roudneshin F. Comparison between classic laryngeal mask and cobra perilaryngeal airway during mechanical ventilation. Tanaffos 2006;5:13-9. Not an exercise intervention study
Ref ID: 4656

(228) Agarwal S, Swanson S, Murphy A, Yaeger K, Sharek P, Halamek LP. Comparing the utility of a standard pediatric resuscitation cart with a pediatric resuscitation cart based on the Broselow tape: a randomized, controlled, crossover trial involving simulated resuscitation scenarios. Pediatrics 2005;116:e326-e333. Not an exercise intervention study
Ref ID: 4657

(229) Agertoft L, Pedersen S. Effects of long-term treatment with an inhaled corticosteroid on growth and pulmonary function in asthmatic children. Respir Med 1994;88:373-81. Drug intervention study
Ref ID: 4658

(230) Agiovlasitis S, Pitetti KH, Guerra M, Fernhall B. Prediction of VO[subscript 2peak] from the 20-m Shuttle-Run Test in Youth with Down Syndrome. Adapted Physical Activity Quarterly 2011 April 1;28(2):146-56. Not an exercise intervention study
Ref ID: 3897

(231) Agrasada GV, Gustafsson J, Kylberg E, Ewald U. Postnatal peer counselling on exclusive breastfeeding of low-birthweight infants: a randomized, controlled trial. Acta Paediatrica 2005;94:1109-15. Study limited to adults
Ref ID: 4659

(232) Agrasada GV, Kylberg E. When and why Filipino mothers of term low birth weight infants interrupted breastfeeding exclusively. Breastfeeding review : professional publication of the Nursing Mothers' Association of Australia 2009;17:5-10. Study limited to adults
Ref ID: 4660

(233) Aguer C, Gavarry O, Gole Y, Boussuges A, Doyard P, Falgairette G. A 5-month weight-reduction programme has a positive effect on body composition, aerobic fitness, and habitual physical activity of severely obese girls: a pilot evaluation study. Journal of Sports Sciences 2010 February;28(3):281-9. Diet & Exercise intervention
Ref ID: 552

(234) Aguilar-Salinas CA, Gómez-Pérez FJ. Declaración de Acapulco: propuesta para la reducción de la incidencia de la diabetes en México^ies
The "Declaración de Acapulco": a proposal to reduce incidence of diabetes in Mexico^ien. Rev invest clín 2006 February;58(1):71-7. Review article
Ref ID: 3923

(235) Aguilar Coronado M, Manrique Rajo L, Tuesta Muñoz M, Musayón Oblitas Y. Depresión y autoestima en adolescentes con obesidad y sobrepeso: un problema que pesa: [revisión]^ies
Depression and self-esteem in teenagers with obesity and overweight: a problem that weighs: [review]^ien. Rev enferm herediana 2010 June;3(1):49-54. Review article
Ref ID: 3924

(236) Aguirre C, Castillo D, Le RO. Desafíos emergentes en la nutrición del adolescente^ies
Emergent challenges in adolescent nutrition^ien. Rev chil pediatr 2010 December;81(6):488-97. Review article
Ref ID: 3925

(237) Ahmed T, Garrigo J, Danta I. Preventing bronchoconstriction in exercise-induced asthma with inhaled heparin. New England Journal of Medicine 1993 July 8;329(2):90-5. Drug intervention study, Acute study
Ref ID: 2261

(238) Ahrens W, Bammann K, de HS, Halford J, Palou A, Pigeot I, Siani A, Sjostrom M, European Consortium of the IDEFICS Project. Understanding and preventing childhood obesity and related disorders--IDEFICS: a European multilevel epidemiological approach. Nutrition Metabolism & Cardiovascular Diseases 2006 May;16(4):302-8. Description versus conduct of study
Ref ID: 1393

(239) Ainslie PN, Campbell IT, Frayn KN, Humphreys SM, Maclaren DP, Reilly T. Physiological and metabolic responses to a hill walk. Journal of Applied Physiology 2002 January;92(1):179-87. Acute study
Ref ID: 1860

(240) Aires L, Santos R, Silva P, Santos P, Oliveira J, Ribeiro JC, Rego C, Mota J. Daily differences in patterns of physical activity among overweight/obese children engaged in a physical activity program. American Journal of Human Biology 2007 November;19(6):871-7. Cross-sectional study
Ref ID: 1154

(241) Aitchison TC, Durnin JV, Beckett C, Pollitt E. Effects of an energy and micronutrient supplement on growth and activity, correcting for non-supplemental sources of energy input in undernourished children in Indonesia. European Journal of Clinical Nutrition 2000 May;54:Suppl-73. Diet Intervention or Supplement Study
Ref ID: 1968

(242) Akber A, Portale AA, Johansen KL. Pedometer-Assessed Physical Activity in Children and Young Adults with CKD. Clinical Journal of the American Society of Nephrology 2012;7(5):720-6. Cross-sectional study
Ref ID: 5115

(243) Akimoto-Gunther L, Hubler M, Santos M, Carolino I, Sonoo N, Botti B, Mota D, Takahachi G. Effects of re-education in eating habits and physical activity on the lipid profile of obese teenagers. Clinical Chemistry & Laboratory Medicine 2002 May;40(5):460-2. Diet & Exercise intervention
Ref ID: 1809

(244) Akkari M, Waisberg G, Braga SR, Yamada HH, Lundberg JS, Goiano EdO, Figueiredo MJPSSd, Santili C. Osteocondrite de Van Neck-Odelberg: relato de 4 casos^ipt
Van neck-odelberg osteochondritis: report on 4 cases^ien. Rev bras ortop 2010 December;45(supl):55-8. Case-Control / Case Study
Ref ID: 3926

(245) Al-Mousawi AM, Williams FN, Mlcak RP, Jeschke MG, Herndon DN, Suman OE. Effects of exercise training on resting energy expenditure and lean mass during pediatric burn rehabilitation. Journal of Burn Care & Research 31(3):400-8, 2010 May-Jun 2010;(3):400-8. Not All Participants were Overweight and/or Obese
Ref ID: 2873

(246) Al-Turkmani MR, Law T, Kellogg MD. Performance evaluation of a particle-enhanced turbidimetric cystatin C assay on the Hitachi 917 analyzer. Clinica Chimica Acta 2008;398(1-2):75-7. Not an exercise intervention study
Ref ID: 5116

(247) Alam S, Afzal K, Maheshwari M, Shukla I. Controlled trial of hypo-osmalar versus World Health Organization oral rehydration solution. Indian Pediatrics 2000;37:952-60. Not an exercise intervention study
Ref ID: 4661

(248) Alarcon OM, Guerrero Y, de Fernandez MR, D'Jesus I, Burguera M, Burguera JL, Di Bernardo ML. Effect of copper supplementation on blood pressure values in patients with stable moderate hypertension. Archivos Latinoamericanos de Nutricion 2003;53(3):271-6. Case-Control / Case Study
Ref ID: 5117

(249) Alayón AN, Castro-Orozco R, Gaviria-Esquivia L, Fernández-Franco M, Benítez-Peña L. Factores de riesgo cardiovascular en escolares entre 7 y 14 años en Cartagena, Colombia, 2009^ies
Cardiovascular risk factors among 7-and 14-year old schoolchildren in Cartagena, Colombia, 2009^ien. Rev salud pública 2011 April;13(2):196-206. Cross-sectional study
Ref ID: 3927

(250) Albano F, Lo VA, Guarino A. The applicability and efficacy of guidelines for the management of acute gastroenteritis in outpatient children: a field-randomized trial on primary care pediatricians. The Journal of pediatrics 2010;156:226-30. Not an exercise intervention study
Ref ID: 4662

(251) Albertson AM, Thompson D, Franko DL, Kleinman RE, Barton BA, Crockett SJ. Consumption of breakfast cereal is associated with positive health outcomes: evidence from the National Heart, Lung, and Blood Institute Growth and Health Study. Nutrition Research 2008;28(11):744-52. Diet Intervention Study
Ref ID: 5118

(252) Albertson AM, Thompson D, Franko DL, Holschuh NM, Bauserman R, Barton BA. Prospective associations among cereal intake in childhood and adiposity, lipid levels, and physical activity during late adolescence. Journal of the American Dietetic Association 2009 October;109(10):1775-80. Prospective Study
Ref ID: 671

(253) Alcântara Neto ODd, Silva RdCR, Assis AMO, Pinto EdJ. Fatores associados à dislipidemia em crianças e adolescentes de escolas públicas de Salvador, Bahia^ipt
Factors associated with dyslipidemia in children and adolescents enrolled in public schools of Salvador, Bahia^ien. Rev bras epidemiol 2012 June;15(2):335-45. Cross-sectional study
Ref ID: 3928

(254) Alemu T, Lindtjorn B. Physical activity, illness and nutritional status among adults in a rural Ethiopian community. International Journal of Epidemiology 1995 October;24(5):977-83. Study limited to adults
Ref ID: 2193

(255) Alencar FH, Yuyama LKO, Varejão MdJ, Marinho HA. Determinantes e conseqüências da insegurança alimentar no Amazonas: a influência dos ecossistemas^ipt. Acta amaz 2007;37(3):413-8. Review article
Ref ID: 574

(256) Alexander D. Prevention of mental retardation: Four decades of research <FNR></FNR><FN>This article is a US Government work and, as such, is in the public domain in the United States of America. </FN>. Mental Retardation & Developmental Disabilities Research Reviews 1998 February;4(1):50-8. Review article
Ref ID: 3504

(257) Alexander ND, Cousens SN, Yahaya H, Abiose A, Jones BR. Ivermectin dose assessment without weighing scales. Bulletin of the World Health Organization 1993;71:361-6. Drug intervention study
Ref ID: 1039

(258) Alexeeva N, Sames C, Jacobs PL, Hobday L, Distasio MM, Mitchell SA, Calancie B. Comparison of training methods to improve walking in persons with chronic spinal cord injury: a randomized clinical trial. Journal of Spinal Cord Medicine 2011;34(4):362-79. Study limited to adults
Ref ID: 1023

(259) Alfie ME, Treem WR. Nonalcoholic fatty liver disease. Pediatric Annals 2006;35(4):290-+. Review article
Ref ID: 5119

(260) Alhassan S, Sirard JR, Robinson TN. The effects of increasing outdoor play time on physical activity in Latino preschool children. International Journal of Pediatric Obesity 2007;2(3):153-8. Not All Participants were Overweight and/or Obese
Ref ID: 1135

(261) Ali A, Williams C. Carbohydrate ingestion and soccer skill performance during prolonged intermittent exercise. Journal of Sports Sciences 2009 December;27(14):1499-508. Diet Intervention or Supplement Study
Ref ID: 612

(262) Alkandari JR, Maughan RJ, Roky R, Aziz AR, Karli U. The implications of Ramadan fasting for human health and well-being. Journal of Sports Sciences 2012;30:S9-S19. Diet Intervention Study
Ref ID: 5120

(263) Allan JD. New directions for the study of overweight. Western Journal of Nursing Research 20(1):7-13, 1998 Feb 1998;(1):7-13. Review article
Ref ID: 2874

(264) Allan M, Richardson GM, Jones-Otazo H. Probability density functions describing 24-hour inhalation rates for use in human health risk assessments: An update and comparison. Human and Ecological Risk Assessment 2008;14(2):372-91. Not an exercise intervention study
Ref ID: 5121

(265) Allor KM, Pivarnik JM, Sam LJ, Perkins CD. Treadmill economy in girls and women matched for height and weight. Journal of Applied Physiology 2000 August;89(2):512-6. Acute study
Ref ID: 1966

(266) Almagiá Flores AA, Rodríguez Rodríguez F, Barrraza Gómez FO, Lizana Arce PJ, Jorquera Aguilera CA. Perfil antropométrico de jugadoras chilenas de fútbol femenino^ies. Int J Morphol 2008 December;26(4):817-21. Cross-sectional study
Ref ID: 3929

(267) Almagià Flores AA, Rodríguez Rodríguez F, Barrraza Gómez FO, Lizana Arce PJ, Ivanovic Marincovich D, Binvignat Gutiérrez O. Perfil antropométrico de jugadores profesionales de voleibol sudamericano^ies. Int J Morphol 2009 March;27(1):53-7. Not an exercise intervention study
Ref ID: 3930

(268) Almeida A, Roveda G, Valin MR, Almeida NCd, Sartor V, Alves SM. Complicações da técnica de fixação tibial com parafuso e arruela para a reconstrução ligamentar do joelho^ipt
Complications of the screw/washer tibial fixation technique for knee ligament reconstruction^ien. Rev bras ortop 2010;45(5):409-14. Not an exercise intervention study
Ref ID: 3931

(269) Almeida Ed, Gonçalves A, El-Khatib S, Padovani CR. Lesão muscular após diferentes métodos de treinamento de musculação^ipt. Fisioter mov 2006 December;19(4):17-23. Acute study
Ref ID: 599

(270) Almeida FAd, Konigsfeld HP, Machado LMdO, Canadas AF, Issa EYO, Giordano RH, Cadaval RAdM. Avaliação de influências sociais e econômicas sobre a pressão arterial de adolescentes de escolas públicas e privadas: um estudo epidemiológico^ipt
Assessment of social and economic influences on blood pressure of adolescents in public and private schools: an epidemiological study^ien. J bras nefrol 2011 June;33(2):142-9. Cross-sectional study
Ref ID: 3932

(271) Almeida GPL, Carneiro KKA, Morais HCRd, Oliveira JBBd. Influência do alongamento dos músculos isquiostibial e retofemoral no pico de torque e potência máxima de joelho^ipt. Fisioter pesqui 2009 December;16(4):346-51. No aerobic exercise or WT intervention
Ref ID: 3933

(272) Almeida MFBd, Guinsburg R, Costa JOd, Anchieta LM, Freire LMS. Material and human resources for neonatal resuscitation in public maternity hospitals in Brazilian state capitals^ien
Recursos materiais e humanos para a reanimação neonatal nas maternidades públicas das capitais brasileiras^ipt. São Paulo med j 2008 May;126(3):156-60. Cross-sectional study
Ref ID: 3934

(273) Almeida TAd, Soares EA. Nutritional and anthropometric profile of adolescent volleyball athletes. Rev bras med esporte 2003 August;9(4):198-203. Cross-sectional study
Ref ID: 3935

(274) Almuzaini KS. Muscle function in Saudi children and adolescents: relationship to anthropometric characteristics during growth. Pediatric Exercise Science 2007 August;19(3):319-33. Cross-sectional study
Ref ID: 1141

(275) Alricsson M, Landstad BJ, Romild U, Gundersen KT. Physical activity, health, BMI and body complaints in high school students. Minerva Pediatrica 2008 February;60(1):19-25. Survey or questionnaire
Ref ID: 999

(276) Als H, Lawhon G, Duffy FH, McAnulty GB, Gibes GR, Blickman JG. Individualized developmental care for the very low-birth-weight preterm infant. Medical and neurofunctional effects. JAMA : the journal of the American Medical Association 1994;272:853-8. Subjects less than 2 years old
Ref ID: 4663

(277) Althuizen E, van Poppel MN, Seidell JC, van der Wijden C, van MW. Design of the New Life(style) study: a randomised controlled trial to optimise maternal weight development during pregnancy. [ISRCTN85313483]. BMC Public Health 2006;6:168. Study limited to adults
Ref ID: 1365

(278) Alvarado BE, Zunzunegui MV, Beland F, Bamvita JM. Life course social and health conditions linked to frailty in Latin American older men and women. Journals of Gerontology Series A-Biological Sciences & Medical Sciences 2008 December;63(12):1399-406. Cross-sectional study
Ref ID: 835

(279) Alvarado R, López Moreno JM. Hamartoma hipotalámico, una causa de pubertad precoz: caso clínico. Rev méd Chile 2001 October;129(10):1179-82. Case-Control / Case Study
Ref ID: 760

(280) Alvarez-Jimenez M, Gonzalez-Blanch C, Vazquez-Barquero JL, Perez-Iglesias R, Martinez-Garcia O, Perez-Pardal T, Ramirez-Bonilla ML, Crespo-Facorro B. Attenuation of antipsychotic-induced weight gain with early behavioral intervention in drug-naive first-episode psychosis patients: A randomized controlled trial. J Clin Psychiatry 2006 August;67(8):1253-60. Study limited to adults
Ref ID: 269

(281) Alvarez M, Sedano S, Cuadrado G, Redondo JC. Effects of an 18-week strength training program on low-handicap golfers' performance. Journal of Strength & Conditioning Research 2012 April;26(4):1110-21. Study limited to adults
Ref ID: 2335

(282) Alves C, Lima RVB. Impacto da atividade física e esportes sobre o crescimento e puberdade de crianças e adolescentes: [revisão]^ipt
Linear growth and puberty in children and adolescents: effects of physical activity and sports: [revision]^ien. Rev paul pediatr 2008 December;26(4):383-91. Review article
Ref ID: 3936

(283) Alves C, Oliveira AC, Brites C. Lipodystrophic syndrome in children and adolescents infected with the human immunodeficiency virus^ien. Braz j infect dis 2008 August;12(4):342-8. Review article
Ref ID: 3937

(284) Alves JG, Gale CR, Souza E, Batty GD. [Effect of physical exercise on bodyweight in overweight children: a randomized controlled trial in a Brazilian slum]. Cad Saude Publica 2008;24 Suppl 2:S353-9.:S353-S359. Primary outcome(s) not assessed
Ref ID: 157

(285) Alves JGB, Siqueira PP, Figueiroa JN. Excesso de peso e inatividade física em crianças moradoras de favelas na região metropolitana do Recife, PE^ipt
Overweight and physical inactivity in children living in favelas in the metropolitan region of Recife, Brazil^ien. J pediatr (Rio J ) 2009 February;85(1):67-71. Cross-sectional study
Ref ID: 3939

(286) Alves SS, Silva SRCd, Ribeiro RS, Vertematti AS, Fisberg M. Avaliaçäo de atividade física, estado nutricional e condiçäo social em adolescentes. Folha méd 2000 March;119(1):26-33. Survey or questionnaire
Ref ID: 3940

(287) Aman J, Skinner TC, de Beaufort CE, Swift PG, Aanstoot HJ, Cameron F, Hvidoere Study Group on Childhood Diabetes. Associations between physical activity, sedentary behavior, and glycemic control in a large cohort of adolescents with type 1 diabetes: the Hvidoere Study Group on Childhood Diabetes. Pediatr Diabetes 2009 June;10(4):234-9. Cross-sectional study
Ref ID: 740

(288) Aman MG, McDougle CJ, Scahill L, Handen B, Arnold LE, Johnson C, Stigler KA, Bearss K, Butter E, Swiezy NB, Sukhodolsky DD, Ramadan Y, Pozdol SL, Nikolov R, Lecavalier L, Kohn AE, Koenig K, Hollway JA, Korzekwa P, Gavaletz A, Mulick JA, Hall KL, Dziura J, Ritz L, Trollinger S et al. Medication and parent training in children with pervasive developmental disorders and serious behavior problems: results from a randomized clinical trial. Journal of the American Academy of Child and Adolescent Psychiatry 2009;48:1143-54. Drug intervention study
Ref ID: 4664

(289) Amaro S, Viggiano A, Di CA, Madeo I, Viggiano A, Baccari ME, Marchitelli E, Raia M, Viggiano E, Deepak S, Monda M, De LB. Kaledo, a new educational board-game, gives nutritional rudiments and encourages healthy eating in children: a pilot cluster randomized trial. Eur J Pediatr 2006 September;165(9):630-5. Not an exercise intervention study
Ref ID: 277

(290) Amaya RA, Kozinetz CA, McMeans A, Schwarzwald H, Kline MW. Lipodystrophy syndrome in human immunodeficiency virus-infected children. Pediatric Infectious Disease Journal 2002;21(5):405-10. Cross-sectional study
Ref ID: 5122

(291) Ambalavanan N, Carlo WA, Bobashev G, Mathias E, Liu B, Poole K, Fanaroff AA, Stoll BJ, Ehrenkranz R, Wright LL. Prediction of death for extremely low birth weight neonates. Pediatrics 2005;116(6):1367-73. Subjects less than 2 years old
Ref ID: 5123

(292) Ambalavanan N, Tyson JE, Kennedy KA, Hansen NI, Vohr BR, Wright LL, Carlo WA, -National-Institute-of-Child-Health-and-Human-Development-Neonatal-Research-Network. Vitamin A supplementation for extremely low birth weight infants: outcome at 18 to 22 months. Pediatrics 2005;115:e249-e254. Subjects less than 2 years old
Ref ID: 4666

(293) Ambler C, Eliakim A, Brasel JA, Lee WN, Burke G, Cooper DM. Fitness and the effect of exercise training on the dietary intake of healthy adolescents. International Journal of Obesity & Related Metabolic Disorders: Journal of the International Association for the Study of Obesity 1998 April;22(4):354-62. Cross-sectional study, Not All Participants were Overweight and/or Obese
Ref ID: 2094

(294) Amenta M, Cascio MT, Di FP, Venturini I. Diet and chronic constipation. Benefits of oral supplementation with symbiotic zir fos (Bifidobacterium longum W11 + FOS Actilight). Acta Bio-Medica de l Ateneo Parmense 2006 December;77(3):157-62. Drug intervention study
Ref ID: 1303

(295) American Dietetic Association (. Position of the American Dietetic Association: individual-, family-, school-, and community-based interventions for pediatric overweight. Journal of the American Dietetic Association 2006 June;106(6):925-45. Review article
Ref ID: 1381

(296) Amigo C, Bustos Muñoz P, Radrigán Kiguel ME, Ureta H. Estado nutricional en escolares de nivel socioeconómico opuesto. Rev méd Chile 1995 September;123(9):1063-70. Cross-sectional study
Ref ID: 3941

(297) Amigo H, Bustos P, Erazo M, Cumsille P, Silva C. Factores determinantes del exceso de peso en escolares: Un estudio multinivel^ies. Rev méd Chile 2007 December;135(12):1510-8. Cross-sectional study
Ref ID: 3942

(298) Amigo H, Erazo M, Bustos P, Aguilar C, Taibo M. Vigilancia nutricional en escolares chilenos: validez de la información^ies. Rev méd Chile 2008 August;136(8):989-95. Cross-sectional study
Ref ID: 3943

(299) Amorim AR, Linne YM, Lourenco PM. Diet or exercise, or both, for weight reduction in women after childbirth. [Review] [68 refs]. Cochrane Database of Systematic Reviews (3):CD005627, 2007 2007;(3):CD005627. Review article
Ref ID: 1199

(300) Amorim MMRd, Melo ASdO, Cardoso MAA, Assunção PLd. Atividade física durante a gravidez: revisão e recomendações^ipt. Femina 2007 August;35(8):521-7. Review article
Ref ID: 3944

(301) Amorim PG, Mendes TdB, Oliveira LSPd, Guerra-Júnior G, Ribeiro JD. Hormônio de crescimento em crianças e adolescentes com fibrose cística^ipt
Growth hormone in children and adolescents with cystic fibrosis^ien. Arq bras endocrinol metab 2011 December;55(9):671-6. Review article
Ref ID: 3945

(302) Amos A, Currie C, Hunt SM, Martin CJ. Health-related behaviour in a small Scottish community. Public Health 1990 March;104(2):131-40. Survey or questionnaire
Ref ID: 2310

(303) Ampuero S, Bee G. The potential to detect boar tainted carcasses by using an electronic nose based on mass spectrometry. Acta Veterinaria Scandinavica 2006 January 2;48:1-2. Animal study
Ref ID: 3505

(304) An JY, Hayman LL, Park YS, Dusaj TK, Ayres CG. Web-based weight management programs for children and adolescents: a systematic review of randomized controlled trial studies. [Review] [50 refs]. Advances in Nursing Science 32(3):222-40, 2009 Jul-Sep 2009;(3):222-40. Review article
Ref ID: 2875

(305) An P, Perusse L, Rankinen T, Borecki IB, Gagnon J, Leon AS, Skinner JS, Wilmore JH, Bouchard C, Rao DC. Familial aggregation of exercise heart rate and blood pressure in response to 20 weeks of endurance training: the HERITAGE family study. International Journal of Sports Medicine 2003 January;24(1):57-62. Study limited to adults
Ref ID: 1774

(306) An P, Borecki IB, Rankinen T, Despres JP, Leon AS, Skinner JS, Wilmore JH, Bouchard C, Rao DC. Evidence of major genes for plasma HDL, LDL cholesterol and triglyceride levels at baseline and in response to 20 weeks of endurance training: the HERITAGE Family Study. International Journal of Sports Medicine 2005 July;26(6):414-9. Study limited to adults
Ref ID: 1492

(307) An P, Teran-Garcia M, Rice T, Rankinen T, Weisnagel SJ, Bergman RN, Boston RC, Mandel S, Stefanovski D, Leon AS, Skinner JS, Rao DC, Bouchard C, HERITAGE Family. Genome-wide linkage scans for prediabetes phenotypes in response to 20 weeks of endurance exercise training in non-diabetic whites and blacks: the HERITAGE Family Study. Diabetologia 2005 June;48(6):1142-9. Study limited to adults
Ref ID: 1504

(308) Anagnostis P. Metabolic syndrome in the Mediterranean region: Current status. Indian Journal of Endocrinology & Metabolism 2012 January;16(1):72-80. Review article
Ref ID: 3506

(309) Anand SS, Davis AD, Ahmed R, Jacobs R, Xie C, Hill A, Sowden J, Atkinson S, Blimkie C, Brouwers M, Morrison K, de KL, Gerstein H, Yusuf S. A family-based intervention to promote healthy lifestyles in an aboriginal community in Canada. Can J Public Health 2007 November;98(6):447-52. No exercise only group
Ref ID: 147

(310) Andersen JR. Sorting criteria. Methods for on-line/at-line sorting of entire male carcasses with emphasis on the Danish method based on skatole content. Acta Veterinaria Scandinavica 2006 January 2;48:S14-3. Review article
Ref ID: 3507

(311) Andersen LB. Tracking of risk factors for coronary heart disease from adolescence to young adulthood with special emphasis on physical activity and fitness. A longitudinal study. Danish medical bulletin 1996;43:407-18. Cohort Study
Ref ID: 988

(312) Andersen LB, Sardinha LB, Froberg K, Riddoch CJ, Page AS, Anderssen SA. Fitness, fatness and clustering of cardiovascular risk factors in children from Denmark, Estonia and Portugal: the European Youth Heart Study. International Journal of Pediatric Obesity 2008;3:Suppl-66. Cross-sectional study
Ref ID: 997

(313) Anderson JW, Greenway FL, Fujioka K, Gadde KM, McKenney J, O'Neil PM. Bupropion SR enhances weight loss: a 48-week double-blind, placebo- controlled trial. Obes Res 2002 July;10(7):633-41. Study limited to adults
Ref ID: 377

(314) Anderson ML, Foster C, McGuigan MR, Seebach E, Porcari JP. Training vs. body image: does training improve subjective appearance ratings? Journal of Strength & Conditioning Research 2004 May;18(2):255-9. Study limited to adults
Ref ID: 1625

(315) Anderson NA, Raafat A, Shwe KH, Barbara J, Contreras M, Fraser ID, Gunson HH, Martlew V, Mijovic V, Goldie DJ. U.K. multicentre study on blood donors for surrogate markers of non-A non-B hepatitis. Part I: Alanine transferase and anti-HBc testing. Transfusion Medicine 1992 December;2(4):301-10. Not an exercise intervention study
Ref ID: 2274

(316) Anderson SE, Bandini LG, Dietz WH, Must A. Relationship between temperament, nonresting energy expenditure, body composition, and physical activity in girls. International Journal of Obesity 2004;28(2):300-6. Survey or questionnaire
Ref ID: 5124

(317) Anderson SE, Bandini LG, Must A. Child temperament does not predict adolescent body composition in girls. International Journal of Obesity 2005;29(1):47-53. Cross-sectional study
Ref ID: 5125

(318) Ando J, Nonaka K, Ozaki K, Sato N, Fujisawa KK, Suzuki K, Yamagata S, Takahashi Y, Nakajima R, Kato N, Ooki S. The Tokyo Twin Cohort Project: Overview and initial findings. Twin Research and Human Genetics 2006;9(6):817-26. Cohort Study
Ref ID: 5126

(319) Andrade FBd, Caldas Junior AdF, Kitoko PM, Batista JEM, Andrade TBd. Prevalence of overweight and obesity in elderly people from Vitória-ES, Brazil^ien
Prevalência de sobrepeso e obesidade em idosos da cidade de Vitória-ES, Brasil^ipt. Ciênc saúde coletiva 2012 March;17(3):749-56. Cross-sectional study, Study limited to adults
Ref ID: 3946

(320) Andrade JC, Andrade VS, Buffolo E, Greco OT, Lopes MG, Macedo Júnior A, Menezes Júnior AdS, Moraes AV, Mota NJM, Pachón JC, Schaldach M, Tebexreni AS, Tomas AA. Avaliaçäo do sensor de contratilidade cardíaca em sistema DDDR: estudo multicêntrico. Rev bras cir cardiovasc 1998 December;13(4):340-50. Study limited to adults
Ref ID: 3947

(321) Andrade KC, Souza SB, Szarfarc SC. Desenvolvimento neuromotor e dentição de crianças atendidas em serviços públicos de saúde do Brasil, no primeiro ano de vida^ipt. Rev bras crescimento desenvolv hum 2007 June;17(2):37-44. Cross-sectional study
Ref ID: 3948

(322) Andreasi V, Michelin E, Rinaldi AE, Burini RC. Aptidão física associada às medidas antropométricas de escolares do ensino fundamental^ipt
Physical fitness and associations with anthropometric measurements in 7 to 15-year-old school children^ien. J pediatr (Rio J ) 2010 December;86(6):497-502. Cross-sectional study
Ref ID: 3949

(323) Andreou E, Philippou C, Papandreou D. Effects of an intervention and maintenance weight loss diet with and without exercise on anthropometric indices in overweight and obese healthy women. Annals of Nutrition & Metabolism 2011;59(2-4):187-92. Study limited to adults
Ref ID: 2336

(324) Andresen Ãy. Boar taint related compounds: Androstenone/skatole/other substances. Acta Veterinaria Scandinavica 2006 January 2;48:S5-4. Animal study
Ref ID: 3508

(325) Andriolo RB, El Dib RP, Ramos L, Atallah AN, da Silva EM. Aerobic exercise training programmes for improving physical and psychosocial health in adults with Down syndrome. [Review] [138 refs][Update of Cochrane Database Syst Rev. 2009;(3):CD005176; PMID: 19588368]. Cochrane Database of Systematic Reviews (5):CD005176, 2010 2010;(5):CD005176. Review article
Ref ID: 535

(326) Angeles-Agdeppa I, Lana RD, Barba CV. A case study on dual forms of malnutrition among selected households in District 1, Tondo, Manila. Asia Pacific Journal of Clinical Nutrition 2003;12(4):438-46. Survey or questionnaire
Ref ID: 1686

(327) Angelini C, Pegoraro E, Turella E, Intino MT, Pini A, Costa C. Deflazacort in Duchenne dystrophy: study of long-term effect.[Erratum appears in Muscle Nerve 1994 Jul;17(7):833]. Muscle & Nerve 1994 April;17(4):386-91. Drug intervention study
Ref ID: 1085

(328) Angelini C, Semplicini C, Ravaglia S, Bembi B, Servidei S, Pegoraro E, Moggio M, Filosto M, Sette E, Crescimanno G, Tonin P, Parini R, Morandi L, Marrosu G, Greco G, Musumeci O, Di Iorio G, Siciliano G, Donati MA, Carubbi F, Ermani M, Mongini T, Toscano A. Observational clinical study in juvenile-adult glycogenosis type 2 patients undergoing enzyme replacement therapy for up to 4 years. J Neurol 2012;259(5):952-8. Observational study
Ref ID: 3188

(329) Angelopoulos PD, Milionis HJ, Grammatikaki E, Moschonis G, Manios Y. Changes in BMI and blood pressure after a school based intervention: the CHILDREN study. European Journal of Public Health 2009;19:319-25. Not All Participants were Overweight and/or Obese
Ref ID: 4667

(330) Angulo BR, Burghardt AR, Lloyd M, Ulrich DA. Physical activity in infants with Down syndrome receiving a treadmill intervention. Infant behavior & development 2008;31:255-69. Subjects less than 2 years old
Ref ID: 4668

(331) AnnagÃ¼r BBa. Obezitede Ã‡eÅŸitli Risk FaktÃ¶rleri ve DÃ¼rtÃ¼sellik. (Turkish). Current Approaches in Psychiatry / Psikiyatride Guncel Yaklasimlar 2010 November;2(4):572-82. Review article
Ref ID: 3509

(332) Anomasiri W, Sanguanrungsirikul S, Saichandee P. Low dose creatine supplementation enhances sprint phase of 400 meters swimming performance. Journal of the Medical Association of Thailand 2004 September;87:Suppl-32. Diet Intervention or Supplement Study
Ref ID: 1557

(333) Antonella EDP, Luca S, Emilia DF, Rosaria PM, Annarita C, Giuseppe C, Franco C, Giuliana V, Adriana F, Salvatore DM, Armido R. Familial and Environmental-Influences on Body-Composition and Body-Fat Distribution in Childhood in Southern Italy. International Journal of Obesity 1994;18(9):596-601. Cross-sectional study
Ref ID: 5127

(334) Antonio J, Sanders MS, Ehler LA, Uelmen J, Raether JB, Stout JR. Effects of exercise training and amino-acid supplementation on body composition and physical performance in untrained women. Nutrition 2000 November;16(11-12):1043-6. Diet Intervention or Supplement Study
Ref ID: 1944

(335) Antonio J, Sanders MS, Van GD. The effects of bovine colostrum supplementation on body composition and exercise performance in active men and women. Nutrition 2001 March;17(3):243-7. Diet Intervention or Supplement Study
Ref ID: 1920

(336) Antoniou EE, Derom C, Thiery E, Fowler T, Southwood TR, Zeegers MP. The Influence of Genetic and Environmental Factors on the Etiology of the Human Umbilical Cord: The East Flanders Prospective Twin Survey. Biology of Reproduction 2011;85(1):137-43. Not an exercise intervention study
Ref ID: 5128

(337) Apetito L, Vasconcelos K, Marim MMF, Detregiachi CRP. Prática de dietas de emagrecimento por escolares adolescentes^ipt
Practical diets for weight loss teen^ien. J Health Sci Inst 2010 December;28(4):329-33. Survey or questionnaire
Ref ID: 3950

(338) Apostol G, Pakalnis A, Laforet GA, Robieson WZ, Olson E, Abi-Saab WM, Saltarelli M. Safety and tolerability of divalproex sodium extended-release in the prophylaxis of migraine headaches: results of an open-label extension trial in adolescents. Headache 2009;49:36-44. Drug intervention study
Ref ID: 4670

(339) Apostol G, Lewis DW, Laforet GA, Robieson WZ, Fugate JM, Abi-Saab WM, Saltarelli MD. Divalproex sodium extended-release for the prophylaxis of migraine headache in adolescents: results of a stand-alone, long-term open-label safety study. Headache 2009;49:45-53. Drug intervention study
Ref ID: 4669

(340) Apovian CM, Bergenstal RM, Cuddihy RM, Qu Y, Lenox S, Lewis MS, Glass LC. Effects of exenatide combined with lifestyle modification in patients with type 2 diabetes. Am J Med 2010 May;123(5):468-17. Study not limited to children and adolescents
Ref ID: 50

(341) Appel-Dingemanse S. Clinical Pharmacokinetics of Tegaserod, a Serotonin 5-HT[sub 4] Receptor Partial Agonist with Promotile Activity. Clinical Pharmacokinetics 2002 October;41(13):1021. Drug intervention study
Ref ID: 3510

(342) Aragona J, Cassady J, Drabman RS. Treating overweight children through parental training and contingency contracting. Journal of Applied Behavior Analysis 1975;8(3):269-78. Lifestyle Intervention
Ref ID: 2393

(343) Aranha MFM, Alves MC, BÃ©rzin F, GaviÃ£o MBD. Efficacy of electroacupuncture for myofascial pain in the upper trapezius muscle: a case series. Brazilian Journal of Physical Therapy / Revista Brasileira de Fisioterapia 2011 September;15(5):371-9. Study limited to adults
Ref ID: 3511

(344) Araújo CL, Dumith SC, Menezes AM, Hallal PC. Peso medido, peso percebido e fatores associados em adolescentes^ipt
Measured weight, self-perceived weight, and associated factors in adolescents^ien. Rev panam salud pública 2010 May;27(5):360-7. Cohort Study
Ref ID: 3951

(345) Araújo FL, Monteiro LZ, Pinheiro MHNP, Silva CABd. Prevalência de fatores de risco para hipertensão arterial em escolares do município de Fortaleza, CE^ipt
Prevalence of hypertension risk factors in students in the city of Fortaleza, Ceará, Brazil^ien. Rev bras hipertens 2010 December;17(4):203-9. Cross-sectional study
Ref ID: 3952

(346) Araújo MFM, Almeida LSd, Silva PCVd, Vasconcelos HCAd, Lopes MVdO, Damasceno MMC. Sobrepeso entre adolescentes de escolas particulares de Fortaleza, CE, Brasil^ipt
Overweight among adolescents from private schools in Fortaleza, CE, Brazil^ien
Sobrepeso entre adolescentes de escuelas privadas de Fortaleza, CE, Brasil^ies. Rev bras enferm 2010 August;63(4):623-8. Cross-sectional study
Ref ID: 3953

(347) Araújo VCd, Konrad LM, Rabacow FM, Graup S, Amboni R, Farias Júnior JCd. Prevalência de excesso de peso em adolescentes brasileiros: um estudo de revisão sistemática^ipt. Rev bras ativ fís saúde 2007;12(3). Review article
Ref ID: 3954

(348) Aránguiz A, García G, Rojas D, Salas B, Martínez R, Mac MK. Estudio descriptivo, comparativo y correlacional del estado nutricional y condición cardiorrespiratoria en estudiantes universitarios de Chile^ies
Descriptive, comparative and correlational study of nutritional and cardio-respiratory condition of Chilean universitary students^ien. Rev chil nutr 2010 March;37(1):70-8. Cross-sectional study
Ref ID: 3955

(349) Arbeit ML, Johnson CC, Mott DS, Harsha DW, Nicklas TA, Webber LS, Berenson GS. The Heart Smart cardiovascular school health promotion: behavior correlates of risk factor change. Preventive Medicine 1992 January;21(1):18-32. Lifestyle Intervention
Ref ID: 2292

(350) Arbesú Ruiz N, Ríos Fernández Adl. La pubertad y la capacidad de trabajo físico. Rev cuba pediatr 1989 June;61(3):382-92. Not an exercise intervention study
Ref ID: 3956

(351) Arboleda Naranjo LH. Beneficios del Ejercicio^ies. Hacia promoc salud 2003 November;(8):77-84. Review article
Ref ID: 3957

(352) Arciero PJ, Bougopoulos CL, Nindl BC, Benowitz NL. Influence of age on the thermic response to caffeine in women. Metabolism: Clinical & Experimental 2000 January;49(1):101-7. Study limited to adults
Ref ID: 1993

(353) Arciero PJ, Hannibal NS, III, Nindl BC, Gentile CL, Hamed J, Vukovich MD. Comparison of creatine ingestion and resistance training on energy expenditure and limb blood flow. Metabolism: Clinical & Experimental 2001 December;50(12):1429-34. Study limited to adults
Ref ID: 1872

(354) Arciero PJ, Ormsbee MJ. Relationship of blood pressure, behavioral mood state, and physical activity following caffeine ingestion in younger and older women. Applied Physiology, Nutrition, & Metabolism = Physiologie Appliquee, Nutrition et Metabolisme 2009 August;34(4):754-62. Study limited to adults
Ref ID: 674

(355) Arcos G, Uarac U, Molina V, Repossi F, Ulloa V. Impacto de la violencia doméstica sobre la salud reproductiva y neonatal. Rev méd Chile 2001 December;129(12):1413-24. Cohort Study
Ref ID: 3958

(356) Ardoy DN, Fernandez-Rodriguez JM, Chillon P, Artero EG, Espana-Romero V, Jimenez-Pavon D, Ruiz JR, Guirado-Escamez C, Castillo MJ, Ortega FB. [Physical fitness enhancement through education, EDUFIT study: background, design, methodology and dropout analysis]. [Spanish]. Revista Espanola de Salud Publica 2010 March;84(2):151-68. Not All Participants were Overweight and/or Obese
Ref ID: 507

(357) Arens U, Barasi M, Belton L, Burley V, Bussell G, Hood S, McLean L, Watling R, Gatenby S. Current literature. Journal of Human Nutrition & Dietetics 16[3], 201-213. 2003. Abstract,
Ref ID: 3512

(358) Arias JL. Free-throw accuracy and success as a function of ball weight in 9- to 11-year-old male players^ien
Precisão e êxito em função do peso da bola em jogadores de 9 a 11 anos de idade^ipt. Motriz rev educ fís (Impr ) 2012 June;18(2):338-44. Not an exercise intervention study
Ref ID: 3959

(359) Arif MA, Arif K. Low birthweight babies in the Third World: maternal nursing versus professional nursing care. J Trop Pediatr 1999;45:278-80. Not an exercise intervention study
Ref ID: 886

(360) Arikawa AY, O'Dougherty M, Kaufman BC, Smith AJ, Thomas W, Warren M, Kurzer MS, Schmitz KH. Women in Steady Exercise Research (WISER): study design and methods. Contemporary Clinical Trials 2010 September;31(5):457-65. Study limited to adults
Ref ID: 2395

(361) Arikawa AY, Kurzer MS, Thomas W, Schmitz KH. No effect of exercise on insulin-like growth factor-I, insulin, and glucose in young women participating in a 16-week randomized controlled trial. Cancer Epidemiology, Biomarkers & Prevention 2010 November;19(11):2987-90. Study limited to adults
Ref ID: 2394

(362) Arikawa AY, Thomas W, Schmitz KH, Kurzer MS. Sixteen weeks of exercise reduces C-reactive protein levels in young women. Medicine & Science in Sports & Exercise 2011 June;43(6):1002-9. Study limited to adults
Ref ID: 2396

(363) Ariza AJ, Laslo KM, Thomson JS, Seshadri R, Binns HJ, Pediatric Practice Research Group. Promoting growth interpretation and lifestyle counseling in primary care. Journal of Pediatrics 2009 April;154(4):596-601. Not an exercise intervention study
Ref ID: 781

(364) Armelagos GJ. The Omnivore'S Dilemma the Evolution of the Brain and the Determinants of Food Choice. Journal of Anthropological Research 2010;66(2):161-86. Review article
Ref ID: 5129

(365) Armstrong LE, Whittlesey MJ, Casa DJ, Elliott TA, Kavouras SA, Keith NR, Maresh CM. No effect of 5% hypohydration on running economy of competitive runners at 23 degrees C. Medicine & Science in Sports & Exercise 2006 October;38(10):1762-9. Not a randomized controlled trial (RCT)
Ref ID: 1343

(366) Armstrong N, Welsman JR. Peak oxygen uptake in relation to growth and maturation in 11-to 17-year-old humans. European Journal of Applied Physiology 2001;85(6):546-51. Cohort Study
Ref ID: 5130

(367) Arnberg K, Larnkjaer A, Michaelsen KF, Molgaard C. Central adiposity and protein intake are associated with arterial stiffness in overweight children. Journal of Nutrition 2012 May;142(5):878-85. Cross-sectional study
Ref ID: 2397

(368) Arnett MG, Lutz B. Effects of rope-jump training on the os calcis stiffness index of postpubescent girls. Medicine & Science in Sports & Exercise 2002 December;34(12):1913-9. Not All Participants were Overweight and/or Obese
Ref ID: 1790

(369) Arnold LE, Amato A, Bozzolo H, Hollway J, Cook A, Ramadan Y, Crowl L, Zhang D, Thompson S, Testa G, Kliewer V, Wigal T, McBurnett K, Manos M. Acetyl-L-carnitine (ALC) in attention-deficit/hyperactivity disorder: a multi-site, placebo-controlled pilot trial. J Child Adolesc Psychopharmacol 2007;17:791-802. Diet Intervention or Supplement Study
Ref ID: 4671

(370) Arocha R. Lipoproteinas de alta densidad (HDL-C) y sus relaciones con el proceso aterosclerÃ³tico. (Spanish). Informe Medico 2002 March;4(3):151. Review article
Ref ID: 3513

(371) Arrebola E, Gomez-Candela C, Fernandez-Fernandez C, Loria V, Munoz-Perez E, Bermejo LM. Evaluation of a lifestyle modification program for treatment of overweight and nonmorbid obesity in primary healthcare and its influence on health-related quality of life. Nutrition in Clinical Practice 2011 June;26(3):316-21. Study limited to adults
Ref ID: 2398

(372) Arroll B, Beaglehole R. Does physical activity lower blood pressure: a critical review of the clinical trials. [Review] [33 refs]. Journal of Clinical Epidemiology 1992 May;45(5):439-47. Review article
Ref ID: 2289

(373) Arroyave LF, Ramirez AC, Velásquez C, Manrique Hernández RD. Factores de riesgo asociados a estrías atróficas en mujeres adolescentes de un colegio privado, Medellín, 2003^ies
Risk factors associated with strechmarks in teenage girls of a private school, Medellin, 2003^ien. CES med 2009 June;23(1,supl):81-6. Case-Control / Case Study
Ref ID: 3960

(374) Arruda ELMd, Lopes AdS. Gordura corporal, nível de atividade física e hábitos alimentares de adolescentes da região serrana da Santa Catarina, Brasil^ipt. Rev bras cineantropom desempenho hum 2007 March;9(1):5-11. Cross-sectional study
Ref ID: 625

(375) Arruda MF. Análise postural computadorizada de alterações musculoesqueléticas decorrentes do sobrepeso em escolares: [revisão]^ipt
Evaluation posture computerized in disturbance on musculoskeletal resulting from by overweight schoolchildren: [revision]^ien. Motriz rev educ fís (Impr ) 2009 March;15(1):143-50. Cross-sectional study
Ref ID: 3961

(376) Arsenault JE, Havel PJ, López-de RD, Penny ME, Van L, Brown KH. Longitudinal measures of circulating leptin and ghrelin concentrations are associated with the growth of young Peruvian children but are not affected by zinc supplementation. The American journal of clinical nutrition 2007;86:1111-9. Diet Intervention or Supplement Study
Ref ID: 4672

(377) Artero EG, Espana-Romero V, Ortega FB, Jimenez-Pavon D, Ruiz JR, Vicente-Rodriguez G, Bueno M, Marcos A, Gomez-Martinez S, Urzanqui A, Gonzalez-Gross M, Moreno LA, Gutierrez A, Castillo MJ. Health-related fitness in adolescents: underweight, and not only overweight, as an influencing factor. The AVENA study. Scandinavian Journal of Medicine & Science in Sports 2010 June;20(3):418-27. Cross-sectional study
Ref ID: 498

(378) Artero EG, Ruiz JR, Ortega FB, Espana-Romero V, Vicente-Rodriguez G, Molnar D, Gottrand F, Gonzalez-Gross M, Breidenassel C, Moreno LA, Gutierrez A, HELENA Study Group. Muscular and cardiorespiratory fitness are independently associated with metabolic risk in adolescents: the HELENA study. Pediatr Diabetes 2011 December;12(8):704-12. Cross-sectional study
Ref ID: 2399

(379) Artioli GG, Iglesias RT, Franchini E, Gualano B, Kashiwagura DB, Solis MY, Benatti FB, Fuchs M, Lancha Junior AH. Rapid weight loss followed by recovery time does not affect judo-related performance. Journal of Sports Sciences 2010 January;28(1):21-32. Not a randomized controlled trial (RCT)
Ref ID: 553

(380) Artz E, Freemark M. The pathogenesis of insulin resistance in children: Metabolic complications and the roles of diet, exercise and pharmacotherapy in the prevention of type 2 diabetes. Pediatr Endocrinol Rev 2004;1(3):296-309. Review article
Ref ID: 3189

(381) Aschemeier B, Lange K, Kordonouri O, Danne T. Paediatric obesity and type 2 diabetes: Strategies for prevention and treatment. Pract Diabetes Int 2008;25(9):368-75. Review article
Ref ID: 3190

(382) Ascher B, Goldberg DJ, Polla L, MÃ´le B, Kinney B, Gasperoni C, Salgarello M, Granado PC, Goldberg DJ, Gasparotti M, Sanchez M, Dierickx CC, Bitter P, Kunzi-Rapp K, Wortmann S, Gottlober P, Diebolder R, Rupp M. IMCAS - International Master Course on Ageing Skin. Paris, January 2001. Journal of Cutaneous Laser Therapy 2001 March;3(1):13-49. Abstract
Ref ID: 3514

(383) Asher MI, Douglas C, Airy M, Andrews D, Trenholme A. Effects of chest physical therapy on lung function in children recovering from acute severe asthma. Pediatr Pulmonol 1990;9:146-51. Rehabilitation study, Study less than 4 weeks
Ref ID: 1075

(384) Ashizawa K, Rahmawati NT, Hastuti J. Body size and shape, and its secular change in Javanese-Indonesian adults. Anthropological Science 2009;117(3):165-70. Cross-sectional study
Ref ID: 5131

(385) Ashton CH. Biomedical benefits of cannabinoids? Addiction Biology 1999 April;4(2):111-26. Review article
Ref ID: 3515

(386) Ashwal S, Wycliffe ND, Holshouser BA. Advanced Neuroimaging in Children with Nonaccidental Trauma. Developmental Neuroscience 2010;32(5-6):343-60. Review article
Ref ID: 5132

(387) Ashwood P, Kwong C, Hansen R, Hertz-Picciotto I, Croen L, Krakowiak P, Walker W, Pessah IN, Water J. Brief Report: Plasma Leptin Levels are Elevated in Autism: Association with Early Onset Phenotype? Journal of Autism & Developmental Disorders 2008 January;38(1):169-75. Not an exercise intervention study
Ref ID: 3801

(388) Ashworth A, Khanum S. Cost-effective treatment for severely malnourished children: what is the best approach? Health policy and planning 1997;12:115-21. Diet Intervention Study
Ref ID: 4673

(389) Ashworth A, Shrimpton R, Jamil K. Growth monitoring and promotion: review of evidence of impact. Maternal & Child Nutrition 2008 January 2;4:86-117. Review article
Ref ID: 3516

(390) Aspée A, Sepúlveda C, Amarales O, Acuña L, Olguín C, Rider L, Gross M, Vila C, Enriquez G. Importancia del manejo del ductus arterioso persistente en la XII Región al implementar el diagnóstico ecográfico y tratamiento quirúrgico a nivel local^ies. Rev chil cardiol 2009 December;28(4):369-74. Retrospective study
Ref ID: 3962

(391) Aspres N, Benítez A, Galindo A, Larguía M. Amamantamiento en recien nacidos prematuros de muy bajo peso al nacer (PMBPN, PN ó1500 grs.) : análisis de una experiencia en una institución pública. Rev Hosp Matern Infant Ramon Sarda 1994;13(3):115-22. Subjects less than 2 years old
Ref ID: 3963

(392) Asserhoj M, Nehammer S, Matthiessen J, Michaelsen KF, Lauritzen L. Maternal fish oil supplementation during lactation may adversely affect long-term blood pressure, energy intake, and physical activity of 7-year-old boys. J Nutr 2009 February;139(2):298-304. Study limited to adults
Ref ID: 142

(393) Assis CMd, Quio VR, Rasseli JG, Cunha FGCd, Salaroli LB. Hábitos alimentares e estado nutricional de jovens: um estudo comparativo^ipt
Eating habits and nutritional status of the youth: a comparative study^ien. Nutrire Rev Soc Bras Aliment Nutr 2009 April;34(1):13-27. Diet Intervention Study
Ref ID: 3964

(394) Astrom E, Jorulf H, Soderhall S. Intravenous pamidronate treatment of infants with severe osteogenesis imperfecta. Archives of Disease in Childhood 2007 April;92(4):332-8. Drug intervention study
Ref ID: 1258

(395) Atabek ME, Pirgon O. Use of metformin in obese adolescents with hyperinsulinemia: a 6-month, randomized, double-blind, placebo-controlled clinical trial. J Pediatr Endocrinol Metab 2008 April;21(4):339-48. No exercise only group, Drug intervention study
Ref ID: 178

(396) Atalah Samur E, Urteaga R, Rebolledo Acevedo A, Delfín C, Ramos H. Patrones alimentarios y de actividad física en escolares de la Región de Aysén. Rev chil pediatr 1999 December;70(6):483-90. Survey or questionnaire
Ref ID: 3965

(397) Athyros VG, Bouloukos VI, Pehlivanidis AN, Papageorgiou AA, Dionysopoulou SG, Symeonidis AN, Petridis DI, Kapousouzi MI, Satsoglou EA, Mikhailidis DP, MetS-Greece Collaborative Group. The prevalence of the metabolic syndrome in Greece: the MetS-Greece Multicentre Study. Diabetes, Obesity & Metabolism 2005 July;7(4):397-405. Cross-sectional study
Ref ID: 1506

(398) Atkin LM, Davies PSW. Diet composition and body composition in preschool children. American Journal of Clinical Nutrition 2000;72(1):15-21. Cross-sectional study
Ref ID: 5133

(399) Atkinson RL, Lee I, Shin HJ, He J. Human adenovirus-36 antibody status is associated with obesity in children. International Journal of Pediatric Obesity 2010;5(2):157-60. Cross-sectional study
Ref ID: 5134

(400) Atlantis E, Barnes EH, Singh MA. Efficacy of exercise for treating overweight in children and adolescents: a systematic review. [Review] [103 refs]. International Journal of Obesity 2006 July;30(7):1027-40. Review article
Ref ID: 1382

(401) Atlantis E, Salmon J, Bauman A. Acute effects of advertisements on children's choices, preferences, and ratings of liking for physical activities and sedentary behaviours: a randomised controlled pilot study. J Sci Med Sport 2008 November;11(6):553-7. Acute study, Primary outcome(s) not assessed
Ref ID: 213

(402) Atsumi T, Iwakura I, Kashiwagi Y, Fujisawa S, Ueha T. Free Radical Scavenging Activity in the Nonenzymatic Fraction of Human Saliva: A Simple DPPH Assay Showing the Effect of Physical Exercise. Antioxidants & Redox Signaling 1999;1(4):537-46. Not an exercise intervention study
Ref ID: 5135

(403) Attux C, Martini LC, Araujo CM, Roma AM, Reis AF, Bressan RA. The effectiveness of a non-pharmacological intervention for weight gain management in severe mental disorders: results from a national multicentric study. Revista Brasileira de Psiquiatria 2011 June;33(2):117-21. Lifestyle Intervention
Ref ID: 2400

(404) Aucouturier J, Isacco L, Thivel D, Fellmann N, Chardigny JM, Duclos M, Duche P. Effect of time interval between food intake and exercise on substrate oxidation during exercise in obese and lean children. Clinical Nutrition 2011 December;30(6):780-5. Not a randomized controlled trial (RCT)
Ref ID: 2401

(405) August GP, Caprio S, Fennoy I, Freemark M, Kaufman FR, Lustig RH, Silverstein JH, Speiser PW, Styne DM, Montori VM. Prevention and treatment of pediatric obesity: An Endocrine Society clinical practice guideline based on expert opinion. J Clin Endocrinol Metab 2008;93(12):4576-99. Review article
Ref ID: 3191

(406) Austin GP, Garrett GE, Tiberio D. Effect of added mass on human unipedal hopping. Perceptual and motor skills 2002;94:834-40. Study limited to adults
Ref ID: 4676

(407) Austin SB, Field AE, Wiecha J, Peterson KE, Gortmaker SL. The impact of a school-based obesity prevention trial on disordered weight-control behaviors in early adolescent girls. Arch Pediatr Adolesc Med 2005 March;159(3):225-30. No exercise only group
Ref ID: 313

(408) Austin SB, Kim J, Wiecha J, Troped PJ, Feldman HA, Peterson KE. School-based overweight preventive intervention lowers incidence of disordered weight-control behaviors in early adolescent girls. Arch Pediatr Adolesc Med 2007 September;161(9):865-9. No exercise only group
Ref ID: 219

(409) AuYeung W, Canales RA, Leckie JO. The fraction of total hand surface area involved in young children's outdoor hand-to-object contacts. Environmental Research 2008;108(3):294-9. Not an exercise intervention study
Ref ID: 5136

(410) Avdic D, Jusupovic F, Kudumovic M. Anthropometric values for boys aged 14-15 years who actively train basketball in comparing to boys of same age who do not train any sports. Healthmed 2008;2(4):253-64. Not an exercise intervention study
Ref ID: 5137

(411) Ayala GX, Elder JP, Campbell NR, Arredondo E, Baquero B, Crespo NC, Slymen DJ. Longitudinal intervention effects on parenting of the Aventuras para Ninos study. Am J Prev Med 2010 February;38(2):154-62. No exercise only group, Not an exercise intervention study
Ref ID: 63

(412) Ayoama R, Hiruma E, Sasaki H. Effects of creatine loading on muscular strength and endurance of female softball players. Journal of Sports Medicine & Physical Fitness 2003 December;43(4):481-7. Diet Intervention or Supplement Study
Ref ID: 1679

(413) Azadbakht L, Mirmiran P, Hedayati M, Esmaillzadeh A, Shiva N, Azizi F. Particle size of LDL is affected by the National Cholesterol Education Program (NCEP) step II diet in dyslipidaemic adolescents. BR J NUTR 2007 July;98(1):134-9. Case-Control / Case Study, Diet Intervention Study
Ref ID: 1213

(414) Azevedo BAR, Ribeiro SML. Avaliação do estado nutricional e do balanço energético de um grupo de atletas de ginástica artística^ipt. Motriz rev educ fís (Impr ) 2007;13(3):165-73. Cross-sectional study
Ref ID: 3967

(415) Azizi F, Bahrainian M, Khamseh ME, Khoshniat M. Intellectual development and thyroid function in children who were breast-fed by thyrotoxic mothers taking methimazole. Journal of Pediatric Endocrinology & Metabolism 2003;16(9):1239-43. Drug intervention study
Ref ID: 5138

(416) Aznar S, Naylor PJ, Silva P, Perez M, Angulo T, Laguna M, Lara MT, Lopez-Chicharro J. Patterns of physical activity in Spanish children: a descriptive pilot study. Child: Care, Health & Development 2011 May;37(3):322-8. Cross-sectional study
Ref ID: 2402

(417) Ã‡elik GGl, TahiroÄŸlu Al, AvcÄ± A. Ã‡ocuk ve Ergenlerde Atipik Antipsikotik Ä°laÃ§larÄ±n Metabolik ve Endokrin Yan Etkileri. (Turkish). Current Approaches in Psychiatry / Psikiyatride Guncel Yaklasimlar 2011 June;3(2):232-50. Review article
Ref ID: 3517

(418) Babamoto KS, Sey KA, Camilleri AJ, Karlan VJ, Catalasan J, Morisky DE. Improving diabetes care and health measures among hispanics using community health workers: results from a randomized controlled trial. Health Educ Behav 2009 February;36(1):113-26. Study not limited to children and adolescents
Ref ID: 137

(419) Baby S, Nguyen M, Tran D, Raffa RB. Substance P antagonists: the next breakthrough in treating depression?: REVIEW ARTICLE. Journal of Clinical Pharmacy & Therapeutics 1999 December;24(6):461. Review article
Ref ID: 526

(420) Bacha JM, Appugliese D, Coleman S, Kaciroti N, Bradley RH, Corwyn RF, Lumeng JC. Maternal perception of neighborhood safety as a predictor of child weight status: The moderating effect of gender and assessment of potential mediators. International Journal of Pediatric Obesity 2010;5(1):72-9. Survey or questionnaire
Ref ID: 556

(421) Bacharach DW, von Duvillard SP, Rundell KW, Meng J, Cring MR, Szmedra L, Castle JM. Carbohydrate drinks and cycling performance. Journal of Sports Medicine & Physical Fitness 1994 June;34(2):161-8. Not a randomized controlled trial (RCT)
Ref ID: 2233

(422) Baciuk EP, Pereira RI, Cecatti JG, Braga AF, Cavalcante SR. Water aerobics in pregnancy: cardiovascular response, labor and neonatal outcomes. Reproductive Health 2008;5:10. Study limited to adults
Ref ID: 4678

(423) Badesch DB, Raskob GE, Elliott CG, Krichman AM, Farber HW, Frost AE, Barst RJ, Benza RL, Liou TG, Turner M, Giles S, Feldkircher K, Miller DP, McGoon MD. Pulmonary arterial hypertension: baseline characteristics from the REVEAL Registry. Chest 2010 February;137(2):376-87. Study limited to adults
Ref ID: 589

(424) Badland HM, Schofield GM, Witten K, Schluter PJ, Mavoa S, Kearns RA, Hinckson EA, Oliver M, Kaiwai H, Jensen VG, Ergler C, McGrath L, McPhee J. Understanding the Relationship between Activity and Neighbourhoods (URBAN) Study: research design and methodology. BMC Public Health 2009;9:224. Description of study from review or magazine or etc. (not the actual study)
Ref ID: 705

(425) Baek HS, Kim YD, Shin JH, Kim JH, Oh JW, Lee HB. Serum leptin and adiponectin levels correlate with exercise-induced bronchoconstriction in children with asthma. Annals of Allergy Asthma & Immunology 2011;107(1):14-21. Cross-sectional study
Ref ID: 5139

(426) Baena Diez JM, Atance Yague RM, Escriba Jordana JM, Conesa GA, Rivera CD, Flores Cebria RM. [Perception of medical advice: in all cases and to all patients equally?]. [Spanish]. Gaceta Sanitaria 1999 January;13(1):46-52. Cross-sectional study
Ref ID: 2031

(427) Baggett CD, Stevens J, Catellier DJ, Evenson KR, McMurray RG, He K, Treuth MS. Compensation or displacement of physical activity in middle-school girls: the Trial of Activity for Adolescent Girls. International Journal of Obesity 2010 July;34(7):1193-9. Study less than 4 weeks
Ref ID: 494

(428) Bahl R, Bhandari N, Taneja S, Bhan MK. The impact of vitamin A supplementation on physical growth of children is dependent on season. European Journal of Clinical Nutrition 1997;51:26-9. Diet Intervention or Supplement Study
Ref ID: 4679

(429) Bailey N. Current choices in omega 3 supplementation. Nutrition Bulletin 2009 March;34(1):85-91. Review article
Ref ID: 3518

(430) Baillie-Hamilton PF. Chemical toxins: A hypothesis to explain the global obesity epidemic. Journal of Alternative and Complementary Medicine 2002;8(2):185-92. Review article
Ref ID: 5140

(431) Baker KG, Robertson VJ, Duck FA. A Review of Therapeutic Ultrasound: Biophysical Effects. Physical Therapy 2001 July;81(7):1351. Review article
Ref ID: 3519

(432) Balagopal P, Bayne E, Sager B, Russell L, Patton N, George D. Effect of lifestyle changes on whole-body protein turnover in obese adolescents. Int J Obes Relat Metab Disord 2003 October;27(10):1250-7. No exercise only group
Ref ID: 354

(433) Balagopal P, George D, Yarandi H, Funanage V, Bayne E. Reversal of obesity-related hypoadiponectinemia by lifestyle intervention: a controlled, randomized study in obese adolescents. J Clin Endocrinol Metab 2005 November;90(11):6192-7. No exercise only group
Ref ID: 302

(434) Balagopal P, George D, Patton N, Yarandi H, Roberts WL, Bayne E, Gidding S. Lifestyle-only intervention attenuates the inflammatory state associated with obesity: a randomized controlled study in adolescents. J Pediatr 2005 March;146(3):342-8. No exercise only group
Ref ID: 312

(435) Balagopal P, Graham TE, Kahn BB, Altomare A, Funanage V, George D. Reduction of elevated serum retinol binding protein in obese children by lifestyle intervention: association with subclinical inflammation. J Clin Endocrinol Metab 2007 May;92(5):1971-4. No exercise only group, No comparative control group
Ref ID: 246

(436) Balagopal P, George D, Sweeten S, Mann KJ, Yarandi H, Mauras N, Vaughan DE. Response of fractional synthesis rate (FSR) of fibrinogen, concentration of D-dimer and fibrinolytic balance to physical activity-based intervention in obese children. J Thromb Haemost 2008 August;6(8):1296-303. No comparative control group
Ref ID: 180

(437) Balagopal PB, Gidding SS, Buckloh LM, Yarandi HN, Sylvester JE, George DE, Funanage VL. Changes in circulating satiety hormones in obese children: a randomized controlled physical activity-based intervention study. Obesity (Silver Spring) 2010 September;18(9):1747-53. No exercise only group, Lifestyle Intervention
Ref ID: 69

(438) Balaguer-Fernandez C, Femenia-Font A, Muedra V, Merino V, Lopez-Castellanoa A. Combined strategies for enhancing the transdermal absorption of midazolam through human skin. Journal of Pharmacy and Pharmacology 2010;62(9):1096-102. Drug intervention study
Ref ID: 5141

(439) Balas-Nakash M, Benítez-Arciniega A, Perichart-Perera O, Valdés-Ramos R, Vadillo-Ortega F. The effect of exercise on cardiovascular risk markers in Mexican school-aged children: comparison between two structured group routines^ien
Efecto del ejercicio sobre marcadores de riesgo cardiovascular en escolares mexicanos: comparación entre dos rutinas grupales^ies. Salud pública Méx 2010 October;52(5):398-405. Not All Participants were Overweight and/or Obese
Ref ID: 3968

(440) Balbinotti MAA, Capozzoli CJ. Motivação à prática regular de atividade física: um estudo exploratório com praticantes em academias de ginástica^ipt. Rev bras educ fís esp 2008 March;22(1):63-80. Study limited to adults
Ref ID: 3969

(441) Balbinotti MAA, Zambonato F, Barbosa MLL, Saldanha RP, Balbinotti CAA. Motivação à prática regular de atividades físicas e esportivas: um estudo comparativo entre estudantes com sobrepeso, obesos e eutróficos^ipt
Motivation of regular physical and sporting activities practice: a comparative study between obeses, overweight, and other students^ien. Motriz rev educ fís (Impr ) 2011 September;17(3):384-94. Not an exercise intervention study
Ref ID: 3970

(442) Balcáza M, Pasquet P, Garine Id. Dieta, actividad física y estado de nutrición en escolares tarahumaras, México^ies. Rev chil salud pública 2009;13(1):30-7. Observational study
Ref ID: 3971

(443) Baldari C, Di LL, Emerenziani GP, Gallotta MC, Sgro P, Guidetti L. Is explosive performance influenced by androgen concentrations in young male soccer players? British Journal of Sports Medicine 2009 March;43(3):191-4. Observational study
Ref ID: 789

(444) Baldwin CM, Lyseng-Williamson KA, Keam SJ. Meropenem: A Review of its Use in the Treatment of Serious Bacterial Infections. Drugs 2008 March 15;68(6):803. Review article
Ref ID: 3520

(445) Balen AH, Anderson RA. Impact of obesity on female reproductive health: British fertility society, policy and practice guidelines. Hum Fertil 2007;10(4):195-206. Review article
Ref ID: 3192

(446) Balfour JAB, Scott LJ. Cinacalcet Hydrochloride. Drugs 2005 January 15;65(2):271-81. Drug intervention study
Ref ID: 3521

(447) Ball EJ, O'Connor J, Abbott R, Steinbeck KS, Davies PSW, Wishart C, Gaskin KJ, Baur LA. Total energy expenditure, body fatness, and physical activity in children aged 6-9 y. American Journal of Clinical Nutrition 2001;74(4):524-8. Cross-sectional study
Ref ID: 5142

(448) Ballard TL, Clapper JA, Specker BL, Binkley TL, Vukovich MD. Effect of protein supplementation during a 6-mo strength and conditioning program on insulin-like growth factor I and markers of bone turnover in young adults.[Erratum appears in Am J Clin Nutr. 2006 Mar;83(3):723]. American Journal of Clinical Nutrition 81(6):1442-8, 2005 Jun 2005;(6):1442-8. Study limited to adults
Ref ID: 2882

(449) Ballard TL, Specker BL, Binkley TL, Vukovich MD. Effect of protein supplementation during a 6-month strength and conditioning program on areal and volumetric bone parameters. Bone 2006 June;38(6):898-904. Study limited to adults
Ref ID: 1385

(450) Ballard TP, Melby CL, Camus H, Cianciulli M, Pitts J, Schmidt S, Hickey MS. Effect of resistance exercise, with or without carbohydrate supplementation, on plasma ghrelin concentrations and postexercise hunger and food intake. Metabolism: Clinical & Experimental 2009 August;58(8):1191-9. Not a randomized controlled trial (RCT)
Ref ID: 717

(451) Ballew C, Khan LK, Kaufmann R, Mokdad A, Miller DT, Gunter EW. Blood lead concentration and children's anthropometric dimensions in the Third National Health and Nutrition Examination Survey (NHANES III), 1988-1994. Journal of Pediatrics 1999;134(5):623-30. Survey or questionnaire
Ref ID: 5143

(452) Ballin AC, Koerner HN, Ballin CH, Pereira R, Alcântara LJL, Taques GR, Mocellin M. Assimetria de tonsilas palatinas: experiência de 10 anos do serviço de otorrinolaringologia do Hospital de Clínicas da Universidade Federal do Paraná^ipt
Palatine tonsils asymmetry: 10 years experience of the otorhinolaryngology service of the Clinical Hospital of the Federal University of Paraná^ien. Arq int otorrinolaringol (Impr ) 2011 March;15(1):67-71. Retrospective study
Ref ID: 3972

(453) Bammann K, Peplies J, Pigeot I, Ahrens W. [IDEFICS: a multicenter European project on diet- and lifestyle-related disorders in children]. [German]. Medizinische Klinik 2007 March 15;102(3):230-5. Description of study from review or magazine or etc. (not the actual study)
Ref ID: 1263

(454) Bangirana P, Giordani B, John CC, Page C, Opoka RO, Boivin MJ. Immediate neuropsychological and behavioral benefits of computerized cognitive rehabilitation in Ugandan pediatric cerebral malaria survivors. Journal of developmental and behavioral pediatrics : JDBP 2009;30:310-8. Rehabilitation study
Ref ID: 4680

(455) Bangirana P, Allebeck P, Boivin MJ, John CC, Page C, Ehnvall A, Musisi S. Cognition, behaviour and academic skills after cognitive rehabilitation in Ugandan children surviving severe malaria: a randomised trial. BMC neurology 2011;11:96. Rehabilitation study
Ref ID: 4681

(456) Banks J, Sharp DJ, Hunt LP, Shield JP. Evaluating the transferability of a hospital-based childhood obesity clinic to primary care: a randomised controlled trial. British Journal of General Practice 2012 January;62(594):e6-12. Lifestyle Intervention
Ref ID: 2404

(457) Banks J, Williams J, Cumberlidge T, Cimonetti T, Sharp DJ, Shield JP. Is healthy eating for obese children necessarily more costly for families? British Journal of General Practice 2012 January;62(594):e1-e5. Diet Intervention Study
Ref ID: 2405

(458) Baptista MN, Vargas JF, Baptista ASD. Depressão e qualidade de vida em uma amostra brasileira de obesos mórbidos^ipt
Depression and quality of life in a morbid obese brazilian sample^ien. Aval psicol 2008 August;7(2):235-47. Survey or questionnaire
Ref ID: 3973

(459) Bar-Or O, Blimkie CJ, Hay JA, MacDougall JD, Ward DS, Wilson WM. Voluntary dehydration and heat intolerance in cystic fibrosis. Lancet 1992 March 21;339(8795):696-9. Study less than 4 weeks
Ref ID: 2291

(460) Baranowski T, Simons-Morton B, Hooks P, Henske J, Tiernan K, Dunn JK, Burkhalter H, Harper J, Palmer J. A center-based program for exercise change among black-American families. Health Education Quarterly 1990;17(2):179-96. Not All Participants were Overweight and/or Obese
Ref ID: 2312

(461) Baranowski T, Baranowski JC, Cullen KW, Thompson DI, Nicklas T, Zakeri IE, Rochon J. The Fun, Food, and Fitness Project (FFFP): the Baylor GEMS pilot study. Ethn Dis 2003;13(1 Suppl 1):S30-S39. No exercise only group
Ref ID: 368

(462) Baranowski T, Baranowski J, Thompson D, Buday R, Jago R, Griffith MJ, Islam N, Nguyen N, Watson KB. Video game play, child diet, and physical activity behavior change a randomized clinical trial. American Journal of Preventive Medicine 2011 January;40(1):33-8. Not All Participants were Overweight and/or Obese
Ref ID: 1101

(463) Baranowski T, Abdelsamad D, Baranowski J, O'Connor TM, Thompson D, Barnett A, Cerin E, Chen TA. Impact of an active video game on healthy children's physical activity. Pediatrics 2012 March;129(3):e636-e642. Not All Participants were Overweight and/or Obese
Ref ID: 2407

(464) Barba G, Troiano E, Russo P, Venezia A, Siani A. Inverse association between body mass and frequency of milk consumption in children. BR J NUTR 2005;93(1):15-9. Survey or questionnaire
Ref ID: 5144

(465) Barbeau P, Gutin B, Litaker M, Owens S, Riggs S, Okuyama T. Correlates of individual differences in body-composition changes resulting from physical training in obese children. Am J Clin Nutr 1999 April;69(4):705-11. No comparative control group
Ref ID: 412

(466) Barbeau P, Litaker MS, Woods KF, Lemmon CR, Humphries MC, Owens S, Gutin B. Hemostatic and inflammatory markers in obese youths: effects of exercise and adiposity. J Pediatr 2002 September;141(3):415-20. No exercise only group
Ref ID: 372

(467) Barbeau P, Gutin B, Litaker MS, Ramsey LT, Cannady WE, Allison J, Lemmon CR, Owens S. Influence of physical training on plasma leptin in obese youths. Can J Appl Physiol 2003 June;28(3):382-96. No exercise only group
Ref ID: 357

(468) Barbeau P, Johnson MH, Howe CA, Allison J, Davis CL, Gutin B, Lemmon CR. Ten months of exercise improves general and visceral adiposity, bone, and fitness in black girls. Obesity 2007 August;15(8):2077-85. Not All Participants were Overweight and/or Obese
Ref ID: 1178

(469) Barbieri RL, Gargiulo AR. Metformin for the treatment of the polycystic ovary syndrome. [Review] [118 refs]. Minerva Ginecologica 2004 February;56(1):63-79. Review article
Ref ID: 1665

(470) Barbosa Filho VC, Reges LAG, Souza EAd, Ribeiro EAG, Lima AB. Práticas esportivas e recreativas em adolescentes com excesso de peso: análise da composição corporal e do desempenho motor^ipt
Sports and recreational activities in overweight adolescents: analyze of body composition and motor performance^ien. Motriz rev educ fís (Impr ) 2011 June;17(2):264-73. Not a randomized controlled trial (RCT)
Ref ID: 3974

(471) Barbosa FP, Oliveira HB, Fernandes PR, Fernandes Filho J. Comparação de equaçoes de estimativa do consumo máiximo de oxigênio em indivíduos jovens^ipt. Acta cir bras 2005;20(supl.1):82-7. Not a randomized controlled trial (RCT)
Ref ID: 3975

(472) Barbosa VLP, Cézar C, Vítolo MR, Lopez FA. Atuação ambulatorial do profissional de educação física no atendimento a crianças e adolescentes obesos^ipt
Outpatient performance of the physical education professional caring for the obese children and adolescents^ien. Rev bras med esporte 1999 February;5(1):31-4. Not an exercise intervention study
Ref ID: 695

(473) Bardsley-Elliot A, Plosker GL. Nelfinavir: An Update on its Use in HIV Infection. Drugs 2000 March;59(3):581-620. Review article
Ref ID: 3522

(474) Bardwell G, Mujuru P, Fitch C, Seidel G, Hu W, Sogodogo K, Chester A. Engaging Youth to Examine Lifestyle Behaviors through Authentic Research with University Partnerships. International Electronic Journal of Health Education 2007 January 1;10:95-103. Cross-sectional study
Ref ID: 3898

(475) Barkin SL, Gesell SB, Poe EK, Ip EH. Changing overweight Latino preadolescent body mass index: the effect of the parent-child dyad. Clinical Pediatrics 2011 January;50(1):29-36. Not All Participants were Overweight and/or Obese
Ref ID: 2408

(476) Barlow SE, Dietz WH. Obesity evaluation and treatment: Expert committee recommendations. Pediatrics 1998;102(3). Review article
Ref ID: 5145

(477) Barnes MJ, Mundel T, Stannard SR. The effects of acute alcohol consumption and eccentric muscle damage on neuromuscular function. Applied Physiology, Nutrition, & Metabolism = Physiologie Appliquee, Nutrition et Metabolisme 2012 February;37(1):63-71. Not an exercise intervention study
Ref ID: 2410

(478) Barnett TA, O'Loughlin J, Gauvin L, Paradis G, Hanley J. Opportunities for student physical activity in elementary schools: A cross-sectional survey of frequency and correlates. Health Education & Behavior 2006;33(2):215-32. Survey or questionnaire
Ref ID: 5146

(479) Barnow S, Bernheim D, Schroder C, Lauffer H, Fusch C, Freyberger HJ. [Obesity in childhood and adolescence--first results of a multimodal intervention study in Mecklenburg-Vorpommern]. [German]. Psychotherapie, Psychosomatik, Medizinische Psychologie 2003 January;53(1):7-14. Not All Participants were Overweight and/or Obese, Lifestyle Intervention
Ref ID: 1779

(480) Barondess JA. Health through the urban lens. Journal of Urban Health-Bulletin of the New York Academy of Medicine 2008;85(5):787-801. Not an exercise intervention study
Ref ID: 5147

(481) Barr-Anderson DJ, Neumark-Sztainer D, Schmitz KH, Ward DS, Conway TL, Pratt C, Baggett CD, Lytle L, Pate RR. But I like PE: factors associated with enjoyment of physical education class in middle school girls. Research Quarterly for Exercise & Sport 2008 March;79(1):18-27. Cross-sectional study
Ref ID: 966

(482) Barrack MT, Van Loan MD, Rauh MJ, Nichols JF. Physiologic and behavioral indicators of energy deficiency in female adolescent runners with elevated bone turnover. American Journal of Clinical Nutrition 2010 September;92(3):652-9. Cross-sectional study
Ref ID: 471

(483) Barreto SM, Passos VMA, Firmo JOA, Guerra HL, Vidigal PG, Lima-Costa MFF. Hypertension and clustering of cardiovascular risk factors in a community in Southeast Brazil - The Bambuí Health and Ageing Study. Arq bras cardiol 2001 December;77(6):576-81. Study limited to adults
Ref ID: 3976

(484) Barrett LA, Morris JG, Stensel DJ, Nevill ME. Exercise and postprandial plasma triacylglycerol concentrations in healthy adolescent boys. Medicine & Science in Sports & Exercise 2007 January;39(1):116-22. Study less than 4 weeks
Ref ID: 1284

(485) Barrett ML, Udani JK. A proprietary alpha-amylase inhibitor from white bean (Phaseolus vulgaris): a review of clinical studies on weight loss and glycemic control. [Review]. Nutrition Journal 2011;10(1):24-33. Review article
Ref ID: 2411

(486) Barría P, Amigo C. Transición Nutricional: una revisión del perfil latinoamericano^ies. Arch latinoam nutr 2006 March;56(1):3-11. Review article
Ref ID: 3977

(487) Barros C, Araújo T, Andrade E, Cruciani F, Matsudo V. Avaliação das variáveis de força muscular, agilidade e composição corporal em crianças vivendo com HIV/AIDS^ipt. Rev bras ciênc mov 2006;14(4):47-54. Cross-sectional study
Ref ID: 3978

(488) Barros JWO, de Almeida MB, dos Santos MAM, de Santana PR, Campos FDCE, Leandro CG. Can birth weight influence nutritional status, physical activity levels and health-related physical fitness levels of children and adolescents? Revista de Nutricao-Brazilian Journal of Nutrition 2011;24(5):777-84. Review article
Ref ID: 5148

(489) Barros MBdA, Zanchetta LM, Moura ECd, Malta DC. Auto-avaliação da saúde e fatores associados, Brasil, 2006^ipt
Auto-evaluación de la salud y factores asociados, Brasil, 2006^ies
Self-rated health and associated factors, Brazil, 2006^ien. Rev saúde pública 2009 November;43(supl.2):27-37. Study limited to adults
Ref ID: 3980

(490) Barros SSH, Lopes AdS, Barros MVGd. Prevalência de baixo nivel de atividade física em crianças pré-escolares^ipt
Prevalence of low physical activity level among preschool children^ien. Rev bras cineantropom desempenho hum 2012;14(4):390-400. Cross-sectional study
Ref ID: 3981

(491) Barry JJ. The Recognition and Management of Mood Disorders as a Comorbidity of Epilepsy. Epilepsia (Series 4) 2003 April 2;44:30-40. Review article
Ref ID: 3524

(492) Barshop NJ, Sirlin CB, Schwimmer JB, Lavine JE. Review article: Epidemiology, pathogenesis and potential treatments of paediatric non-alcoholic fatty liver disease. Aliment Pharmacol Ther 2008;28(1):13-24. Review article
Ref ID: 3193

(493) Barst RJ, Ivy D, Dingemanse J, Widlitz A, Schmitt K, Doran A, Bingaman D, Nguyen N, Gaitonde M, van Giersbergen PL. Pharmacokinetics, safety, and efficacy of bosentan in pediatric patients with pulmonary arterial hypertension. Clinical Pharmacology & Therapeutics 2003 April;73(4):372-82. Drug intervention study
Ref ID: 1756

(494) Bartlett AV, Torun B, Morales C, Cano F, Cruz JR. Oral gentamicin is not effective treatment for persistent diarrhea. Acta Paediatrica Supplement 1992 September;381:149-54. Drug intervention study
Ref ID: 2282

(495) Bartolucci G, Younger J. Tentative classification of neuropsychiatric disturbances in Prader-Willi syndrome. Journal of Intellectual Disability Research 1994 December;38(6):621-9. Review article
Ref ID: 3802

(496) Baruki SBS, Rosado LEFPdL, Rosado GP, Ribeiro RdCL. Associação entre estado nutricional e atividade física em escolares da Rede Municipal de Ensino em Corumbá - MS^ipt. Rev bras med esporte 2006 April;12(2):90-4. Cross-sectional study
Ref ID: 3982

(497) Bas P, Romagnoli M, Gomez-Cabrera MC, Bas JL, Aura JV, Franco N, Bas T. Beneficial effects of aerobic training in adolescent patients with moderate idiopathic scoliosis. European Spine Journal 2011 August;20:Suppl-9. Not a randomized controlled trial (RCT)
Ref ID: 2412

(498) Bass MM, Duchowny CA, Llabre MM. The effect of therapeutic horseback riding on social functioning in children with autism. Journal of autism and developmental disorders 2009;39:1261-7. Not a randomized controlled trial (RCT)
Ref ID: 4683

(499) Bass SL, Naughton G, Saxon L, Iuliano BS, Daly R, Briganti EM, Hume C, Nowson C. Exercise and calcium combined results in a greater osteogenic effect than either factor alone: a blinded randomized placebo-controlled trial in boys. Journal of bone and mineral research : the official journal of the American Society for Bone and Mineral Research 2007;22:458-64. Inappropriate Comparison Group
Ref ID: 4684

(500) Bassan H, Bassan M, Pinhasov A, Kariv N, Giladi E, Gozes I, Harel S. The pregnant spontaneously hypertensive rat as a model of asymmetric intrauterine growth retardation and neurodevelopmental delay. Hypertension in Pregnancy 2005;24(3):201-11. Animal study
Ref ID: 5149

(501) Bassett DR. Physical activity of Canadian and American children: a focus on youth in Amish, Mennonite, and modern cultures. Applied Physiology Nutrition and Metabolism-Physiologie Appliquee Nutrition et Metabolisme 2008;33(4):831-5. Review article
Ref ID: 5150

(502) Basso RP, Jamami M, Pessoa BV, Labadessa IG, Regueiro EMG, Di Lorenzo VAP. Avaliação da capacidade de exercício em adolescentes asmáticos e saudáveis^ipt
Assessment of exercise capacity among asthmatic and healthy adolescents^ien. Rev bras fisioter 2010 June;14(3):252-8. Review article
Ref ID: 3983

(503) Bassols J, Prats-Puig A, Vazquez-Ruiz M, Garcia-Gonzalez MM, Martinez-Pascual M, Avelli P, Martinez-Martinez R, Fabrega R, Colomer-Virosta C, Soriano-Rodriguez P, Diaz M, de Zegher F, Ibanez L, Lopez-Bermejo A. Placental FTO expression relates to fetal growth. International Journal of Obesity 2010;34(9):1365-70. Not an exercise intervention study
Ref ID: 5151

(504) Bastani F, Hidarnia A, Montgomery KS, Aguilar-Vafaei ME, Kazemnejad A. Does relaxation education in anxious primigravid Iranian women influence adverse pregnancy outcomes?: a randomized controlled trial. The Journal of perinatal & neonatal nursing 2006;20:138-46. Study limited to adults
Ref ID: 4685

(505) Bateman A, Culpan FJ, Pickering AD, Powell JH, Scott OM, Greenwood RJ. The effect of aerobic training on rehabilitation outcomes after recent severe brain injury: a randomized controlled evaluation. Archives of Physical Medicine & Rehabilitation 2001 February;82(2):174-82. Not All Participants were Overweight and/or Obese
Ref ID: 1928

(506) Bateman LA, Slentz CA, Willis LH, Shields AT, Piner LW, Bales CW, Houmard JA, Kraus WE. Comparison of aerobic versus resistance exercise training effects on metabolic syndrome (from the Studies of a Targeted Risk Reduction Intervention Through Defined Exercise - STRRIDE-AT/RT). American Journal of Cardiology 2011 September 15;108(6):838-44. Study limited to adults
Ref ID: 1087

(507) Batista Júnior ML, Franchini E, Uchida MC, Rosa LFBPC. Efeito da suplementação de creatina sobre o desempenho na velocidade do swing e no tempo para percorrer três bases (home base - terceira base) em atletas da seleção brasileira de beisebol juvenil (16 a 18 anos)^ipt. Rev bras ciênc mov 2005;13(4):85-92. Diet Intervention or Supplement Study
Ref ID: 3984

(508) Batista EdS, Sabarense CM, Priore SE, Rosa DD, Montezano IM, Peluzio MdCG. Hábito alimentar, níveis de lipídios sangüíneos e o status antioxidante de adultos jovens fumantes e não fumantes^ipt. Rev nutr 2009 June;22(3):377-88. Cross-sectional study
Ref ID: 3985

(509) Batrouni L, Navarro A, Sabulsky J, Fanto S, Rodriguez A. Situación alimentaria de escolares en relación con su condición social: Córdoba, República Argentina. Arch latinoam nutr 1993 March;43(1):12-9. Diet Intervention or Supplement Study
Ref ID: 3986

(510) Battaglini CL, Hackney AC, Garcia R, Groff D, Evans E, Shea T. The effects of an exercise program in leukemia patients. Integrative Cancer Therapies 2009 June;8(2):130-8. Study limited to adults
Ref ID: 699

(511) Battistella PA, Ruffilli R, Moro R, Fabiani M, Bertoli S, Antolini A, Zacchello F. A placebo-controlled crossover trial of nimodipine in pediatric migraine. Headache 1990;30(5):264-8. Drug intervention study
Ref ID: 3194

(512) Bauer CR, Langer JC, Shankaran S, Bada HS, Lester B, Wright LL, Krause SH, Smeriglio VL, Finnegan LP, Maza PL, Verter J. Acute neonatal effects of cocaine exposure during pregnancy. Archives of Pediatrics & Adolescent Medicine 2005;159:824-34. Not an exercise intervention study
Ref ID: 4686

(513) Baum M. Ask the expert. Pediatr Nephrol 2000 January 15;14(2):184-5. Abstract
Ref ID: 524

(514) Bautista-Castano I, Sangil-Monroy M, Serra-Majem L, Comite dN. [Knowledge and gaps on the role of nutrition and physical activity on the onset of childhood obesity]. [Spanish]. Medicina Clinica 2004 December 4;123(20):782-93. Review article
Ref ID: 1565

(515) Baynard T, Miller WC, Fernhall B. Effects of exercise on vasodilatory capacity in endurance- and resistance-trained men. European Journal of Applied Physiology 2003 March;89(1):69-73. Not a randomized controlled trial (RCT)
Ref ID: 1768

(516) Bayne-Smith M, Fardy PS, Azzollini A, Magel J, Schmitz KH, Agin D. Improvements in heart health behaviors and reduction in coronary artery disease risk factors in urban teenaged girls through a school-based intervention: the PATH program. American Journal of Public Health 2004 September;94(9):1538-43. Lifestyle Intervention
Ref ID: 1593

(517) Bárzaga Arencibia ZM, Barranco Pedraza LM, López Leyva A, de la Torre Rosés M. La moxibustión en el tratamiento de la infertilidad femenina asociada a la insuficiencia de Yang de riñón^ies
Moxibustion in the treatment of feminine infertility associated to Yang insuffiency of kidney^ien. Arch méd Camaguey 2009;13(1). Drug intervention study
Ref ID: 3987

(518) BÃ©rard E, Crosnier HÃ, Six-Beneton A, Chevallier T, Cochat P, Broyer M. Recombinant human growth hormone treatment of children on hemodialysis. Pediatr Nephrol 1998 May;12(4):304-10. Drug intervention study
Ref ID: 3525

(519) Bean MK, Mazzeo SE, Stern M, Bowen D, Ingersoll K. A values-based Motivational Interviewing (MI) intervention for pediatric obesity: study design and methods for MI Values. Contemporary Clinical Trials 2011 September;32(5):667-74. Description of study from review or magazine or etc. (not the actual study)
Ref ID: 1091

(520) Beauchamp MR, Rhodes RE, Hua S, Morton KL, Kreutzer C, Liang JA, Khou KY, Dominelli PB, Daoud DM, Sherman MF, Dunlop WL, Sheel AW. Testing the effects of an expectancy-based intervention among adolescents: Can placebos be used to enhance physical health? Psychology Health & Medicine 2011 August;16(4):405-17. Not a randomized controlled trial (RCT)
Ref ID: 2414

(521) Becerra C, Gonzales GF, Villena A, De la Cruz D, Florián A. Prevalencia de anemia en gestantes, Hospital Regional de Pucallpa, Perú^ies. Rev panam salud pública 1998 May;3(5):285-92. Cross-sectional study
Ref ID: 3988

(522) Beck CC, Lopes AdS, Giuliano IdCB, Borgatto AF. Fatores de risco cardiovascular em adolescentes de município do sul do Brasil: prevalência e associações com variáveis sociodemográficas^ipt
Cardiovacular risk factors in adolescents from a town in the Brazilian South: prevalence and association with sociodemographic variables^ien. Rev bras epidemiol 2011 March;14(1):36-49. Cross-sectional study
Ref ID: 3989

(523) Becker AJ, Uckert S, Stief CG, Jonas U. Growth hormone, somatomedins and men's health. The aging male : the official journal of the International Society for the Study of the Aging Male 2002;5:258-62. Review article
Ref ID: 4687

(524) Becker DJ, Gordon RY, Morris PB, Yorko J, Gordon YJ, Li M, Iqbal N. Simvastatin vs therapeutic lifestyle changes and supplements: randomized primary prevention trial. Mayo Clinic Proceedings 2008 July;83(7):758-64. Drug intervention study
Ref ID: 920

(525) Becker MdMC, Silva OB, Moreira IEG, Victor EG. Pressão arterial em adolescentes durante teste ergométrico^ipt. Arq bras cardiol 2007 March;88(3):329-33. Cross-sectional study
Ref ID: 3990

(526) Beckett C, Durnin JV, Aitchison TC, Pollitt E. Effects of an energy and micronutrient supplement on anthropometry in undernourished children in Indonesia. European Journal of Clinical Nutrition 2000 May;54:Suppl-9. Diet Intervention or Supplement Study
Ref ID: 1969

(527) Beckham SG, Earnest CP. Metabolic cost of free weight circuit weight training. Journal of Sports Medicine & Physical Fitness 2000 June;40(2):118-25. Not a randomized controlled trial (RCT)
Ref ID: 1955

(528) Becque MD, Katch VL, Rocchini AP, Marks CR, Moorehead C. Coronary risk incidence of obese adolescents: reduction by exercise plus diet intervention. Pediatrics 1988 May;81(5):605-12. No exercise only group
Ref ID: 447

(529) Beech BM, Klesges RC, Kumanyika SK, Murray DM, Klesges L, McClanahan B, Slawson D, Nunnally C, Rochon J, McLain-Allen B, Pree-Cary J. Child- and parent-targeted interventions: the Memphis GEMS pilot study. Ethn Dis 2003;13(1 Suppl 1):S40-S53. No exercise only group
Ref ID: 367

(530) Beech BM, Kumanyika SK, Baranowski T, Davis M, Robinson TN, Sherwood NE, Taylor WC, Relyea G, Zhou A, Pratt C, Owens A, Thompson NS. Parental cultural perspectives in relation to weight-related behaviors and concerns of African-American girls. Obesity Research 2004 September;12:Suppl-19S. No exercise only group
Ref ID: 1580

(531) Behar Astudillo R, Hernández T. Deportes y trastornos de la conducta alimentaria. Rev méd Chile 2002 March;130(3):287-94. Survey or questionnaire
Ref ID: 751

(532) Behar A. Trastornos de la conducta alimentaria no especificados, síndromes parciales y cuadros subclínicos: una alerta para la atención primaria: [revisión]^ies. Rev méd Chile 2008 December;136(12):1589-98. Review article
Ref ID: 3991

(533) Beilin L, Burke V, Milligan R. Strategies for prevention of adult hypertension and cardiovascular risk behaviour in childhood. An Australian perspective. Journal of Human Hypertension 1996 February;10:Suppl-4. Cross-sectional study
Ref ID: 2180

(534) Belcher JD, Ellison RC, Shepard WE, Bigelow C, Webber LS, Wilmore JH, Parcel GS, Zucker DM, Luepker RV. Lipid and lipoprotein distributions in children by ethnic group, gender, and geographic location--preliminary findings of the Child and Adolescent Trial for Cardiovascular Health (CATCH). Preventive Medicine 1993 March;22(2):143-53. Lifestyle Intervention
Ref ID: 2268

(535) Bell C, Seals DR, Monroe MB, Day DS, Shapiro LF, Johnson DG, Jones PP. Tonic sympathetic support of metabolic rate is attenuated with age, sedentary lifestyle, and female sex in healthy adults. Journal of Clinical Endocrinology & Metabolism 2001 September;86(9):4440-4. Study limited to adults
Ref ID: 1896

(536) Bell C, Stob NR, Seals DR. Thermogenic responsiveness to beta-adrenergic stimulation is augmented in exercising versus sedentary adults: role of oxidative stress. Journal of Physiology 2006 February 1;570(Pt:3):3-35. Study limited to adults
Ref ID: 1426

(537) Bell EF, Hansen NI, Morriss FH, Stoll BJ, Ambalavanan N, Gould JB, Laptook AR, Walsh MC, Carlo WA, Shankaran S, Das A, Higgins RD. Impact of Timing of Birth and Resident Duty-Hour Restrictions on Outcomes for Small Preterm Infants. Pediatrics 2010;126(2):222-31. Not an exercise intervention study
Ref ID: 5152

(538) Bellissimo N, Thomas SG, Goode RC, Anderson GH. Effect of short-duration physical activity and ventilation threshold on subjective appetite and short-term energy intake in boys. Appetite 2007;49:644-51. Study less than 4 weeks
Ref ID: 4688

(539) Belury MA. DIETARY CONJUGATED LINOLEIC ACID IN HEALTH: Physiological Effects and Mechanisms of Action. Annual Review of Nutrition 2002 August;22(1):505. Review article
Ref ID: 3526

(540) Ben-Menachem E. Vigabatrin's Complicated Journeyâ€”To Be or Not to Be? Epilepsy Currents 2009 September;9(5):130-2. Retrospective study
Ref ID: 3527

(541) Ben Ounis OF, Elloumi MF, Ben C, I, Zbidi AF, Amri MF, Lac GF, Tabka Z. Effects of two-month physical-endurance and diet-restriction programmes on lipid profiles and insulin resistance in obese adolescent boys. Diabetes Metab 2008;34:595-600. No comparative control group
Ref ID: 462

(542) Ben AN, Chaouachi A, Chamari K, Chtara M, Castagna C. Positional role and competitive-level differences in elite-level men's basketball players. Journal of Strength & Conditioning Research 2010 May;24(5):1346-55. Cross-sectional study
Ref ID: 538

(543) Ben OO, Elloumi M, Amri M, Zbidi A, Tabka Z. Impact of diet, exercise and diet combined with exercise programs on plasma lipoprotein and adiponectin levels in obese girls. J Sports Sci Med 2008;7(4):437-45. No comparative control group
Ref ID: 461

(544) Ben OO, Elloumi M, Zouhal H, Makni E, Denguezli M, Amri M, Lac G, Tabka Z. Effect of individualized exercise training combined with diet restriction on inflammatory markers and IGF-1/IGFBP-3 in obese children. Ann Nutr Metab 2010;56(4):260-6. No exercise only group
Ref ID: 47

(545) Ben SH, Gaigi I, El FH, Gaigi S, El AJ. [Bulimia and anorexia among the teenagers]. [French]. Tunisie Medicale 2011 November;89(11):820-4. Not a randomized controlled trial (RCT)
Ref ID: 2416

(546) Bender BG, Fuhlbrigge A, Walders N, Zhang L. Overweight, race, and psychological distress in children in the Childhood Asthma Management Program. Pediatrics 2007 October;120(4):805-13. Not a randomized controlled trial (RCT)
Ref ID: 214

(547) Beneke R, Hutler M, Jung M, Leithauser RM. Modeling the blood lactate kinetics at maximal short-term exercise conditions in children, adolescents, and adults. Journal of Applied Physiology 2005 August;99(2):499-504. Not an exercise intervention study
Ref ID: 1496

(548) Beneke R, Hutler M, Leithauser RM. Carbohydrate and fat metabolism related to blood lactate in boys and male adolescents. European Journal of Applied Physiology 2009;105(2):257-63. Not an exercise intervention study
Ref ID: 5153

(549) Benevento BT, Sipski ML. Neurogenic Bladder, Neurogenic Bowel, and Sexual Dysfunction in People With Spinal Cord Injury. Physical Therapy 2002 June;82(6):601-12. Review article
Ref ID: 3528

(550) Bengmark S, Gil A. Productos finales de la glicaciÃ³n y de la lipoxidaciÃ³n como amplificadores de la inflamaciÃ³n: papel de los alimentos. (Spanish). Nutricion Hospitalaria 2007 November;22(6):625-40. Review article
Ref ID: 3529

(551) Benini J, Karolczak APB. Benefícios de um programa de educação postural para alunos de uma escola municipal de Garibaldi, RS^ipt
Benefits of a posture education program for schoolchildren in the city of Garibaldi, RS^ien. Fisioter pesqui 2010 December;17(4):346-51. Educational intervention
Ref ID: 3992

(552) Benjamin SE, Ammerman A, Sommers J, Dodds J, Neelon B, Ward DS. Nutrition and physical activity self-assessment for child care (NAP SACC): results from a pilot intervention. Journal of nutrition education and behavior 2007;39:142-9. Lifestyle Intervention
Ref ID: 4689

(553) Benjamin SE, Tate DF, Bangdiwala SI, Neelon BH, Ammerman AS, Dodds JM, Ward DS. Preparing Child Care Health Consultants to address childhood overweight: a randomized controlled trial comparing web to in-person training. Matern Child Health J 2008 September;12(5):662-9. Not an exercise intervention study
Ref ID: 221

(554) Bennell K, Khan KM, Matthews B, De GM, Cook E, Holzer K, Wark JD. Hip and ankle range of motion and hip muscle strength in young female ballet dancers and controls. British Journal of Sports Medicine 1999 October;33(5):340-6. Cross-sectional study
Ref ID: 1994

(555) Bensch GW, Greos LS, Gawchik S, Kpamegan E, Newman KB. Linear growth and bone maturation are unaffected by 1 year of therapy with inhaled flunisolide hydrofluoroalkane in prepubescent children with mild persistent asthma: a randomized, double-blind, placebo-controlled trial. Annals of allergy, asthma & immunology : official publication of the American College of Allergy, Asthma, & Immunology 2011;107:323-9. Drug intervention study
Ref ID: 4690

(556) Benson AC, Torode ME, Singh MA. Muscular strength and cardiorespiratory fitness is associated with higher insulin sensitivity in children and adolescents. International Journal of Pediatric Obesity 2006;1(4):222-31. Cross-sectional study
Ref ID: 1299

(557) Benson AC, Torode ME, Fiatarone Singh MA. A rationale and method for high-intensity progressive resistance training with children and adolescents. Contemp Clin Trials 2007 July;28(4):442-50. Description versus conduct of study
Ref ID: 255

(558) Benson AC, Torode ME, Fiatarone Singh MA. The effect of high-intensity progressive resistance training on adiposity in children: a randomized controlled trial. Int J Obes (Lond) 2008 June;32(6):1016-27. Not All Participants were Overweight and/or Obese
Ref ID: 194

(559) Benson JE, Geiger CJ, Eiserman PA, Wardlaw GM. Relationship between nutrient intake, body mass index, menstrual function, and ballet injury. Journal of the American Dietetic Association 1989 January;89(1):58-63. Diet Intervention or Supplement Study
Ref ID: 2326

(560) Bentley ME, Caulfield LE, Ram M, Santizo MC, Hurtado E, Rivera JA, Ruel MT, Brown KH. Zinc supplementation affects the activity patterns of rural Guatemalan infants. Journal of Nutrition 1997;127(7):1333-8. Subjects less than 2 years old
Ref ID: 5154

(561) Berenson AB, Radecki CM, Grady JJ, Rickert VI, Thomas A. A prospective, controlled study of the effects of hormonal contraception on bone mineral density. Obstetrics & Gynecology 2001 October;98(4):576-82. Study limited to adults
Ref ID: 1892

(562) Berenson AB, Breitkopf CR, Grady JJ, Rickert VI, Thomas A. Effects of hormonal contraception on bone mineral density after 24 months of use. Obstetrics & Gynecology 2004 May;103(5:Pt 1):t-906. Study limited to adults
Ref ID: 1635

(563) Berenson GS, Shear CL, Chiang YK, Webber LS, Voors AW. Combined low-dose medication and primary intervention over a 30-month period for sustained high blood pressure in childhood. The American journal of the medical sciences 1990;299:79-86. Not an exercise intervention study
Ref ID: 1073

(564) Beresford SA, Locke E, Bishop S, West B, McGregor BA, Bruemmer B, Duncan GE, Thompson B. Worksite study promoting activity and changes in eating (PACE): design and baseline results. Obesity 2007 November;15:Suppl-15S. Study limited to adults
Ref ID: 1127

(565) Bergamo VR. Estabilidade: aspecto significativo na previsão do talento no basquetebol feminino^ipt. Rev bras ciênc mov 2004;12(2):51-6. Not an exercise intervention study
Ref ID: 3994

(566) Bergh C, Brodin U, Lindberg G, Sodersten P. Randomized controlled trial of a treatment for anorexia and bulimia nervosa. Proceedings of the National Academy of Sciences of the United States of America 2002 July 9;99(14):9486-91. Not an exercise intervention study
Ref ID: 1813

(567) Bergmann MLdA, Bergmann GG, Halpern R, Rech RR, Constanzi CB, Alli LR. Colesterol total e fatores associados: estudo de base escolar no sul do Brasil^ipt
Associated factors to total cholesterol: school based study in southern Brazil^ien
Colesterol total y factores asociados: estudio de base escolar en el sur del Brasil^ies. Arq bras cardiol 2011 July;97(1):17-25. Cross-sectional study
Ref ID: 3995

(568) Bergstrom J, Hultman E. Synthesis of muscle glycogen in man after glucose and fructose infusion. Acta Medica Scandinavica 1967 July;182(1):93-107. Not an exercise intervention study
Ref ID: 2417

(569) Berkey CS, Rockett HR, Gillman MW, Colditz GA. One-year changes in activity and in inactivity among 10- to 15-year-old boys and girls: relationship to change in body mass index. Pediatrics 2003 April;111(4:Pt 1):t-43. Cross-sectional study
Ref ID: 1762

(570) Berleze A, Haeffner LSB, Valentini NC. Desempenho motor de crianças obesas: uma investigação do processo e produto de habilidades motoras fundamentais^ipt. Rev bras cineantropom desempenho hum 2007 June;9(2). Cross-sectional study
Ref ID: 595

(571) BermÃºdez-HumarÃ¡n LG, Kharrat P, Chatel JM, Langella P. Lactococci and lactobacilli as mucosal delivery vectors for therapeutic proteins and DNA vaccines. Microbial Cell Factories 2011 January 2;10(Suppl 1):1-10. Review article
Ref ID: 3530

(572) Bernbaum JC, Umbach DM, Ragan NB, Ballard JL, Archer JL, Schmidt-Davis H, Rogan WJ. Pilot studies of estrogen-related physical findings in wants. Environmental Health Perspectives 2008;116(3):416-20. Subjects less than 2 years old
Ref ID: 5155

(573) Bernhoft A, Nafstad I, Engen P, Skaare JU. Effects of Prenatal and Postnatal Exposure to 3,3',4,4',5-Pentachlorobiphenyl on Physical Development, Neurobehavior and Xenobiotic-Metabolizing Enzymes in Rats. Environmental Toxicology and Chemistry 1994;13(10):1589-97. Animal study
Ref ID: 5156

(574) Berntsen S, Mowinckel P, Carlsen KH, Lodrup Carlsen KC, Pollestad Kolsgaard ML, Joner G, Anderssen SA. Obese children playing towards an active lifestyle. Int J Pediatr Obes 2010;5(1):64-71. No exercise only group, No comparative control group
Ref ID: 118

(575) Bertakis KD, Azari R. The impact of obesity on primary care visits. Obes Res 2005 September;13(9):1615-23. Not an exercise intervention study
Ref ID: 295

(576) Bertapelli F, Gorla JI, Costa LT, Freire F. Composição corporal em jovens com síndrome de down: aspectos genéticos, ambientais e fisiológicos^ipt
Body composition in down syndrome youngters: genetic, environmental and physiologic aspects^ien. Arq ciências saúde UNIPAR 2012 August;15(2). Review article
Ref ID: 3996

(577) Bertrais S, Preziosi P, Mermen L, Galan P, Hercberg S, Oppert JM. Sociodemographic and Geographic Correlates of Meeting Current Recommendations for Physical Activity in Middle-Aged French Adults: the SupplÃ©mentation en Vitamines et MinÃ©raux Antioxydants (SUVIMAX) Study. American Journal of Public Health 2004 September;94(9):1560-6. Cross-sectional study
Ref ID: 3803

(578) Besier TF, Lloyd DG, Ackland TR, Cochrane JL. Anticipatory effects on knee joint loading during running and cutting maneuvers. Medicine & Science in Sports & Exercise 2001 July;33(7):1176-81. Not an exercise intervention study
Ref ID: 1906

(579) Beske SD, Alvarez GE, Ballard TP, Davy KP. Gender difference in cardiovagal baroreflex gain in humans. Journal of Applied Physiology 2001 November;91(5):2088-92. Study limited to adults
Ref ID: 1887

(580) Bessesen DH. Update on obesity. J Clin Endocrinol Metab 2008;93(6):2027-34. Review article
Ref ID: 3195

(581) Betancourt León H, Díaz ME. Análisis longitudinal de los indicadores peso-edad, talla-edad y peso–talla en adolescentes de la Escuela Nacional de Ballet de Cuba^ies. An venez nutr 2005;18(2):177-85. Cohort Study
Ref ID: 3997

(582) Betancourt León H, Aréchiga Viramontes J, Ramírez García CM, Díaz Sánchez ME. Determinación del peso corporal para la estatura de bailarines de ballet y danza moderna y folclórica de Cuba^ies. An venez nutr 2009;22(2):69-75. Cross-sectional study
Ref ID: 3998

(583) Betzig L. Means, variances, and ranges in reproductive success: comparative evidence. Evolution and Human Behavior 2012;33(4):309-17. Not an exercise intervention study
Ref ID: 5157

(584) Bevan JC, Veall GR, Macnab AJ, Ries CR, Marsland C. Midazolam premedication delays recovery after propofol without modifying involuntary movements. Anesthesia and analgesia 1997;85:50-4. Drug intervention study
Ref ID: 4691

(585) Beyrouty P, Chan HM. Co-consumption of selenium and vitamin E altered the reproductive and developmental toxicity of methylmercury in rats. Neurotoxicology and Teratology 2006;28(1):49-58. Animal study
Ref ID: 5158

(586) Bhandari N, Mazumder S, Bahl R, Martines J, Black RE, Bhan MK, -Infant-Feeding-Study-Group. An educational intervention to promote appropriate complementary feeding practices and physical growth in infants and young children in rural Haryana, India. The Journal of nutrition 2004;134:2342-8. Educational intervention, Diet Intervention or Supplement Study
Ref ID: 4692

(587) Bhandari N, Taneja S, Mazumder S, Bahl R, Fontaine O, Bhan MK, Zinc Study Group. Adding zinc to supplemental iron and folic acid does not affect mortality and severe morbidity in young children. Journal of Nutrition 2007 January;137(1):112-7. Diet Intervention or Supplement Study
Ref ID: 1289

(588) Bhugra D, Mastrogianni A, Maharajh H, Harvey S. Prevalence of bulimic behaviours and eating attitudes in schoolgirls from Trinidad and Barbados. Transcultural Psychiatry 2003 September;40(3):409-28. Not an exercise intervention study
Ref ID: 1693

(589) Bhutta ZA, Nizami SQ, Isani Z. Zinc supplementation in malnourished children with persistent diarrhea in Pakistan. Pediatrics 1999;103:e42. Diet Intervention or Supplement Study
Ref ID: 4693

(590) Biassio LG, Matsudo SMM, Matsudo VKR. Impacto da menarca nas variáveis antropométricas e neuromotoras da aptidão física, analisado longitudinalmente^ipt. Rev bras ciênc mov 2004;12(2):97-101. Not an exercise intervention study
Ref ID: 3999

(591) Biban P, Zangardi T, Baraldi E, Dussini N, Chiandetti L, Zacchello F. Mixed exhaled nitric oxide and plasma nitrites and nitrates ire newborn infants. Life Sciences 2001;68(25):2789-97. Subjects less than 2 years old
Ref ID: 5159

(592) Biddle MG, Vincent G, McCambridge A, Britton G, Dewes O, Elley CR, Moyes SA, Edge J. Randomised controlled trial of informal team sports for cardiorespiratory fitness and health benefit in Pacific adults. Journal of Primary Health Care 2011 December;3(4):269-77. Study limited to adults
Ref ID: 2418

(593) Biddle SJ, Gorely T, Stensel DJ. Health-enhancing physical activity and sedentary behaviour in children and adolescents. [Review] [146 refs]. Journal of Sports Sciences 2004 August;22(8):679-701. Review article
Ref ID: 1588

(594) Bielen E, Fagard R, Amery A. Inheritance of heart structure and physical exercise capacity: a study of left ventricular structure and exercise capacity in 7-year-old twins. Eur Heart J 1990 January;11(1):7-16. Not an exercise intervention study
Ref ID: 2316

(595) Bier ID, Wilson J, Studt P, Shakleton M. Auricular Acupuncture, Education, and Smoking Cessation: A Randomized, Sham-Controlled Trial. American Journal of Public Health 2002 October;92(10):1642-7. Not an exercise intervention study
Ref ID: 3804

(596) Bilaceroglu S, Perim K, Buyuksirin M, Celikten E. Prednisolone: a beneficial and safe adjunct to antituberculosis treatment? A randomized controlled trial. International Journal of Tuberculosis & Lung Disease 1999 January;3(1):47-54. Drug intervention study
Ref ID: 2043

(597) Bilger M, Speraw S, LaFranchi SH, Hanna CE. Androgen replacement in adolescents and young women with hypopituitarism. Journal of Pediatric Endocrinology 2005 April;18(4):355-62. Drug intervention study
Ref ID: 1523

(598) Billoo AG, Murtaza G, Memon MA, Khaskheli SA, Iqbal K, Rao MH. Comparison of oral versus injectable vitamin-D for the treatment of nutritional vitamin-D deficiency rickets. Journal of the College of Physicians and Surgeons Pakistan 2009;19:428-31. Diet Intervention or Supplement Study
Ref ID: 4694

(599) Binesh MT, Adeli K. Pharmacological management of metabolic syndrome and its lipid complications. DARU 2010;18(3):146-54. Drug intervention study
Ref ID: 3531

(600) Binks M, van MT. Utilization patterns and user characteristics of an ad libitum Internet weight loss program. Journal of Medical Internet Research 2010;12(1):e9. Diet Intervention Study
Ref ID: 561

(601) Bird SR, Wiles J, Robbins J. The effect of sodium bicarbonate ingestion on 1500-m racing time. Journal of Sports Sciences 1995 October;13(5):399-403. Diet Intervention or Supplement Study
Ref ID: 2192

(602) Birketvedt GS, Thom E, Bernersen B, Florholmen J. Combination of diet, exercise and intermittent treatment of cimetidine on body weight and maintenance of weight loss. A 42 months follow-up study. Med Sci Monit 2000 July;6(4):699-703. Study limited to adults
Ref ID: 400

(603) Birks EJ, Tansley PD, Hardy J, George RS, Bowles CT, Burke M, Banner NR, Khaghani A, Yacoub MH. Left ventricular assist device and drug therapy for the reversal of heart failure. New England Journal of Medicine 2006 November 2;355(18):1873-84. Drug intervention study
Ref ID: 1334

(604) Bischoff SC, Damms-Machado A, Betz C, Herpertz S, Legenbauer T, Low T, Wechsler JG, Bischoff G, Austel A, Ellrott T. Multicenter evaluation of an interdisciplinary 52-week weight loss program for obesity with regard to body weight, comorbidities and quality of life--a prospective study. International Journal of Obesity 2012 April;36(4):614-24. Observational study
Ref ID: 2419

(605) Bishop C, Hudson VM, Hilton SC, Wilde C. A pilot study of the effect of inhaled buffered reduced glutathione on the clinical status of patients with cystic fibrosis. Chest 2005 January;127(1):308-17. Drug intervention study
Ref ID: 1551

(606) Bispo Júnior RZ, Kawano CT, Guedes AV. Chronic multiple knee ligament injuries: epidemiological analysis of more than one hundred cases^ien. Clinics 2008;63(1):3-8. Retrospective study
Ref ID: 4000

(607) Bissonnette B, Sessler DI. Mild Hypothermia Does Not Impair Postanesthetic Recovery in Infants and Children. Anesthesia and analgesia 1993;76(1):168-72. Not an exercise intervention study
Ref ID: 5160

(608) Bjelland M, Bergh IH, Grydeland M, Klepp KI, Andersen LF, Anderssen SA, Ommundsen Y, Lien N. Changes in adolescents' intake of sugar-sweetened beverages and sedentary behaviour: results at 8 month mid-way assessment of the HEIA study--a comprehensive, multi-component school-based randomized trial. International Journal of Behavioral Nutrition & Physical Activity 2011;8:63. Survey or questionnaire
Ref ID: 2420

(609) Bjork J, Albin M, Grahn P, Jacobsson H, Ardo J, Wadbro J, Ostergren PO. Recreational values of the natural environment in relation to neighbourhood satisfaction, physical activity, obesity and wellbeing. Journal of Epidemiology & Community Health 2008 April;62(4):e2. Survey or questionnaire
Ref ID: 981

(610) Black LE, Swan PD, Alvar BA. Effects of intensity and volume on insulin sensitivity during acute bouts of resistance training. Journal of Strength & Conditioning Research 2010 April;24(4):1109-16. Acute study
Ref ID: 555

(611) Black MA, Cable NT, Thijssen DHJ, Green DJ. Importance of measuring the time course of flow-mediated dilatation in humans. Hypertension 2008;51(2):203-10. Not a randomized controlled trial (RCT)
Ref ID: 5161

(612) Black MM, Dubowitz H, Hutcheson J, Berenson HJ, Starr RH. A randomized clinical trial of home intervention for children with failure to thrive. Pediatrics 1995;95:807-14. Subjects less than 2 years old
Ref ID: 4695

(613) Black MM, Hager ER, Le K, Anliker J, Arteaga SS, Diclemente C, Gittelsohn J, Magder L, Papas M, Snitker S, Treuth MS, Wang Y. Challenge! Health promotion/obesity prevention mentorship model among urban, black adolescents. Pediatrics 2010 August;126(2):280-8. No exercise only group
Ref ID: 28

(614) Blackwell PL. The influence of touch on child development: Implications for intervention. Infants and Young Children 2000;13(1):25-39. Review article
Ref ID: 5162

(615) Blair D, Buskirk ER. Habitual daily energy expenditure and activity levels of lean and adult-onset and child-onset obese women. American Journal of Clinical Nutrition 1987 March;45(3):540-50. Acute study
Ref ID: 2342

(616) Blair SN, Chandler JV, Ellisor DB, Langley T. Improving physical fitness by exercise training programs. Southern Medical Journal 1980 December;73(12):1594-6. Not a randomized controlled trial (RCT)
Ref ID: 2376

(617) Blanc S, Normand S, Pachiaudi C, Duvareille M, Gharib C. Leptin responses to physical inactivity induced by simulated weightlessness. American Journal of Physiology-Regulatory Integrative and Comparative Physiology 2000;279(3):R891-R898. Acute study
Ref ID: 5163

(618) Blevins T, Pullman J, Malloy J, Yan P, Taylor K, Schulteis C, Trautmann M, Porter L. DURATION-5: exenatide once weekly resulted in greater improvements in glycemic control compared with exenatide twice daily in patients with type 2 diabetes. Journal of Clinical Endocrinology & Metabolism 2011 May;96(5):1301-10. Drug intervention study
Ref ID: 2421

(619) Bleyenheuft Y, Thonnard JL. Grip Control in Children before, during, and after Impulsive Loading. Journal of Motor Behavior 2010;42(3):169-77. Not All Participants were Overweight and/or Obese
Ref ID: 5164

(620) Block KI, Block P, Gyllenhaal C. The Role of Optimal Healing Environments in Patients Undergoing Cancer Treatment: Clinical Research Protocol Guidelines. Journal of Alternative & Complementary Medicine 2004 October 2;10:S-157. Review article
Ref ID: 3532

(621) Bloom DE, Canning D, Shenoy ES. The effect of vaccination on children's physical and cognitive development in the Philippines. Applied Economics 2012;44(21):2777-83. Survey or questionnaire
Ref ID: 5165

(622) Bloomfield SA. Contributions of physical activity to bone health over the lifespan. Topics in Geriatric Rehabilitation 2005;21(1):68-76. Review article
Ref ID: 5166

(623) Boa-Sorte N, Neri LA, Leite ME, Brito SM, Meirelles AR, Luduvice FBS, Santos JP, Viveiros MR, Ribeiro-Júnior HC. Percepção materna e autopercepção do estado nutricional de crianças e adolescentes de escolas privadas^ipt. J pediatr (Rio J ) 2007 August;83(4):349-56. Cross-sectional study
Ref ID: 4001

(624) Bodinier M, Brossard C, Triballeau S, Morisset M, Guerin-Marchand C, Pineau F, de Coppet P, Moneret-Vautrin DA, Blank U, Denery-Papini S. Evaluation of an in vitro mast cell degranulation test in the context of food allergy to wheat. International Archives of Allergy and Immunology 2008;146(4):307-20. Not an exercise intervention study
Ref ID: 5167

(625) Boellner SW, Earl CQ, Arora S. Modafinil in children and adolescents with attention-deficit/hyperactivity disorder: a preliminary 8-week, open-label study. Curr Med Res Opin 2006;22:2457-65. Drug intervention study
Ref ID: 4696

(626) Boellner SW, Stark JG, Krishnan S, Zhang Y. Pharmacokinetics of lisdexamfetamine dimesylate and its active metabolite, d-amphetamine, with increasing oral doses of lisdexamfetamine dimesylate in children with attention-deficit/hyperactivity disorder: a single-dose, randomized, open-label, crossover study. Clinical Therapeutics 2010;32:252-64. Drug intervention study
Ref ID: 4697

(627) Boer JM, Ehnholm C, Menzel HJ, Havekes LM, Rosseneu M, O'Reilly DS, Tiret L. Interactions between lifestyle-related factors and the ApoE polymorphism on plasma lipids and apolipoproteins. The EARS Study. European Atherosclerosis Research Study. Arteriosclerosis, Thrombosis & Vascular Biology 1997 September;17(9):1675-81. Study limited to adults
Ref ID: 2123

(628) Bogarin R, Chanoine JP. Efficacy, safety and tolerability of orlistat, a lipase inhibitor, in the treatment of adolescent weight excess. Therapy 2009;6(1):23-30. Review article
Ref ID: 3196

(629) Bogin B, Varela-Silva MI. Leg length, proportion, health and beauty: a review. Anthropologischer Anzeiger 2009;67(4):439-59. Review article
Ref ID: 5168

(630) Bohdjalian A, Prager G, Rosak C, Weiner R, Jung R, Schramm M, Aviv R, Schindler K, Haddad W, Rosenthal N, Ludvik B. Improvement in glycemic control in morbidly obese type 2 diabetic subjects by gastric stimulation. Obesity Surgery 2009 September;19(9):1221-7. Not an exercise intervention study
Ref ID: 693

(631) Bohler T, Alex C, Becker E, Becker R, Hoffmann S, Hutzler D, Jung C, Laufersweiler-Lochmann F, Radu C. [Quality indicators for ambulatory health education programme for overweight and obese children and adolescents]. [German]. Gesundheitswesen 2004 November;66(11):748-53. Review article
Ref ID: 1569

(632) Bohórquez IM, Caballero S, Carrera L, Chávez R, Espinoza R, Flores L, Llanos M, Luna E, Vega J, Vera J, Salvatierra H, Pereyra H. Factores asociados a síntomas depresivos en trabajadoras sexuales^ies
Factors associated to depression symptomatology in sex workers^ien. An Fac Med (Perú) 2010 December;71(4):277-82. Cross-sectional study
Ref ID: 4002

(633) Boisseau N, Vermorel M, Rance M, Duche P, Patureau-Mirand P. Protein requirements in male adolescent soccer players. European Journal of Applied Physiology 2007;100(1):27-33. Not a randomized controlled trial (RCT)
Ref ID: 5169

(634) Boivin MJ, Busman RA, Parikh SM, Bangirana P, Page CF, Opoka RO, Giordani B. A pilot study of the neuropsychological benefits of computerized cognitive rehabilitation in Ugandan children with HIV. Neuropsychology 2010;24:667-73. Not an exercise intervention study
Ref ID: 4698

(635) Bolgla LA, Uhl TL. Electromyographic analysis of hip rehabilitation exercises in a group of healthy subjects. Journal of Orthopaedic & Sports Physical Therapy 2005 August;35(8):487-94. Study limited to adults
Ref ID: 1470

(636) Bolotova NV, Lazebnikova SV, Chicheva GV, Raigorodskaia NI. [The effectiveness of transcranial treatment using the AMO-ATOS-E apparatus for the correction of the reproductive system disorders in adolescent girls]. [Russian]. Voprosy Kurortologii, Fizioterapii i Lechebnoi Fizicheskoi Kultury (6):30-3, 2010 Nov-Dec 2010 November;(6):30-3. Not an exercise intervention study
Ref ID: 2422

(637) Bolster DR, Pikosky MA, McCarthy LM, Rodriguez NR. Exercise affects protein utilization in healthy children. Journal of Nutrition 2001 October;131(10):2659-63. Not a randomized controlled trial (RCT)
Ref ID: 1891

(638) Boman K, Hellsten G, Bruce A, Hallmans G, Nilsson TK. Endurance physical activity, diet and fibrinolysis. Atherosclerosis 1994 March;106(1):65-74. Study limited to adults
Ref ID: 2242

(639) Bond CM, Bonci LJ, Granger LR, Johnson CL, Malina RM, Milne LW, Ryan RA, Vanderbunt EM. National Athletic Trainers' Association Position Statement: Preventing, Detecting, and Managing Disordered Eating in Athletes. Journal of Athletic Training 2008 January;43(1):80-108. Review article
Ref ID: 3805

(640) Bond M, Wyatt K, Lloyd J, Welch K, Taylor R. Systematic review of the effectiveness and cost-effectiveness of weight management schemes for the under fives: a short report. [Review] [95 refs]. Health Technology Assessment (Winchester, England) 2009 December 20;13(61):1-75. Review article
Ref ID: 624

(641) Bondi M, Grugni G, Velardo A, Biella O, Venneri MG, Morabito F, Menozzi R, Del Rio G. Adrenomedullary response to caffeine in prepubertal and pubertal obese subjects. International Journal of Obesity 1999;23(9):992-6. Diet Intervention or Supplement Study
Ref ID: 5170

(642) Bondy CA. Congenital Cardiovascular Disease in Turner Syndrome. Congenital Heart Disease 2008 January;3(1):2-15. Review article
Ref ID: 3533

(643) Bonifati MD, Ruzza G, Bonometto P, Berardinelli A, Gorni K, Orcesi S, Lanzi G, Angelini C. A multicenter, double-blind, randomized trial of deflazacort versus prednisone in duchenne muscular dystrophy. Muscle Nerve 2000;23(9):1344-7. Drug intervention study
Ref ID: 3197

(644) Bonifazi M, Bela E, Carli G, Lodi L, Martelli G, Zhu B, Lupo C. Influence of training on the response of androgen plasma concentrations to exercise in swimmers. European Journal of Applied Physiology & Occupational Physiology 1995;70(2):109-14. Acute study
Ref ID: 2209

(645) Bonneau M. Factors affecting the level of androstenone. Acta Veterinaria Scandinavica 2006 January 2;48:S7-3. Animal study
Ref ID: 3534

(646) Bonnefoy XR, Braubach M, Moissonnier B, Monolbaev K, RÃ¶bbel N. Housing and Health in Europe: Preliminary Results of a Pan-European Study. American Journal of Public Health 93[9], 1559-1563. 2003. Survey or questionnaire,
Ref ID: 3535

(647) Bonofiglio D, Maggiolini M, Marsico S, Giorno A, Catalano S, Aquila S, Andò S. Critical years and stages of puberty for radial bone mass apposition during adolescence. Hormone and metabolic research = Hormon und Stoffwechselforschung = Hormones et métabolisme 1999;31:478-82. Cross-sectional study
Ref ID: 4699

(648) Boot AM, Nauta J, de Jong MCJW, Groothoff JW, Lilien MR, van Wijk JA, Kist-van Holthe JE, Hokken-Koelega S, Pols HAP, de Muinck Keizer-Schrama S, Boot. Bone mineral density, bone metabolism and body composition of children with chronic renal failure, with and without growth hormone treatment. Clinical Endocrinology 1998 November;49(5):665-72. Diet Intervention or Supplement Study
Ref ID: 548

(649) Booth CK, Coad RA, Forbes-Ewan CH, Thomson GF, Niro PJ. The physiological and psychological effects of combat ration feeding during a 12-day training exercise in the tropics. Military Medicine 2003 January;168(1):63-70. Diet Intervention Study
Ref ID: 1776

(650) Booth ML, Macaskill P, Lazarus R, Baur LA. Sociodemographic distribution of measures of body fatness among children and adolescents in New South Wales, Australia. Int J Obes Relat Metab Disord 1999 May;23(5):456-62. Not an exercise intervention study, Observational study
Ref ID: 409

(651) Bopp CM, Townsend DK, Barstow TJ. Characterizing near-infrared spectroscopy responses to forearm post-occlusive reactive hyperemia in healthy subjects. European Journal of Applied Physiology 2011 November;111(11):2753-61. Not an exercise intervention study
Ref ID: 2424

(652) Boreham C, Savage JM, Primrose D, Cran G, Strain J. Coronary risk factors in schoolchildren. Archives of Disease in Childhood 1993 February;68(2):182-6. Cross-sectional study
Ref ID: 2269

(653) Boreham CA, Kennedy RA, Murphy MH, Tully M, Wallace WF, Young I. Training effects of short bouts of stair climbing on cardiorespiratory fitness, blood lipids, and homocysteine in sedentary young women. British Journal of Sports Medicine 2005 September;39(9):590-3. Not All Participants were Overweight and/or Obese
Ref ID: 1481

(654) Borges AF, Borin JP, De Marco A. Avaliação de indicadores antropométricos e neuromusculares de jovens escolares do ensino fundamental do interior paulista^ipt. Motriz rev educ fís (Impr ) 2010 June;16(2):326-37. Not a randomized controlled trial (RCT)
Ref ID: 4003

(655) Borges CR, Kõhler MLK, Leite MdL, Silva ABF, Camargo ATd, Kanunfre CC. Influência da televisão na prevalência de obesidade infantil em Ponta Grossa, Paraná^ipt. Ciênc cuid saúde 2007 September;6(3):305-11. Cross-sectional study
Ref ID: 4004

(656) Borggraefe I, Schaefer JS, Klaiber M, Dabrowski E, Ammann-Reiffer C, Knecht B, Berweck S, Heinen F, Meyer-Heim A. Robotic-assisted treadmill therapy improves walking and standing performance in children and adolescents with cerebral palsy. European Journal of Paediatric Neurology 2010 November;14(6):496-502. Not a randomized controlled trial (RCT)
Ref ID: 2425

(657) Borovsky J, Kersz MJ, Kuper E. Valor de un examen médico de ingreso a una institución deportiva. Rev Argent Med Deporte 1996;18(61):106-12. Not an exercise intervention study
Ref ID: 792

(658) Borradaile KE, Foster GD, May H, Karpyn A, Sherman S, Grundy K, Nachmani J, Vander VS, Boruch RF. Associations between the Youth/Adolescent Questionnaire, the Youth/Adolescent Activity Questionnaire, and body mass index z score in low-income inner-city fourth through sixth grade children. American Journal of Clinical Nutrition 2008 June;87(6):1650-5. Cross-sectional study
Ref ID: 940

(659) Borrelli A, Mattiazzi L, Capucchio MT, Biolatti C, Cagnasso A, Gianella P, D'Angelo A. Cachexia secondary to intracranial anaplastic (malignant) ependymoma in a boxer dog. Journal of Small Animal Practice 2009;50(10):554-7. Animal study
Ref ID: 5171

(660) Borsheim E, Kien CL, Pearl WM. Differential effects of dietary intake of palmitic acid and oleic acid on oxygen consumption during and after exercise. Metabolism: Clinical & Experimental 2006 September;55(9):1215-21. Diet Intervention or Supplement Study
Ref ID: 1358

(661) Bortman M. Factores de riesgo de bajo peso al nacer^ies. Rev panam salud pública 1998 May;3(5):314-21. Cross-sectional study
Ref ID: 601

(662) Bortsov AV, Liese AD, Bell RA, Dabelea D, D'Agostino RB, Jr., Hamman RF, Klingensmith GJ, Lawrence JM, Maahs DM, McKeown R, Marcovina SM, Thomas J, Williams DE, Mayer-Davis EJ. Sugar-sweetened and diet beverage consumption is associated with cardiovascular risk factor profile in youth with type 1 diabetes. Acta Diabetologica 2011 December;48(4):275-82. Cross-sectional study
Ref ID: 2426

(663) Bosa VL, Mello EDd, Mocelin HT, Benedetti FJ, Fischer GB. Avaliação do estado nutricional de crianças e adolescentes com bronquiolite obliterante pós-infecciosa^ipt
Assessment of nutritional status in children and adolescents with post-infectious bronchiolitis obliterans^ien. J pediatr (Rio J ) 2008 August;84(4):323-30. Cross-sectional study
Ref ID: 4005

(664) Boschi V, Siervo M, Nasti G, Trapanese E, D'Orsi P, Augelli E, Papa A, Margiotta N, Bellini O, Falconi C. Interdisciplinary treatment of a female outpatient population. Organizational model and preliminary results. Eating & Weight Disorders: EWD 2002 December;7(4):268-75. Diet Intervention Study
Ref ID: 1785

(665) Botvin GJ, Cantlon A, Carter BJ, Williams CL. Reducing adolescent obesity through a school health program. Journal of Pediatrics 1979 December;95(6):1060-3. Lifestyle Intervention
Ref ID: 2381

(666) Bouhlel E, Denguezli M, Zaouali M, Tabka Z, Shephard RJ. Ramadan fastings effect on plasma leptin, adiponectin concentrations, and body composition in trained young men. International Journal of Sport Nutrition & Exercise Metabolism 2008 December;18(6):617-27. Study limited to adults
Ref ID: 830

(667) Bourgeois JM, Nagel K, Pearce E, Wright M, Barr RD, Tarnopolsky MA. Creatine monohydrate attenuates body fat accumulation in children with acute lymphoblastic leukemia during maintenance chemotherapy. Pediatric Blood & Cancer 2008;51(2):183-7. Diet Intervention or Supplement Study
Ref ID: 5172

(668) Bourgois J, Vrijens J. The Conconi test: a controversial concept for the determination of the anaerobic threshold in young rowers. International Journal of Sports Medicine 1998 November;19(8):553-9. Not a randomized controlled trial (RCT)
Ref ID: 2072

(669) Boury JM, Larkin KT, Krummel DA. Factors related to postpartum depressive symptoms in low-income women. Women & Health 2004;39(3):19-34. Study limited to adults
Ref ID: 1607

(670) Boutelle KN, Cafri G, Crow SJ. Parent-only treatment for childhood obesity: a randomized controlled trial. Obesity 2011 March;19(3):574-80. Lifestyle Intervention
Ref ID: 2427

(671) Bouten CVC, Koekkoek KTM, Verduin M, Kodde R, Janssen JD. A triaxial accelerometer and portable data processing unit for the assessment of daily physical activity. Ieee Transactions on Biomedical Engineering 1997;44(3):136-47. Not an exercise intervention study
Ref ID: 5173

(672) Bouza E, MuÃ±oz P. Linezolid: pharmacokinetic characteristics and clinical studies. Clinical Microbiology & Infection 2001 August 3;7:75-82. Drug intervention study
Ref ID: 3536

(673) Bowden RG, Lanning BA, Doyle EI, Slonaker B, Johnston HM, Scanes G. Systemic glucose level changes with a carbohydrate-restricted and higher protein diet combined with exercise. Journal of American College Health 2007 September;56(2):147-52. Diet Intervention Study
Ref ID: 1146

(674) Bower M, Collins S, Cottrill C, Cwynarski K, Montoto S, Nelson M, Nwokolo N, Powles T, Stebbing J, Wales N, Webb A. British HIV Association guidelines for HIV-associated malignancies 2008. HIV Medicine 2008 July;9(6):336-88. Review article
Ref ID: 3537

(675) Bowling FG, Munce TB. Abnormal protein glycoforms in Prader-Willi syndrome. Journal of Intellectual Disability Research 2008 October;52(10):812. Not an exercise intervention study
Ref ID: 3806

(676) Boyd RN, Dobson F, Parrott J, Love S, Oates J, Larson A, Burchall G, Chondros P, Carlin J, Nattrass G, Graham HK. The effect of botulinum toxin type A and a variable hip abduction orthosis on gross motor function: a randomized controlled trial. European Journal of Neurology 2001 November;8:Suppl-19. Drug intervention study
Ref ID: 1863

(677) Boyle CA. Surveillance of developmental disabilities with an emphasis on special studies. Reprod Toxicol 1997;11(2-3):271-4. Not an exercise intervention study
Ref ID: 5174

(678) Boyle MH, Willms JD. Multilevel Modelling of Hierarchical Data in Developmental Studies. Journal of Child Psychology & Psychiatry & Allied Disciplines 2001 January;42(1):141. Not an exercise intervention study
Ref ID: 496

(679) Boynton JR, Thomas TN, Peterson KE, Wiecha J, Sobol AM, Gortmaker SL. Impact of television viewing patterns on fruit and vegetable consumption among adolescents. Pediatrics in review / American Academy of Pediatrics 2003;112:1321-6. Prospective Study
Ref ID: 4700

(680) Bozza R, Stabelini Neto A, Ulbrich AZ, Vasconcelos ÍQAd, Mascarenhas LPG, Brito LMS, Campos Wd. Circunferência da cintura, índice de massa corporal e fatores de risco cardiovascular na adolescência^ipt
Waist circumference, body mass index and cardiovascular risk factors in adolescence^ien. Rev bras cineantropom desempenho hum 2009;11(3):286-91. Cross-sectional study
Ref ID: 4006

(681) Braam W, Smits MG, Didden R, Curfs LMG. Melatonin is effective in treating sleep problems in Angelman syndrome but problems in metabolising melatonin may be part of the Angelman phenotype. Journal of Intellectual Disability Research 2008 October;52(10):814. Diet Intervention or Supplement Study
Ref ID: 3807

(682) Braamskamp MJ, Wijburg FA, Wiegman A. Drug therapy of hypercholesterolaemia in children and adolescents. Drugs 2012 April 16;72(6):759-72. Review article
Ref ID: 2428

(683) Bracco MM, Colugnati FAB, Pratt M, Taddei JAAC. Multivariate hierarchical model for physical inactivity among public school children^ien. J pediatr (Rio J ) 2006 August;82(4):302-7. Survey or questionnaire
Ref ID: 4007

(684) Bracken RM, Linnane DM, Brooks S. Alkalosis and the plasma catecholamine response to high-intensity exercise in man. Medicine & Science in Sports & Exercise 2005 February;37(2):227-33. Acute study
Ref ID: 1547

(685) Bracko MR, George JD. Prediction of ice skating performance with off-ice testing in women's ice hockey players. Journal of Strength & Conditioning Research 2001 February;15(1):116-22. Cross-sectional study
Ref ID: 1879

(686) Bracko MR. On-ice performance characteristics of elite and non-elite women's ice hockey players. Journal of Strength & Conditioning Research 2001 February;15(1):42-7. Not an exercise intervention study
Ref ID: 1880

(687) Bradley RH, McRitchie S, Houts RM, Nader P, O'Brien M, NICHD Early Child Care Research Network. Parenting and the decline of physical activity from age 9 to 15. International Journal of Behavioral Nutrition & Physical Activity 2011;8:33. Cross-sectional study
Ref ID: 2429

(688) Bradney M, Pearce G, Naughton G, Sullivan C, Bass S, Beck T, Carlson J, Seeman E. Moderate exercise during growth in prepubertal boys: changes in bone mass, size, volumetric density, and bone strength: a controlled prospective study. Journal of bone and mineral research : the official journal of the American Society for Bone and Mineral Research 1998;13:1814-21. Not All Participants were Overweight and/or Obese
Ref ID: 4701

(689) Bradshaw B. The role of the family in managing therapy in minority children with type 2 diabetes mellitus. Journal of Pediatric Endocrinology 2002 April;15:Suppl-51. Not an exercise intervention study
Ref ID: 1825

(690) Brady ML, Allan AM, Caldwell KK. A Limited Access Mouse Model of Prenatal Alcohol Exposure that Produces Long-Lasting Deficits in Hippocampal-Dependent Learning and Memory. Alcoholism-Clinical and Experimental Research 2012;36(3):457-66. Animal study
Ref ID: 5175

(691) Braga PD, Molina MdCB, Cade NV. Expectativas de adolescentes em relação a mudanças do perfil nutricional^ipt. Ciênc saúde coletiva 2007 October;12(5):1221-8. Cross-sectional study
Ref ID: 4008

(692) Braham R, Finch CF, McIntosh A, McCrory P. Community level Australian Football: a profile of injuries. Journal of Science & Medicine in Sport 2004 March;7(1):96-105. Not an exercise intervention study
Ref ID: 1631

(693) Branca F, Popkin BM, Simopoulos AP. Preface by guest editors. International Journal of Obesity 2008 November;32:Suppl-3. Editorial or letter or comment
Ref ID: 858

(694) Brandäo CMA, Lombardi MT, Nishida SK, Hauache OM, Vieira JGH. Serum leptin concentration during puberty in healthy nonobese adolescents. Braz j med biol res 2003 October;36(10):1293-6. Not All Participants were Overweight and/or Obese
Ref ID: 724

(695) Brandes JM, Itskovitz J, Scher A, Gershonibaruch R. The Physical and Mental-Development of Co-Sibs Surviving Selective Reduction of Multifetal Pregnancies. Human Reproduction 1990;5(8):1014-7. Not an exercise intervention study
Ref ID: 5177

(696) Brandes JM, Scher A, Itzkovits J, Thaler I, Sarid M, Gershoni BR. Growth and development of children conceived by in vitro fertilization. Pediatrics 1992;90:424-9. Not an exercise intervention study
Ref ID: 1055

(697) Brandes M. The importance of physical activity and fitness for human health. Bundesgesundheitsblatt-Gesundheitsforschung-Gesundheitsschutz 2012;55(1):96-101. Review article
Ref ID: 5178

(698) Brandou F, Dumortier M, Garandeau P, Mercier J, Brun JF. Effects of a two-month rehabilitation program on substrate utilization during exercise in obese adolescents. Diabetes & Metabolism 2003 February;29(1):20-7. Not a randomized controlled trial (RCT)
Ref ID: 1767

(699) Brandou F, Savy-Pacaux AM, Marie J, Bauloz M, Maret-Fleuret I, Borrocoso S, Mercier J, Brun JF. Impact of high- and low-intensity targeted exercise training on the type of substrate utilization in obese boys submitted to a hypocaloric diet. Diabetes & Metabolism 2005 September;31(4:Pt 1):t-35. Diet Intervention Study
Ref ID: 1439

(700) Brandstetter S, Klenk J, Berg S, Galm C, Fritz M, Peter R, Prokopchuk D, Steiner RP, Wartha O, Steinacker J, Wabitsch M. Overweight prevention implemented by primary school teachers: a randomised controlled trial. Obesity Facts 2012;5(1):1-11. Not All Participants were Overweight and/or Obese
Ref ID: 2430

(701) Branger B, Cadudal JL, Delobel M, Ouoba H, Yameogo P, Ouedraogo D, Guerin D, Valea A, Zombre C, Ancel P, personnels-des CREN. [Spiruline as a food supplement in case of infant malnutrition in Burkina-Faso]. Archives de pédiatrie : organe officiel de la Sociéte française de pédiatrie 2003;10:424-31. Diet Intervention or Supplement Study
Ref ID: 4702

(702) Branski LK, Herndon DN, Barrow RE, Kulp GA, Klein GL, Suman OE, Przkora R, Meyer W, Huang T, Lee JO, Chinkes DL, Mlcak RP, Jeschke MG. Randomized controlled trial to determine the efficacy of long-term growth hormone treatment in severely burned children. Ann Surg 2009;250(4):514-22. Drug intervention study
Ref ID: 3199

(703) Bratland-Sanda S, Rosenvinge JH, Vrabel KA, Norring C, Sundgot-Borgen J, Ro O, Martinsen EW. Physical activity in treatment units for eating disorders: clinical practice and attitudes. Eating & Weight Disorders: EWD 2009 June;14(2-3):e106-e112. Survey or questionnaire
Ref ID: 640

(704) Braun B, Gerson L, Hagobian T, Grow D, Chipkin SR. No effect of short-term testosterone manipulation on exercise substrate metabolism in men. Journal of Applied Physiology 2005 November;99(5):1930-7. Study limited to adults
Ref ID: 1462

(705) Bravender T, Russell A, Chung RJ, Armstrong SC. A "novel" intervention: a pilot study of children's literature and healthy lifestyles. Pediatrics 2010 March;125(3):e513-e517. Not an exercise intervention study
Ref ID: 60

(706) Breman JG, Holloway CN. Malaria surveillance counts. American Journal of Tropical Medicine and Hygiene 2007;77(6):36-47. Not an exercise intervention study
Ref ID: 5179

(707) Brennan L, Walkley J, Fraser SF, Greenway K, Wilks R. Motivational interviewing and cognitive behaviour therapy in the treatment of adolescent overweight and obesity: study design and methodology. Contemporary Clinical Trials 2008 May;29(3):359-75. Description of study from review or magazine or etc. (not the actual study)
Ref ID: 968

(708) Brent RL, Tanski S, Weitzman M. A pediatric perspective on the unique vulnerability and resilience of the embryo and the child to environmental toxicants: The importance of rigorous research concerning age and agent. Pediatrics 2004;113(4):935-44. Not an exercise intervention study
Ref ID: 5180

(709) Brentano MA, Cadore EL, da Silva EM, Ambrosini AB, Coertjens M, Petkowicz R, Viero I, Kruel LF. Physiological adaptations to strength and circuit training in postmenopausal women with bone loss. Journal of Strength & Conditioning Research 2008 November;22(6):1816-25. Study limited to adults
Ref ID: 864

(710) Briancon S, Bonsergent E, Agrinier N, Tessier S, Legrand K, Lecomte E, Aptel E, Hercberg S, Collin JF, PRALIMAP Trial Group. PRALIMAP: study protocol for a high school-based, factorial cluster randomised interventional trial of three overweight and obesity prevention strategies. Trials [Electronic Resource] 2010;11:119. Lifestyle Intervention
Ref ID: 2431

(711) Briceño Y, Chirinos J, Paoli M, Zerpa Y. Panhipopituitarismo secundario a macroadenoma hipofisario no funcionante en la adolescencia^ies. Rev venez endocrinol metab 2009 February;7(1):35-40. Case-Control / Case Study
Ref ID: 4009

(712) Bricout VA, Guinot M, Faure P, Flore P, Eberhard Y, Garnier P, Favre Juvin A. Are Hormonal Responses to Exercise in Young Men with Downâ€™s Syndrome Related to Reduced Endurance Performance? Journal of Neuroendocrinology 2008 May;20(5):558-65. Not an exercise intervention study
Ref ID: 3538

(713) Brilla LR, Haley TF. Effect of magnesium supplementation on strength training in humans. Journal of the American College of Nutrition 1992 June;11(3):326-9. Diet Intervention or Supplement Study
Ref ID: 2288

(714) Brinkworth GD, Buckley JD, Slavotinek JP, Kurmis AP. Effect of bovine colostrum supplementation on the composition of resistance trained and untrained limbs in healthy young men. European Journal of Applied Physiology 2004 January;91(1):53-60. Diet Intervention or Supplement Study
Ref ID: 1674

(715) Brisch KH, Buchheim A, Kohntop B, Kunzke D, Schmucker G, Kachele H, Pohlandt F. Early preventive psychotherapeutic intervention for parents of a premature infant with very low birth weight: The Ulm Study. <ORIGINAL> PRAVENTIVES PSYCHOTHERAPEUTISCHES INTERVENTIONSPROGRAMM FUR ELTERN NACH DER GEBURT EINES SEHR KLEINEN FRUHGEBORENEN - ULMER MODELL. RANDOMISIERTE LANGSSCHNITTSTUDIE. Monatsschrift Fur Kinderheilkunde 1996;144:1206-12. Not an exercise intervention study
Ref ID: 4703

(716) Brisch KH, Bechinger D, Betzler S, Heinemann H. Early preventive attachment-oriented psychotherapeutic intervention program with parents of a very low birthweight premature infant: results of attachment and neurological development. Attachment & human development 2003;5:120-35. Subjects less than 2 years old
Ref ID: 4704

(717) Brismar T, Maurex L, Cooray G, Juntti-Berggren L, Lindstrom P, Ekberg K, Adner N, Andersson S. Predictors of cognitive impairment in type 1 diabetes. Psychoneuroendocrinology 2007;32(8-10):1041-51. Cross-sectional study
Ref ID: 5181

(718) Brito EC, Vimaleswaran KS, Brage S, Andersen LB, Sardinha LB, Wareham NJ, Ekelund U, Loos RJ, Franks PW. PPARGC1A sequence variation and cardiovascular risk-factor levels: a study of the main genetic effects and gene x environment interactions in children from the European Youth Heart Study. Diabetologia 2009 April;52(4):609-13. Cross-sectional study
Ref ID: 793

(719) Brockmann V, Caussade L, Holmgren P, Prado A, Reyes M, Viviani G, Bertrand N. Actividad física y obesidad en niños con asma^ies. Rev chil pediatr 2007 October;78(5):482-8. Not an exercise intervention study
Ref ID: 4010

(720) Brodersen NH, Steptoe A, Boniface DR, Wardle J. Trends in physical activity and sedentary behaviour in adolescence: ethnic and socioeconomic differences. British Journal of Sports Medicine 2007 March;41(3):140-4. Cohort Study
Ref ID: 1265

(721) Broeder CE, Burrhus KA, Svanevik LS, Wilmore JH. The effects of either high-intensity resistance or endurance training on resting metabolic rate. American Journal of Clinical Nutrition 1992 April;55(4):802-10. Study limited to adults
Ref ID: 2290

(722) Brondel L, Romer MA, Nougues PM, Touyarou P, Davenne D. Acute partial sleep deprivation increases food intake in healthy men. Am J Clin Nutr 2010 June;91(6):1550-9. Not an exercise intervention study
Ref ID: 52

(723) Bronhara B, Vieira VCR. Proporcionalidade corporal na avaliação antropométrica de adolescentes pós-menarca^ipt. Rev nutr 2007 February;20(1):27-37. Cross-sectional study
Ref ID: 4011

(724) Bronner YL, Paige DM. Current Concepts in Infant Nutrition. Journal of Nurse-Midwifery 1992;37(2):S43-S58. Review article
Ref ID: 5182

(725) Brosnan M, Walker I. A Preliminary Investigation into the Potential Role of Waist Hip Ratio (WHR) Preference within the Assortative Mating Hypothesis of Autistic Spectrum Disorders. Journal of Autism & Developmental Disorders 2009 January;39(1):164-71. Not an exercise intervention study
Ref ID: 3808

(726) Brotman LM, Dawson-McClure S, Huang KY, Theise R, Kamboukos D, Wang J, Petkova E, Ogedegbe G. Early childhood family intervention and long-term obesity prevention among high-risk minority youth. Pediatrics 2012 March;129(3):e621-e628. Not an exercise intervention study
Ref ID: 2432

(727) Brown BD, Harris KJ, Harris JL, Parker M, Ricci C, Noonan C. Translating the diabetes prevention program for Northern Plains Indian youth through community-based participatory research methods. Diabetes Educator 2010 November;36(6):924-35. Not an exercise intervention study
Ref ID: 1084

(728) Brown KH, López-de RD, Arsenault JE, Peerson JM, Penny ME. Comparison of the effects of zinc delivered in a fortified food or a liquid supplement on the growth, morbidity, and plasma zinc concentrations of young Peruvian children. The American journal of clinical nutrition 2007;85:538-47. Diet Intervention or Supplement Study
Ref ID: 4705

(729) Brown LD, Heermann JA. The effect of developmental care on preterm infant outcome. Applied nursing research : ANR 1997;10:190-7. Retrospective study
Ref ID: 952

(730) Brown NA, Jensen JL. The development of contact force construction in the dynamic-contact task of cycling [corrected].[Erratum appears in J Biomech. 2003 Apr;36(4):619]. Journal of Biomechanics 2003 January;36(1):1-8. Not a randomized controlled trial (RCT)
Ref ID: 1781

(731) Brown T, Avenell A, Edmunds LD, Moore H, Whittaker V, Avery L, Summerbell C. Systematic review of long-term lifestyle interventions to prevent weight gain and morbidity in adults. [Review] [68 refs]. Obes Rev 2009 November;10(6):627-38. Review article
Ref ID: 652

(732) Brownell KD, Kelman JH, Stunkard AJ. Treatment of obese children with and without their mothers: changes in weight and blood pressure. Pediatrics 1983 April;71(4):515-23. Lifestyle Intervention
Ref ID: 2367

(733) Brownson RC, Chriqui JF, Burgeson CR, Fisher MC, Ness RB. Translating Epidemiology Into Policy to Prevent Childhood Obesity: The Case for Promoting Physical Activity in School Settings. Annals of Epidemiology 2010;20(6):436-44. Review article
Ref ID: 5183

(734) Brunstrom JM, Mitchell GL. Flavor-nutrient learning in restrained and unrestrained eaters. Physiology & Behavior 2007;90(1):133-41. Diet Intervention or Supplement Study
Ref ID: 5184

(735) Brutsaert TD, Spielvogel H, Soria R, Caceres E, Buzenet G, Haas JD. Effect of developmental and ancestral high-altitude exposure on VO(2)peak of Andean and European/North American natives. American Journal of Physical Anthropology 1999;110(4):435-55. Not an exercise intervention study
Ref ID: 5185

(736) Bryner RW, Toffle RC, Ullrich IH, Yeater RA. The effects of exercise intensity on body composition, weight loss, and dietary composition in women. Journal of the American College of Nutrition 1997 February;16(1):68-73. Study limited to adults
Ref ID: 2147

(737) Brzycki M. Pills, Powders & Potions. Coach & Athletic Director 2007 March;76(8):63-5. Review article
Ref ID: 3809

(738) Buchan DS, Ollis S, Thomas NE, Baker JS. The influence of a high intensity physical activity intervention on a selection of health related outcomes: an ecological approach. BMC Public Health 2010;10:8. Description of study from review or magazine or etc. (not the actual study)
Ref ID: 575

(739) Buchan DS, Ollis S, Thomas NE, Buchanan N, Cooper SM, Malina RM, Baker JS. Physical activity interventions: effects of duration and intensity. Scandinavian Journal of Medicine & Science in Sports 2011 December;21(6):e341-e350. Not All Participants were Overweight and/or Obese
Ref ID: 2434

(740) BUCHHEIT MART, HOROBEANU COSM, MENDEZ-VILLANUEVA ALBE, SIMPSON BM, BOURDON PC. Effects of age and spa treatment on match running performance over two consecutive games in highly trained young soccer players. Journal of Sports Sciences 2011 March 15;29(6):591-8. Acute study
Ref ID: 3885

(741) Buckley JD, Brinkworth GD, Abbott MJ. Effect of bovine colostrum on anaerobic exercise performance and plasma insulin-like growth factor I. Journal of Sports Sciences 2003 July;21(7):577-88. Diet Intervention or Supplement Study
Ref ID: 1734

(742) Buckley JM, Souhrada JF. A comparison of pulmonary function tests in detecting exercise-induced bronchoconstriction. Pediatrics 1975 November;56(5:pt-2 suppl):t-2. Acute study
Ref ID: 2392

(743) Bueno N, Fletcher BJ, Fletcher GF, Serra S, Cruz PM, Kelly D, Meirelles L, Atkinson E, Tabor LA, Ramos A, Castro I. Coronary risk factors in adult children of parents with coronary heart disease: a comparison survey of southeastern Brazil and southeastern United States. Preventive Cardiology 2005;8(3):149-54. Survey or questionnaire
Ref ID: 1493

(744) Bueno O, Bueno G, Moreno LA, Nuviala RJ, PÃ©rez-GonzÃ¡lez JM, Bueno M. Zinc supplementation in infants with asymmetric intra uterine growth retardation; effect on growth, nutritional status and leptin secretion. Nutricion Hospitalaria 2008 May;23(3):212-9. Diet Intervention or Supplement Study
Ref ID: 3539

(745) Bueno VC, Lombardi Júnior I, Medeiros WM, Azevedo MMA, Len CA, Terreri MT, Natour J, Hilário MOE. Reabilitação em artrite idiopática juvenil^ipt. Rev bras reumatol 2007 June;47(3):197-203. Review article
Ref ID: 4012

(746) Buff CdG, Ramos E, Souza FI, Sarni RO. Freqüência de síndrome metabólica em crianças e adolescentes com sobrepeso e obesidade^ipt
Frequency of metabolic syndrome in overweight and obese children and adolescents^ien. Rev paul pediatr 2007 September;25(3):221-6. Cross-sectional study
Ref ID: 4013

(747) Buffart LM, van den Berg-Emons RJ, van Wijlen-Hempel MS, Stam HJ, Roebroeck ME. Health-related physical fitness of adolescents and young adults with myelomeningocele. European Journal of Applied Physiology 2008 May;103(2):181-8. Cross-sectional study
Ref ID: 960

(748) Buhring B, Oliva M, Bravo C. Determinación no experimental de la conducta sedentaria en escolares^ies. Rev chil nutr 2009 March;36(1):23-30. Review article
Ref ID: 4014

(749) Bullock N, Comfort P. An investigation into the acute effects of depth jumps on maximal strength performance. Journal of Strength & Conditioning Research 2011 November;25(11):3137-41. Acute study
Ref ID: 2435

(750) Bundy AC, Naughton G, Tranter P, Wyver S, Baur L, Schiller W, Bauman A, Engelen L, Ragen J, Luckett T, Niehues A, Stewart G, Jessup G, Brentnall J. The Sydney playground project: popping the bubblewrap--unleashing the power of play: a cluster randomized controlled trial of a primary school playground-based intervention aiming to increase children's physical activity and social skills. BMC Public Health 2011;11:680. Description of study from review or magazine or etc. (not the actual study)
Ref ID: 2436

(751) Buntain HM, Greer RM, Schluter PJ, Wong JC, Batch JA, Potter JM, Lewindon PJ, Powell E, Wainwright CE, Bell SC. Bone mineral density in Australian children, adolescents and adults with cystic fibrosis: a controlled cross sectional study. Thorax 2004 February;59(2):149-55. Cross-sectional study
Ref ID: 1669

(752) Buonani C, Fernandes RA, Silveira LS, Bastos KdN, Monteiro PA, Viotto Filho I, Júnior F. Prevenção da síndrome metabólica em crianças obesas: uma proposta de intervenção^ipt
Prevention of metabolic syndrome in obese children: a proposal of intervention^ien. Rev paul pediatr 2011 June;29(2):186-92. Not a randomized controlled trial (RCT)
Ref ID: 4015

(753) Burbano JC, Fornasini M, Acosta M. Fe de errata: prevalencia y factores de riesgo de sobrepeso en colegialas de 12 a 19 años en una región semiurbana del Ecuador. Rev panam salud pública 2003 August;14(2):96. Cross-sectional study
Ref ID: 4016

(754) Burbano JC, Fornasini M, Acosta M. Prevalencia y factores de riesgo de sobrepeso en colegialas de 12 a 19 años en una región semiurbana del Ecuador. Rev panam salud pública 2003 May;13(5):277-84. Cross-sectional study
Ref ID: 4017

(755) Burdette HL, Wadden TA, Whitaker RC. Neighborhood safety, collective efficacy, and obesity in women with young children. Obesity 2006;14(3):518-25. Cross-sectional study
Ref ID: 5186

(756) Burgert TS, Duran EJ, Goldberg-Gell R, Dziura J, Yeckel CW, Katz S, Tamborlane WV, Caprio S. Short-term metabolic and cardiovascular effects of metformin in markedly obese adolescents with normal glucose tolerance. Pediatr Diabetes 2008 December;9(6):567-76. No exercise only group, Drug intervention study
Ref ID: 159

(757) Burgos MS, Reuter CP, Burgos LT, Pohl HH, Pauli LTS, Horta JA, Reckziegel MB, Franke SIR, Prá D, Camargo M. Uma análise entre índices pressóricos, obesidade e capacidade cardiorrespiratória em escolares^ipt
Comparison analysis of blood pressure, obesity, and cardio-respiratory fitness in schoolchildren^ien. Arq bras cardiol 2010 June;94(6):788-93. Cross-sectional study
Ref ID: 4018

(758) Burk A, Timpmann S, Medijainen L, Vahi M, Oopik V. Time-divided ingestion pattern of casein-based protein supplement stimulates an increase in fat-free body mass during resistance training in young untrained men. Nutrition Research 2009 June;29(6):405-13. Diet Intervention or Supplement Study
Ref ID: 706

(759) Burke DG, Smith-Palmer T, Holt LE, Head B, Chilibeck PD. The effect of 7 days of creatine supplementation on 24-hour urinary creatine excretion. Journal of Strength & Conditioning Research 15(1):59-62, 2001 Feb 2001;(1):59-62. Study limited to adults
Ref ID: 2902

(760) Burke DG, Chilibeck PD, Parise G, Tarnopolsky MA, Candow DG. Effect of alpha-lipoic acid combined with creatine monohydrate on human skeletal muscle creatine and phosphagen concentration. International Journal of Sport Nutrition & Exercise Metabolism 2003 September;13(3):294-302. Diet Intervention or Supplement Study
Ref ID: 1690

(761) Burke LE, Warziski M, Styn MA, Music E, Hudson AG, Sereika SM. A randomized clinical trial of a standard versus vegetarian diet for weight loss: the impact of treatment preference. Int J Obes (Lond) 2008 January;32(1):166-76. Study limited to adults
Ref ID: 222

(762) Burke LE, Conroy MB, Sereika SM, Elci OU, Styn MA, Acharya SD, Sevick MA, Ewing LJ, Glanz K. The effect of electronic self-monitoring on weight loss and dietary intake: a randomized behavioral weight loss trial. Obesity 2011 February;19(2):338-44. Study limited to adults
Ref ID: 1061

(763) Burke V, Beilin LJ, Milligan R, Thompson C. Assessment of nutrition and physical activity education programmes in children. [Review] [33 refs]. Clinical & Experimental Pharmacology & Physiology 1995 March;22(3):212-6. Review article
Ref ID: 2199

(764) Burke V, Thompson C, Taggart AC, Spickett EE, Beilin LJ, Vandongen R, Milligan RA, Dunbar DL. Differences in response to nutrition and fitness education programmes in relation to baseline levels of cardiovascular risk in 10 to 12-year-old children. Journal of Human Hypertension 1996 September;10:Suppl-106. Not All Participants were Overweight and/or Obese
Ref ID: 2159

(765) Burke V, Milligan RA, Thompson C, Taggart AC, Dunbar DL, Spencer MJ, Medland A, Gracey MP, Vandongen R, Beilin LJ. A controlled trial of health promotion programs in 11-year-olds using physical activity "enrichment" for higher risk children. Journal of Pediatrics 1998 May;132(5):840-8. Not All Participants were Overweight and/or Obese
Ref ID: 2091

(766) Burke V, Beilin LJ, Dunbar D. Family lifestyle and parental body mass index as predictors of body mass index in Australian children: a longitudinal study. International Journal of Obesity 2001;25(2):147-57. Not a randomized controlled trial (RCT)
Ref ID: 5187

(767) Burke V, Giangiulio N, Gillam HF, Beilin LJ, Houghton S. Physical activity and nutrition programs for couples: a randomized controlled trial. J Clin Epidemiol 2003 May;56(5):421-32. Study limited to adults
Ref ID: 360

(768) Burke V, Beilin LJ, Durkin K, Stritzke WG, Houghton S, Cameron CA. Television, computer use, physical activity, diet and fatness in Australian adolescents. International Journal of Pediatric Obesity 2006;1(4):248-55. Cross-sectional study
Ref ID: 1298

(769) Burman P, Ritzen EM, Lindgren AC. Endocrine dysfunction in Prader-Willi syndrome: a review with special reference to GH. [Review] [119 refs]. Endocrine Reviews 2001 December;22(6):787-99. Review article
Ref ID: 1869

(770) Burnier D, Dubois L, Girard M. Arguments at Mealtime and Child Energy Intake. Journal of nutrition education and behavior 2011;43(6):473-81. Cross-sectional study
Ref ID: 5188

(771) Burns JS, Williams PL, Sergeyev O, Korrick SA, Lee MM, Revich B, Altshul L, Del Prato JT, Humblet O, Patterson DG, Turner WE, Starovoytov M, Hauser R. Serum Concentrations of Organochlorine Pesticides and Growth among Russian Boys. Environmental Health Perspectives 2012;120(2):303-8. Cohort Study
Ref ID: 5189

(772) Burns SF, Corrie H, Holder E, Nightingale T, Stensel DJ. A single session of resistance exercise does not reduce postprandial lipaemia. Journal of Sports Sciences 2005 March;23(3):251-60. Acute study
Ref ID: 1501

(773) Burns SF, Oo HH, Tran AT. Effect of sprint interval exercise on postexercise metabolism and blood pressure in adolescents. International Journal of Sport Nutrition & Exercise Metabolism 2012 February;22(1):47-54. No control group (NC)
Ref ID: 2438

(774) Burr JF, Jamnik VK, Gledhill N. Physiological fitness and health adaptations from purposeful training using off-road vehicles. European Journal of Applied Physiology 2011 August;111(8):1841-50. Not an exercise intervention study
Ref ID: 2439

(775) Burri BJ, Neidlinger TR, Clifford AJ. Serum carotenoid depletion follows first-order kinetics in healthy adult women fed naturally low carotenoid diets. Journal of Nutrition 2001 August;131(8):2096-100. Diet Intervention or Supplement Study
Ref ID: 1903

(776) Burrows Argote R. Prevención y tratamiento de la obesidad desde la niñez: la estrategia para disminuir las enfermedades crónicas no transmisibles del adulto. Rev méd Chile 2000 January;128(1):105-10. Review article
Ref ID: 4021

(777) Burrows Argote R, Burgueño A, Díaz Bustos E, Gattás Z, SALAS A, De la Maza C. Protocolos de tratamiento. In: Albala Brevis C, Kain B, Burrows Argote R, Díaz Bustos E, editors. Obesidad: un desafío pendiente.Santiago de Chile: Universitaria; 2000. p. 221-8.¬

Not a randomized controlled trial (RCT)
Ref ID: 4019

(778) Burrows Argote R. Prevención, diagnóstico y tratamiento de la obesidad infantil y juvenil: recomendaciones actuales. Rev chil nutr 2000 April;27(1):31-5. Review article
Ref ID: 4020

(779) Burrows Argote R, GATTAS Z, Leiva B, Barrera A, Burgueño A. Características biológicas, familiares y metabólicas de la obesidad infantil y juvenil. Rev méd Chile 2001 October;129(10):1155-62. Retrospective study
Ref ID: 4022

(780) BURROWS A, Díaz B, Sciaraffia M, GATTAS Z, Montoya C, Lera M. Hábitos de ingesta y actividad física en escolares, según tipo de establecimiento al que asisten^ies. Rev méd Chile 2008 January;136(1):53-63. Survey or questionnaire
Ref ID: 4023

(781) Burrows R, Ceballos X, Burgueno M, Muzzo S. Trends in puberal development of school age children living in the Metropolitan Region of Chile. Revista Medica de Chile 2010;138(1):61-7. Cohort Study
Ref ID: 5190

(782) Burrows T, Warren JM, Baur LA, Collins CE. Impact of a child obesity intervention on dietary intake and behaviors. Int J Obes (Lond) 2008 October;32(10):1481-8. No comparative control group
Ref ID: 174

(783) Burrows T, Warren JM, Collins CE. The impact of a child obesity treatment intervention on parent child-feeding practices. Int J Pediatr Obes 2010;5(1):43-50. No comparative control group
Ref ID: 119

(784) Burton E, Stice E. Evaluation of a healthy-weight treatment program for bulimia nervosa: a preliminary randomized trial. Behaviour Research & Therapy 2006 December;44(12):1727-38. Diet Intervention or Supplement Study
Ref ID: 1338

(785) Burtscher M, Gatterer H, Faulhaber M, Gerstgrasser W, Schenk K. Effects of intermittent hypoxia on running economy. International Journal of Sports Medicine 2010 September;31(9):644-50. Not All Participants were Overweight and/or Obese
Ref ID: 2440

(786) Bush CL, Pittman S, McKay S, Ortiz T, Wong WW, Klish WJ. Park-based obesity intervention program for inner-city minority children. Journal of Pediatrics 517;151(5):513-7. Diet & Exercise intervention
Ref ID: 1148

(787) Busnello FM, Bodanese LC, Pellanda LC, Santos ZE. Nutritional intervention and the impact on adherence to treatment in patients with metabolic syndrome. Arq Bras Cardiol 2011 September;97(3):217-24. Diet Intervention or Supplement Study
Ref ID: 2441

(788) Bussau VA, Fairchild TJ, Rao A, Steele P, Fournier PA. Carbohydrate loading in human muscle: an improved 1 day protocol. European Journal of Applied Physiology 2002 July;87(3):290-5. Diet Intervention or Supplement Study
Ref ID: 1810

(789) Bustamante Valdivia A, Seabra AFT, Silva RMGd, Maia JAR. Efectos de la actividad física y del nivel socioecómico en el sobrepeso y obesidad de escolares, Lima Este 2005^ies. Rev peru med exp salud publica 2007 June;24(2):121-8. Survey or questionnaire
Ref ID: 4025

(790) Bustamante C, Ulloa R, Melo C, Muñoz M, Sanhueza X. Evaluaciones de aptitud laboral para trabajos en gran altitud. Bol Cient Asoc Chil Segur 2000 December;2(4):62-5. Not an exercise intervention study
Ref ID: 4026

(791) Butler D. Science of dieting: slim pickings. Nature 2004 March 18;428(6980):252-4. Diet Intervention or Supplement Study
Ref ID: 1659

(792) Butte NE, Wong WW, Adolph AL, Puyau MR, Vohra FA, Zakeri IF. Validation of Cross-Sectional Time Series and Multivariate Adaptive Regression Splines Models for the Prediction of Energy Expenditure in Children and Adolescents Using Doubly Labeled Water. Journal of Nutrition 2010;140(8):1516-23. Not an exercise intervention study
Ref ID: 5191

(793) Butte NF, Wong WW, Hopkinson JM, Heinz CJ, Mehta NR, Smith EO. Energy requirements derived from total energy expenditure and energy deposition during the first 2 y of life. American Journal of Clinical Nutrition 2000 December;72(6):1558-69. Subjects less than 2 years old
Ref ID: 1947

(794) Butte NF, Christiansen E, Sorensen TIA. Energy imbalance underlying the development of childhood obesity. Obesity 2007;15(12):3056-66. Not an exercise intervention study
Ref ID: 5192

(795) Buzi F, Corna A, Pilotta A, Negrini F, Lombardi A, Re T, Ambrosi B. Loperamide test: a simple and highly specific screening test for hypercortisolism in children and adolescents. Acta Paediatrica 1997 November;86(11):1177-80. Drug intervention study
Ref ID: 2119

(796) Byrd-Williams CE, Belcher BR, Spruijt-Metz D, Davis JN, Ventura EE, Kelly L, Berhane K, Azen S, Goran MI. Increased physical activity and reduced adiposity in overweight Hispanic adolescents. Med Sci Sports Exerc 2010 March;42(3):478-84. No exercise only group, No comparative control group
Ref ID: 76

(797) Byrne NM, Hills AP, Hunter GR, Weinsier RL, Schutz Y. Metabolic equivalent: one size does not fit all. Journal of Applied Physiology 2005 September;99(3):1112-9. Study limited to adults
Ref ID: 1484

(798) Caan BJ, Flatt SW, Rock CL, Ritenbaugh C, Newman V, Pierce JP. Low-energy reporting in women at risk for breast cancer recurrence. Women's Healthy Eating and Living Group. Cancer Epidemiology, Biomarkers & Prevention 2000 October;9(10):1091-7. Not an exercise intervention study
Ref ID: 1954

(799) Caballero B, Clay T, Davis SM, Ethelbah B, Rock BH, Lohman T, Norman J, Story M, Stone EJ, Stephenson L, Stevens J. Pathways: a school-based, randomized controlled trial for the prevention of obesity in American Indian schoolchildren. Am J Clin Nutr 2003 November;78(5):1030-8. No exercise only group
Ref ID: 351

(800) Caballero C, Hernández B, Moreno H, Hernández-Girón C, Campero L, Cruz A, Lazcano-Ponce E. Obesidad, actividad e inactividad física en adolescentes de Morelos, México: un estudio longitudinal^ies. Arch latinoam nutr 2007 September;57(3):231-7. Cohort Study
Ref ID: 4027

(801) Cabral Sd, Barbosa FP, Cabral BG, Knackfuss MI, Medeiros HJ, Fernandes Filho J. A seleção brasileira de voleibol infanto-juvenil feminina e o seu perfil dermatoglífico^ipt. Acta cir bras 2005;20(supl.1):22-6. No control group (NC)
Ref ID: 4028

(802) Cade WT, Reeds DN, Mondy KE, Overton ET, Grassino J, Tucker S, Bopp C, Laciny E, Hubert S, Lassa-Claxton S, Yarasheski KE. Yoga lifestyle intervention reduces blood pressure in HIV-infected adults with cardiovascular disease risk factors. HIV Medicine 2010 July 1;11(6):379-88. Study limited to adults
Ref ID: 488

(803) Cadepond P, Ulmann MD, Baulieu MD. RU486 (MIFEPRISTONE): Mechanisms of Action and Clinical Uses. Annual Review of Medicine 1997 February;48(1):129. Review article
Ref ID: 3540

(804) Caggiani Malzone M, Farré Silva Y. 3er Consenso Uruguayo de Hipertensión Arterial en el Niño y el Adolescente^ies. Arch pediatr Urug 2006 October;77(3):300-7. Review article
Ref ID: 4029

(805) Cagnacci A, Cannoletta M, Volpe A. High-dose short-term folate administration modifies ambulatory blood pressure in postmenopausal women. A placebo-controlled study. European Journal of Clinical Nutrition 2009 October;63(10):1266-8. Study limited to adults
Ref ID: 3541

(806) Cagney KA, Browning CR. Exploring neighborhood-level variation in asthma and other respiratory diseases - The contribution of neighborhood social context. Journal of General Internal Medicine 2004;19(3):229-36. Survey or questionnaire
Ref ID: 5193

(807) Cai GW, Cole SA, Butte N, Bacino C, Diego V, Tan K, Goring HH, O'Rahilly S, Farooqi IS, Comuzzie AG. A quantitative trait locus on chromosome 18q for physical activity and dietary intake in Hispanic children. Obesity 2006;14(9):1596-604. Not an exercise intervention study
Ref ID: 5194

(808) Cain PA, Ahl R, Hedstrom E, Ugander M, Allansdotter-Johnsson A, Friberg P, Marild S, Arheden H. Physiological determinants of the variation in left ventricular mass from early adolescence to late adulthood in healthy subjects.[Erratum appears in Clin Physiol Funct Imaging. 2007 Jul;27(4):254], [Republished in Clin Physiol Funct Imaging. 2007 Jul;27(4):255-62; PMID: 17564676]. Clinical Physiology & Functional Imaging 2005 November;25(6):332-9. Not an exercise intervention study
Ref ID: 1456

(809) CalaÃ±as-Continente A, Arrizabalaga JJ, CaixÃ s A, Cuatrecasas G, DÃ­az-FernÃ¡ndez MJ, GarcÃ­a-Luna PP, Goday A, Masmiquel L, Monereo S, Morales MJ, Moreiro J, Moreno B, Ricart W, Vidal J, Cordido F. Strategies for treating overweight in adolescents and their families. Endocrinol Nutr 2008;55(SUPPL. 4):60-77. Review article
Ref ID: 3200

(810) Calders P, Elmahgoub S, de Mettelinge TR, Vandenbroeck C, Dewandele I, Rombaut L, Vandevelde A, Cambier D. Effect of combined exercise training on physical and metabolic fitness in adults with intellectual disability: a controlled trial. Clinical Rehabilitation 25(12):1097-108, 2011 Dec 2011;(12):1097-108. Study limited to adults
Ref ID: 2906

(811) Caliandro P, Grugni G, Padua L, Kodra Y, Tonali P, Gargantini L, Ragusa L, Crino A, Taruscio D. Quality of life assessment in a sample of patients affected by Prader-Willi syndrome. Journal of Paediatrics & Child Health 2007 December;43(12):826-30. Survey or questionnaire
Ref ID: 1137

(812) Calles-Escandon J, Arciero PJ, Gardner AW, Bauman C, Poehlman ET. Basal fat oxidation decreases with aging in women. Journal of Applied Physiology 1995 January;78(1):266-71. Study limited to adults
Ref ID: 2214

(813) Camargo Lemos DM. Factores asociados a la calidad de vida en adolescentes de Bucaramanga^ies
Factors associated to living standards for teenagers in Bucaramanga^ien. Rev Univ Ind Santander, Salud 2009 April 30;41(1):33-42. Cross-sectional study
Ref ID: 4030

(814) Cameron EC, Maehle V, Reid J. The effects of an early physical therapy intervention for very preterm, very low birth weight infants: a randomized controlled clinical trial. Pediatric physical therapy : the official publication of the Section on Pediatrics of the American Physical Therapy Association 2005;17:107-19. Subjects less than 2 years old
Ref ID: 4706

(815) Cameron JW. Self-esteem changes in children enrolled in weight management programs. Issues in Comprehensive Pediatric Nursing 22(2-3):75-85, 1999 Apr-Sep 1999;(2-3):75-85. Survey or questionnaire
Ref ID: 2907

(816) Campbell-Yeo ML, Allen AC, Joseph KS, Ledwidge JM, Allen VM, Dooley KC. Study protocol: A double blind placebo controlled trial examining the effect of domperidone on the composition of breast milk [NCT00308334]. BMC pregnancy and childbirth 2006;6:17. Drug intervention study
Ref ID: 4707

(817) Campbell K, Waters E, O'Meara S, Summerbell C. Interventions for preventing obesity in childhood. A systematic review. [Review] [31 refs]. Obes Rev 2001 August;2(3):149-57. Review article
Ref ID: 1862

(818) Campbell K, Hesketh K, Crawford D, Salmon J, Ball K, McCallum Z. The Infant Feeding Activity and Nutrition Trial (INFANT) an early intervention to prevent childhood obesity: cluster-randomised controlled trial. BMC Public Health 2008 March 31;8:103.:103. Description versus conduct of study
Ref ID: 187

(819) Campbell ML. Administering Child Protection - A Feminist Analysis of the Conceptual Practices of Organizational Power. Canadian Public Administration-Administration Publique du Canada 1992;35(4):501-18. Case-Control / Case Study
Ref ID: 5195

(820) Campbell VS, Sinha DP, Patterson AW. KAP study on project lifestyle (Jamaica). Cajanus 1992;25(1):25-48. Survey or questionnaire
Ref ID: 698

(821) Campo Osaba MA, Del Val JL, Lapena C, Laguna V, Garcia A, Lozano O, Martin Z, Rodriguez R, Borras E, Orfila F, Tierno MT. The effectiveness of a health promotion with group intervention by clinical trial. Study protocol. BMC Public Health 2012;12:209. Description of study from review or magazine or etc. (not the actual study)
Ref ID: 2443

(822) Campos Cavada I. Factores de riesgo modificables para enfermedad cardiovascular en niños^ies
Modifiable risk factors for cardiovascular diseases in children^ien. An venez nutr 2010 December;23(2):100-7. Review article
Ref ID: 4031

(823) Campos Wd, Stabelini Neto A, Bozza R, Ulbrich AZ, Bertin RL, Mascarenhas LPG, Silva SGd, Sasaki JE. Atividade física, consumo de lipídios e fatores de risco para aterosclerose em adolescentes^ipt
Physical activity, lipid consumption and risk factors for atherosclerosis in adolescents^ien. Arq bras cardiol 2010 May;94(5):601-7. Cross-sectional study
Ref ID: 4032

(824) Can F, Yilmaz I, Erden Z. Morphological characteristics and performance variables of women soccer players. Journal of Strength & Conditioning Research 2004 August;18(3):480-5. Not an exercise intervention study
Ref ID: 1599

(825) Candow DG, Burke NC, Smith-Palmer T, Burke DG. Effect of whey and soy protein supplementation combined with resistance training in young adults. International Journal of Sport Nutrition & Exercise Metabolism 2006 June;16(3):233-44. Diet Intervention or Supplement Study
Ref ID: 1353

(826) Canessa E. Modeling of body mass index by Newton's second law. Journal of Theoretical Biology 2007;248(4):646-56. Not an exercise intervention study
Ref ID: 5196

(827) Canessa F, Santullo B, López A. Rendimiento en la carrera de 30 metros en niños sanos de 3 a 4 años. Kinesiologia 2002 March;(66):16-20. Not an exercise intervention study
Ref ID: 745

(828) Canhadas IL, Silva RLP, Chaves CR, Portes LA. Anthropometric and physical fitness characteristics of young male soccer players^ien. Rev bras cineantropom desempenho hum 2010 August;12(4). Cross-sectional study
Ref ID: 4033

(829) Canuto KJ, McDermott RA, Cargo M, Esterman AJ. Study protocol: a pragmatic randomised controlled trial of a 12-week physical activity and nutritional education program for overweight Aboriginal and Torres Strait Islander women. BMC Public Health 2011;11:655. Study limited to adults
Ref ID: 1052

(830) Capozza RF, Cointry GR, Cure-Ramirez P, Ferretti JL, Cure-Cure C. A DXA study of muscle-bone relationships in the whole body and limbs of 2512 normal men and pre- and post-menopausal women. Bone 2004;35(1):283-95. Study limited to adults
Ref ID: 5197

(831) Caranti DA, de Mello MT, Prado WL, Tock L, Siqueira KO, de PA, Lofrano MC, Cristofalo DM, Lederman H, Tufik S, Damaso AR. Short- and long-term beneficial effects of a multidisciplinary therapy for the control of metabolic syndrome in obese adolescents. Metabolism: Clinical & Experimental 2007 September;56(9):1293-300. Lifestyle Intervention, Not a randomized controlled trial (RCT)
Ref ID: 1182

(832) Cardon G, De B, I, De CD. Knowledge and perceptions about back education among elementary school students, teachers, and parents in Belgium. The Journal of school health 2002;72:100-6. Educational intervention
Ref ID: 4708

(833) Cardon G, Labarque V, Smits D, De B, I. Promoting physical activity at the pre-school playground: the effects of providing markings and play equipment. Preventive Medicine 2009;48:335-40. Not All Participants were Overweight and/or Obese
Ref ID: 4709

(834) Cardoso AT, Oliveira YRDd, Carvalho Jd, Nahas MV. Efeitos da atividade física de baixo teor metabólico sobre alguns componentes corporais: % G, Gord. Total e massa metabólica magra. Rev bras ciênc esporte 1987 May;8(2/3):163-5. Study limited to adults
Ref ID: 4034

(835) Cardoso LD, de Castro IRR, Gomes FD, Leite ID. Individual and school environment factors associated with overweight in adolescents of the municipality of Rio de Janeiro, Brazil. Public Health Nutrition 2011;14(5):914-22. Cross-sectional study
Ref ID: 5198

(836) Carei TR, Fyfe-Johnson AL, Breuner CC, Brown MA. Randomized controlled clinical trial of yoga in the treatment of eating disorders. J Adolesc Health 2010 April;46(4):346-51. Not All Participants were Overweight and/or Obese
Ref ID: 566

(837) Carey VJ, Yong FH, Frenkel LM, McKinney RE. Pediatric AIDS prognosis using somatic growth velocity. Aids 1998;12(11):1361-9. Not an exercise intervention study
Ref ID: 5199

(838) Carletti L, Rodrigues AN, Perez AJ, Vassallo DV. Resposta da pressão arterial ao esforço em adolescentes: influência do sobrepeso e obesidade^ipt. Arq bras cardiol 2008 July;91(1):25-30. Not a randomized controlled trial (RCT)
Ref ID: 4035

(839) Carlo WA, Goudar SS, Jehan I, Chomba E, Tshefu A, Garces A, Parida S, Althabe F, McClure EM, Derman RJ, Goldenberg RL, Bose C, Hambidge M, Panigrahi P, Buekens P, Chakraborty H, Hartwell TD, Moore J, Wright LL, -First-Breath-Study-Group. High mortality rates for very low birth weight infants in developing countries despite training. Pediatrics 2010;126:e1072-e1080. Subjects less than 2 years old
Ref ID: 4710

(840) Carlock JM, Smith SL, Hartman MJ, Morris RT, Ciroslan DA, Pierce KC, Newton RU, Harman EA, Sands WA, Stone MH. The relationship between vertical jump power estimates and weightlifting ability: a field-test approach. Journal of Strength & Conditioning Research 2004 August;18(3):534-9. Acute study
Ref ID: 1596

(841) Carlson JA, Sallis JF, Ramirez ER, Patrick K, Norman GJ. Physical activity and dietary behavior change in Internet-based weight loss interventions: comparing two multiple-behavior change indices. Preventive Medicine 2012 January;54(1):50-4. Study limited to adults
Ref ID: 1033

(842) Carlson MC, Saczynski JS, Rebok GW, Seeman T, Glass TA, McGill S, Tielsch J, Frick KD, Hill J, Fried LP. Exploring the effects of an "everyday" activity program on executive function and memory in older adults: Experience Corps. The Gerontologist 2008;48:793-801. Study limited to adults
Ref ID: 4711

(843) Carlson SE, Werkman SH, Peeples JM, Cooke RJ, Tolley EA. Arachidonic-Acid Status Correlates with 1St Year Growth in Preterm Infants. Proceedings of the National Academy of Sciences of the United States of America 1993;90(3):1073-7. Subjects less than 2 years old
Ref ID: 5200

(844) Carneiro O, Jardim PC. Pressäo arterial em tribo Xavante: comparaçäo 15 anos depois. Arq bras cardiol 1993 November;61(5):279-82. Study limited to adults
Ref ID: 4036

(845) Carnelosso ML, Barbosa MA, Porto CC, Silva SA, Carvalho MMd, Oliveira ALI. Prevalência de fatores de risco para doenças cardiovasculares na região leste de Goiânia (GO)^ipt
Prevalence of risk factors for cardiovascular diseases in the east region of Goiânia, Goiás State^ien. Ciênc saúde coletiva 2010 June;15(supl.1):1073-80. Cross-sectional study
Ref ID: 4037

(846) Caronia LM, Martin C, Welt CK, Sykiotis GP, Quinton R, Thambundit A, Avbelj M, Dhruvakumar S, Plummer L, Hughes VA, Seminara SB, Boepple PA, Sidis Y, Crowley WF, Martin KA, Hall JE, Pitteloud N. A Genetic Basis for Functional Hypothalamic Amenorrhea. New England Journal of Medicine 2011;364(3):215-25. Study limited to adults
Ref ID: 5201

(847) Carpenter WH, Poehlman ET, Oconnell M, Goran MI. Influence of Body-Composition and Resting Metabolic-Rate on Variation in Total-Energy Expenditure - A Metaanalysis. American Journal of Clinical Nutrition 1995;61(1):4-10. Review article
Ref ID: 5202

(848) Carpentier MY, Mullins LL, Elkin TD, Wolfe-Christensen C. Prevalence of multiple health-related behaviors in adolescents with cancer. Journal of Pediatric Hematology/Oncology 2008 December;30(12):902-7. Survey or questionnaire
Ref ID: 833

(849) Carr A, Cooper DA. Adverse effects of antiretroviral therapy. Lancet 2000 October 21;356(9239):1423. Review article
Ref ID: 3543

(850) Carrasco F, Moreno M, Irribarra V, Rodríguez L, Martin MA, Alarcón A, Mizón C, Echenique C, Saavedra V, Pizarra T, Atalah E. Evaluación de un programa piloto de intervención en adultos con sobrepeso u obesidad, en riesgo de diabetes^ies. Rev méd Chile 2008 January;136(1):13-21. Study limited to adults
Ref ID: 4038

(851) Carrasco P, Pérez B, Angel B, Albala B, Santos M, Larenas Y, Montalvo V. Prevalencia de diabetes tipo 2 y obesidad en dos poblaciones aborígenes de Chile en ambiente urbano^ies. Rev méd Chile 2004 October;132(10):1189-97. Study limited to adults
Ref ID: 4039

(852) Carrel AL, Myers SE, Whitman BY, Allen DB. Growth hormone improves body composition, fat utilization, physical strength and agility, and growth in Prader-Willi syndrome: A controlled study. Journal of Pediatrics 1999 February;134(2):215-21. Drug intervention study
Ref ID: 2050

(853) Carrel AL, Myers SE, Whitman BY, Allen DB. Sustained benefits of growth hormone on body composition, fat utilization, physical strength and agility, and growth in Prader-Willi syndrome are dose-dependent. Journal of Pediatric Endocrinology 2001 September;14(8):1097-105. Drug intervention study
Ref ID: 1890

(854) Carrel AL, Clark RR, Peterson SE, Nemeth BA, Sullivan J, Allen DB. Improvement of fitness, body composition, and insulin sensitivity in overweight children in a school-based exercise program: a randomized, controlled study. Arch Pediatr Adolesc Med 2005 October;159(10):963-8. No exercise only group, Lifestyle Intervention
Ref ID: 298

(855) Carrel AL, Clark RR, Peterson S, Eickhoff J, Allen DB. School-based fitness changes are lost during the summer vacation. Archives of Pediatrics & Adolescent Medicine 2007 June;161(6):561-4. Not an exercise intervention study
Ref ID: 1224

(856) Carrel AL, McVean JJ, Clark RR, Peterson SE, Eickhoff JC, Allen DB. School-based exercise improves fitness, body composition, insulin sensitivity, and markers of inflammation in non-obese children. Journal of Pediatric Endocrinology 2009 May;22(5):409-15. Not All Participants were Overweight and/or Obese
Ref ID: 716

(857) Carrel AL, Myers SE, Whitman BY, Eickhoff J, Allen DB. Long-Term Growth Hormone Therapy Changes the Natural History of Body Composition and Motor Function in Children with Prader-Willi Syndrome. Journal of Clinical Endocrinology & Metabolism 2010;95(3):1131-6. Drug intervention study
Ref ID: 5203

(858) Carrera F, Ramos K, Velasco CA. Manejo de la obesidad infantil^ies. Rev GASTROHNUP 2010 January 15;12(1):S31-S37. Survey or questionnaire
Ref ID: 4040

(859) Carruth BR, Skinner JD. Revisiting the picky eater phenomenon: Neophobic behaviors of young children. Journal of the American College of Nutrition 2000;19(6):771-80. Diet Intervention Study
Ref ID: 5204

(860) Carter FA, Bulik CM. Childhood obesity prevention programs: how do they affect eating pathology and other psychological measures?. [Review] [56 refs]. Psychosomatic Medicine 2008 April;70(3):363-71. Review article
Ref ID: 974

(861) Carter GT, Weiss MD, Chamberlain JR, Han JJ, Abresch RT, MirÃ³ J, Jensen MP. Aging with muscular dystrophy: Pathophysiology and clinical management. Phys Med Rehabil Clin North Am 2010;21(2):429-50. Review article
Ref ID: 3201

(862) Carter NJ, Keating GM. Bosentan: In pediatric patients with pulmonary arterial hypertension. Pediatr Drugs 2010;12(1):63-73. Drug intervention study
Ref ID: 3202

(863) Caruso-Davis MK, Guillot TS, Podichetty VK, Mashtalir N, Dhurandhar NV, Dubuisson O, Yu Y, Greenway FL. Efficacy of low-level laser therapy for body contouring and spot fat reduction. Obesity Surgery 2011 June;21(6):722-9. Study limited to adults
Ref ID: 2446

(864) Caruso JF, Signorile JF, Perry AC, Leblanc B, Williams R, Clark M, Bamman MM. The effects of albuterol and isokinetic exercise on the quadriceps muscle group. Medicine & Science in Sports & Exercise 1995 November;27(11):1471-6. Drug intervention study
Ref ID: 2191

(865) Caruso JF, Hamill JL, De GN. Oral albuterol dosing during the latter stages of a resistance exercise program. Journal of Strength & Conditioning Research 2005 February;19(1):102-7. Drug intervention study
Ref ID: 1545

(866) Carvalho Filho G, Chueire AG, Ignácio H, Carneiro MdO, Francese Neto J, Canesin AC. Tratamento cirúrgico de luxação congênita do quadril pós marcha: redução aberta e osteotomia de Salter
Surgical Treatment of the congenital dislocation of the hip after walking age: open reduction and Salter´s osteotomy. Acta ortop bras 2003 March;11(1):42-7. Not an exercise intervention study
Ref ID: 4041

(867) Carvalho JFd, Caleiro MTC, Bonfá E. Hyperhomocysteinemia and primary antiphospholipid syndrome^ien
Hiper-homocisteinemia e síndrome antifosfolípide primária^ipt. Rev bras reumatol 2009 August;49(4). Cross-sectional study
Ref ID: 4042

(868) Carvalho JFd, Caleiro MTC. Lipoprotein(a) in primary antiphospholipid syndrome^ien
Lipoproteína(a) na síndrome antifosfolípide primária^ipt. Rev bras reumatol 2009 June;49(3). Cross-sectional study
Ref ID: 4043

(869) Carvalho LAPd, Rodacki ALF. A influência de duas mochilas sobre a cinemática da coluna de crianças^ipt. Rev bras educ fís esp 2008 March;22(1):44-52. Not an exercise intervention study
Ref ID: 4044

(870) Carvalho P, Oliveira B, Barros R, Padrao P, Moreira P, Teixeira VH. Impact of fluid restriction and ad libitum water intake or an 8% carbohydrate-electrolyte beverage on skill performance of elite adolescent basketball players. International Journal of Sport Nutrition & Exercise Metabolism 2011 June;21(3):214-21. Diet Intervention or Supplement Study
Ref ID: 2447

(871) Casanueva E. V, Milos G, Chiang S. MT, Espejo G, Cid C, Riquelme M. Efectos de un programa de entrenamiento aeróbico de dos años de duración sobre el perfil lipídico de escolares obesos. Rev chil pediatr 1992 December;63(6):312-5. Lifestyle Intervention
Ref ID: 815

(872) Casazza K, Ciccazzo M. Improving the dietary patterns of adolescents using a computer-based approach. Journal of School Health 2006 February;76(2):43-6. Diet Intervention Study
Ref ID: 1420

(873) Casazza K, Ciccazzo M. The method of delivery of nutrition and physical activity information may play a role in eliciting behavior changes in adolescents. Eating Behaviors 2007 January;8(1):73-82. Lifestyle Intervention
Ref ID: 1292

(874) Casazza K, Hanks LJ, Beasley TM, Fernandez JR. Beyond Thriftiness: Independent and Interactive Effects of Genetic and Dietary Factors on Variations in Fat Deposition and Distribution Across Populations. American Journal of Physical Anthropology 2011;145(2):181-91. Not an exercise intervention study
Ref ID: 5205

(875) Casey AA, Elliott M, Glanz K, Haire-Joshu D, Lovegreen SL, Saelens BE, Sallis JF, Brownson RC. Impact of the food environment and physical activity environment on behaviors and weight status in rural U.S. communities. Preventive Medicine 2008 December;47(6):600-4. Cross-sectional study
Ref ID: 843

(876) Casiro O, Bingham W, MacMurray B, Whitfield M, Saigal S, Vincer M, Long W. One-year follow-up of 89 infants with birth weights of 500 to 749 grams and respiratory distress syndrome randomized to two rescue doses of synthetic surfactant or air placebo. Canadian Exosurf Neonatal Study Group. Canadian Exosurf Neonatal Follow-Up Group. The Journal of pediatrics 1995;126:S53-S60. Subjects less than 2 years old
Ref ID: 4712

(877) Castelli DM, Hillman CH, Hirsch J, Hirsch A, Drollette E. FIT Kids: Time in target heart zone and cognitive performance. Preventive Medicine 2011 June;52:Suppl-9. Not a randomized controlled trial (RCT)
Ref ID: 1043

(878) Castelnuovo G, Manzoni GM, Villa V, Cesa GL, Pietrabissa G, Molinari E. The STRATOB study: design of a randomized controlled clinical trial of Cognitive Behavioral Therapy and Brief Strategic Therapy with telecare in patients with obesity and binge-eating disorder referred to residential nutritional rehabilitation. Trials [Electronic Resource] 2011;12:114. Description of study from review or magazine or etc. (not the actual study)
Ref ID: 2449

(879) Castillo C, Kain B. Consejería en vida sana y cambio de conductas en escolares obesos: intervención controlada en madres/cuidadoras^ies
Counseling on healthy lifestyles and behavioral change in obese school children: controlled intervention in mothers/caretakers^ien. Rev chil nutr 2010 June;37(2):155-63. Not a randomized controlled trial (RCT)
Ref ID: 4045

(880) Castillo I, Molina-García J. Adiposidad corporal y bienestar psicológico: efectos de la actividad física en universitarios de Valencia, España^ies
Adiposity and psychological well-being: effects of physical activity on university students in Valencia, Spain^ien. Rev panam salud pública 2009 October;26(4):334-40. Study limited to adults
Ref ID: 4046

(881) Castro IRRd, Cardoso LO, Engstrom EM, Levy RB, Monteiro CA. Vigilância de fatores de risco para doenças não transmissíveis entre adolescentes: a experiência da cidade do Rio de Janeiro, Brasil^ipt. Cad saúde pública 2008 October;24(10):2279-88. Survey or questionnaire
Ref ID: 4047

(882) Catellier DJ, Hannan PJ, Murray DM, Addy CL, Conway TL, Yang S, Rice JC. Imputation of missing data when measuring physical activity by accelerometry. Medicine & Science in Sports & Exercise 2005 November;37(11:Suppl):Suppl-62. Not an exercise intervention study
Ref ID: 1447

(883) Catenassi FZ, Marques I, Bastos CB, Basso L, Ronque ERV, Gerage AM. Relação entre índice de massa corporal e habilidade motora grossa em crianças de quatro a seis anos^ipt. Rev bras med esporte 2007 August;13(4):227-30. Not a randomized controlled trial (RCT)
Ref ID: 4048

(884) Catina P. Psychological modeling and adaptations in cognitive representations with increased resistance during motor skill acquisition. Journal of Strength & Conditioning Research 2009 March;23(2):668-76. Study limited to adults
Ref ID: 791

(885) Cattai GB, Hintze LJ, Nardo Junior N. Validação interna do questionário de estágio de prontidão para mudança do comportamento alimentar e de atividade física^ipt
Internal validation of the stage of change questionnaire for alimentary and physical activity behaviors^ien. Rev paul pediatr 2010 June;28(2):194-9. Not an exercise intervention study
Ref ID: 4049

(886) Cattai GBP, Rocha FA, Hintze LJ, Pagan BGM, Junior NN. Programa de tratamento multiprofissional da obesidade: os desafios da prática^ipt. Ciênc cuid saúde 2008 May;7(supl.1):121-6. Lifestyle Intervention
Ref ID: 4050

(887) Cauchi S, Stutzmann F, Cavalcanti-Proenca C, Durand E, Pouta A, Hartikainen AL, Marre M, Vol S, Tammelin T, Laitinen J, Gonzalez-Izquierdo A, Blakemore AI, Elliott P, Meyre D, Balkau B, Jarvelin MR, Froguel P. Combined effects of MC4R and FTO common genetic variants on obesity in European general populations. Journal of Molecular Medicine 2009 May;87(5):537-46. Cohort Study
Ref ID: 766

(888) Caumo W, Hidalgo MPL, Schmidt AP, Iwamoto CW, Adamatti LC, Bergmann J, Ferreira MBC. Effect of pre-operative anxiolysis on postoperative pain response in patients undergoing total abdominal hysterectomy. Anaesthesia 2002 August;57(8):740-6. Drug intervention study
Ref ID: 3544

(889) Cavalcanti CBdS, Barros MVGd, Menêses AL, Santos CM, Azevedo AMP, Guimarães FJdSP. Obesidade abdominal em adolescentes: prevalência e associação com atividade física e hábitos alimentares^ipt
Abdominal obesity in adolescents: prevalence and association with physical activity and eating habits^ien. Arq bras cardiol 2010 March;94(3):371-7. Survey or questionnaire
Ref ID: 4051

(890) Cawley MM, Benson LM. Current Trends in Managing Oral Mucositis. Clinical Journal of Oncology Nursing 2005 October;9(5):584-92. Not an exercise intervention study
Ref ID: 3545

(891) Cebulj Navarrete D, Vildoso Castillo JF, Quezada Donoso E, Figueroa Mellado F, Prieto Correa MJ, Díaz Narváez VP, Maturana Aracena P, Orellana Campos B. Función pulmonar en niños sanos de 7 y 8 años de las comunas de Cerro Navia y Los Andes expuestos a diferentes niveles de contaminación por MP10^ies
Lung functionin healthy children aged 7 and 8 of the comunes of Cerro Navia and Los Andes exposes to different levels of pollution by MP10^ien. Salud UNINORTE 2011 December;27(2):198-209. Not an exercise intervention study
Ref ID: 4052

(892) Cecil JE, Tavendale R, Watt P, Hetherington MM, Palmer CNA. An Obesity-Associated FTO Gene Variant and Increased Energy Intake in Children. New England Journal of Medicine 2008;359(24):2558-66. Cross-sectional study
Ref ID: 5206

(893) Cederborg J, Freinkel S, John F, Motte P, Spence E, Spitz T, Weaver A, Wong K, Wrightson C. healthy news. Health (Time Inc Health) 2000 September;14(7):19. Description of study from review or magazine or etc. (not the actual study)
Ref ID: 3887

(894) Cejudo P, Bautista J, Montemayor T, Villagomez R, Jimenez L, Ortega F, Campos Y, Sanchez H, Arenas J. Exercise training in mitochondrial myopathy: a randomized controlled trial. Muscle & Nerve 2005 September;32(3):342-50. Not a randomized controlled trial (RCT)
Ref ID: 1479

(895) Celermajer DS. Wait for weight or "waste" the waist: the benefits of early intervention in childhood obesity. Journal of the American College of Cardiology 2009 December 15;54(25):2407-8. Editorial or letter or comment
Ref ID: 619

(896) Celio AA. Early intervention of eating- and weight-related problems via the Internet in overweight adolescents: A randomized controlled trial. United States -- California: University of California, San Diego and San Diego State University; 2005.¬

Lifestyle Intervention
Ref ID: 5103

(897) Cerezo Correa MdP, Vergara Quintero MdC, Nieto Murillo E, Cifuentes Aguirre OL, Parra Sánchez JH. Características de salud pública de estudiantes de una universidad privada de la ciudad de Manizales^ies
Public health characteristics of a private university students in Manizales^ien
Carateristicas de saúde publica de estudantes duma universidade privada da cidade de Manizales^ipt. Hacia promoc salud 2011 June;16(1):73-86. Study limited to adults
Ref ID: 4053

(898) Cesar CMPdCdS, Oliveira TA, Santos JFKd, Souza Ed, Camano L. Uso da monitorizaçäo da contraçäo uterina e da autopalpaçäo materna para prevenir a prematuridade. Femina 1998 October;26(9):743-6. Subjects less than 2 years old
Ref ID: 4054

(899) Cesar JA, Mendoza-Sassi R, Horta BL, Ribeiro PRP, D'Avila AC, Santos FM, Martins PB, Brandolt RR. Basic indicators of child health in an urban area in southern Brazil: estimating prevalence rates and evaluating differentials^ien. J pediatr (Rio J ) 2006 December;82(6):437-44. Cross-sectional study
Ref ID: 651

(900) Cesar MC, Borin JP, Gonelli PR, Simoes RA, de Souza TM, Montebelo MI. The effect of local muscle endurance training on cardiorespiratory capacity in young women. Journal of Strength & Conditioning Research 2009 September;23(6):1637-43. Study limited to adults
Ref ID: 680

(901) Cezar C. O tratamento da obesidade estruturado em terapêutica multiprofissional. Pediatr mod 2000 March;36(3):140-6. Review article
Ref ID: 4055

(902) Chad KE, Bailey DA, McKay HA, Zello GA, Snyder RE. The effect of a weight-bearing physical activity program on bone mineral content and estimated volumetric density in children with spastic cerebral palsy. The Journal of pediatrics 1999;135:115-7. Not a randomized controlled trial (RCT), Not All Participants were Overweight and/or Obese
Ref ID: 4713

(903) Chaddock L, Erickson KI, Prakash RS, Kim JS, Voss MW, VanPatter M, Pontifex MB, Raine LB, Konkel A, Hillman CH, Cohen NJ, Kramer AF. A neuroimaging investigation of the association between aerobic fitness, hippocampal volume, and memory performance in preadolescent children. Brain Research 2010;1358:172-83. Not a randomized controlled trial (RCT)
Ref ID: 5207

(904) Chaddock L, Hillman CH, Buck SM, Cohen NJ. Aerobic Fitness and Executive Control of Relational Memory in Preadolescent Children. Medicine and science in sports and exercise 2011;43(2):344-9. Not a randomized controlled trial (RCT)
Ref ID: 5208

(905) Chae HW, Kwon YN, Rhie YJ, Kim HS, Kim YS, Paik IY, Suh SH, Kim DH. Effects of a structured exercise program on insulin resistance, inflammatory markers and physical fitness in obese Korean children. J Pediatr Endocrinol Metab 2010 October;23(10):1065-72. No exercise only group
Ref ID: 4

(906) Chagas PSdCC, Soares TBdC, Mancini MC, Fonseca STd, Vaz DV, Gontijo APB. Mudanças antropométricas e habilidade motora em crianças no início da marcha independente^ipt. Fisioter pesqui 2006;13(2):43-9. Subjects less than 2 years old
Ref ID: 654

(907) Chagnon YC, Rice T, Perusse L, Borecki IB, Ho-Kim MA, Lacaille M, Pare C, Bouchard L, Gagnon J, Leon AS, Skinner JS, Wilmore JH, Rao DC, Bouchard C, HERITAGE Family. Genomic scan for genes affecting body composition before and after training in Caucasians from HERITAGE. Journal of Applied Physiology 2001 May;90(5):1777-87. Not an exercise intervention study
Ref ID: 1921

(908) Chamorro-Vina C, Ruiz JR, Santana-Sosa E, Gonzalez VM, Madero L, Perez M, Fleck SJ, Perez A, Ramirez M, Lucia A. Exercise during hematopoietic stem cell transplant hospitalization in children. Medicine & Science in Sports & Exercise 2010 June;42(6):1045-53. Study less than 4 weeks
Ref ID: 529

(909) Champagne CM, Bray GA, Kurtz AA, Monteiro JBR, Tucker E, Volaufova J, DeLany JP. Energy intake and energy expenditure: A controlled study comparing dietitians and non-dietitians. Journal of the American Dietetic Association 2002;102(10):1428-32. Not an exercise intervention study
Ref ID: 5209

(910) Champkin J. Making information beautiful - and clear. Significance 2011 March;8(1):39-41. Not an exercise intervention study
Ref ID: 3546

(911) Chan Osilla K, Van Busum K, Schnyer C, Wozar Larkin J, Eibner C, Mattke S. Systematic Review of the Impact of Worksite Wellness Programs. American Journal of Managed Care 2012 February;18(2):e68-e81. Review article
Ref ID: 3547

(912) CHANG CY, SCHIANO TD. Review article: drug hepatotoxicity. Alimentary Pharmacology & Therapeutics 2007 May 15;25(10):1135-51. Review article
Ref ID: 3548

(913) Chang KW. Randomized controlled trial of Coblation versus electrocautery tonsillectomy. Otolaryngol Head Neck Surg 2005;132(2):273-80. Not an exercise intervention study
Ref ID: 3203

(914) Chang MW, Brown R, Nitzke S. Participant recruitment and retention in a pilot program to prevent weight gain in low-income overweight and obese mothers. BMC Public Health 2009 November 21;9:424.:424. Study not limited to children and adolescents
Ref ID: 80

(915) Chang MW, Nitzke S, Brown R. Design and outcomes of a Mothers In Motion behavioral intervention pilot study. J Nutr Educ Behav 2010 May;42(3 Suppl):S11-S21. Study not limited to children and adolescents
Ref ID: 46

(916) Chanoine JP, Hampl S, Jensen C, Boldrin M, Hauptman J. Effect of orlistat on weight and body composition in obese adolescents: a randomized controlled trial. JAMA 2005 June 15;293(23):2873-83. No exercise only group, Drug intervention study
Ref ID: 309

(917) Chaparro CM, Dewey KG. Use of lipid-based nutrient supplements (LNS) to improve the nutrient adequacy of general food distribution rations for vulnerable sub-groups in emergency settings. Maternal & Child Nutrition 2010 January 2;6:1-69. Diet Intervention or Supplement Study
Ref ID: 3549

(918) Chapelot D. The Role of Snacking in Energy Balance: a Biobehavioral Approach. Journal of Nutrition 2011;141(1):158-62. Diet Intervention or Supplement Study
Ref ID: 5210

(919) Chapman P, Toma RB, Tuveson RV, Jacob M. Nutrition knowledge among adolescent high school female athletes. Adolescence 1997;32(126):437-46. Diet Intervention or Supplement Study
Ref ID: 2140

(920) Chaput JP, Visby T, Nyby S, Klingenberg L, Gregersen NT, Tremblay A, Astrup A, Sjodin A. Video game playing increases food intake in adolescents: a randomized crossover study. American Journal of Clinical Nutrition 2011 June;93(6):1196-203. Acute study
Ref ID: 2451

(921) Charbonnel B, Karasik A, Liu J, Wu M, Meininger G, Sitagliptin S. Efficacy and safety of the dipeptidyl peptidase-4 inhibitor sitagliptin added to ongoing metformin therapy in patients with type 2 diabetes inadequately controlled with metformin alone. Diabetes Care 2006 December;29(12):2638-43. Drug intervention study
Ref ID: 1322

(922) Chateauvieux SÃ, Morceau F, Dicato M, Diederich M. Molecular and Therapeutic Potential and Toxicity of Valproic Acid. Journal of Biomedicine & Biotechnology 2010 January;1-18. Drug intervention study
Ref ID: 3550

(923) Chavarro JE, Peterson KE, Sobol AM, Wiecha JL, Gortmaker SL. Effects of a school-based obesity-prevention intervention on menarche (United States). Cancer Causes Control 2005 December;16(10):1245-52. No exercise only group
Ref ID: 296

(924) Chaves CRMdM, Oliveira CQd, Britto JAAd, Elsas MICG. Exercício aeróbico, treinamento de força muscular e testes de aptidão física para adolescentes com fibrose cística: revisão da literatura^ipt
Exercise testing, aerobic and strength training for adolescents with cystic fibrosis: a literature review^ien. Rev bras saúde matern infant 2007 September;7(3):245-50. Review article
Ref ID: 4056

(925) Chaves ES, Silva VMd, Costa FBC, Araujo TLd. Avaliação do índice de massa corporal em crianças e adolescentes de uma Escola Pública de Fortaleza - Ceará^ipt
Evaluación del índice de masa corpórea en niños y adolescentes de una escuela pública de Fortaleza-Ceará^ies. Rev paul enferm 2004 March;23(1):37-42. Not All Participants were Overweight and/or Obese
Ref ID: 487

(926) Chavez-Tapia NC, Sanchez-Avila F, Vasquez-Fernandez F, Torres-Machorro A, Tellez-Avila FI, Uribe M. Non-alcoholic fatty-liver disease in pediatric populations. J Pediatr Endocrinol Metab 2007;20(10):1059-73. Review article
Ref ID: 3204

(927) Chawla K, Mishra R, Sachdeva V, Beenu. Correlation of antioxidants and fitness levels in undergraduate medical students. Indian Journal of Physiology & Pharmacology 2007 July;51(3):293-5. Study limited to adults
Ref ID: 1114

(928) Checon K, Fonseca VM, Faria CPd, Carletti L, Molina MdCB. Reprodutibilidade do questionário de avaliação de atividade física para crianças aplicado no Estudo Saúdes: Vitória^ipt
The reproducibility of the Saúdes: Vitória Study's physical activity assessment questionnaire for children^ien. Rev bras saúde matern infant 2011 June;11(2):173-80. Cross-sectional study, Survey or questionnaire
Ref ID: 4057

(929) Chehuen MdR, Bezerra AIL, Bartholomeu T, Junqueira NO, Rezende JAS, Basso L, Oliveira JA, Lemos WP, Tani G, Prista A, Maia JAR, Forjaz CLdM. Risco cardiovascular e prática de atividade física em crianças e adolescentes de Muzambinho/MG: influência do gênero e da idade^ipt
Cardiovascular risk and physical activity practice in children and adolescents of Muzambinho/MG: influence of gender and age^ien. Rev bras med esporte 2011 August;17(4):232-6. Cross-sectional study
Ref ID: 4058

(930) Chellini E, Talassi F, Corbo G, Berti G, De SM, Rusconi F, Piffer S, Caranci N, Petronio MG, Sestini P, Dell'Orco V, Bonci E, Armenio L, La GS, Gruppo Collaborativo SIDR. [Environmental, social and demographic characteristics of children and adolescents, resident in different Italian areas]. [Italian]. Epidemiologia e Prevenzione 2005 March;29(2:Suppl):Suppl-23. Cross-sectional study
Ref ID: 1477

(931) Chen CC, Lin SY. The impact of rope jumping exercise on physical fitness of visually impaired students. Research in Developmental Disabilities 2011 January;32(1):25-9. Not All Participants were Overweight and/or Obese, Not a randomized controlled trial (RCT)
Ref ID: 2452

(932) Chen HL, Lee CL, Tseng HI, Yang SN, Yang RC, Jao HC. Assisted exercise improves bone strength in very low birthweight infants by bone quantitative ultrasound. Journal of Paediatrics and Child Health 2010;46:653-9. Subjects less than 2 years old
Ref ID: 4714

(933) Chen J, Sadakata M, Ishida M, Sekizuka N, Sayama M. Baby massage ameliorates neonatal jaundice in full-term newborn infants. The Tohoku journal of experimental medicine 2011;223:97-102. Subjects less than 2 years old
Ref ID: 4715

(934) Chen JL, Weiss SJ, Heyman MB, Cooper B, Lustig RH. The Active Balance Childhood program for improving coping and quality of life in Chinese American children. Nursing Research 2010;59:270-9. No exercise only group
Ref ID: 4716

(935) Chen JL, Weiss S, Heyman MB, Lustig RH. Efficacy of a child-centred and family-based program in promoting healthy weight and healthy behaviors in Chinese American children: a randomized controlled study. J Public Health (Oxf) 2010 June;32(2):219-29. No exercise only group
Ref ID: 78

(936) Chen JL, Weiss S, Heyman MB, Cooper B, Lustig RH. The efficacy of the web-based childhood obesity prevention program in Chinese American adolescents (Web ABC study). J Adolesc Health 2011 August;49(2):148-54. Lifestyle Intervention
Ref ID: 2453

(937) Chen KT, Chen YY, Wu HJ, Chang CK, Lee WT, Lu YY, Liu CC, Yang RS, Lin JC. Decreased anaerobic performance and hormone adaptation after expedition to Peak Lenin. Chinese Medical Journal 2008 November 20;121(22):2229-33. Not a randomized controlled trial (RCT)
Ref ID: 840

(938) Chen LL, Su YC, Su CH, Lin HC, Kuo HW. Acupressure and meridian massage: combined effects on increasing body weight in premature infants.[Erratum appears in J Clin Nurs. 2008 Aug;17(15):2089]. Journal of Clinical Nursing 17(9):1174-81, 2008 May 2008;(9):1174-81. Subjects less than 2 years old
Ref ID: 2918

(939) Chen SL, Li LL, Li HL. [Effect of massage in traditional Chinese medicine on short penis in male obese children]. Chinese Journal of Clinical Rehabilitation 2006;10:168-9. Not an exercise intervention study
Ref ID: 4717

(940) Chen TL, Mao HC, Lai CH, Li CY, Kuo CH. [The effect of yoga exercise intervention on health related physical fitness in school-age asthmatic children]. [Chinese]. Hu Li Tsa Chih Journal of Nursing 2009;56:42-52. Not a randomized controlled trial (RCT)
Ref ID: 4718

(941) Chen X, Wang Y. Is ideal body image related to obesity and lifestyle behaviours in African American adolescents? Child: Care, Health & Development 2012 March;38(2):219-28. Survey or questionnaire
Ref ID: 2454

(942) Cheng-Mayer C, Watkins D, Marthas M, Picker LJ. 20[sup th] Annual Symposium on Nonhuman Primate Models for AIDS. Journal of Medical Primatology 32[4/5], 265. 2003. Abstract,
Ref ID: 3551

(943) Cherkas LF, Hunkin JL, Kato BS, Richards JB, Gardner JP, Surdulescu GL, Kimura M, Lu X, Spector TD, Aviv A. The association between physical activity in leisure time and leukocyte telomere length. Archives of Internal Medicine 2008 January 28;168(2):154-8. Survey or questionnaire
Ref ID: 1005

(944) Cherng RJ, Liu CF, Lau TW, Hong RB. Effect of treadmill training with body weight support on gait and gross motor function in children with spastic cerebral palsy. American Journal of Physical Medicine & Rehabilitation 2007 July;86(7):548-55. Not All Participants were Overweight and/or Obese
Ref ID: 1211

(945) Cheung BM. Drug treatment for obesity in the post-sibutramine era. Drug Safety 2011 August 1;34(8):641-50. Drug intervention study
Ref ID: 2455

(946) Cheung SS, Sleivert GG. Lowering of skin temperature decreases isokinetic maximal force production independent of core temperature. European Journal of Applied Physiology 2004 May;91(5-6):723-8. Not an exercise intervention study
Ref ID: 1642

(947) Chevallier JM, Paita M, Rodde-Dunet MH, Marty M, Nogues F, Slim K, Basdevant A. Predictive factors of outcome after gastric banding: a nationwide survey on the role of center activity and patients' behavior. Ann Surg 2007 December;246(6):1034-9. Survey or questionnaire
Ref ID: 1134

(948) Chiappetta DA, Alvarez-Lorenzo C, Rey-Rico A, Taboada P, Concheiro A, Sosnik A. N-alkylation of poloxamines modulates micellar assembly and encapsulation and release of the antiretroviral efavirenz. European Journal of Pharmaceutics and Biopharmaceutics 2010;76(1):24-37. Drug intervention study
Ref ID: 5211

(949) Chiaratti PS, Sprocatti R, Piovesana AM. Crianças de alto risco: evoluçäo dos aspectos linguísticos e cognitivos. Temas desenvolv 2001 August;10(57):19-23. Not an exercise intervention study
Ref ID: 731

(950) Childs JD, Teyhen DS, Van Wyngaarden JJ, Dougherty BF, Ladislas BJ, Helton GL, Robinson ME, Wu SS, George SZ. Predictors of web-based follow-up response in the Prevention Of Low Back Pain In The Military Trial (POLM). BMC Musculoskeletal Disorders 2011;12:132. Study limited to adults
Ref ID: 2456

(951) Chinapaw MJ, Proper KI, Brug J, van MW, Singh AS. Relationship between young peoples' sedentary behaviour and biomedical health indicators: a systematic review of prospective studies. [Review]. Obes Rev 2011 July;12(7):e621-e632. Review article
Ref ID: 2457

(952) Chipps BE. Asthma in Infants and Children. Clin Cornerstone 2008;8(4):44-61. Review article
Ref ID: 3205

(953) Cho AH, Killeya-Jones LA, O'Daniel JM, Kawamoto K, Gallagher P, Haga S, Lucas JE, Trujillo GM, Joy SV, Ginsburg GS. Effect of genetic testing for risk of type 2 diabetes mellitus on health behaviors and outcomes: study rationale, development and design. BMC Health Services Research 2012;12:16. Description of study from review or magazine or etc. (not the actual study)
Ref ID: 2458

(954) Chomitz VR, Collins J, Kim J, Kramer E, McGowan R. Promoting healthy weight among elementary school children via a health report card approach. Archives of Pediatrics & Adolescent Medicine 2003;157:765-72. Not a randomized controlled trial (RCT)
Ref ID: 4719

(955) Chomtho S, Wells JC, Williams JE, Davies PS, Lucas A, Fewtrell MS. Infant growth and later body composition: evidence from the 4-component model. American Journal of Clinical Nutrition 2008 June;87(6):1776-84. Cross-sectional study
Ref ID: 939

(956) Choudhuri D, Choudhuri S, Kulkarni VA. Physical fitness: a comparative study between students of residential (Sainik) and non-residential schools (aged 12-14 years). Indian Journal of Physiology & Pharmacology 2002 July;46(3):328-32. Cross-sectional study
Ref ID: 1783

(957) Chrisoulidou A, Kousta E, Beshyah SA, Robinson S, Johnston DG. How much, and by what mechanisms, does growth hormone replacement improve the quality of life in GH-deficient adults?. [Review] [52 refs]. Baillieres Clinical Endocrinology & Metabolism 1998 July;12(2):261-79. Review article
Ref ID: 2065

(958) Christ ER, Cummings MH, Westwood NB, Sawyer BM, Pearson TC, Sonksen PH, Russell-Jones DL. The importance of growth hormone in the regulation of erythropoiesis, red cell mass, and plasma volume in adults with growth hormone deficiency. Journal of Clinical Endocrinology & Metabolism 1997 September;82(9):2985-90. Study limited to adults
Ref ID: 2127

(959) Christian JG, Byers TE, Christian KK, Goldstein MG, Bock BC, Prioreschi B, Bessesen DH. A computer support program that helps clinicians provide patients with metabolic syndrome tailored counseling to promote weight loss. Journal of the American Dietetic Association 2011 January;111(1):75-83. Study limited to adults
Ref ID: 2459

(960) Christiansen LI, LÃ¤hteenmÃ¤ki PLA, Mannelin MR, SeppÃ¤nen-Laakso TE, Hiltunen RVK, Yliruusi JK. Cholesterol-lowering effect of spreads enriched with microcrystalline plant sterols in hypercholesterolemic subjects. European Journal of Nutrition 2001 April;40(2):66. Diet Intervention or Supplement Study
Ref ID: 491

(961) Christiansen T, Paulsen SK, Bruun JM, Overgaard K, Ringgaard S, Pedersen SB, Positano V, Richelsen B. Comparable reduction of the visceral adipose tissue depot after a diet-induced weight loss with or without aerobic exercise in obese subjects: a 12-week randomized intervention study. Eur J Endocrinol 2009 May;160(5):759-67. No comparative control group
Ref ID: 135

(962) Christiansen T, Paulsen SK, Bruun JM, Pedersen SB, Richelsen B. Exercise training versus diet-induced weight-loss on metabolic risk factors and inflammatory markers in obese subjects: a 12-week randomized intervention study. Am J Physiol Endocrinol Metab 2010 April;298(4):E824-E831. No comparative control group
Ref ID: 70

(963) Christiansen T, Paulsen SK, Bruun JM, Ploug T, Pedersen SB, Richelsen B. Diet-induced weight loss and exercise alone and in combination enhance the expression of adiponectin receptors in adipose tissue and skeletal muscle, but only diet-induced weight loss enhanced circulating adiponectin. J Clin Endocrinol Metab 2010 February;95(2):911-9. Study not limited to children and adolescents, No comparative control group
Ref ID: 74

(964) Christison A, Khan HA. Exergaming for health: a community-based pediatric weight management program using active video gaming. Clinical Pediatrics 2012 April;51(4):382-8. Not a randomized controlled trial (RCT)
Ref ID: 2460

(965) Christofaro DGD, Andrade SMd, Fernandes RA, Ohara D, Dias DF, Freitas Júnior IF, Oliveira DRd. Prevalência de fatores de risco para doenças cardiovasculares entre escolares em Londrina - PR: diferenças entre classes econômicas^ipt
Prevalence of risk factors for cardiovascular diseases among students of Londrina - PR: differences between economic classes^ien. Rev bras epidemiol 2011 March;14(1):27-35. Cross-sectional study
Ref ID: 4059

(966) Chromiak JA, Smedley B, Carpenter W, Brown R, Koh YS, Lamberth JG, Joe LA, Abadie BR, Altorfer G. Effect of a 10-week strength training program and recovery drink on body composition, muscular strength and endurance, and anaerobic power and capacity. Nutrition 2004 May;20(5):420-7. Diet Intervention or Supplement Study
Ref ID: 1640

(967) Chu L, Riddell MC, Takken T, Timmons BW. Carbohydrate intake reduces fat oxidation during exercise in obese boys. European Journal of Applied Physiology 2011 December;111(12):3135-41. Diet Intervention or Supplement Study
Ref ID: 2461

(968) Chuang SC, Gallo V, Michaud D, Overvad K, Tjonneland A, Clavel-Chapelon F, Romieu I, Straif K, Palli D, Pala V, Tumino R, Sacerdote C, Panico S, Peeters PH, Lund E, Gram IT, Manjer J, Borgquist S, Riboli E, Vineis P. Exposure to environmental tobacco smoke in childhood and incidence of cancer in adulthood in never smokers in the European Prospective Investigation into Cancer and Nutrition. Cancer Causes & Control 2011 March;22(3):487-94. Not an exercise intervention study
Ref ID: 2462

(969) Chubak J, Tworoger SS, Yasui Y, Ulrich CM, Stanczyk FZ, McTiernan A. Associations between reproductive and menstrual factors and postmenopausal sex hormone concentrations. Cancer Epidemiology, Biomarkers & Prevention 2004 August;13(8):1296-301. Study limited to adults
Ref ID: 1603

(970) Chumnijarakij T, Nuchprayoon T, Chitinand S, Onthuam Y, Quamkul N, Dusitsin N, Viputsiri OA, Chotiwan P, Limpongsanurak S, Sukomol P. Maternal risk factors for low birth weight newborn in Thailand. Journal of the Medical Association of Thailand 1992 August;75(8):445-52. Not an exercise intervention study
Ref ID: 2275

(971) Church C, Lee S, Bagg EAL, McTaggart JS, Deacon R, Gerken T, Lee A, Moir L, Mecinovic J, Quwailid MM, Schofield CJ, Ashcroft FM, Cox RD. A Mouse Model for the Metabolic Effects of the Human Fat Mass and Obesity Associated FTO Gene. Plos Genetics 2009;5(8). Animal study
Ref ID: 5212

(972) Church C, Moir L, McMurray F, Girard C, Banks GT, Teboul L, Wells S, Bruning JC, Nolan PM, Ashcroft FM, Cox RD. Overexpression of Fto leads to increased food intake and results in obesity. Nature Genetics 2010;42(12):1086-U147. Animal study
Ref ID: 5213

(973) Ciampo LAD, Rodrigues DMS, Ciampo IRLD, Cardoso VC, Bettiol H, Barbieri MA. Percepção corporal e atividade física em uma coorte de adultos jovens brasileiros^ipt
Body image and physical activity among a brazilian youth cohort^ien. Rev bras crescimento desenvolv hum 2010;20(3):671-9. Cohort Study
Ref ID: 4060

(974) Ciampolini M, Bini S, Giommi A, Vicarelli D, Giannellini V. Same growth and different energy intake over four years in children suffering from chronic non-specific diarrhoea. International journal of obesity and related metabolic disorders : journal of the International Association for the Study of Obesity 1994;18:17-23. Not an exercise intervention study
Ref ID: 4720

(975) Ciccolo JT, Dunsiger SI, Williams DM, Bartholomew JB, Jennings EG, Ussher MH, Kraemer WJ, Marcus BH. Resistance training as an aid to standard smoking cessation treatment: a pilot study. Nicotine & Tobacco Research 2011 August;13(8):756-60. Study limited to adults
Ref ID: 2463

(976) Cieslak TJ, Frost G, Klentrou P. Effects of physical activity, body fat, and salivary cortisol on mucosal immunity in children. Journal of Applied Physiology 2003 December;95(6):2315-20. Cross-sectional study
Ref ID: 1706

(977) Cimadon HMS, Geremia R, Pellanda LC. Hábitos alimentares e fatores de risco para aterosclerose em estudantes de Bento Gonçalves (RS)^ipt
Dietary habits and risk factors for atherosclerosis in students from Bento Gonçalves (state of Rio Grande do Sul)^ien. Arq bras cardiol 2010 August;95(2):166-72. Cross-sectional study
Ref ID: 4061

(978) Cimolin V, Galli M, Grugni G, Vismara L, Albertini G, Rigoldi C, Capodaglio P. Gait patterns in Prader-Willi and Down syndrome patients. Journal of Neuroengineering & Rehabilitation 2010;7:28. Not an exercise intervention study
Ref ID: 495

(979) Cinar V, Mogulkoc R, Baltaci AK, Polat Y. Adrenocorticotropic hormone and cortisol levels in athletes and sedentary subjects at rest and exhaustion: effects of magnesium supplementation. Biological Trace Element Research 2008 March;121(3):215-20. Diet Intervention or Supplement Study
Ref ID: 1002

(980) Cinar V, Polat Y, Baltaci AK, Mogulkoc R. Effects of magnesium supplementation on testosterone levels of athletes and sedentary subjects at rest and after exhaustion. Biological Trace Element Research 2011 April;140(1):18-23. Diet Intervention or Supplement Study
Ref ID: 2464

(981) Citrome L, Vreeland B. Schizophrenia, obesity, and antipsychotic medications: What can we do? Postgraduate Medicine 2008;120(2):18-33. Review article
Ref ID: 3206

(982) Claeyssens S, Peynet J, Gazengel C, Alcalay M, Bertrand MA, Briquel ME, Derlon A, Guerois C, D'Oiron R, Pautard B, Stieltjes N, Sultan Y, Bridey F, Goudemand J. FACTEUR IX-LFB(TM): Experiment in surgery. Sang Thrombose Vaisseaux 1998;10:52-7. Drug intervention study
Ref ID: 919

(983) Clapp JF, Kiess W. Cord blood leptin reflects fetal fat mass. Journal of the Society for Gynecologic Investigation 1998;5(6):300-3. Not an exercise intervention study
Ref ID: 5214

(984) Clapp JF, III, Simonian S, Lopez B, Appleby-Wineberg S, Harcar-Sevcik R. The one-year morphometric and neurodevelopmental outcome of the offspring of women who continued to exercise regularly throughout pregnancy. American Journal of Obstetrics & Gynecology 1998 March;178(3):594-9. Subjects less than 2 years old
Ref ID: 2098

(985) Clar D, Cruciani D, Molinari J. Hidratación en la alta competencia: debito hidrosalino del tenista de alta competición. Rev Argent Med Deporte 1995;17(56):24-30. Not an exercise intervention study
Ref ID: 4062

(986) Clark EN, Dewey AM, Temple JL. Effects of daily snack food intake on food reinforcement depend on body mass index and energy density. Am J Clin Nutr 2010 February;91(2):300-8. Not an exercise intervention study
Ref ID: 73

(987) Clarkson PM, Devaney JM, Gordish-Dressman H, Thompson PD, Hubal MJ, Urso M, Price TB, Angelopoulos TJ, Gordon PM, Moyna NM, Pescatello LS, Visich PS, Zoeller RF, Seip RL, Hoffman EP. ACTN3 genotype is associated with increases in muscle strength in response to resistance training in women. Journal of Applied Physiology 2005 July;99(1):154-63. Study limited to adults
Ref ID: 1490

(988) Clarson CL, Mahmud FH, Baker JE, Clark HE, McKay WM, Schauteet VD, Hill DJ. Metformin in combination with structured lifestyle intervention improved body mass index in obese adolescents, but did not improve insulin resistance. Endocrine 2009 August;36(1):141-6. No exercise only group, No comparative control group
Ref ID: 125

(989) Claudi-Magnussen C. The consumers' view/reaction. Acta Veterinaria Scandinavica 2006 January 2;48:S4-2. Abstract
Ref ID: 3552

(990) Clauss SB, Holmes KW, Hopkins P, Stein E, Cho M, Tate A, Johnson-Levonas AO, Kwiterovich PO. Efficacy and safety of lovastatin therapy in adolescent girls with heterozygous familial hypercholesterolemia. Pediatrics 2005;116(3):682-8. Diet Intervention Study, Drug intervention study
Ref ID: 3207

(991) Cleland V, Crawford D, Baur LA, Hume C, Timperio A, Salmon J. A prospective examination of children's time spent outdoors, objectively measured physical activity and overweight. International Journal of Obesity 2008 November;32(11):1685-93. Cross-sectional study
Ref ID: 859

(992) Clemmens D, Hayman LL. Increasing activity to reduce obesity in adolescent girls: a research review. [Review] [39 refs]. JOGNN - Journal of Obstetric, Gynecologic, & Neonatal Nursing 33(6):801-8, 2004 Nov-Dec 2004;(6):801-8. Review article
Ref ID: 2921

(993) Cliff DP, Wilson A, Okely AD, Mickle KJ, Steele JR. Feasibility of SHARK: a physical activity skill-development program for overweight and obese children. Journal of Science & Medicine in Sport 2007 August;10(4):263-7. Not a randomized controlled trial (RCT)
Ref ID: 1226

(994) Cliff DP, Okely AD, Smith LM, McKeen K. Relationships between fundamental movement skills and objectively measured physical activity in preschool children. Pediatric Exercise Science 2009 November;21(4):436-49. Cross-sectional study
Ref ID: 614

(995) Cliff DP, Okely AD, Morgan PJ, Steele JR, Jones RA, Colyvas K, Baur LA. Movement skills and physical activity in obese children: randomized controlled trial. Medicine & Science in Sports & Exercise 43(1):90-100, 2011 Jan 2011;(1):90-100. No control group (NC)
Ref ID: 2922

(996) Clifton P. Dietary fatty acids and inflammation. Nutrition & Dietetics 2009 March;66(1):7-11. Review article
Ref ID: 3553

(997) Clough GF, Turzyniecka M, Walter L, Krentz AJ, Wild SH, Chipperfield AJ, Gamble J, Byrne CD. Muscle microvascular dysfunction in central obesity is related to muscle insulin insensitivity but is not reversed by high-dose statin treatment. Diabetes 2009 May;58(5):1185-91. Study limited to adults, No exercise only group
Ref ID: 136

(998) Coelho CM, Silva RCd, Egashira EM, Ribeiro SML. Evolução do estado nutricional de crianças com mielomeningoceleem período de três anos^ipt
Tree-year period evolution of the nutritional condition of children with mielomeningocelis^ien
Evolución en tres años de la condición nutricional de niños con mielomeningocele^ies. Mundo saúde (Impr ) (1995) 2009 September;33(3):347-51. Cross-sectional study
Ref ID: 4063

(999) Coffey VG, Jemiolo B, Edge J, Garnham AP, Trappe SW, Hawley JA. Effect of consecutive repeated sprint and resistance exercise bouts on acute adaptive responses in human skeletal muscle. American Journal of Physiology - Regulatory Integrative & Comparative Physiology 2009 November;297(5):R1441-R1451. Acute study
Ref ID: 653

(1000) Cohen D, Scott M, Wang FZ, McKenzie TL, Porter D. School design and physical activity among middle school girls. Journal of Physical Activity & Health 2008;5:719-31. Cross-sectional study
Ref ID: 4721

(1001) Cohen MS, Zak V, Atz AM, Printz BF, Pinto N, Lambert L, Pemberton V, Li JS, Margossian R, Dunbar-Masterson C, McCrindle BW. Anthropometric measures after Fontan procedure: implications for suboptimal functional outcome. Am Heart J 2010 December;160(6):1092-8, 1098. Not a randomized controlled trial (RCT), Not an exercise intervention study
Ref ID: 6

(1002) Colado JC, Garcia-Masso X, Gonzalez LM, Triplett NT, Mayo C, Merce J. Two-leg squat jumps in water: an effective alternative to dry land jumps. International Journal of Sports Medicine 2010 February;31(2):118-22. Not a randomized controlled trial (RCT)
Ref ID: 572

(1003) Colak R, Ozcelik O. Effects of short-period exercise training and orlistat therapy on body composition and maximal power production capacity in obese patients. Physiological Research 2004;53(1):53-60. Diet Intervention Study, Drug intervention study
Ref ID: 1664

(1004) Cole CR, Rising R, Hakim A, Danon M, Mehta R, Choudhury S, Sundaresh M, Lifshitz F. Comprehensive assessment of the components of energy expenditure in infants using a new infant respiratory chamber. Journal of the American College of Nutrition 1999;18(3):233-41. Subjects less than 2 years old
Ref ID: 5215

(1005) Coledam DHC, Arruda GAd, Oliveira ARd. Efeito crônico do alongamento estático realizado durante o aquecimento sobre a flexibilidade de crianças^ipt
Chronic effect of static stretching performed during warm-up on flexibility in children^ien. Rev bras cineantropom desempenho hum 2012;14(3):296-304. Not a randomized controlled trial (RCT)
Ref ID: 4064

(1006) Coleman KJ, Raynor HR, Mueller DM, Cerny FJ, Dorn JM, Epstein LH. Providing sedentary adults with choices for meeting their walking goals. Preventive Medicine 1999 May;28(5):510-9. Study limited to adults
Ref ID: 2027

(1007) Coleman KJ, Heath EM, Alcalá IS. Overweight and aerobic fitness in children in the United States/Mexico border region. Rev panam salud pública 2004 April;15(4). Cross-sectional study
Ref ID: 4065

(1008) Colín RE, Castillo ML, Orea TA, Vergara CA, Keirns DC, Villa RA. Outcomes of a school-based intervention (RESCATE) to improve physical activity patterns in Mexican children aged 8-10 years. Health Education Research 2010;25:1042-9. Not All Participants were Overweight and/or Obese
Ref ID: 4722

(1009) Coll CdVN, Amorim TC, Hallal PC. Percepção de adolescentes e adultos referente à influência da mídia sobre o estilo de vida^ipt
Perception of adolescents and adults on the influence of media on Lifestyle^ien. Rev bras ativ fís saúde 2010 June;15(2). Cross-sectional study
Ref ID: 4066

(1010) Collaer ML, Brook CG, Conway GS, Hindmarsh PC, Hines M. Motor development in individuals with congenital adrenal hyperplasia: strength, targeting, and fine motor skill. Psychoneuroendocrinology 2009;34:249-58. Not an exercise intervention study
Ref ID: 4723

(1011) Collaku A, Rankinen T, Rice T, Leon AS, Rao DC, Skinner JS, Wilmore JH, Bouchard C. A genome-wide linkage scan for dietary energy and nutrient intakes: the Health, Risk Factors, Exercise Training, and Genetics (HERITAGE) Family Study. American Journal of Clinical Nutrition 2004 May;79(5):881-6. Not an exercise intervention study
Ref ID: 1637

(1012) Collard DC, Verhagen EA, Chinapaw MJ, Knol DL, van MW. Effectiveness of a school-based physical activity injury prevention program: a cluster randomized controlled trial. Archives of Pediatrics & Adolescent Medicine 2010 February;164(2):145-50. Not All Participants were Overweight and/or Obese
Ref ID: 592

(1013) Collins AL, McCarthy HD. Evaluation of factors determining the precision of body composition measurements by air displacement plethysmography. European Journal of Clinical Nutrition 2003;57(6):770-6. Not an exercise intervention study
Ref ID: 5216

(1014) Collins CE, Morgan PJ, Jones P, Fletcher K, Martin J, Aguiar EJ, Lucas A, Neve M, McElduff P, Callister R. Evaluation of a commercial web-based weight loss and weight loss maintenance program in overweight and obese adults: a randomized controlled trial. BMC Public Health 2010 November 3;10:669.:669. Study limited to adults
Ref ID: 14

(1015) Collins CE, Okely AD, Morgan PJ, Jones RA, Burrows TL, Cliff DP, Colyvas K, Warren JM, Steele JR, Baur LA. Parent diet modification, child activity, or both in obese children: an RCT. Pediatrics 2011 April;127(4):619-27. No non-intervention control group
Ref ID: 1083

(1016) Collins DL, Evans JM, Grundy RH. The efficiency of multiple impulse therapy for musculoskeletal complaints. Journal of Manipulative & Physiological Therapeutics 2006 February;29(2):162. Not an exercise intervention study
Ref ID: 1422

(1017) Colton P, Olmsted M, Daneman D, Rydall A, Rodin G. Disturbed eating behavior and eating disorders in preteen and early teenage girls with type 1 diabetes: a case-controlled study. Diabetes Care 2004 July;27(7):1654-9. Cross-sectional study
Ref ID: 1614

(1018) Comfort P, Pearson SJ, Mather D. An electromyographical comparison of trunk muscle activity during isometric trunk and dynamic strengthening exercises. Journal of Strength & Conditioning Research 2011 January;25(1):149-54. Study limited to adults
Ref ID: 2469

(1019) Comité Nacional de Medicina del Deporte Infanto-Juvenil.Subcomité de Emidemiología. Consenso sobre factores de riesgo de enfermedad cardiovascular en pediatría. Sedentarismo^ies. Arch argent pediatr 2005 May;103(5):450-63. Review article
Ref ID: 4067

(1020) Conde WL, Borges C. O risco de incidência e persistência da obesidade entre adultos Brasileiros segundo seu estado nutricional ao final da adolescência^ipt
The risk of incidence and persistence of obesity among Brazilian adults according to their nutritional status at the end of adolescence^ien. Rev bras epidemiol 2011 September;14(supl.1):71-9. Not an exercise intervention study
Ref ID: 4068

(1021) Conkin J, Powell MR, Gernhardt ML. Age affects severity of venous gas emboli on decompression from 14.7 to 4.3 psia. Aviation Space & Environmental Medicine 2003 November;74(11):1142-50. Study limited to adults
Ref ID: 1702

(1022) Connelly JB, Duaso MJ, Butler G. A systematic review of controlled trials of interventions to prevent childhood obesity and overweight: a realistic synthesis of the evidence. [Review] [43 refs]. Public Health 2007 July;121(7):510-7. Review article
Ref ID: 1212

(1023) Conners CK, Casat CD, Gualtieri CT, Weller E, Reader M, Reiss A, Weller RA, Khayrallah M, Ascher J. Bupropion hydrochloride in attention deficit disorder with hyperactivity. Journal of the American Academy of Child and Adolescent Psychiatry 1996;35:1314-21. Drug intervention study
Ref ID: 4724

(1024) Connolly AM, Schierbecker J, Renna R, Florence J. High dose weekly oral prednisone improves strength in boys with Duchenne muscular dystrophy. Neuromuscular Disorders 2002 December;12(10):917-25. Drug intervention study
Ref ID: 1791

(1025) Conrod PJ, Peterson JB, Pihl RO. Reliability and validity of alcohol-induced heart rate increase as a measure of sensitivity to the stimulant properties of alcohol. Psychopharmacology 2001 July 15;157(1):20. Drug intervention study
Ref ID: 486

(1026) Conte M, Gonçalves A, Aragon FF, Padovani CR. Influência da massa corporal sobre a aptidão física em adolescentes: estudo a partir de escolares do ensino fundamental e médio de Sorocaba/SP. Rev bras med esporte 2000 April;6(2):44-9. Cohort Study
Ref ID: 4069

(1027) Contento IR, Koch PA, Lee H, Calabrese-Barton A. Adolescents demonstrate improvement in obesity risk behaviors after completion of choice, control & change, a curriculum addressing personal agency and autonomous motivation. J Am Diet Assoc 2010 December;110(12):1830-9. No exercise only group, Not an exercise intervention study
Ref ID: 8

(1028) Contopoulos-Ioannidis DG, Seto I, Hamm MP, Thomson D, Hartling L, Ioannidis JPA, Curtis S, Constantin E, Batmanabane G, Klassen T, Williams K. Empirical evaluation of age groups and age-subgroup analyses in pediatric randomized trials and pediatric meta-analyses. Pediatrics 2012;129(SUPPL. 3):S161-S184. Review article
Ref ID: 3208

(1029) Conwell LS, Trost SG, Spence L, Brown WJ, Batch JA. The feasibility of a home-based moderate-intensity physical activity intervention in obese children and adolescents. British Journal of Sports Medicine 2010 March;44(4):250-5. Not a randomized controlled trial (RCT)
Ref ID: 563

(1030) Cooper JA, Watras AC, Shriver T, Adams AK, Schoeller DA. Influence of dietary fatty acid composition and exercise on changes in fat oxidation from a high-fat diet. Journal of Applied Physiology 2010 October;109(4):1011-8. Study limited to adults
Ref ID: 2471

(1031) Cooper JA, Watras AC, Paton CM, Wegner FH, Adams AK, Schoeller DA. Impact of exercise and dietary fatty acid composition from a high-fat diet on markers of hunger and satiety. Appetite 2011 February;56(1):171-8. Study limited to adults
Ref ID: 2472

(1032) Cooper JL. Dietary Lipids in the Aetiology of Alzheimerâ€™s Disease: Implications for Therapy. Drugs & Aging 2003 April;20(6):399-418. Review article
Ref ID: 3554

(1033) Cooper L, Lockwood B. Difficulty breathing? Just get eating. Pharm J 2006;276(7402):629-36. Not an exercise intervention study
Ref ID: 3209

(1034) Cooper R, Hypponen E, Berry D, Power C. Associations between parental and offspring adiposity up to midlife: the contribution of adult lifestyle factors in the 1958 British Birth Cohort Study. American Journal of Clinical Nutrition 2010;92(4):946-53. Cohort Study
Ref ID: 5217

(1035) Copeland KC, Zeitler P, Geffner M, Guandalini C, Higgins J, Hirst K, Kaufman FR, Linder B, Marcovina S, McGuigan P, Pyle L, Tamborlane W, Willi S, -TODAY-Study-Group. Characteristics of adolescents and youth with recent-onset type 2 diabetes: the TODAY cohort at baseline. The Journal of clinical endocrinology and metabolism 2011;96:159-67. Cohort Study
Ref ID: 4725

(1036) Copetti J, Neutzling MB, Silva MCd. Barreiras à prática de atividades físicas em adolescentes de uma cidade do sul do Brasil^ipt
Barriers to physical activity practice in adolescents of southern Brazilian city^ien. Rev bras ativ fís saúde 2010 June;15(2). Cross-sectional study
Ref ID: 4070

(1037) Coppins DF, Margetts BM, Fa JL, Brown M, Garrett F, Huelin S. Effectiveness of a multi-disciplinary family-based programme for treating childhood obesity (the Family Project). European Journal of Clinical Nutrition 2011 August;65(8):903-9. Lifestyle Intervention
Ref ID: 2473

(1038) Corapcioglu F, Guvenc BH, Sarper N, Aydogan A, Akansel G, Arisoy ES. Peritoneal tuberculosis with elevated serum CA 125 level mimicking advanced ovarian carcinoma in an adolescent. Turkish Journal of Pediatrics 2006;48(1):69-72. Not an exercise intervention study
Ref ID: 5218

(1039) Corbo GM, Forastiere F, Rusconi F, De SM, Biggeri A, Russo A, Chellini E, Brunetti L, Gruppo Collaborativo SIDR. [Dietary habits, life styles and respiratory symptoms in childhood]. [Italian]. Epidemiologia e Prevenzione 2005 March;29(2:Suppl):Suppl-6. Cross-sectional study
Ref ID: 1476

(1040) Corbo GM, Forastiere F, De SM, Brunetti L, Bonci E, Bugiani M, Chellini E, La GS, Migliore E, Pistelli R, Rusconi F, Russo A, Simoni M, Talassi F, Galassi C, Collaborative Group. Wheeze and asthma in children: associations with body mass index, sports, television viewing, and diet. Epidemiology 2008 September;19(5):747-55. Survey or questionnaire
Ref ID: 899

(1041) Cornish SM, Chilibeck PD, Burke DG. The effect of creatine monohydrate supplementation on sprint skating in ice-hockey players. Journal of Sports Medicine & Physical Fitness 2006 March;46(1):90-8. Diet Intervention or Supplement Study
Ref ID: 1401

(1042) Corr M, De Souza MJ, Toombs RJ, Williams NI. Circulating leptin concentrations do not distinguish menstrual status in exercising women. Human Reproduction 2011 March;26(3):685-94. Study limited to adults
Ref ID: 2474

(1043) Correa B. Determinación del perfil antropométrico y cualidades físicas de niños futbolistas de Bogotá^ies
Determination of the anthropometric and physical qualities profile in footballers children of Bogotá^ien. Rev cienc salud (Bogotá) 2008 August;6(2):74-84. Cross-sectional study
Ref ID: 4071

(1044) Correia CT, Almeida JP, Santos PE, Sequeira AF, Marques CE, Miguel TS, Abreu RL, Oliveira GG, Vicente AM. Pharmacogenetics of risperidone therapy in autism: association analysis of eight candidate genes with drug efficacy and adverse drug reactions. Pharmacogenomics Journal 2010 October;10(5):418-30. Drug intervention study
Ref ID: 3555

(1045) Corso ACT, Caldeira GV, Fiates GMR, Schmitz BdAS, Ricardo GD, Vasconcelos FdAGd. Fatores comportamentais associados ao sobrepeso e à obesidade em escolares do Estado de Santa Catarina^ipt
Behavioral factors associated with overweight and with obesity in students in the State of Santa Catarina^ien
Factores de comportamiento asociados al sobrepeso y a la obesidad en escolares del Estado de Santa Catarina^ies. Rev bras estud popul 2012 June;29(1):117-31. Cross-sectional study
Ref ID: 4072

(1046) Cosgrove MJ, Wilson J, Watt D, Grant SF. The relationship between selected physiological variables of rowers and rowing performance as determined by a 2000 m ergometer test. Journal of Sports Sciences 1999 November;17(11):845-52. Not a randomized controlled trial (RCT)
Ref ID: 2060

(1047) Cossio-Bolaños M, Figueroa P, Cossio-Bolaños WJ, Lázari E, Arruda M. Parámetros del crecimiento físico de niños que viven a moderada altitud^ies. Rev méd hered 2012 June;23(2):96-105. Cross-sectional study
Ref ID: 4073

(1048) Costa ARCd, Teodoro TN, Araújo MdFMd. Análise dos conhecimentos e da prática de profissionais de saúde na promoção e no apoio à amamentação: estudo de revisão^ipt. Comun ciênc saúde 2009 March;20(1):55-63. Review article
Ref ID: 4074

(1049) Costa FFd, Assis MAAd. Nível de atividade física e comportamentos sedentários de escolares de sete a dez anos de Florianópolis-SC^ipt
Physical activity and sedentary behaviour of schoolchildren aged 7 to 10 in Florianópolis-SC^ien. Rev bras ativ fís saúde 2011 March;16(1). Survey or questionnaire
Ref ID: 4075

(1050) Costa MF, Barbosa SCT, Barletta M, Dantas DV, Kehrig HA, Seixas TG, Malm O. Seasonal differences in mercury accumulation in Trichiurus lepturus (Cutlassfish) in relation to length and weight in a Northeast Brazilian estuary. Environmental Science and Pollution Research 2009;16(4):423-30. Not an exercise intervention study
Ref ID: 5219

(1051) Costa MCD, Cordoni Junior L, Matsuo T. Sobrepeso em adolescentes de 14 a 19 anos em um município da região Sul do Brasil^ipt
Overweight in adolescents aged 14 to 19 years old in a Southern Brazilian city^ien. Rev bras saúde matern infant 2007 September;7(3):263-70. Cross-sectional study
Ref ID: 4076

(1052) Costacurta M, Di Renzo L, Bianchi A, Fabiocchi F, De Lorenzo A, Docimo R. Obesity and dental caries in paediatric patients. A cross-sectional study. European Journal of Paediatric Dentistry 2011;12(2):112-6. Cross-sectional study
Ref ID: 5220

(1053) Costanzi CB, Halpern R, Rech RR, Bergmann MLdA, Alli LR, Mattos APd. Fatores associados a níveis pressóricos elevados em escolares de uma cidade de porte médio do sul do Brasil^ipt
Associated factors in high blood pressure among schoolchildren in a middle size city, southern Brazil^ien. J pediatr (Rio J ) 2009 August;85(4):335-40. Cross-sectional study
Ref ID: 4077

(1054) Coté CJ, Rolf N, Liu LM, Goudsouzian NG, Ryan JF, Zaslavsky A, Gore R, Todres TD, Vassallo S, Polaner D. A single-blind study of combined pulse oximetry and capnography in children. Anesthesiology 1991;74:980-7. Drug intervention study
Ref ID: 4726

(1055) Cotton B, Smith A, Hansen I, Davis C, Doyle A, Walsh A. Physician-directed primary care intervention to reduce risk factors for type 2 diabetes in high-risk youth. American Journal of the Medical Sciences 2006 September;332(3):108-11. No control group (NC)
Ref ID: 1349

(1056) Cottrell L, Spangler-Murphy E, Minor V, Downes A, Nicholson P, Neal WA. A kindergarten cardiovascular risk surveillance study: CARDIAC-Kinder. American Journal of Health Behavior 2005 November;29(6):595-606. Lifestyle Intervention
Ref ID: 1443

(1057) Coudreau SK, Tounian P, Bonhomme G, Froguel P, Girardet JP, Guy-Grand B, Basdevant A, Clement K. Role of the DGAT gene C79T single-nucleotide polymorphism in french obese subjects. Obesity Research 2003;11(10):1163-7. Not an exercise intervention study
Ref ID: 5221

(1058) Coukell AJ, Brogden RN. Liposomal Amphotericin B: Therapeutic Use in the Management of Fungal Infections and Visceral Leishmaniasis. Drugs 1998 April;55(4):585-612. Drug intervention study
Ref ID: 558

(1059) Counterweight Project Team. Evaluation of the Counterweight Programme for obesity management in primary care: a starting point for continuous improvement. British Journal of General Practice 2008 August;58(553):548-54. Not a randomized controlled trial (RCT)
Ref ID: 903

(1060) Courneya KS, Sellar CM, Stevinson C, McNeely ML, Peddle CJ, Friedenreich CM, Tankel K, Basi S, Chua N, Mazurek A, Reiman T. Randomized controlled trial of the effects of aerobic exercise on physical functioning and quality of life in lymphoma patients. Journal of Clinical Oncology 2009 September 20;27(27):4605-12. Study limited to adults
Ref ID: 666

(1061) Courneya KS, Stevinson C, McNeely ML, Sellar CM, Peddle CJ, Friedenreich CM, Mazurek A, Chua N, Tankel K, Basi S, Reiman T. Predictors of adherence to supervised exercise in lymphoma patients participating in a randomized controlled trial. Ann Behav Med 2010 August;40(1):30-9. Study not limited to children and adolescents
Ref ID: 36

(1062) Courteix D, Lespessailles E, Loiseau-Peres S, Obert P, Ferry B, Benhamou CL. Lean tissue mass is a better predictor of bone mineral content and density than body weight in prepubertal girls. Revue du Rhumatisme (English Edition) 1998 May;65(5):328-36. Not a randomized controlled trial (RCT)
Ref ID: 2088

(1063) Courteix D, Jaffre C, Lespessailles E, Benhamou L. Cumulative effects of calcium supplementation and physical activity on bone accretion in premenarchal children: a double-blind randomised placebo-controlled trial. International Journal of Sports Medicine 2005 June;26(5):332-8. Not All Participants were Overweight and/or Obese
Ref ID: 1519

(1064) Couser RJ, Hoekstra RE, Ferrara TB, Wright GB, Cabalka AK, Connett JE. Neurodevelopmental follow-up at 36 months' corrected age of preterm infants treated with prophylactic indomethacin. Archives of Pediatrics & Adolescent Medicine 2000;154:598-602. Subjects less than 2 years old
Ref ID: 4727

(1065) Cousins JH, Rubovits DS, Dunn JK, Reeves RS, Ramirez AG, Foreyt JP. Family versus individually oriented intervention for weight loss in Mexican American women. Public Health Rep 1992 September;107(5):549-55. Study limited to adults
Ref ID: 437

(1066) Cousins JM, Langer SM, Rhew LK, Thomas C. The role of state health departments in supporting community-based obesity prevention. Preventing Chronic Disease 2011 July;8(4):A87. Not an exercise intervention study
Ref ID: 2475

(1067) Coutant R, Carel JC, Timsit J, Boitard C, Bougneres P. Insulin and the prevention of insulin-dependent diabetes mellitus. [Review] [20 refs]. Diabetes & Metabolism 1997 September;23:Suppl-8. Review article
Ref ID: 2122

(1068) Coutinho W. The first decade of sibutramine and orlistat: a reappraisal of their expanding roles in the treatment of obesity and associated conditions. [Review] [60 refs]. Arquivos Brasileiros de Endocrinologia e Metabologia 2009 March;53(2):262-70. Review article
Ref ID: 746

(1069) Coutinho W. The first decade of sibutramine and orlistat: a reappraisal of their expanding roles in the treatment of obesity and associated conditions^ien
A primeira década da sibutramina e do orlistate: reavaliação do seu crescente papel no tratamento da obesidade e condições associadas^ipt. Arq bras endocrinol metab 2009 March;53(2):262-70. Drug intervention study
Ref ID: 4078

(1070) Coutts AJ, Murphy AJ, Dascombe BJ. Effect of direct supervision of a strength coach on measures of muscular strength and power in young rugby league players. Journal of Strength & Conditioning Research 2004 May;18(2):316-23. Not a randomized controlled trial (RCT)
Ref ID: 1629

(1071) Cowie RL, Boulet LP, Keith PK, Scott-Wilson CA, House KW, Dorinsky PM. Tolerability of a salmeterol xinafoate/fluticasone propionate hydrofluoroalkane metered-dose inhaler in adolescent and adult patients with persistent asthma: a 52-week, open-label, stratified, parallel-group, multicenter study. Clinical Therapeutics 2007;29:1390-402. Drug intervention study
Ref ID: 4728

(1072) Cox G, Jenkins DG. The physiological and ventilatory responses to repeated 60 s sprints following sodium citrate ingestion. Journal of Sports Sciences 1994 October;12(5):469-75. Diet Intervention or Supplement Study
Ref ID: 2227

(1073) Cox JH, Cortright RN, Dohm GL, Houmard JA. Effect of aging on response to exercise training in humans: skeletal muscle GLUT-4 and insulin sensitivity. Journal of Applied Physiology 1999 June;86(6):2019-25. Study limited to adults
Ref ID: 2020

(1074) Coyne KS, Kaplan SA, Chapple CR, Sexton CC, Kopp ZS, Bush EN, Aiyer LP, EpiLUTS Team. Risk factors and comorbid conditions associated with lower urinary tract symptoms: EpiLUTS. BJU International 2009 April;103:Suppl-32. Study limited to adults
Ref ID: 783

(1075) Coyote-Estrada N, Liliana Miranda-Lora Ar. Tratamiento farmacolÃ³gico de la obesidad en niÃ±os. (Spanish). Boletin Medico del Hospital Infantil de Mexico 2008 November;65(6):547-67. Drug intervention study
Ref ID: 3556

(1076) Cradock AL, Kawachi I, Colditz GA, Gortmaker SL, Buka SL. Neighborhood social cohesion and youth participation in physical activity in Chicago. Social Science & Medicine 2009;68(3):427-35. Not a randomized controlled trial (RCT)
Ref ID: 5222

(1077) Cradock AL, Melly SJ, Allen JG, Morris JS, Gortmaker SL. Youth destinations associated with objective measures of physical activity in adolescents. J Adolesc Health 2009 September;45(3:Suppl):Suppl-8. Not a randomized controlled trial (RCT)
Ref ID: 692

(1078) Craig IS, Morgan DW. Relationship between 800-m running performance and accumulated oxygen deficit in middle-distance runners. Medicine & Science in Sports & Exercise 1998 November;30(11):1631-6. Study limited to adults
Ref ID: 2075

(1079) Craigie AM, Macleod M, Barton KL, Treweek S, Anderson AS, WeighWell team. Supporting postpartum weight loss in women living in deprived communities: design implications for a randomised control trial. European Journal of Clinical Nutrition 2011 August;65(8):952-8. Study limited to adults
Ref ID: 1093

(1080) Crawford D, Cleland V, Timperio A, Salmon J, Andrianopoulos N, Roberts R, Giles-Corti B, Baur L, Ball K. The longitudinal influence of home and neighbourhood environments on children's body mass index and physical activity over 5 years: the CLAN study. International Journal of Obesity 2010 July;34(7):1177-87. Not an exercise intervention study
Ref ID: 493

(1081) Crawford PB, Gosliner W, Strode P, Samuels SE, Burnett C, Craypo L, Yancey AK. Walking the talk: Fit WIC wellness programs improve self-efficacy in pediatric obesity prevention counseling. American Journal of Public Health 2004 September;94(9):1480-5. Survey or questionnaire
Ref ID: 1594

(1082) Cremonini F, Camilleri M, Clark MM, Beebe TJ, Locke GR, Zinsmeister AR, Herrick LM, Talley NJ. Associations among binge eating behavior patterns and gastrointestinal symptoms: a population-based study.[Erratum appears in Int J Obes (Lond). 2010 Jan;34(1):214]. International Journal of Obesity 2009 March;33(3):342-53. Diet Intervention Study
Ref ID: 787

(1083) Crider LB, Hall AK. Street Wise Part 1: Promoting Safe Bicycling and Walking to School. Teaching Elementary Physical Education 2005 May 1;16(3):8-11. Editorial or letter or comment
Ref ID: 3899

(1084) Criswell D, Powers S, Lawler J, Tew J, Dodd S, Iryiboz Y, Tulley R, Wheeler K. Influence of a carbohydrate-electrolyte beverage on performance and blood homeostasis during recovery from football. International Journal of Sport Nutrition 1991 June;1(2):178-91. Diet Intervention or Supplement Study
Ref ID: 2294

(1085) Crocker MK, Yanovski JA. Pediatric Obesity: Etiology and Treatment. Endocrinol Metab Clin North Am 2009;38(3):525-48. Review article
Ref ID: 3210

(1086) Croti UA, Beani L, Moscardini AC, Souza Júnior AS, Souza AS, Sobrinho SH, De Marchi CH, Godoy MFd, Braile DM. Tomografia computadorizada na avaliação tardia do tratamento cirúrgico da conexão anômala total de veias pulmonares^ipt
Computed tomography in late evaluation of surgical treatment of pulmonary veins total anomalous connection^ien. Rev bras cir cardiovasc 2011 December;26(4):532-43. Not an exercise intervention study
Ref ID: 4079

(1087) Crum AJ, Langer EJ. Mind-set matters: exercise and the placebo effect. Psychological Science 2007 February;18(2):165-71. Study limited to adults
Ref ID: 1248

(1088) Cruvinel MGC, Bittencourt PFS, Costa JRdR, Barbosa PRV. Volume gástrico residual e risco de aspiração pulmonar em crianças com refluxo gastroesofágico: estudo comparativo^ipt
Residual gastric volume and risk for pulmonary aspiration in children with gastroesophageal reflux: comparative study^ien. Rev bras anestesiol 2004 February;54(1):37-42. Not an exercise intervention study
Ref ID: 713

(1089) Culnane M, Fowler M, Lee SS, McSherry G, Brady M, O'Donnell K, Mofenson L, Gortmaker SL, Shapiro DE, Scott G, Jimenez E, Moore EC, Diaz C, Flynn PM, Cunningham B, Oleske J. Lack of long-term effects of in utero exposure to zidovudine among uninfected children born to HIV-infected women. Pediatric AIDS Clinical Trials Group Protocol 219/076 Teams. JAMA : the journal of the American Medical Association 1999;281:151-7. Cohort Study
Ref ID: 4729

(1090) Cumbá Abréu Cdl, Betancourt Vega CO, Díaz Castrillo O, Pommerenck Martínez CW. Capacidad física de trabajo en niños asmáticos. Rev cuba hig epidemiol 1986 September;24(3):353-61. Not an exercise intervention study
Ref ID: 4080

(1091) Cummings DE, Merriam GR. Growth Hormone Therapy in Adults. 54, 513-533. 2003. Review article,
Ref ID: 3211

(1092) Cundiff DK, Nigg CR. Diet and diabetic retinopathy: insights from the Diabetes Control and Complications Trial (DCCT). Medgenmed [Computer File]: Medscape General Medicine 2005;7(1):3. Lifestyle Intervention
Ref ID: 1438

(1093) Cuneo RC, Salomon F, Wiles CM, Hesp R, Sonksen PH. Growth hormone treatment in growth hormone-deficient adults. II. Effects on exercise performance. Journal of Applied Physiology 1991 February;70(2):695-700. Study limited to adults
Ref ID: 2301

(1094) Cunha AMRd, Lemônica L. Incidência da síndrome da mama fantasma e suas características clínicas^ipt. Rev bras mastologia 2002 December;12(4):29-38. Retrospective study
Ref ID: 4081

(1095) Cunha MT, Santos ACd, Silva GFC, Oehlmeyer KD, Baldo TMI. Teste de caminhada de seis minutos (TC6') em criança obesa: relato de caso^ipt. Pediatria (São Paulo) 2009 September;31(3):214-8. Case-Control / Case Study
Ref ID: 4082

(1096) Cunningham PM, Brennan D, O'Connell M, MacMahon P, O'Neill P, Eustace S. Patterns of bone and soft-tissue injury at the symphysis pubis in soccer players: observations at MRI. AJR American Journal of Roentgenology 2007 March;188(3):W291-W296. Not an exercise intervention study
Ref ID: 1272

(1097) Cusick A, McIntyre S, Novak I, Lannin N, Lowe K. A comparison of goal attainment scaling and the Canadian Occupational Performance Measure for paediatric rehabilitation research. Pediatric rehabilitation 2006;9:149-57. No non-intervention control group
Ref ID: 4730

(1098) Cutler JA. Randomized clinical trials of weight reduction in nonhypertensive persons. Annals of Epidemiology 1991;1:363-70. Review article
Ref ID: 4731

(1099) D'Adamo E, Chiarelli F, Mohn A. Treatment of non-alcoholic fatty liver disease (NAFLD) in the paediatric population. Recent Pat Endocr Metab Immune Drug Discov 2009;3(2):94-101. Review article
Ref ID: 3212

(1100) D'Anci KE. Nutrition Updates. Nutrition Reviews 2008 January;66(1):60-3. Abstract
Ref ID: 3557

(1101) D'Argent J. Gastric electrical stimulation as therapy of morbid obesity: preliminary results from the French study. Obesity Surgery 2002 April;12:Suppl-25S. Not an exercise intervention study
Ref ID: 1839

(1102) D'Avanzo B, Nanni O, La VC, Franceschi S, Negri E, Giacosa A, Conti E, Montella M, Talamini R, Decarli A. Physical activity and breast cancer risk. Cancer Epidemiology, Biomarkers & Prevention 1996 March;5(3):155-60. Case-Control / Case Study
Ref ID: 2163

(1103) D'Hondt E, Segers V, Deforche B, Shultz SP, Tanghe A, Gentier I, De Bourdeaudhuij I, De Clercq D, Lenoir M. The role of vision in obese and normal-weight children's gait control. Gait & Posture 2011;33(2):179-84. Not All Participants were Overweight and/or Obese
Ref ID: 5224

(1104) D'hooge R, Hellinckx T, Van LC, Stegen S, De SJ, Van AS, Dewolf D, Calders P. Influence of combined aerobic and resistance training on metabolic control, cardiovascular fitness and quality of life in adolescents with type 1 diabetes: a randomized controlled trial. Clinical Rehabilitation 25(4):349-59, 2011 Apr 2011;(4):349-59. Not All Participants were Overweight and/or Obese
Ref ID: 2927

(1105) Da Silva ME, Fernandez JM, Castillo E, Nunez VM, Vaamonde DM, Poblador MS, Lancho JL. Influence of vibration training on energy expenditure in active men. Journal of Strength & Conditioning Research 21(2):470-5, 2007 May 2007;(2):470-5. No control group (NC)
Ref ID: 2928

(1106) Dahlkoetter J, Callahan EJ, Linton J. Obesity and the unbalanced energy equation: exercise versus eating habit change. Journal of Consulting & Clinical Psychology 1979 October;47(5):898-905. Not All Participants were Overweight and/or Obese
Ref ID: 2380

(1107) Dai J, Jiang Z, Zhang B. [Exercise and nutrition therapy for simple obesity in children]. Chinese Journal of Clinical Rehabilitation 2006;10:20-2. Primary outcome(s) not assessed
Ref ID: 4732

(1108) Dale D, Corbin CB, Dale KS. Restricting opportunities to be active during school time: do children compensate by increasing physical activity levels after school? Research quarterly for exercise and sport 2000;71:240-8. Not a randomized controlled trial (RCT)
Ref ID: 4733

(1109) Daley AJ, Mutrie N, Crank H, Coleman R, Saxton J. Exercise therapy in women who have had breast cancer: design of the Sheffield women's exercise and well-being project. Health Education Research 2004 December;19(6):686-97. Study limited to adults
Ref ID: 1570

(1110) Daley AJ, Copeland RJ, Wright NP, Wales JK. Protocol for: Sheffield Obesity Trial (SHOT): a randomised controlled trial of exercise therapy and mental health outcomes in obese adolescents [ISRCNT83888112]. BMC Public Health 2005;5:113. Description of study from review or magazine or etc. (not the actual study)
Ref ID: 1440

(1111) Daley AJ, Copeland RJ, Wright NP, Wales JK. 'I can actually exercise if I want to; it isn't as hard as I thought': a qualitative study of the experiences and views of obese adolescents participating in an exercise therapy intervention. J Health Psychol 2008 September;13(6):810-9. Not a randomized controlled trial (RCT)
Ref ID: 162

(1112) Dallocchio C, Arbasino C, Klersy C, Marchioni E. The effects of physical activity on psychogenic movement disorders. Mov Disord 2010;25(4):421-5. Study limited to adults
Ref ID: 3213

(1113) Daly HB. Laboratory Rat Experiments Show Consumption of Lake-Ontario Salmon Causes Behavioral-Changes - Support for Wildlife and Human Research Results. Journal of Great Lakes Research 1993;19(4):784-8. Animal study
Ref ID: 5225

(1114) Damiani D. Uso de hormônio de crescimento na síndrome de Prader-Willi: [revisão]^ipt. Arq bras endocrinol metab 2008 July;52(5):833-8. Review article
Ref ID: 4083

(1115) Damiano DL, Dodd K, Taylor NF. Should we be testing and training muscle strength in cerebral palsy? Dev Med Child Neurol 2002;44:68-72. Review article
Ref ID: 4734

(1116) Damsgaard CT, Molgaard C, Matthiessen J, Gyldenlove SN, Lauritzen L. The effects of n-3 long-chain polyunsaturated fatty acids on bone formation and growth factors in adolescent boys. Pediatric Research 2012;71(6):713-9. Diet Intervention or Supplement Study
Ref ID: 5226

(1117) Danda AK, -S-R, Chinnaswami R. Comparison of gap arthroplasty with and without a temporalis muscle flap for the treatment of ankylosis. Journal of oral and maxillofacial surgery : official journal of the American Association of Oral and Maxillofacial Surgeons 2009;67:1425-31. Not an exercise intervention study
Ref ID: 4735

(1118) Danel C, Moh R, Minga A, Anzian A, Ba-Gomis O, Kanga C, Nzunetu G, Gabillard D, Rouet Fo, Sorho S, Chaix ML, EholiÃ© S, Menan H, Sauvageot D, Bissagnene E, Salamon R, Anglaret X. CD4-guided structured antiretroviral treatment interruption strategy in HIV-infected adults in west Africa (Trivacan ANRS 1269 trial): a randomised trial. Lancet 2006 June 17;367(9527):1981-9. Study limited to adults
Ref ID: 3558

(1119) Dangi CBS, Firodiya A. TRIPLE-NEGATIVE BREAST CANCER AND IT'S THERAPEUTIC OPTIONS. International Journal of Pharma & Bio Sciences 2012 April 13;3(2):B. Not an exercise intervention study
Ref ID: 3559

(1120) Daniels S. Pharmacological treatment of obesity in paediatric patients. [Review] [33 refs]. Paediatric Drugs 2001;3(6):405-10. Review article
Ref ID: 1908

(1121) Daniels SR, Greer FR. Lipid screening and cardiovascular health in childhood. Pediatrics 2008;122(1):198-208. Review article
Ref ID: 3214

(1122) Danis A, Kyriazis Y, Klissouras V. The effect of training in male prepubertal and pubertal monozygotic twins. European Journal of Applied Physiology 2003 May;89(3-4):309-18. Not a randomized controlled trial (RCT)
Ref ID: 1749

(1123) Dantas PROF, Lira FAS, Borba VVL, Costa MJC, Trombetta IC, Santos MSB, Santos AC. Vitamin C restores blood pressure and vasodilator response during mental stress in obese children. Arq Bras Cardiol 2011;96(6):490-7. Diet Intervention or Supplement Study
Ref ID: 3215

(1124) Dapi LN, Hornell A, Janlert U, Stenlund H, Larsson C. Energy and nutrient intakes in relation to sex and socio-economic status among school adolescents in urban Cameroon, Africa. Public Health Nutrition 2011 May;14(5):904-13. Cross-sectional study
Ref ID: 2477

(1125) Daray LA, Henagan TM, Zanovec M, Earnest CP, Johnson LG, Winchester J, Tuuri G, Stewart LK. Endurance and resistance training lowers C-reactive protein in young, healthy females. Applied Physiology, Nutrition, & Metabolism = Physiologie Appliquee, Nutrition et Metabolisme 2011 October;36(5):660-70. Study limited to adults
Ref ID: 2478

(1126) Datar A, Sturm R. Physical education in elementary school and body mass index: Evidence from the early childhood longitudinal study. American Journal of Public Health 2004;94:1501-6. Not All Participants were Overweight and/or Obese
Ref ID: 4736

(1127) Davidson ZE, Truby H. A review of nutrition in Duchenne muscular dystrophy. J Hum Nutr Diet 2009;22(5):383-93. Review article
Ref ID: 3216

(1128) Davis AM, James RL, Boles RE, Goetz JR, Belmont J, Malone B. The use of TeleMedicine in the treatment of paediatric obesity: feasibility and acceptability. Matern Child Nutr 2011 January;7(1):71-9. No exercise only group
Ref ID: 9

(1129) Davis B, Carpenter C. Proximity of Fast-Food Restaurants to Schools and Adolescent Obesity. American Journal of Public Health 2009 March;99(3):505-10. Diet Intervention or Supplement Study
Ref ID: 3810

(1130) Davis C, Kennedy SH, Ravelski E, Dionne M. The role of physical activity in the development and maintenance of eating disorders. Psychological Medicine 1994 November;24(4):957-67. Not a randomized controlled trial (RCT)
Ref ID: 2221

(1131) Davis CE, Hunsberger S, Murray DM, Fabsitz RR, Himes JH, Stephenson LK, Caballero B, Skipper B. Design and statistical analysis for the Pathways study. American Journal of Clinical Nutrition 1999 April;69(4:Suppl):Suppl-763S. Description of study from review or magazine or etc. (not the actual study)
Ref ID: 2037

(1132) Davis CL, Tkacz J, Gregoski M, Boyle CA, Lovrekovic G. Aerobic exercise and snoring in overweight children: a randomized controlled trial. Obesity (Silver Spring) 2006 November;14(11):1985-91. Same subjects as another study already included
Ref ID: 259

(1133) Davis CL, Tomporowski PD, Boyle CA, Waller JL, Miller PH, Naglieri JA, Gregoski M. Effects of aerobic exercise on overweight children's cognitive functioning: a randomized controlled trial. Res Q Exerc Sport 2007 December;78(5):510-9. Same subjects as another study already included
Ref ID: 196

(1134) Davis CL, Tomporowski PD, McDowell JE, Austin BP, Miller PH, Yanasak NE, Allison JD, Naglieri JA. Exercise improves executive function and achievement and alters brain activation in overweight children: a randomized, controlled trial. Health Psychol 2011 January;30(1):91-8. Same subjects as another study already included
Ref ID: 442

(1135) Davis CL, Cooper S. Fitness, fatness, cognition, behavior, and academic achievement among overweight children: do cross-sectional associations correspond to exercise trial outcomes? Preventive Medicine 2011 June;52:Suppl-9. Cross-sectional study
Ref ID: 1032

(1136) Davis JN, Tung A, Chak SS, Ventura EE, Byrd-Williams CE, Alexander KE, Lane CJ, Weigensberg MJ, Spruijt-Metz D, Goran MI. Aerobic and strength training reduces adiposity in overweight Latina adolescents. Med Sci Sports Exerc 2009 July;41(7):1494-503. No exercise only group
Ref ID: 113

(1137) Davis JN, Kelly LA, Lane CJ, Ventura EE, Byrd-Williams CE, Alexandar KA, Azen SP, Chou CP, Spruijt-Metz D, Weigensberg MJ, Berhane K, Goran MI. Randomized control trial to improve adiposity and insulin resistance in overweight Latino adolescents. Obesity 2009 August;17(8):1542-8. No exercise only group
Ref ID: 704

(1138) Davis JN, Ventura EE, Shaibi GQ, Byrd-Williams CE, Alexander KE, Vanni AK, Meija MR, Weigensberg MJ, Spruijt-Metz D, Goran MI. Interventions for improving metabolic risk in overweight Latino youth. [Review]. International Journal of Pediatric Obesity 2010 October;5(5):451-5. Review article
Ref ID: 2481

(1139) Davis JN, Gyllenhammer LE, Vanni AA, Meija M, Tung A, Schroeder ET, Spruijt-Metz D, Goran MI. Startup circuit training program reduces metabolic risk in Latino adolescents. Medicine & Science in Sports & Exercise 2011 November;43(11):2195-203. Primary outcome(s) not assessed
Ref ID: 2482

(1140) Davis JN, Ventura EE, Tung A, Munevar MA, Hasson RE, Byrd-Williams C, Vanni AK, Spruijt-Metz D, Weigensberg M, Goran MI. Effects of a randomized maintenance intervention on adiposity and metabolic risk factors in overweight minority adolescents. Pediatric Obesity 2012 February;7(1):16-27. Follow-up Study
Ref ID: 2483

(1141) Davis KL, Kang M, Boswell BB, DuBose KD, Altman SR, Binkley HM. Validity and reliability of the medicine ball throw for kindergarten children. Journal of Strength & Conditioning Research 2008 November;22(6):1958-63. Not an exercise intervention study
Ref ID: 866

(1142) Davis PJ, McGowan FX, Landsman I, Maloney K, Hoffmann P. Effect of antiemetic therapy on recovery and hospital discharge time. A double-blind assessment of ondansetron, droperidol, and placebo in pediatric patients undergoing ambulatory surgery. Anesthesiology 1995;83:956-60. Drug intervention study
Ref ID: 4737

(1143) Davis PJ, Greenberg JA, Gendelman M, Fertal K. Recovery characteristics of sevoflurane and halothane in preschool-aged children undergoing bilateral myringotomy and pressure equalization tube insertion. Anesthesia and analgesia 1999;88:34-8. Drug intervention study
Ref ID: 892

(1144) Davis SM, Clay T, Smyth M, Gittelsohn J, Arviso V, Flint-Wagner H, Rock BH, Brice RA, Metcalfe L, Stewart D, Vu M, Stone EJ. Pathways curriculum and family interventions to promote healthful eating and physical activity in American Indian schoolchildren. Prev Med 2003 December;37(6 Pt 2):S24-S34. Primary outcome(s) not assessed
Ref ID: 348

(1145) Davison GW, Ashton T, George L, Young IS, McEneny J, Davies B, Jackson SK, Peters JR, Bailey DM. Molecular detection of exercise-induced free radicals following ascorbate prophylaxis in type 1 diabetes mellitus: a randomised controlled trial. Diabetologia 2008 November;51(11):2049-59. Diet Intervention or Supplement Study
Ref ID: 882

(1146) Davison K, Coates AM, Buckley JD, Howe PR. Effect of cocoa flavanols and exercise on cardiometabolic risk factors in overweight and obese subjects. Int J Obes (Lond) 2008 August;32(8):1289-96. Study limited to adults
Ref ID: 181

(1147) Davy BM, Harrell K, Stewart J, King DS. Body weight status, dietary habits, and physical activity levels of middle school-aged children in rural Mississippi. Southern Medical Journal 2004 June;97(6):571-7. Cross-sectional study
Ref ID: 1608

(1148) Dawson B, Vladich T, Blanksby BA. Effects of 4 weeks of creatine supplementation in junior swimmers on freestyle sprint and swim bench performance. Journal of Strength & Conditioning Research 2002 November;16(4):485-90. Diet Intervention or Supplement Study
Ref ID: 1794

(1149) Dâmaso AR, Teixeira LR, Nascimento CMO. Obesidade: subsídios para o desenvolvimento de atividades motoras. Rev paul educ fís 1994 June;8(1):98-111. Review article
Ref ID: 4084

(1150) Dâmaso AR, Teixeira LR, Curi CMOdN. Atividades motoras na obesidade. In: Fisberg M, editor. Obesidade na infância e adolescência.Säo Paulo: Fundo Editorial Byk; 1995. p. 91-9.¬

Review article
Ref ID: 4085

(1151) Dämon S, Dietwch S, Widhalm K. PRESTO--Prevention Study of Obesity: a project to prevent obesity during childhood and adolescence. Acta Paediatr Suppl 2005;94:47-8. Lifestyle Intervention
Ref ID: 4738

(1152) De-Windt AC, Asehnoune K, Roquilly A, Guillaud C, Le RC, Pinaud M, Lejus C. An opioid-free anaesthetic using nerve blocks enhances rapid recovery after minor hand surgery in children. European journal of anaesthesiology 2010;27:521-5. Drug intervention study
Ref ID: 4739

(1153) de Alwis NMW, Day CP. Current and future therapeutic strategies in NAFLD. Curr Pharm Des 2010;16(17):1958-62. Review article
Ref ID: 3217

(1154) de Backer TLM, Smedema JP, Carlier SG. Current Management of Primary Pulmonary Hypertension. BioDrugs 2001 December;15(12):801-17. Review article
Ref ID: 3560

(1155) De Bruyne RML, Fitzpatrick E, Dhawan A. Fatty liver disease in children: Eat now pay later. Hepatol Int 2010;4(1):375-85. Review article
Ref ID: 3218

(1156) de Carvalho MV, Marins JC, Silami-Garcia E. The influence of water versus carbohydrate-electrolyte hydration on blood components during a 16-km military march. Military Medicine 2007 January;172(1):79-82. Diet Intervention or Supplement Study
Ref ID: 1281

(1157) De Gáspari JC, Schwartz GM. Vivências em arte circense: motivos de aderência e expectativas^ipt. Motriz rev educ fís (Impr ) 2007;13(3):158-64. Not a randomized controlled trial (RCT)
Ref ID: 4086

(1158) de Groot JF, Takken T, van BM, Gooskens R, Schoenmakers M, Versteeg C, Vanhees L, Helders P. Randomized controlled study of home-based treadmill training for ambulatory children with spina bifida. Neurorehabilitation & Neural Repair 2011 September;25(7):597-606. Not All Participants were Overweight and/or Obese
Ref ID: 2484

(1159) de Heer HD, Koehly L, Pederson R, Morera O. Effectiveness and spillover of an after-school health promotion program for Hispanic elementary school children. American Journal of Public Health 2011 October;101(10):1907-13. Not All Participants were Overweight and/or Obese
Ref ID: 2485

(1160) De Hert M, Dobbelaere M, Sheridan EM, Cohen D, Correll CU. Metabolic and endocrine adverse effects of second-generation antipsychotics in children and adolescents: A systematic review of randomized, placebo controlled trials and guidelines for clinical practice. Eur Psychiatry 2011;26(3):144-58. Review article
Ref ID: 3219

(1161) De Jongh S, Ose L, Szamosi T, GagnÃ© C, Lambert M, Scott R, Perron P, Dobbelaere D, Saborio M, Tuohy MB, Stepanavage M, Sapre A, Gumbiner B, Mercuri M, Van Trotsenburg ASP, Bakker HD, Kastelein JJP. Efficacy and safety of statin therapy in children with familial hypercholesterolemia: A randomized, double-blind, placebo-controlled trial with simvastatin. Circulation 2002;106(17):2231-7. Drug intervention study
Ref ID: 3220

(1162) de Lima SP, de Mello MT, Elias N, Fonseca FA, de PA, Carnier J, Oyama LM, Tock L, Tufik S, Damaso AR. Improvement in HOMA-IR is an independent predictor of reduced carotid intima-media thickness in obese adolescents participating in an interdisciplinary weight-loss program. Hypertension Research - Clinical & Experimental 2011 February;34(2):232-8. Lifestyle Intervention
Ref ID: 2486

(1163) De Lorenzo A, Bertini I, Iacopino L, Pagliato E, Testolin C, Testolin G. Body composition measurement in highly trained male athletes - A comparison of three methods. Journal of Sports Medicine and Physical Fitness 2000;40(2):178-83. Not All Participants were Overweight and/or Obese
Ref ID: 5227

(1164) de Meij JS, Chinapaw MJ, van Stralen MM, van der Wal MF, van DL, van MW. Effectiveness of JUMP-in, a Dutch primary school-based community intervention aimed at the promotion of physical activity. British Journal of Sports Medicine 2011 October;45(13):1052-7. Not a randomized controlled trial (RCT)
Ref ID: 2487

(1165) de Mello ED, Luft VC, Meyer F. [Individual outpatient care versus group education programs. Which leads to greater change in dietary and physical activity habits for obese children?]. J Pediatr (Rio J) 2004 November;80(6):468-74. No exercise only group, No comparative control group
Ref ID: 317

(1166) de Mello MT, de PA, Carnier J, Sanches PL, Correa FA, Tock L, Ernandes RM, Tufik S, Damaso AR. Long-term effects of aerobic plus resistance training on the metabolic syndrome and adiponectinemia in obese adolescents. Journal of Clinical Hypertension 2011 May;13(5):343-50. No non-intervention control group
Ref ID: 2488

(1167) de Onis M, Garza C, Onyango AW, Rolland-Cachera MF. WHO growth standards for infants and young children. Archives de Pediatrie 2009;16(1):47-53. Not an exercise intervention study
Ref ID: 5228

(1168) De Palo EF, Gatti R, Lancerin F, Cappellin E, Solda G, De Palo CB, Spinella P. Urinary insulin-like growth factor I in athletes, before and after physical exercise, and in sedentary subjects. Clinica Chimica Acta 2002;322(1-2):51-7. Not an exercise intervention study
Ref ID: 5229

(1169) de Ramirez SS, Enquobahrie DA, Nyadzi G, Mjungu D, Magombo F, Ramirez M, Sachs SE, Willett W. Prevalence and correlates of hypertension: a cross-sectional study among rural populations in sub-Saharan Africa. Journal of Human Hypertension 2010 December;24(12):786-95. Cross-sectional study
Ref ID: 2489

(1170) De Ravel TJL, Swillen A, Willekens D, Descheemaeker MJ, Govers V, Borghgraef M, Vermeesch JR, Fryns JP. Molecular karyotyping is important in determining the cause of behavioural phenotypes. Journal of Intellectual Disability Research 2008 October;52(10):813. Case-Control / Case Study
Ref ID: 3811

(1171) de Silva-Sanigorski AM, Bell AC, Kremer P, Nichols M, Crellin M, Smith M, Sharp S, de GF, Carpenter L, Boak R, Robertson N, Swinburn BA. Reducing obesity in early childhood: results from Romp & Chomp, an Australian community-wide intervention program. American Journal of Clinical Nutrition 2010 April;91(4):831-40. Not a randomized controlled trial (RCT)
Ref ID: 568

(1172) De Souza FMB, Pereira RP, Minuque NP, Do Carmo CM, De Mello MHM, Villaca P, Tanaka C. Postural adjustment after an unexpected perturbation in children with haemophilia. Haemophilia 2012;18(3):e311-e315. Not a randomized controlled trial (RCT)
Ref ID: 5230

(1173) De Ste Croix MB, Armstrong N, Chia MY, Welsman JR, Parsons G, Sharpe P. Changes in short-term power output in 10- to 12-year-olds. Journal of Sports Sciences 2001 February;19(2):141-8. Not a randomized controlled trial (RCT)
Ref ID: 1933

(1174) De Vitta A, Madrigal C, Sales VS. Peso corporal e peso do material escolar transportado por crianças em idade escolar. Fisioter mov 2003 June;16(2):55-60. Cross-sectional study
Ref ID: 709

(1175) De Vitta A, Martinez MG, Piza NT, Simeão SFdAP, Ferreira NP. Prevalência e fatores associados à dor lombar em escolares^ipt
Prevalence of lower back pain and associated factors in students^ien. Cad saúde pública 2011 August;27(8):1520-8. Cross-sectional study
Ref ID: 4087

(1176) de Vries C, Garneau CJ, Nadadur G, Parkinson MB. Considering Secular and Demographic Trends in Designing Long Lifetime Products for Target User Populations. Journal of Mechanical Design 2011;133(8). Not an exercise intervention study
Ref ID: 5231

(1177) de Zoysa NS, Jayaweera KK, Vaithianathan T. Manual plasmapheresis in the treatment of Guillain-Barre syndrome. Journal of Clinical Apheresis 1994;9(2):147-50. Drug intervention study
Ref ID: 2228

(1178) De BF, Fischer JE, Hoffmann K, Renz-Polster H. A participatory parent-focused intervention promoting physical activity in preschools: design of a cluster-randomized trial. BMC Public Health 2010;10:49. Description of study from review or magazine or etc. (not the actual study)
Ref ID: 573

(1179) de BS, Mathern GW, Bookheimer S, Dobkin B. Locomotor training remodels fMRI sensorimotor cortical activations in children after cerebral hemispherectomy. Neurorehabilitation & Neural Repair 2007 November;21(6):497-508. Study less than 4 weeks
Ref ID: 1152

(1180) De CK, Ottevaere C, Sjostrom M, Moreno LA, Warnberg J, Valtuena J, Manios Y, Dietrich S, Mauro B, Artero EG, Molnar D, Hagstromer M, Ruiz JR, Sarri K, Kafatos A, Gottrand F, de HS, Maes L, De B, I, HELENA Study Group. Self-reported physical activity in European adolescents: results from the HELENA (Healthy Lifestyle in Europe by Nutrition in Adolescence) study. Public Health Nutrition 2011 February;14(2):246-54. Cross-sectional study
Ref ID: 2490

(1181) De CC, Malinow MR, van Kranenburg GP, Geurten PG, Longford NT, Keizer HA. Influence of exercise and menstrual cycle phase on plasma homocyst(e)ine levels in young women--a prospective study. Scandinavian Journal of Medicine & Science in Sports 1999 October;9(5):272-8. Acute study
Ref ID: 1998

(1182) de GL, de-Groot CJ, Hopkins B. An instrument to measure independent walking: are there differences between preterm and fullterm infants? Journal of Child Neurology 1997;12:37-41. Subjects less than 2 years old
Ref ID: 934

(1183) de GS, Dallmeijer AJ, Post MW, Angenot EL, van den Berg-Emons RJ, van der Woude LH. Prospective analysis of lipid profiles in persons with a spinal cord injury during and 1 year after inpatient rehabilitation. Archives of Physical Medicine & Rehabilitation 2008 March;89(3):531-7. Not an exercise intervention study
Ref ID: 994

(1184) de GS, van der Woude LH, Niezen A, Smit CA, Post MW. Evaluation of the physical activity scale for individuals with physical disabilities in people with spinal cord injury. Spinal Cord 2010 July;48(7):542-7. Cross-sectional study
Ref ID: 501

(1185) de JW, van-Aalderen WM, Kraan J, Koëter GH, van der Schans CP. Inspiratory muscle training in patients with cystic fibrosis. Respir Med 2001;95:31-6. Not a randomized controlled trial (RCT)
Ref ID: 4740

(1186) De LM, Segato G, Busetto L, Favretti F, Aigner F, Weiss H, de GC, Gaggiotti G, Himpens J, Limao J, Scheyer M, Toppino M, Zurmeyer EL, Bottani G, Penthaler H. Progress in implantable gastric stimulation: summary of results of the European multi-center study. Obesity Surgery 2004 September;14:Suppl-9. Study limited to adults
Ref ID: 1582

(1187) De SJ, Van den Broeck M, Jonckheer MH. Study of lumbar spine bone mineral density in obese children. Acta Paediatrica 1995 March;84(3):313-5. Not an exercise intervention study
Ref ID: 2207

(1188) Debanne T, Laffaye G. Predicting the throwing velocity of the ball in handball with anthropometric variables and isotonic tests. Journal of Sports Sciences 2011 April;29(7):705-13. Study limited to adults
Ref ID: 2491

(1189) DeBar LL, Ritenbaugh C, Vuckovic N, Stevens VJ, Aickin M, Elliot D, Moe E, Orwoll E, Ernst D, Irving LM. YOUTH: decisions and challenges in designing an osteoporosis prevention intervention for teen girls. Preventive Medicine 2004 November;39(5):1047-55. Description of study from review or magazine or etc. (not the actual study)
Ref ID: 1583

(1190) DeBar LL, Ritenbaugh C, Aickin M, Orwoll E, Elliot D, Dickerson J, Vuckovic N, Stevens VJ, Moe E, Irving LM. Youth: a health plan-based lifestyle intervention increases bone mineral density in adolescent girls.[Erratum appears in Arch Pediatr Adolesc Med. 2007 Feb;161(2):130]. Archives of Pediatrics & Adolescent Medicine 2006 December;160(12):1269-76. Not All Participants were Overweight and/or Obese
Ref ID: 1316

(1191) DeBar LL, Dickerson J, Clarke G, Stevens VJ, Ritenbaugh C, Aickin M. Using a website to build community and enhance outcomes in a group, multi-component intervention promoting healthy diet and exercise in adolescents. Journal of Pediatric Psychology 2009 June;34(5):539-50. Lifestyle Intervention
Ref ID: 748

(1192) DeBar LL, Schneider M, Ford EG, Hernandez AE, Showell B, Drews KL, Moe EL, Gillis B, Jessup AN, Stadler DD, White M, HEALTHY study group. Social marketing-based communications to integrate and support the HEALTHY study intervention. International Journal of Obesity 2009 August;33:Suppl-9. Cohort Study
Ref ID: 710

(1193) DeBar LL, Schneider M, Drews KL, Ford EG, Stadler DD, Moe EL, White M, Hernandez AE, Solomon S, Jessup A, Venditti EM, HEALTHY study group. Student public commitment in a school-based diabetes prevention project: impact on physical health and health behavior. BMC Public Health 2011;11:711. Review article
Ref ID: 2492

(1194) DeBar LL, Stevens VJ, Perrin N, Wu P, Pearson J, Yarborough BJ, Dickerson J, Lynch F. A primary care-based, multicomponent lifestyle intervention for overweight adolescent females. Pediatrics 2012 March;129(3):e611-e620. Lifestyle Intervention
Ref ID: 1042

(1195) Deconinck FJA, De Clercq D, Van Coster R, Oostra A, Dewitte G, Savelsbergh GJR, Cambier D, Lenoir M. Sensory contributions to balance in boys with developmental coordination disorder. Adapted Physical Activity Quarterly 2008;25(1):17-35. Not a randomized controlled trial (RCT)
Ref ID: 5232

(1196) Dedoussis GV, Yannakoulia M, Timpson NJ, Manios Y, Kanoni S, Scott RA, Papoutsakis C, Deloukas P, Pitsiladis YP, Davey-Smith G, Hirschhorn JN, Lyon HN. Does a short breastfeeding period protect from FTO-induced adiposity in children? International Journal of Pediatric Obesity 2011 June;6(2-2):e326-e335. Cohort Study
Ref ID: 2494

(1197) Deforche B, De B, I, Tanghe A, Hills AP, De BP. Changes in physical activity and psychosocial determinants of physical activity in children and adolescents treated for obesity. Patient Education & Counseling 2004 December;55(3):407-15. Not a randomized controlled trial (RCT)
Ref ID: 1566

(1198) Deforche B, De B, I, Tanghe A, Debode P, Hills AP, Bouckaert J. Post-treatment phone contact: a weight maintenance strategy in obese youngsters. Int J Obes (Lond) 2005 May;29(5):543-6. No exercise only group
Ref ID: 314

(1199) Degoricija V, Zjacic-Rotkvic V, Marout J, Sefer S, Troskot B. Clinical and neurohumoral response to posture, physical exercise, and ascites treatment in Child-Pugh C liver cirrhosis: randomized prospective trial. Croatian Medical Journal 2003 April;44(2):178-86. Not an exercise intervention study
Ref ID: 1758

(1200) DeJongh ED, Binkley TL, Specker BL. Fat mass gain is lower in calcium-supplemented than in unsupplemented preschool children with low dietary calcium intakes. Am J Clin Nutr 2006 November;84(5):1123-7. Secondary analysis, No exercise only group
Ref ID: 261

(1201) del RÃ­o-Navarro BE, Hidalgo-Castro EMa, Luis Sienra-Monge JJ. Asma. (Spanish). Boletin Medico del Hospital Infantil de Mexico 2009 January;66(1):3-33. Review article
Ref ID: 3561

(1202) del Rio-Navarro B, Cisneros-Rivero M, Berber-Eslava A, Espinola-Reyna G, Sienra-Monge J. Exercise induced bronchospasm in asthmatic and non-asthmatic obese children. Allergologia et Immunopathologia 2000 January;28(1):5-11. Acute study
Ref ID: 1984

(1203) del Valle MF, Perez M, Santana-Sosa E, Fiuza-Luces C, Bustamante-Ara N, Gallardo C, Villasenor A, Graell M, Morande G, Romo GR, Lopez-Mojares LM, Ruiz JR, Lucia A. Does resistance training improve the functional capacity and well being of very young anorexic patients? A randomized controlled trial. J Adolesc Health 2010 April;46(4):352-8. Not All Participants were Overweight and/or Obese
Ref ID: 565

(1204) Delahanty LM, Nathan DM, Lachin JM, Hu FB, Cleary PA, Ziegler GK, Wylie-Rosett J, Wexler DJ, Diabetes Control and Complications Trial/Epidemiology of Diabetes. Association of diet with glycated hemoglobin during intensive treatment of type 1 diabetes in the Diabetes Control and Complications Trial. American Journal of Clinical Nutrition 2009 February;89(2):518-24. Cross-sectional study
Ref ID: 812

(1205) DeLany JP, Bray GA, Harsha DW, Volaufova J. Energy expenditure in preadolescent African American and white boys and girls: the Baton Rouge Children's Study. American Journal of Clinical Nutrition 2002;75(4):705-13. Cohort Study
Ref ID: 5233

(1206) Delgado HL, Hurtado E. Crecimiento físico y menarquia en adolescentes de Guatemala. Arch latinoam nutr 1990 December;40(4):503-17. Cross-sectional study
Ref ID: 817

(1207) Delisle H, Agueh V, Fayomi B. Partnership research on nutrition transition and chronic diseases in West Africa - trends, outcomes and impacts. Bmc International Health and Human Rights 2011;11. Cross-sectional study
Ref ID: 5234

(1208) Dell'Agnello G, Maschietto D, Bravaccio C, Calamoneri F, Masi G, Curatolo P, Besana D, Mancini F, Rossi A, Poole L, Escobar R, Zuddas A, -LYCY-Study-Group. Atomoxetine hydrochloride in the treatment of children and adolescents with attention-deficit/hyperactivity disorder and comorbid oppositional defiant disorder: A placebo-controlled Italian study. European neuropsychopharmacology : the journal of the European College of Neuropsychopharmacology 2009;19:822-34. Drug intervention study
Ref ID: 4741

(1209) Della CC, Alisi A, Iorio R, Alterio A, Nobili V. Expert opinion on current therapies for nonalcoholic fatty liver disease. [Review]. Expert Opin Pharmacother 2011 August;12(12):1901-11. Review article
Ref ID: 2495

(1210) Dellagrana RA, Silva MPd, Smolarek AdC, Bozza R, Stabelini Neto A, Campos Wd. Composição corporal, maturação sexual e desempenho motor de jovens praticantes de handebol^ipt
Body composition, sexual maturation and motor performance the young practitioners handball^ien. Motriz rev educ fís (Impr ) 2010 December;16(4):880-8. Cross-sectional study
Ref ID: 4088

(1211) Delva J, OMalley PM, Johnston LD. Health-related behaviors and overweight: a study of Latino adolescents in the United States of America^ien. Rev panam salud pública 2007 January;21(1):11-20. Cross-sectional study
Ref ID: 639

(1212) DeMattia L, Lemont L, Meurer L. Do interventions to limit sedentary behaviours change behaviour and reduce childhood obesity? A critical review of the literature. [Review] [32 refs]. Obes Rev 2007 January;8(1):69-81. Review article
Ref ID: 1286

(1213) Demeer K, Bergman R, Kusner JS, Voorhoeve HWA. Differences in Physical Growth of Aymara and Quechua Children Living at High-Altitude in Peru. American Journal of Physical Anthropology 1993;90(1):59-75. Cross-sectional study
Ref ID: 5235

(1214) Demke DM, Peters GR, Linet OI, Metzler CM, Klott KA. Effects of a fish oil concentrate in patients with hypercholesterolemia. Atherosclerosis 1988 March;70(1-2):73-80. Diet Intervention or Supplement Study
Ref ID: 2496

(1215) Demling RH, DeSanti L. The rate of restoration of body weight after burn injury, using the anabolic agent oxandrolone, is not age dependent. Burns 2001 February;27(1):46-51. No non-intervention control group
Ref ID: 1931

(1216) Demo R, Senestrari D, Ferreyra JE. [Young football players aerobic perfomance in sub-maximum exercise with exhaustion at a moderate altitude without acclimation: experience in El Condor]. [Spanish]. Revista de la Facultad de Ciencias Medicas de Cordoba 2007;64(1):8-17. Not a randomized controlled trial (RCT)
Ref ID: 1113

(1217) Dempsey RL, Mazzone MF, Meurer LN. Does oral creatine supplementation improve strength? A meta-analysis. Journal of Family Practice 2002 November;51(11):945-51. Review article
Ref ID: 1787

(1218) Demura S, Yamaji S, Goshi F, Nagasawa Y. The influence of transient change of total body water on relative body fats based on three bioelectrical impedance analyses methods. Comparison between before and after exercise with sweat loss, and after drinking. Journal of Sports Medicine & Physical Fitness 2002 March;42(1):38-44. Diet Intervention or Supplement Study
Ref ID: 1851

(1219) den Hoed M, Westerterp KR. Body composition is associated with physical activity in daily life as measured using a triaxial accelerometer in both men and women. International Journal of Obesity 2008;32(8):1264-70. Cross-sectional study
Ref ID: 5236

(1220) Denadai RC, Sigulem DM, Vítolo MR, Fisberg M, Dâmaso AR. Efeito da atividade motora sobre a composiçäo corporal, taxa metabólica basal e diária de adolescentes obesos. Rev paul pediatr 1996 December;14(4):163-8. Not a randomized controlled trial (RCT)
Ref ID: 4089

(1221) Denadai RC, Vítolo MR, Macedo AS, Teixeira L, Cezar C, Dâmaso AR. Efeitos do exercício moderado e da orientaçäo nutricional sobre a composiçäo corporal de adolescentes obesos avaliados por densitometria óssea (DEXA). Rev paul educ fís 1998 December;12(2):210-8. Lifestyle Intervention
Ref ID: 779

(1222) Derave W, Eijnde BO, Verbessem P, Ramaekers M, Van LM, Richter EA, Hespel P. Combined creatine and protein supplementation in conjunction with resistance training promotes muscle GLUT-4 content and glucose tolerance in humans. Journal of Applied Physiology 2003 May;94(5):1910-6. Diet Intervention or Supplement Study
Ref ID: 1761

(1223) Derman O, Cinemre A, Kanbur N, Dogan M, Kilic M, Karaduman E. Effect of swimming on bone metabolism in adolescents. Turkish Journal of Pediatrics 2008 March;50(2):149-54. Not a randomized controlled trial (RCT)
Ref ID: 908

(1224) Deruelle F, Baron B. Vitamin C: Is Supplementation Necessary for Optimal Health? Journal of Alternative & Complementary Medicine 2008 December;14(10):1291-8. Review article
Ref ID: 3562

(1225) Detsch C, Luz AMH, Candotti CT, Oliveira DSd, Lazaron F, Guimarães LK, Schimanoski P. Prevalência de alterações posturais em escolares do ensino médio em uma cidade no Sul do Brasil^ipt. Rev panam salud pública 2007 April;21(4):231-8. Survey or questionnaire
Ref ID: 4090

(1226) Deus RKBCd, Bustamante A, Lopes VP, Seabra AT, Silva RMGd, Maia JAR. Modelação longitudinal dos níveis de coordenação motora de crianças dos seis aos 10 anos de idade da Região Autônoma dos Açores, Portugal^ipt
Longetudinal modeling of motor coordination levels of children aged six to 10 years of age from the Autonomous Region of Azores, Portugal^ien. Rev bras educ fís esp 2010 June;24(2):259-73. Survey or questionnaire
Ref ID: 4091

(1227) Deutschbein T, Unger N, Jaeger A, Broecker-Preuss M, Mann K, Petersenn S. Influence of various confounding variables and storage conditions on metanephrine and normetanephrine levels in plasma. Clinical Endocrinology 2010 August;73(2):153-60. Not a randomized controlled trial (RCT)
Ref ID: 484

(1228) Devaney JM, Tosi LL, Fritz DT, Gordish-Dressman HA, Jiang S, Orkunoglu-Suer FE, Gordon AH, Harmon BT, Thompson PD, Clarkson PM, Angelopoulos TJ, Gordon PM, Moyna NM, Pescatello LS, Visich PS, Zoeller RF, Brandoli C, Hoffman EP, Rogers MB. Differences in fat and muscle mass associated with a functional human polymorphism in a post-transcriptional BMP2 gene regulatory element. Journal of Cellular Biochemistry 2009 August 15;107(6):1073-82. Not an exercise intervention study
Ref ID: 703

(1229) DeVault N, Kennedy T, Hermann J, Mwavita M, Rask P, Jaworsky A. It's all about kids: preventing overweight in elementary school children in Tulsa, OK. J Am Diet Assoc 2009 April;109(4):680-7. No exercise only group
Ref ID: 131

(1230) Dewan B, Balasubramanian A. Troxipide in the Management of Gastritis: A Randomized Comparative Trial in General Practice. Gastroenterology Research & Practice 2010 January;2010:1-7. Drug intervention study
Ref ID: 3563

(1231) Dewey KG, Cohen RJ, Brown KH, Rivera LL. Effects of exclusive breastfeeding for four versus six months on maternal nutritional status and infant motor development: results of two randomized trials in Honduras. Journal of Nutrition 2001 February;131(2):262-7. Subjects less than 2 years old
Ref ID: 1929

(1232) DeWolfe JA, Jack E. Weight control in adolescent girls: a comparison of the effectiveness of three approaches to follow-up. Journal of School Health 1984 October;54(9):347-9. Follow-up Study
Ref ID: 2361

(1233) Di Marzio D, Mohn A, De Martino M, Chiarelli F. Macroangiopathy in adults and children with diabetes: Risk factors (Part 2). Horm Metab Res 2006;38(11):706-20. Review article
Ref ID: 3221

(1234) Diallo O, Dore E, Duche P, Van PE. Effects of plyometric training followed by a reduced training programme on physical performance in prepubescent soccer players. Journal of Sports Medicine & Physical Fitness 2001;41:342-8. Not a randomized controlled trial (RCT)
Ref ID: 4742

(1235) Dias RG, Alves MJNN, Pereira AC, Rondon MUPB, dos Santos MR, Krieger JE, Krieger MH, Negrao CE. Glu298Asp eNOS gene polymorphism causes attenuation in nonexercising muscle vasodilatation. Physiological Genomics 2009;37(2):99-107. Study limited to adults
Ref ID: 5237

(1236) Dias RMR, Carvalho FO, Souza CFd, Avelar A, Altimari LR, Cyrino ES. Características antropométricas e de desempenho motor de atletas de futsal em diferentes categorias^ipt. Rev bras cineantropom desempenho hum 2007 September;9(3). Cross-sectional study
Ref ID: 4092

(1237) Diaz-Gomez NM, Domenech E, Cortabarria C, Barroso F, Castells S, Jimenez A. The Effect of Zinc Supplementation on Linear Growth, Body Composition, and Growth Factors in Preterm Infants. Pediatrics 2003 May;111(5):1002. Diet Intervention or Supplement Study
Ref ID: 3564

(1238) Diaz A, Vogiatzi MG, Sanz MM, German J. Evaluation of short stature, carbohydrate metabolism and other endocrinopathies in Bloom's syndrome. Hormone Research 2006;66:111-7. Not an exercise intervention study
Ref ID: 4743

(1239) Diaz RG, Esparza-Romero J, Moya-Camarena SY, Robles-Sardin AE, Valencia ME. Lifestyle intervention in primary care settings improves obesity parameters among Mexican youth. Journal of the American Dietetic Association 2010 February;110(2):285-90. Lifestyle Intervention
Ref ID: 602

(1240) Dib SA. Resistência à insulina e síndrome metabólica no diabetes melito do tipo 1^ipt. Arq bras endocrinol metab 2006 April;50(2):250-63. Review article
Ref ID: 659

(1241) Dicken-Kano R, Bell MM. Pedometers as a means to increase walking and achieve weight loss. J Am Board Fam Med 2006 September;19(5):524-5. Not an exercise intervention study
Ref ID: 270

(1242) Dickinson S, Hancock DP, Petocz P, Ceriello A, Brand-Miller J. High-glycemic index carbohydrate increases nuclear factor-kappaB activation in mononuclear cells of young, lean healthy subjects. Am J Clin Nutr 2008 May;87(5):1188-93. Not an exercise intervention study
Ref ID: 184

(1243) Dickson-Parnell BE, Zeichner A. Effects of a short-term exercise program on caloric consumption. Health Psychology 1985;4(5):437-48. Study limited to adults
Ref ID: 2355

(1244) Dickson JM, Weavers HM, Mitchell N, Winter EM, Wilkinson ID, Van Beek EJ, Wild JM, Griffiths PD. The effects of dehydration on brain volume -- preliminary results. International Journal of Sports Medicine 2005 July;26(6):481-5. Not an exercise intervention study
Ref ID: 1491

(1245) Dieruf K, Burtner PA, Provost B, Phillips J, Bernitsky-Beddingfield A, Sullivan KJ. A Pilot Study of Quality of Life in Children with Cerebral Palsy After Intensive Body Weight-Supported Treadmill Training. Pediatric Physical Therapy 2009;21(1):45-52. Study less than 4 weeks
Ref ID: 5238

(1246) Dikel W, Olness K. Self-hypnosis, biofeedback, and voluntary peripheral temperature control in children. Pediatrics 1980 September;66(3):335-40. Not an exercise intervention study
Ref ID: 2378

(1247) Dimatos SC, Souza JAd, Dimatos OC, Araújo EJ, Dimatos DC, Pereima MJL. Tricobezoar na infância: relato de três casos e revisão da literatura^ipt. ACM arq catarin med 2009 July;38(2):112-5. Case-Control / Case Study
Ref ID: 4093

(1248) Dimkpa U, Oji JO. Association of heart rate recovery after exercise with indices of obesity in healthy, non-obese adults. Eur J Appl Physiol 2010 March;108(4):695-9. Study limited to adults, Not an exercise intervention study
Ref ID: 84

(1249) Dimkpa U, Oji JO. Relationship of body mass index with haemodynamic variables and abnormalities in young adults. Journal of Human Hypertension 2010;24(4):230-6. Cross-sectional study
Ref ID: 5239

(1250) DiNapoli PP, Lewis JB. Understanding school-age obesity: through participatory action research. MCN, American Journal of Maternal Child Nursing 2008 March;33(2):104-10. Cross-sectional study
Ref ID: 985

(1251) Dinis-Oliveira RJ, Duarte JA, SÃ¡nchez-Navarro A, RemiÃ£o F, Bastos ML, Carvalho F. Paraquat Poisonings: Mechanisms of Lung Toxicity, Clinical Features, and Treatment. Critical Reviews in Toxicology 2008 January;38(1):13-71. Review article
Ref ID: 3565

(1252) Diniz IMS, Lopes AdS, Dummel CCB, Rieger T. Crescimento físico e adiposidade corporal de escolares^ipt. Rev bras cineantropom desempenho hum 2006 June;8(2). Cross-sectional study
Ref ID: 4094

(1253) Diniz IMS, Lopes AdS, Borgatto AF. Crescimento físico e composição corporal de escolares de diferentes grupos étnicos do Estado do Rio Grande do Sul, Brasil^ipt. Rev bras cineantropom desempenho hum 2008 March;10(1). Cross-sectional study
Ref ID: 4095

(1254) Diniz MB, Coldebella CR, Zuanon AC, Cordeiro RdC. Alterações orais em crianças prematuras e de baixo peso ao nascer: a importância da relação entre pediatras e odontopediatras^ipt
Oral abnormalities in preterm and low birth weight infants: the importance of the relationship between pediatricians and pediatric dentists^ien. Rev paul pediatr 2011 September;29(3):440-53. Review article
Ref ID: 4096

(1255) Ditunno JF, Jr., Barbeau H, Dobkin BH, Elashoff R, Harkema S, Marino RJ, Hauck WW, Apple D, Basso DM, Behrman A, Deforge D, Fugate L, Saulino M, Scott M, Chung J, Spinal Cord Injury Locomotor Trial Group. Validity of the walking scale for spinal cord injury and other domains of function in a multicenter clinical trial. Neurorehabilitation & Neural Repair 2007 November;21(6):539-50. Not All Participants were Overweight and/or Obese
Ref ID: 1151

(1256) Dixon JB, Jones K, Dixon M. Medical versus surgical interventions for the metabolic complications of obesity in children. Semin Pediatr Surg 2009 August;18(3):168-75. Review article
Ref ID: 726

(1257) Díaz franco MB, Duran reina MC, Ramírez Pérez MF. Perfil funcional después de un entrenamiento con pesas en jugadores de futbol entre 15 y 19 años de edad de las divisiones menores del club Santa Fe C. D.^ies. Rev colomb rehabil 2004 October;1(3):49-56. No control group (NC)
Ref ID: 4097

(1258) Díaz RG, Esparza RJ, Moya-Camarena SY, Robles-Sardín AE, Valencia ME. Lifestyle intervention in primary care settings improves obesity parameters among Mexican youth. Journal of the American Dietetic Association 2010;110:285-90. Lifestyle Intervention
Ref ID: 4744

(1259) Djuric Z, DiLaura NM, Jenkins I, Darga L, Jen CK, Mood D, Bradley E, Hryniuk WM. Combining weight-loss counseling with the weight watchers plan for obese breast cancer survivors. Obes Res 2002 July;10(7):657-65. Study limited to adults
Ref ID: 376

(1260) Dobbins M, De CK, Robeson P, Husson H, Tirilis D. School-based physical activity programs for promoting physical activity and fitness in children and adolescents aged 6-18. [Review] [408 refs]. Cochrane Database of Systematic Reviews (1):CD007651, 2009 2009;(1):CD007651. Review article
Ref ID: 807

(1261) Doberenz J, Birkenfeld C, Kluge H, Eder K. Effects ofL-carnitine supplementation in pregnant sows on plasma concentrations of insulin-like growth factors, various hormones and metabolites and chorion characteristics. Journal of Animal Physiology & Animal Nutrition 2006 December;90(11/12):487-99. Animal study
Ref ID: 3566

(1262) Dobkin B, Apple D, Barbeau H, Basso M, Behrman A, Deforge D, Ditunno J, Dudley G, Elashoff R, Fugate L, Harkema S, Saulino M, Scott M, Spinal Cord Injury Locomotor Trial Group. Weight-supported treadmill vs over-ground training for walking after acute incomplete SCI. Neurology 2006 February 28;66(4):484-93. Not All Participants were Overweight and/or Obese
Ref ID: 1413

(1263) Dodd CJ, Welsman JR, Armstrong N. Energy intake and appetite following exercise in lean and overweight girls. Appetite 2008 November;51(3):482-8. Acute study
Ref ID: 183

(1264) Dodd JM, Grivell RM, Crowther CA, Robinson JS. Antenatal interventions for overweight or obese pregnant women: a systematic review of randomised trials. [Review]. BJOG: An International Journal of Obstetrics & Gynaecology 117(11):1316-26, 2010 Oct 2010;(11):1316-26. Review article
Ref ID: 2943

(1265) Dodd KJ, Foley S. Partial body-weight-supported treadmill training can improve walking in children with cerebral palsy: a clinical controlled trial. Developmental Medicine & Child Neurology 2007 February;49(2):101-5. Not a randomized controlled trial (RCT)
Ref ID: 1282

(1266) Dodd SL, Brooks E, Powers SK, Tulley R. The effects of caffeine on graded exercise performance in caffeine naive versus habituated subjects. European Journal of Applied Physiology & Occupational Physiology 1991;62(6):424-9. Diet Intervention or Supplement Study
Ref ID: 2299

(1267) Doherty M, Dimitriou L. Comparison of lung volume in Greek swimmers, land based athletes, and sedentary controls using allometric scaling. British Journal of Sports Medicine 1997 December;31(4):337-41. Cross-sectional study
Ref ID: 2115

(1268) Dolev E, Burstein R, Lubin F, Wishnizer R, Chetrit A, Shefi M, Deuster PA. Interpretation of zinc status indicators in a strenuously exercising population. Journal of the American Dietetic Association 1995 April;95(4):482-4. Review article
Ref ID: 2215

(1269) Dolinsky DH, Brouwer RJ, Evenson KR, Siega-Riz AM, Ostbye T. Correlates of sedentary time and physical activity among preschool-aged children. Preventing Chronic Disease 2011 November;8(6):A131. Cross-sectional study
Ref ID: 2497

(1270) Dollfus C, Blanche S, Trocme N, Funck-Brentano I, Bonnet F, Levan P. Correction of facial lipoatrophy using autologous fat transplants in HIV-infected adolescents. HIV Medicine 2009;10(5):263-8. Not an exercise intervention study
Ref ID: 5240

(1271) Domingues RB, Teixeira AL, Domingues SA. Physical practice is associated with less functional disability in medical students with migraine^ien
A prática de exercícios físicos está associada a menor comprometimento funcional da migrânea entre estudantes de medicina^ipt. Arq neuropsiquiatr 2011 February;69(1):39-43. Survey or questionnaire
Ref ID: 4098

(1272) Dominici N, Ivanenko YP, Lacquaniti F. Control of foot trajectory in walking toddlers: Adaptation to load changes. Journal of Neurophysiology 2007;97(4):2790-801. Not an exercise intervention study
Ref ID: 5241

(1273) Donaghue KC, Pena MM, Chan AK, Blades BL, King J, Storlien LH, Silink M. Beneficial effects of increasing monounsaturated fat intake in adolescents with type 1 diabetes. Diabetes Research & Clinical Practice 2000 June;48(3):193-9. Diet Intervention Study
Ref ID: 1979

(1274) Donahue RP, Prineas RJ, Gomez O, Hong CP. Familial Resemblance of Body-Fat Distribution - the Minneapolis-Childrens-Blood-Pressure-Study. International Journal of Obesity 1992;16(3):161-7. Cross-sectional study
Ref ID: 5242

(1275) Dong W, Colhoun HM, Poulter NR. Blood pressure in women using oral contraceptives: results from the Health Survey for England 1994. Journal of Hypertension 1997 October;15(10):1063-8. Cross-sectional study
Ref ID: 2121

(1276) Dong Y, Pollock N, Stallmann-Jorgensen IS, Gutin B, Lan L, Chen TC, Keeton D, Petty K, Holick MF, Zhu H. Low 25-hydroxyvitamin D levels in adolescents: race, season, adiposity, physical activity, and fitness. Pediatrics 2010 June;125(6):1104-11. Cross-sectional study
Ref ID: 519

(1277) Donma MM, Donma O. The influence of feeding patterns on head circumference among Turkish infants during the first 6 months of life. Brain & Development 1997;19(6):393-7. Subjects less than 2 years old
Ref ID: 5243

(1278) Donma MM, Donma O. Infant feeding and growth: A study on Turkish infants from birth to 6 months. Pediatrics International 1999;41(5):542-8. Subjects less than 2 years old
Ref ID: 5244

(1279) Donnelly JE, Kirk EP, Jacobsen DJ, Hill JO, Sullivan DK, Johnson SL. Effects of 16 mo of verified, supervised aerobic exercise on macronutrient intake in overweight men and women: the Midwest Exercise Trial. Am J Clin Nutr 2003 November;78(5):950-6. Study limited to adults
Ref ID: 352

(1280) Donnelly JE, Hill JO, Jacobsen DJ, Potteiger J, Sullivan DK, Johnson SL, Heelan K, Hise M, Fennessey PV, Sonko B, Sharp T, Jakicic JM, Blair SN, Tran ZV, Mayo M, Gibson C, Washburn RA. Effects of a 16-month randomized controlled exercise trial on body weight and composition in young, overweight men and women: the Midwest Exercise Trial. Arch Intern Med 2003 June 9;163(11):1343-50. Study limited to adults
Ref ID: 362

(1281) Donnelly JE, Sullivan DK, Smith BK, Jacobsen DJ, Washburn RA, Johnson SL, Hill JO, Mayo MS, Spaeth KR, Gibson C. Alteration of dietary fat intake to prevent weight gain: Jayhawk Observed Eating Trial. Obesity (Silver Spring) 2008 January;16(1):107-12. Not an exercise intervention study
Ref ID: 202

(1282) Donnelly JE, Greene JL, Gibson CA, Smith BK, Washburn RA, Sullivan DK, DuBose K, Mayo MS, Schmelzle KH, Ryan JJ, Jacobsen DJ, Williams SL. Physical Activity Across the Curriculum (PAAC): a randomized controlled trial to promote physical activity and diminish overweight and obesity in elementary school children. Prev Med 2009 October;49(4):336-41. Not All Participants were Overweight and/or Obese
Ref ID: 101

(1283) Donnelly JE, Lambourne K. Classroom-based physical activity, cognition, and academic achievement. [Review]. Preventive Medicine 2011 June;52:Suppl-42. Review article
Ref ID: 1044

(1284) Dore E, Martin R, Ratel S, Duche P, Bedu M, Van PE. Gender differences in peak muscle performance during growth. International Journal of Sports Medicine 2005 May;26(4):274-80. Cross-sectional study
Ref ID: 1532

(1285) Dorofeyeva EE, Dorofeyev AE. Biochemical and heart adaptations to physical training and supplementation with amino acids. Journal of Strength & Conditioning Research 2004 November;18(4):738-40. Diet Intervention or Supplement Study
Ref ID: 1568

(1286) Dorosko SM. Vitamin A, Mastitis, and Mother-to-Child Transmission of HIV-1 through Breast-feeding: Current Information and Gaps in Knowledge. Nutrition Reviews 2005 October;63(10):332-46. Review article
Ref ID: 3567

(1287) Dotan R, Ohana S, Bediz C, Falk B. Blood lactate disappearance dynamics in boys and men following exercise of similar and dissimilar peak-lactate concentrations. Journal of Pediatric Endocrinology 2003 March;16(3):419-29. Acute study
Ref ID: 1757

(1288) Dougherty KA, Baker LB, Chow M, Kenney WL. Two percent dehydration impairs and six percent carbohydrate drink improves boys basketball skills. Medicine and science in sports and exercise 2006;38:1650-8. Acute study
Ref ID: 4745

(1289) Dougherty KA, Chow M, Kenney WL. Critical environmental limits for exercising heat-acclimated lean and obese boys. Eur J Appl Physiol 2010 March;108(4):779-89. Not an exercise intervention study
Ref ID: 56

(1290) Douma-van Riet DC, Engelbert RH, van Genderen FR, Ter Horst-De Ronde MT, de Goede-Bolder A, Hartman A. Physical fitness in children with haemophilia and the effect of overweight. Haemophilia 2009 March;15(2):519-27. Cross-sectional study
Ref ID: 776

(1291) Dow CB. Young Children and Movement: The Power of Creative Dance. Young Children 2010 March 1;65(2):30-5. Review article
Ref ID: 3900

(1292) Dowda M, McKenzie TL, Cohen DA, Scott MM, Evenson KR, Bedimo-Rung AL, Voorhees CC, Almeida MJ. Commercial venues as supports for physical activity in adolescent girls. Preventive Medicine 2007;45:163-8. Cross-sectional study
Ref ID: 4746

(1293) Downs DS, Feinberg M, Hillemeier MM, Weisman CS, Chase GA, Chuang CH, Parrott R, Francis LA. Design of the Central Pennsylvania Women's Health Study (CePAWHS) strong healthy women intervention: improving preconceptional health. Matern Child Health J 2009 January;13(1):18-28. Study limited to adults, Description versus conduct of study
Ref ID: 198

(1294) Downs SM, Farmer A, Quintanilha M, Berry TR, Mager DR, Willows ND, McCargar LJ. From Paper to Practice: Barriers to Adopting Nutrition Guidelines in Schools. Journal of nutrition education and behavior 2012;44(2):114-22. Cross-sectional study
Ref ID: 5245

(1295) Dowson JH. Pharmacological treatment for attention-deficit/hyperactivity disorder (ADHD) in adults. Curr Psychiatry Rev 2006;2(3):317-31. Review article
Ref ID: 3222

(1296) Doyle-Baker PK, Venner AA, Lyon ME, Fung T. Impact of a combined diet and progressive exercise intervention for overweight and obese children: the B.E. H.I.P. study. Applied Physiology, Nutrition, & Metabolism = Physiologie Appliquee, Nutrition et Metabolisme 2011 August;36(4):515-25. Multiple interventions
Ref ID: 2499

(1297) Doyle-Lucas AF, Akers JD, Davy BM. Energetic efficiency, menstrual irregularity, and bone mineral density in elite professional female ballet dancers. Journal of Dance Medicine & Science 2010;14(4):146-54. Not a randomized controlled trial (RCT)
Ref ID: 2500

(1298) Doyle AC, Goldschmidt A, Huang C, Winzelberg AJ, Taylor CB, Wilfley DE. Reduction of overweight and eating disorder symptoms via the Internet in adolescents: a randomized controlled trial. J Adolesc Health 2008 August;43(2):172-9. No exercise only group
Ref ID: 168

(1299) Dórea V, Ronque ERV, Cyrino ES, Serassuelo Junior H, Gobbo LA, Carvalho FO, Souza CFd, Melo JCd, Gaion PA. Aptidão física relacionada à saúde em escolares de Jequié, BA, Brasil^ipt. Rev bras med esporte 2008 December;14(6):494-9. Cross-sectional study
Ref ID: 4099

(1300) Dreifuss FE, Rosman NP, Cloyd JC, Pellock JM, Kuzniecky RI, Lo WD, Matsuo F, Sharp GB, Conry JA, Bergen DC, Bell WE. A comparison of rectal diazepam gel and placebo for acute repetitive seizures. The New England journal of medicine 1998;338:1869-75. Drug intervention study
Ref ID: 912

(1301) Dresser R. Wanted. Hastings Center Report 1992 January;22(1):24. Editorial or letter or comment
Ref ID: 579

(1302) Dreyhaupt J, Koch B, Wirt T, Schreiber A, Brandstetter S, Kesztyus D, Wartha O, Kobel S, Kettner S, Prokopchuk D, Hundsdorfer V, Klepsch M, Wiedom M, Sufeida S, Fischbach N, Muche R, Seufert T, Steinacker JM. Evaluation of a health promotion program in children: Study protocol and design of the cluster-randomized Baden-Wurttemberg primary school study [DRKS-ID: DRKS00000494]. BMC Public Health 2012;12:157. Description of study from review or magazine or etc. (not the actual study)
Ref ID: 2501

(1303) Drieling RL, Ma J, Stafford RS. Evaluating clinic and community-based lifestyle interventions for obesity reduction in a low-income Latino neighborhood: Vivamos Activos Fair Oaks Program. BMC Public Health 2011;11:98. Multiple interventions
Ref ID: 2502

(1304) du AS, Lartey A, Brown KH, Zlotkin S, Briend A, Dewey KG, Osborn DA, Evans N, Kluckow M, Bowen JR, Rieger I. Randomized comparison of 3 types of micronutrient supplements for home fortification of complementary foods in Ghana: effects on growth and motor development Low superior vena cava flow and effect of inotropes on neurodevelopment to 3 years in preterm infants. Am J Clin Nutr 2007;86:412-20. Diet Intervention or Supplement Study
Ref ID: 4747

(1305) Du H, Xu X, Yao T, Wu X. [Application of combined epidural-spinal anesthesia in pediatric surgery and postoperative analgesia]. Beijing da xue xue bao Yi xue ban = Journal of Peking University Health sciences 2003;35:642-4. Drug intervention study
Ref ID: 4748

(1306) Du X, Zhu K, Trube A, Zhang Q, Ma G, Hu X, Fraser DR, Greenfield H. School-milk intervention trial enhances growth and bone mineral accretion in Chinese girls aged 10-12 years in Beijing. The British journal of nutrition 2004;92:159-68. Diet Intervention or Supplement Study
Ref ID: 4749

(1307) Du X, Zhu K, Trube A, Zhang Q, Ma G, Hu X, Fraser DR, Greenfield H. School-milk intervention trial enhances growth and bone mineral accretion in Chinese girls aged 10-12 years in Beijing.[Erratum appears in Br J Nutr. 2005 Apr;93(4):571-2]. BR J NUTR 2004 July;92(1):159-68. Diet Intervention or Supplement Study
Ref ID: 1613

(1308) Du XQ, Greenfield H, Fraser DR, Ge KY, Liu ZH, He W. Milk consumption and bone mineral content in Chinese adolescent girls. Bone 2002;30:521-8. Cross-sectional study
Ref ID: 4750

(1309) Dua JS, Cooper AR, Fox KR, Graham SA. Exercise training in adults with congenital heart disease: feasibility and benefits. International Journal of Cardiology 2010 January 21;138(2):196-205. Study limited to adults
Ref ID: 594

(1310) Duarte CM, Nascimento VBd, Akerman M. Gravidez na adolescência e exclusão social: análise de disparidades intra-urbanas. Rev panam salud pública 2006 April;19(4):236-43. Cross-sectional study
Ref ID: 4100

(1311) DuBose KD, Mayo MS, Gibson CA, Green JL, Hill JO, Jacobsen DJ, Smith BK, Sullivan DK, Washburn RA, Donnelly JE. Physical activity across the curriculum (PAAC): rationale and design. Contemp Clin Trials 2008 January;29(1):83-93. Description versus conduct of study
Ref ID: 227

(1312) Duckworth LC, Gately PJ, Radley D, Cooke CB, King RF, Hill AJ. RCT of a high-protein diet on hunger motivation and weight-loss in obese children: an extension and replication. Obesity (Silver Spring) 2009 September;17(9):1808-10. No exercise only group, No comparative control group
Ref ID: 129

(1313) Ducro-Steverink D. Selection against boar taint: a simulation study. Acta Veterinaria Scandinavica 2006 January 2;48:6-3. Animal study
Ref ID: 3568

(1314) Due A, Larsen TM, Mu H, Hermansen K, Stender S, Astrup A. Comparison of 3 ad libitum diets for weight-loss maintenance, risk of cardiovascular disease, and diabetes: a 6-mo randomized, controlled trial. Am J Clin Nutr 2008 November;88(5):1232-41. Study limited to adults, No exercise only group
Ref ID: 152

(1315) Duff SV, Gordon AM. Learning of grasp control in children with hemiplegic cerebral palsy. Dev Med Child Neurol 2003;45:746-57. Not a randomized controlled trial (RCT)
Ref ID: 4751

(1316) Duffy FH, McAnulty GB, McCreary MC, Cuchural GJ, Komaroff AL. EEG spectral coherence data distinguish chronic fatigue syndrome patients from healthy controls and depressed patients-A case control study. 11[1], 82-94. 2011. BioMed Central. Case-Control / Case Study,
Ref ID: 3569

(1317) Duffy G, Spence SH. The effectiveness of cognitive self-management as an adjunct to a behavioural intervention for childhood obesity: a research note. Journal of child psychology and psychiatry, and allied disciplines 1993;34:1043-50. Behavior Modification Intervention
Ref ID: 4752

(1318) Dugas LR, Ebersole K, Schoeller D, Yanovski JA, Barquera S, Rivera J, Durazo-Arzivu R, Luke A. Very low levels of energy expenditure among pre-adolescent Mexican-American girls. International Journal of Pediatric Obesity 2008;3(2):123-6. Cross-sectional study
Ref ID: 957

(1319) Duggins M, Cherven P, Carrithers J, Messamore J, Harvey A. Impact of family YMCA membership on childhood obesity: a randomized controlled effectiveness trial. J Am Board Fam Med 2010 May;23(3):323-33. No exercise only group, No comparative control group
Ref ID: 44

(1320) Duijkers IJM, Klipping C, Grob P, Korver T. Effects of a monophasic combined oral contraceptive containing nomegestrol acetate and 17Î²-oestradiol on ovarian function in comparison to a monophasic combined oral contraceptive containing drospirenone and ethinylestradiol. European Journal of Contraception & Reproductive Health Care 2010 October;15(5):314-25. Drug intervention study
Ref ID: 3570

(1321) Dujovne CA. Treatment of familial hypercholesterolemia and other genetic dyslipidemias. Curr Treat Options Cardiovasc Med 2004;6(4):269-78. Review article
Ref ID: 3223

(1322) Dumith SC. Physical activity in Brazil: a systematic review^ien
Atividade física no Brasil: uma revisão sistemática^ipt. Cad saúde pública 2009;25(supl.3):S415-S426. Review article
Ref ID: 4101

(1323) Dumith SC, Farias Júnior JC. Sobrepeso e obesidade em crianças e adolescentes: comparação de três critérios de classificação baseados no índice de massa corporal^ipt
Overweight and obesity in children and adolescents: comparison of three classification criteria based on body mass index^ien. Rev panam salud pública 2010 July;28(1):30-5. Cohort Study
Ref ID: 4103

(1324) Dumith SC, Hallal PC, Menezes AMB, Araújo CL. Sedentary behavior in adolescents: the 11-year follow-up of the 1993 Pelotas (Brazil) birth cohort study^ien
Comportamento sedentário em adolescentes: a visita de 11 anos da coorte de nascimentos de Pelotas, Rio Grande do Sul, Brasil, 1993^ipt. Cad saúde pública 2010 October;26(10):1928-36. Survey or questionnaire
Ref ID: 4102

(1325) Dumith SC, Domingues MR, Mendoza-Sassi RA, Cesar JA. Atividade física durante a gestação e associação com indicadores de saúde materno-infantil^ipt
Physical activity during pregnancy and its association with maternal and child health indicators^ien
Actividad física durante la gestación y asociación con indicadores de salud materno-infantil^ies. Rev saúde pública 2012 April;46(2):327-33. Cross-sectional study
Ref ID: 4104

(1326) Dumith SdC, Azevedo Júnior MR, Rombaldi AJ. Aptidão física relacionada à saúde de alunos do ensino fundamental do município de Rio Grande, RS, Brasil^ipt. Rev bras med esporte 2008 October;14(5):454-9. Cross-sectional study
Ref ID: 4105

(1327) Dumoulin C, Bourbonnais D, Morin M, Gravel D, Lemieux MC. Predictors of success for physiotherapy treatment in women with persistent postpartum stress urinary incontinence. Archives of physical medicine and rehabilitation 2010;91:1059-63. Study limited to adults
Ref ID: 4754

(1328) Duncan BB, Schmidt MI, Polanczyk CA, Homrich CS, Rosa RdS, Achutti AC. Fatores de risco para doenças näo-transmissíveis em área metropolitana na regiäo sul do Brasil: prevalência e simultaneidade. Rev saúde pública 1993 February;27(1):43-8. Survey or questionnaire
Ref ID: 814

(1329) Duncan MJ, Al-Nakeeb Y, Nevill AM. Effects of a 6-week circuit training intervention on body esteem and body mass index in British primary school children. Body Image 2009 June;6(3):216-20. Not All Participants were Overweight and/or Obese
Ref ID: 117

(1330) Dunn AL, Andersen RE, Jakicic JM. Lifestyle physical activity interventions. History, short- and long-term effects, and recommendations. [Review] [77 refs]. American Journal of Preventive Medicine 1998 November;15(4):398-412. Review article
Ref ID: 2073

(1331) Dunshea FR, Cox ML. Effect of dietary protein on body composition and insulin resistance using a pig model of the child and adolescent. Nutrition & Dietetics 2008;65:S60-S65. Animal study
Ref ID: 5246

(1332) Dunton GF, Liao Y, Intille SS, Spruijt-Metz D, Pentz M. Investigating children's physical activity and sedentary behavior using ecological momentary assessment with mobile phones. Obesity 2011 June;19(6):1205-12. Not All Participants were Overweight and/or Obese
Ref ID: 2503

(1333) Dupuis JM, Vivant JF, Daudet G, Bouvet A, Clement M, Dazord A, Dumet N, David M, Bellon G. [Personal sports training in the management of obese boys aged 12 to 16 years]. [French]. Archives de Pediatrie 2000 November;7(11):1185-93. Not a randomized controlled trial (RCT)
Ref ID: 1945

(1334) Duquia RP, Dumith SdC, Reichert FF, Madruga SW, Duro LN, Menezes AMB, Araújo CL. Epidemiologia das pregas cutâneas triciptal e subescapular elevadas em adolescentes^ipt. Cad saude publica 2008 January;24(1):113-21. Cross-sectional study, Cohort Study
Ref ID: 4106

(1335) Durant N, Harris SK, Doyle S, Person S, Saelens BE, Kerr J, Norman GJ, Sallis JF. Relation of school environment and policy to adolescent physical activity. Journal of School Health 205 June;79(4):153-9. Survey or questionnaire
Ref ID: 786

(1336) Durant RH, Baranowski T, Johnson M, Thompson WO. The relationship among television watching, physical activity, and body composition of young children. Pediatrics 1994 October;94(4:Pt 1):t-55. Observational study
Ref ID: 2235

(1337) Durnin JV, Aitchison TC, Beckett C, Husaini M, Pollitt E. Nutritional intake of an undernourished infant population receiving an energy and micronutrient supplement in Indonesia. European Journal of Clinical Nutrition 2000;54 Suppl 2:S43-S51. Subjects less than 2 years old
Ref ID: 848

(1338) Duruz J. Food As Nostalgia: Eating the Fifties and Sixties. Australian Historical Studies 1999 October;30(113):231. Editorial or letter or comment
Ref ID: 3571

(1339) Dutton GR, Davis MP, Welsch MA, Brantley PJ. Promoting physical activity for low-income minority women in primary care. Am J Health Behav 2007 November;31(6):622-31. Study limited to adults
Ref ID: 223

(1340) Duvall J. A comparison of engagement strategies for encouraging outdoor walking. Journal of Physical Activity & Health 2012 January;9(1):62-70. Study limited to adults
Ref ID: 2504

(1341) Dvorchik B. Moderate liver impairment has no influence on daptomycin pharmacokinetics. Journal of Clinical Pharmacology 2004 July;44(7):715-22. Study limited to adults
Ref ID: 1617

(1342) Dwyer JT, Feldman HA, Yang M, Webber LS, Must A, Perry CL, Nader PR, Parcel GS. Maintenance of lightweight correlates with decreased cardiovascular risk factors in early adolescence. J Adolesc Health 2002 August;31(2):117-24. Not an exercise intervention study, Cohort Study
Ref ID: 375

(1343) Dwyer T, Coonan WE, Leitch DR, Hetzel BS, Baghurst RA. An investigation of the effects of daily physical activity on the health of primary school students in South Australia. International Journal of Epidemiology 1983 September;12(3):308-13. Not a randomized controlled trial (RCT)
Ref ID: 2366

(1344) Dybdahl R. Children and mothers in war: an outcome study of a psychosocial intervention program. Child development 2001;72:1214-30. Not an exercise intervention study
Ref ID: 4755

(1345) Dyer AR, Cutter GR, Liu KQ, Armstrong MA, Friedman GD, Hughes GH, Dolce JJ, Raczynski J, Burke G, Manolio T. Alcohol intake and blood pressure in young adults: the CARDIA Study. Journal of Clinical Epidemiology 1990;43(1):1-13. Study limited to adults
Ref ID: 2313

(1346) Dyke JV, Kirk AB, Martinelango K, Dasgupta PK. Sample processing method for the determination of perchlorate in milk. Analytica Chimica Acta 2006;567(1):73-8. Diet Intervention or Supplement Study
Ref ID: 5247

(1347) Dzewaltowski DA, Rosenkranz RR, Geller KS, Coleman KJ, Welk GJ, Hastmann TJ, Milliken GA. HOP'N after-school project: an obesity prevention randomized controlled trial. International Journal of Behavioral Nutrition and Physical Activity 2010;7. Diet & Exercise intervention
Ref ID: 5248

(1348) Eakin EG, Youlden DR, Baade PD, Lawler SP, Reeves MM, Heyworth JS, Fritschi L. Health behaviors of cancer survivors: data from an Australian population-based survey. Cancer Causes Control 2007 October;18(8):881-94. Not an exercise intervention study, Observational study
Ref ID: 226

(1349) Eaton DK, Kann L, Kinchen S, Ross J, Hawkins J, Harris WA, Lowry R, McManus T, Chyen D, Shanklin S, Lim C, Grunbaum JA, Wechsler H. Youth risk behavior surveillance - United States, 2005. Journal of School Health 2006;76(7):353-72. Review article
Ref ID: 5249

(1350) Eaton DK, Foti K, Brener ND, Crosby AE, Flores G, Kann L. Associations Between Risk Behaviors and Suicidal Ideation and Suicide Attempts: Do Racial/Ethnic Variations in Associations Account for Increased Risk of Suicidal Behaviors Among Hispanic/Latina 9th- to 12th-Grade Female Students? Archives of Suicide Research 2011;15(2):113-26. Survey or questionnaire
Ref ID: 5250

(1351) Eaton DK, Kann L, Kinchen S, Shanklin S, Flint KH, Hawkins J, Harris WA, Lowry R, McManus T, Chyen D, Whittle L, Lim C, Wechsler H, Centers for Disease Control and Prevention (DHHS/PHS). Youth Risk Behavior Surveillance--United States, 2011. Morbidity and Mortality Weekly Report. Surveillance Summaries. Volume 61, Number 4. Centers for Disease Control and Prevention; 2012 Jun 8. ¬

Review article
Ref ID: 3901

(1352) Ebbeling CB, Rodriguez NR. Effects of exercise combined with diet therapy on protein utilization in obese children. Medicine & Science in Sports & Exercise 1999 March;31(3):378-85. Diet & Exercise intervention
Ref ID: 2039

(1353) Ebbeling CB, Feldman HA, Osganian SK, Chomitz VR, Ellenbogen SJ, Ludwig DS. Effects of decreasing sugar-sweetened beverage consumption on body weight in adolescents: a randomized, controlled pilot study. Pediatrics 2006 March;117(3):673-80. Not an exercise intervention study
Ref ID: 285

(1354) Ebbeling CB, Ludwig DS. Pediatric obesity prevention initiatives: more questions than answers. Archives of Pediatrics & Adolescent Medicine 2010 November;164(11):1067-9. Editorial or letter or comment
Ref ID: 2505

(1355) Ebbeling CB, Swain JF, Feldman HA, Wong WW, Hachey DL, Garcia LE, Ludwig DS. Effects of dietary composition on energy expenditure during weight-loss maintenance. JAMA : the journal of the American Medical Association 2012;307:2627-34. Diet Intervention Study
Ref ID: 4756

(1356) Ebbeling CB, Pawlak DB, Ludwig DS. Childhood obesity: public-health crisis, common sense cure. Lancet 2002 August 10;360(9331):473. Review article
Ref ID: 3572

(1357) Ebben WP, Wurm B, VanderZanden TL, Spadavecchia ML, Durocher JJ, Bickham CT, Petushek EJ. Kinetic analysis of several variations of push-ups. Journal of Strength & Conditioning Research 2011 October;25(10):2891-4. Not a randomized controlled trial (RCT)
Ref ID: 2506

(1358) Ebrahim S, Smith GD. Systematic review of randomised controlled trials of multiple risk factor interventions for preventing coronary heart disease. BMJ 1997 June 7;314(7095):1666-74. Review article
Ref ID: 2136

(1359) Ebrahimi S, Pormahmodi A, Kamkar A. Study of zinc supplementation on growth of schoolchildren in Yasuj, Southwest of Iran. Pakistan Journal of Nutrition 2006;5:341-2. Diet Intervention or Supplement Study
Ref ID: 4757

(1360) Edgar TS. Clinical Utility Botulinum Toxin in the Treatment of Cerebral Palsy: Comprehensive Review. Journal of Child Neurology 2001 January;16(1):37. Review article
Ref ID: 3573

(1361) Edouard T, Deal C, Van VG, Gaulin N, Moreau A, Rauch F, Alos N. Muscle-bone characteristics in children with Prader-Willi syndrome. Journal of Clinical Endocrinology & Metabolism 2012 February;97(2):E275-E281. Not an exercise intervention study
Ref ID: 2507

(1362) Eek MN, Tranberg R, Zugner R, Alkema K, Beckung E. Muscle strength training to improve gait function in children with cerebral palsy. Developmental Medicine & Child Neurology 2008 October;50(10):759-64. Not a randomized controlled trial (RCT)
Ref ID: 884

(1363) Egede LE. Lifestyle modification to improve blood pressure control in individuals with diabetes: is physician advice effective? Diabetes Care 2003 March;26(3):602-7. Study limited to adults
Ref ID: 1772

(1364) Eguchi R, Cheik NC, Oyama LM, do Nascimento CMO, de Mello MT, Tufik S, Damaso A. Effects of the Chronic Exercise on the Circulating Concentration of Leptin and Ghrelin in Rats With Diet-induced Obesity. Revista Brasileira de Medicina do Esporte 2008;14(3):182-7. Animal study
Ref ID: 5251

(1365) Eiben G, Lissner L. Health Hunters--an intervention to prevent overweight and obesity in young high-risk women. Int J Obes (Lond) 2006 April;30(4):691-6. Study limited to adults
Ref ID: 294

(1366) Eiholzer U, Gisin R, Weinmann C, Kriemler S, Steinert H, Torresani T, Zachmann M, Prader A. Treatment with human growth hormone in patients with Prader-Labhart-Willi syndrome reduces body fat and increases muscle mass and physical performance. European Journal of Pediatrics 1998 May;157(5):368-77. Drug intervention study
Ref ID: 2089

(1367) Eiholzer U, l'Allemand D, Schlumpf M, Rousson V, Gasser T, Fusch C. Growth hormone and body composition in children younger than 2 years with Prader-Willi syndrome. The Journal of pediatrics 2004;144:753-8. Subjects less than 2 years old
Ref ID: 4758

(1368) Eiholzer U, Grieser J, Schlumpf M, l'Allemand D. Clinical effects of treatment for hypogonadism in male adolescents with prader-labhart-willi syndrome. Hormone Research 2007;68(4):178-84. Drug intervention study
Ref ID: 5252

(1369) Eiholzer U, Meinhardt U, Petro R, Witassek F, Gutzwiller F, Gasser T. High-intensity training increases spontaneous physical activity in children: a randomized controlled study. J Pediatr 2010 February;156(2):242-6. No comparative control group
Ref ID: 88

(1370) Einfeld S, GÃ¶tz J, Holsinger RMD. Pathogenetic mechanisms in Down Syndrome. Journal of Intellectual Disability Research 2008 October;52(10):813. Review article
Ref ID: 3812

(1371) Eisele R, Maier E, Kinzl L, Gude U. [Stationary thromboprophylaxis in casualty surgery. Relevance of postoperative mobility and preexisting risk factors]. [German]. Unfallchirurg 2004 April;107(4):294-9. Not an exercise intervention study
Ref ID: 1638

(1372) Eisenmann JC, Pivarnik JM, Malina RM. Scaling peak VO2 to body mass in young male and female distance runners. Journal of Applied Physiology 2001 June;90(6):2172-80. Not a randomized controlled trial (RCT)
Ref ID: 1918

(1373) Eisenmann JC, Welk GJ, Wickel EE, Blair SN. Combined influence of cardiorespiratory fitness and body mass index on cardiovascular disease risk factors among 8-18 year old youth: The Aerobics Center Longitudinal Study. International Journal of Pediatric Obesity 2007;2(2):66-72. Not a randomized controlled trial (RCT)
Ref ID: 1173

(1374) Eisenmann JC, Gentile DA, Welk GJ, Callahan R, Strickland S, Walsh M, Walsh DA. SWITCH: rationale, design, and implementation of a community, school, and family-based intervention to modify behaviors related to childhood obesity. BMC Public Health 2008 June 29;8:223.:223. Description versus conduct of study
Ref ID: 175

(1375) Eisenmann JC, Subcommittee on Assessment in Pediatric Obesity Management Programs NAoCHaRI. Assessment of obese children and adolescents: a survey of pediatric obesity-management programs. Pediatrics 2011 September;128:Suppl-8. Survey or questionnaire
Ref ID: 2508

(1376) Ekblom O, Oddsson K, Ekblom B. Health-related fitness in Swedish adolescents between 1987 and 2001. Acta Paediatrica 2004 May;93(5):681-6. Cross-sectional study
Ref ID: 1620

(1377) Ekelund U, Anderssen SA, Froberg K, Sardinha LB, Andersen LB, Brage S, European Youth Heart Study Group. Independent associations of physical activity and cardiorespiratory fitness with metabolic risk factors in children: the European youth heart study. Diabetologia 2007 September;50(9):1832-40. Cross-sectional study
Ref ID: 1188

(1378) El-Hajj FG, Nabulsi M, Tamim H, Maalouf J, Salamoun M, Khalife H, Choucair M, Arabi A, Vieth R. Effect of vitamin D replacement on musculoskeletal parameters in school children: a randomized controlled trial. Journal of Clinical Endocrinology & Metabolism 2006 February;91(2):405-12. Diet Intervention or Supplement Study
Ref ID: 1423

(1379) El AW, El AS, Moseley L. Associations between physical activity and health parameters in adolescent pupils in Egypt. International Journal of Environmental Research and Public Health 2010;7:1649-69. Not All Participants were Overweight and/or Obese
Ref ID: 4759

(1380) Elbl L, Hrstkova H, Tomaskova I, Blazek B, Michalek J. Long-term serial echocardiographic examination of late anthracycline cardiotoxicity and its prevention by dexrazoxane in paediatric patients. European Journal of Pediatrics 2005 November;164(11):678-84. Drug intervention study
Ref ID: 1461

(1381) Elder JP, McGraw SA, Stone EJ, Reed DB, Harsha DW, Greene T, Wambsgans KC. CATCH: process evaluation of environmental factors and programs. Health Education Quarterly Suppl 2:S107-27, 1994 1994;S107-S127. Description of study from review or magazine or etc. (not the actual study)
Ref ID: 2248

(1382) Elder JP, McKenzie TL, Arredondo EM, Crespo NC, Ayala GX. Effects of a multi-pronged intervention on children's activity levels at recess: the Aventuras para Ninos study. Advances in Nutrition 2011 March;2(2):171S-6S. Not All Participants were Overweight and/or Obese
Ref ID: 2509

(1383) Elferink-Gemser MT, Visscher C, Lemmink KA, Mulder TW. Relation between multidimensional performance characteristics and level of performance in talented youth field hockey players. Journal of Sports Sciences 2004 November;22(11-12):1053-63. Not a randomized controlled trial (RCT)
Ref ID: 1558

(1384) Elia M, Ritz P. Total energy expenditure in the elderly. European Journal of Clinical Nutrition 2000 June 2;54(6):S92. Abstract
Ref ID: 511

(1385) Elia M. Changing concepts of nutrient requirements in disease: Implications for artificial nutritional.. Lancet 1995 May 20;345(8960):1279. Review article
Ref ID: 570

(1386) Eliakim A, Barstow TJ, Brasel JA, Ajie H, Lee WN, Renslo R, Berman N, Cooper DM. Effect of exercise training on energy expenditure, muscle volume, and maximal oxygen uptake in female adolescents. Journal of Pediatrics 1996 October;129(4):537-43. Not All Participants were Overweight and/or Obese
Ref ID: 2160

(1387) Eliakim A, Burke GS, Cooper DM. Fitness, fatness, and the effect of training assessed by magnetic resonance imaging and skinfold-thickness measurements in healthy adolescent females. American Journal of Clinical Nutrition 1997 August;66(2):223-31. Cross-sectional study
Ref ID: 2130

(1388) Eliakim A, Brasel JA, Mohan S, Wong WL, Cooper DM. Increased physical activity and the growth hormone-IGF-I axis in adolescent males. American Journal of Physiology 1998 July;275(1:Pt 2):t-14. Not All Participants were Overweight and/or Obese
Ref ID: 2085

(1389) Eliakim A, Makowski GS, Brasel JA, Cooper DM. Adiposity, lipid levels, and brief endurance training in nonobese adolescent males. International Journal of Sports Medicine 2000 July;21(5):332-7. Not All Participants were Overweight and/or Obese
Ref ID: 1963

(1390) Eliakim A, Scheett TP, Newcomb R, Mohan S, Cooper DM. Fitness, training, and the growth hormone-->insulin-like growth factor I axis in prepubertal girls. Journal of Clinical Endocrinology & Metabolism 2001 June;86(6):2797-802. Not All Participants were Overweight and/or Obese
Ref ID: 1915

(1391) Eliakim A, Scheett T, Allmendinger N, Brasel JA, Cooper DM. Training, muscle volume, and energy expenditure in nonobese American girls. Journal of Applied Physiology 2001 January;90(1):35-44. Not All Participants were Overweight and/or Obese
Ref ID: 1937

(1392) Eliakim A, Kaven G, Berger I, Friedland O, Wolach B, Nemet D. The effect of a combined intervention on body mass index and fitness in obese children and adolescents - a clinical experience. European Journal of Pediatrics 2002 August;161(8):449-54. Not a randomized controlled trial (RCT)
Ref ID: 1805

(1393) Eliakim A, Friedland O, Kowen G, Wolach B, Nemet D. Parental obesity and higher pre-intervention BMI reduce the likelihood of a multidisciplinary childhood obesity program to succeed--a clinical observation. Journal of Pediatric Endocrinology 2004 August;17(8):1055-61. Multiple interventions
Ref ID: 1586

(1394) Eliakim A, Barzilai M, Wolach B, Nemet D. Should we treat elevated thyroid stimulating hormone levels in obese children and adolescents? International Journal of Pediatric Obesity 2006;1(4):217-21. Inappropriate Outcomes
Ref ID: 1300

(1395) Eliakim A, Nemet D, Balakirski Y, Epstein Y. The effects of nutritional-physical activity school-based intervention on fatness and fitness in preschool children. J Pediatr Endocrinol Metab 2007 June;20(6):711-8. No exercise only group
Ref ID: 224

(1396) Eliasson AC, Forssberg H, Ikuta K, Apel I, Westling G, Johansson R. Development of Human Precision Grip .5. Anticipatory and Triggered Grip Actions During Sudden Loading. Experimental Brain Research 1995;106(3):425-33. Acute study
Ref ID: 5254

(1397) Eliasson K, Elfving B, Nordgren B, Mattsson E. Urinary incontinence in women with low back pain. Manual Therapy 2008 June;13(3):206-12. Survey or questionnaire
Ref ID: 975

(1398) Ellenbecker TS, Roetert EP. An isokinetic profile of trunk rotation strength in elite tennis players. Medicine & Science in Sports & Exercise 2004 November;36(11):1959-63. Not a randomized controlled trial (RCT)
Ref ID: 1575

(1399) Elliot DL, Moe EL, Goldberg L, DeFrancesco CA, Durham MB, Hix-Small H. Definition and outcome of a curriculum to prevent disordered eating and body-shaping drug use. Journal of School Health 2006 February;76(2):67-73. Survey or questionnaire
Ref ID: 1419

(1400) Elliott JG. Practitioner Review: School Refusal: Issues of Conceptualisation, Assessment, and Treatment. Journal of Child Psychology & Psychiatry & Allied Disciplines 1999 October;40(7):1001. Review article
Ref ID: 3574

(1401) Ellis DA, Janisse H, Naar-King S, Kolmodin K, Jen KL, Cunningham P, Marshall S. The effects of multisystemic therapy on family support for weight loss among obese African-American adolescents: findings from a randomized controlled trial. J Dev Behav Pediatr 2010 July;31(6):461-8. No exercise only group
Ref ID: 34

(1402) Ellis JA, Ponsonby AL, Pezic A, Williamson E, Cochrane JA, Dickinson JL, Dwyer T. APOE Genotype and Cardio-Respiratory Fitness Interact to Determine Adiposity in 8-Year-Old Children from the Tasmanian Infant Health Survey. PLoS ONE 2011;6(11). Cohort Study
Ref ID: 5255

(1403) Elloumi M, Ben Ounis O, Makni E, Van Praagh E, Tabka Z, Lac G. Effect of individualized weight-loss programmes on adiponectin, leptin and resistin levels in obese adolescent boys. Acta Paediatrica 2009;98(9):1487-93. No comparative control group
Ref ID: 5256

(1404) Elloumi M, Makni E, Ounis OB, Moalla W, Zbidi A, Zaoueli M, Lac G, Tabka Z. Six-minute walking test and the assessment of cardiorespiratory responses during weight-loss programmes in obese children. Physiotherapy research international : the journal for researchers and clinicians in physical therapy 2011;16:32-42. Primary outcome(s) not assessed
Ref ID: 4761

(1405) Ells LJ, Hillier FC, Shucksmith J, Crawley H, Harbige L, Shield J, Wiggins A, Summerbell CD. A systematic review of the effect of dietary exposure that could be achieved through normal dietary intake on learning and performance of school-aged children of relevance to UK schools. [Review] [50 refs]. BR J NUTR 2008 November;100(5):927-36. Review article
Ref ID: 883

(1406) Ellsworth DL, Coady SA, Chen W, Srinivasan SR, Elkasabany A, Gustat J, Boerwinkle E, Berenson GS. Influence of the beta 2-adrenergic receptor Arg16Gly polymorphism on longitudinal changes in obesity from childhood through young adulthood in a biracial cohort: the Bogalusa Heart Study. International Journal of Obesity 2002;26(7):928-37. Cross-sectional study
Ref ID: 5257

(1407) Elmahgoub SM, Lambers S, Stegen S, Van LC, Cambier D, Calders P. The influence of combined exercise training on indices of obesity, physical fitness and lipid profile in overweight and obese adolescents with mental retardation. European Journal of Pediatrics 2009 November;168(11):1327-33. Not a randomized controlled trial (RCT)
Ref ID: 675

(1408) Elmahgoub SS, Calders P, Lambers S, Stegen SM, Van LC, Cambier DC. The effect of combined exercise training in adolescents who are overweight or obese with intellectual disability: the role of training frequency. Journal of Strength & Conditioning Research 2011 August;25(8):2274-82. Not a randomized controlled trial (RCT)
Ref ID: 2510

(1409) Elnour A, Hambraeus L, Eltom M, Dramaix M, Bourdoux P, Caccetta RA-A, Kroft KD, Beilin LJ, Puddey IB, Jooste PL, Weight MJ, Lombard CJ, Zimmermann M, Adou P, Torresani T, Zeder C, Hurrell R, Hunt J, Roughead ZK. Current literature. Journal of Human Nutrition & Dietetics 13[3], 225-239. 2000. Abstract,
Ref ID: 3575

(1410) Elster J. Don't Burn Your Bridge Before You Come To It: Some Ambiguities and Complexities of Precommitment. Texas Law Review 2003 June;81(7):1751. Editorial or letter or comment
Ref ID: 3576

(1411) Emes C, Velde B, Moreau M, Murdoch DD, Trussell R. An activity based weight control program. Adapted Physical Activity Quarterly 1990;7:314-24. No exercise only group
Ref ID: 452

(1412) Eneli IU, Skybo T, Camargo CA, Jr. Weight loss and asthma: a systematic review. [Review] [68 refs]. Thorax 2008 August;63(8):671-6. Review article
Ref ID: 910

(1413) Enes CC, Slater B. Obesidade na adolescência e seus principais fatores determinantes^ipt
Obesity in adolescence and its main determinants^ien. Rev bras epidemiol 2010 March;13(1):163-71. Review article
Ref ID: 4107

(1414) Engel SM, Zhu C, Berkowitz GS, Calafat AM, Silva MJ, Miodovnik A, Wolff MS. Prenatal phthalate exposure and performance on the Neonatal Behavioral Assessment Scale in a multiethnic birth cohort. Neurotoxicology 2009;30(4):522-8. Cohort Study
Ref ID: 5258

(1415) Engstrom C, Davidson D. Hot Flashes. 10[4], 533-535. 2006. Oncology Nursing Society. Case-Control / Case Study,
Ref ID: 3577

(1416) Entin PL, Gest C, Trancik S, Richard CJ. Fuel oxidation in relation to walking speed: influence of gradient and external load. European Journal of Applied Physiology 2010 October;110(3):515-21. Not a randomized controlled trial (RCT)
Ref ID: 2511

(1417) Epstein LH, Wing RR, Koeske R, Valoski A. Effects of diet plus exercise on weight change in parents and children. J Consult Clin Psychol 1984 June;52(3):429-37. No exercise only group
Ref ID: 443

(1418) Epstein LH, Wing RR, Penner BC, Kress MJ. Effect of diet and controlled exercise on weight loss in obese children. J Pediatr 1985 September;107(3):358-61. No exercise only group
Ref ID: 448

(1419) Epstein LH, Kuller LH, Wing RR, Valoski A, McCurley J. The effect of weight control on lipid changes in obese children. American Journal of Diseases of Children 1989 April;143(4):454-7. Behavior Modification Intervention
Ref ID: 2325

(1420) Epstein LH, Valoski A, Wing RR, McCurley J. Ten-year follow-up of behavioral, family-based treatment for obese children. JAMA 1990 November 21;264(19):2519-23. No exercise only group
Ref ID: 439

(1421) Epstein LH, McCurley J, Wing RR, Valoski A. Five-year follow-up of family-based behavioral treatments for childhood obesity. J Consult Clin Psychol 1990 October;58(5):661-4. No exercise only group
Ref ID: 440

(1422) Epstein LH, McKenzie SJ, Valoski A, Klein KR, Wing RR. Effects of mastery criteria and contingent reinforcement for family-based child weight control. Addict Behav 1994 March;19(2):135-45. No exercise only group
Ref ID: 434

(1423) Epstein LH, Valoski A, Wing RR, McCurley J. Ten-year outcomes of behavioral family-based treatment for childhood obesity. Health Psychol 1994 September;13(5):373-83. No exercise only group
Ref ID: 432

(1424) Epstein LH, Valoski AM, Vara LS, McCurley J, Wisniewski L, Kalarchian MA, Klein KR, Shrager LR. Effects of decreasing sedentary behavior and increasing activity on weight change in obese children. Health Psychol 1995 March;14(2):109-15. No comparative control group
Ref ID: 431

(1425) Epstein LH, Valoski AM, Kalarchian MA, McCurley J. Do children lose and maintain weight easier than adults: a comparison of child and parent weight changes from six months to ten years. Obes Res 1995 September;3(5):411-7. No exercise only group
Ref ID: 430

(1426) Epstein LH, Coleman KJ, Myers MD. Exercise in treating obesity in children and adolescents. [Review] [52 refs]. Medicine & Science in Sports & Exercise 1996 April;28(4):428-35. Review article
Ref ID: 2179

(1427) Epstein LH, Saelens BE, Myers MD, Vito D. Effects of decreasing sedentary behaviors on activity choice in obese children. Health Psychol 1997 March;16(2):107-13. Not an exercise intervention study
Ref ID: 426

(1428) Epstein LH. Integrating theoretical approaches to promote physical activity. American Journal of Preventive Medicine 1998;15(4):257-65. Review article
Ref ID: 5259

(1429) Epstein LH, Myers MD, Raynor HA, Saelens BE. Treatment of pediatric obesity. [Review] [135 refs]. Pediatrics 1998 March;101(3:Pt 2):t-70. Review article
Ref ID: 2108

(1430) Epstein LH, Paluch RA, Gordy CC, Dorn J. Decreasing sedentary behaviors in treating pediatric obesity. Arch Pediatr Adolesc Med 2000 March;154(3):220-6. No exercise only group, No comparative control group
Ref ID: 403

(1431) Epstein LH, Paluch RA, Raynor HA. Sex differences in obese children and siblings in family-based obesity treatment. Obes Res 2001 December;9(12):746-53. No comparative control group
Ref ID: 390

(1432) Epstein LH, Paluch RA, Consalvi A, Riordan K, Scholl T. Effects of manipulating sedentary behavior on physical activity and food intake. Journal of Pediatrics 2002 March;140(3):334-9. Study less than 4 weeks
Ref ID: 1841

(1433) Epstein LH, Paluch RA, Kilanowski CK, Raynor HA. The effect of reinforcement or stimulus control to reduce sedentary behavior in the treatment of pediatric obesity. Health Psychol 2004 July;23(4):371-80. No exercise only group, No comparative control group
Ref ID: 327

(1434) Epstein LH, Roemmich JN, Saad FG, Handley EA. The value of sedentary alternatives influences child physical activity choice. International journal of behavioral medicine 2004;11:236-42. Not All Participants were Overweight and/or Obese
Ref ID: 4762

(1435) Epstein LH, Roemmich JN, Paluch RA, Raynor HA. Physical activity as a substitute for sedentary behavior in youth. Annals of Behavioral Medicine 2005 June;29(3):200-9. Study less than 4 weeks
Ref ID: 1507

(1436) Epstein LH, Roemmich JN, Stein RI, Paluch RA, Kilanowski CK. The challenge of identifying behavioral alternatives to food: clinic and field studies. Ann Behav Med 2005 December;30(3):201-9. No exercise only group, No comparative control group
Ref ID: 292

(1437) Epstein LH, Roemmich JN, Paluch RA, Raynor HA. Influence of changes in sedentary behavior on energy and macronutrient intake in youth. American Journal of Clinical Nutrition 2005 February;81(2):361-6. Not All Participants were Overweight and/or Obese
Ref ID: 1546

(1438) Epstein LH, Roemmich JN, Robinson JL, Paluch RA, Winiewicz DD, Fuerch JH, Robinson TN. A randomized trial of the effects of reducing television viewing and computer use on body mass index in young children. Arch Pediatr Adolesc Med 2008 March;162(3):239-45. Not an exercise intervention study
Ref ID: 190

(1439) Epstein LH, Roemmich JN, Cavanaugh MD, Paluch RA. The motivation to be sedentary predicts weight change when sedentary behaviors are reduced. International Journal of Behavioral Nutrition & Physical Activity 2011;8:13. Study less than 4 weeks
Ref ID: 2512

(1440) Erickson C, Stigler K, Wink L, Mullett J, Kohn A, Posey D, McDougle C. A prospective open-label study of aripiprazole in fragile X syndrome. Psychopharmacology 2011 July;216(1):85-90. Drug intervention study
Ref ID: 3578

(1441) Eriksen J, Mujinja P, Warsame M, Nsimba S, Kouyaté B, Gustafsson LL, Jahn A, Müller O, Sauerborn R, Tomson G. Effectiveness of a community intervention on malaria in rural Tanzania - a randomised controlled trial. African Health Sciences 2010;10:332-40. Survey or questionnaire
Ref ID: 4763

(1442) Erwin HE, Woods AM, Woods MK, Castelli DM. Chapter 6: Children's Environmental Access in Relation to Motor Competence, Physical Activity, and Fitness. Journal of Teaching in Physical Education 2007 October 1;26(4):404-15. Survey or questionnaire
Ref ID: 3902

(1443) Esco MR, Olson MS, Williford H. Relationship of push-ups and sit-ups tests to selected anthropometric variables and performance results: a multiple regression study. Journal of Strength & Conditioning Research 2008 November;22(6):1862-8. Cohort Study
Ref ID: 865

(1444) Escobar-Chaves SL, Markham CM, Addy RC, Greisinger A, Murray NG, Brehm B. The Fun Families Study: intervention to reduce children's TV viewing. Obesity (Silver Spring) 2010 February;18 Suppl 1:S99-101.:S99-101. No exercise only group
Ref ID: 64

(1445) Espana-Romero V, Ortega FB, Ruiz JR, Artero EG, Martinez-Gomez D, Vicente-Rodriguez G, Moliner-Urdiales D, Gracia-Marco L, Ciarapica D, Widhalm K, Castillo Garzon MJ, Sjostrom M, Moreno LA. Role of cardiorespiratory fitness on the association between physical activity and abdominal fat content in adolescents: the HELENA study. International Journal of Sports Medicine 2010 October;31(10):679-82. Cross-sectional study
Ref ID: 2513

(1446) Espinoza-Navarro O, Vega C, Urrutia A, Moreno A, Rodríguez H. Patrones antropométricos y consumo máximo de oxígeno (VO2) entre niños escolares chilenos Aymaras y no Aymaras de 10 a 12 años, que viven en altura (3.500 msnm) y en la planicie (500 msnm)^ies
Anthropometric patterns and oxygen consumption (VO2) of school-children Aymara and non-Aymara of 10-12 years, living in high altitude (3500m) and the plain (500 m), from Chile^ien. Int J Morphol 2009 December;27(4):1313-8. Cross-sectional study
Ref ID: 4108

(1447) Esquivel Lauzurique M, González Fernández C. Desarrollo físico y nutrición de preescolares habaneros según nuevos patrones de crecimiento de la OMS^ies
Physical development and nutritional status of preschool-age children in Havana according to the new WHO Growth References^ien. Rev cuba salud pública 2009 March;35(1):0. Cross-sectional study
Ref ID: 4109

(1448) Esquivel Solis V. Factores asociados a la obesidad en mujeres en edad fértil. Rev costarric salud pública 2004 December;13(25):42-7. Cross-sectional study
Ref ID: 4110

(1449) Esquivel Solís V, Alvarado MV. Estado nutricional de mujeres con sobrepeso y obesidad del área de cobertura del programa de atención integral en salud (PAIS) 2006^ies. Acta méd costarric 2009 December;51(4):222-8. Study limited to adults
Ref ID: 4111

(1450) Estabrooks PA, Shoup JA, Gattshall M, Dandamudi P, Shetterly S, Xu S. Automated telephone counseling for parents of overweight children: a randomized controlled trial. Am J Prev Med 2009 January;36(1):35-42. No exercise only group
Ref ID: 141

(1451) Estalella I, Rica I, Perez de NG, Bilbao JR, Vazquez JA, San Pedro JI, Busturia MA, Castano L, Spanish MODY Group. Mutations in GCK and HNF-1alpha explain the majority of cases with clinical diagnosis of MODY in Spain. Clinical Endocrinology 2007 October;67(4):538-46. Not an exercise intervention study
Ref ID: 1164

(1452) Eston RG, Rowlands AV, Ingledew DK. Validity of heart rate, pedometry, and accelerometry for predicting the energy cost of children's activities. Journal of Applied Physiology 1998 January;84(1):362-71. Cross-sectional study
Ref ID: 2104

(1453) Estrada MC, Velásquez MI, Orrego M. Neumomediastino espontáneo: reporte de un caso y revisión de la literatura^ies
Spontaneous pneumomediastinum: a case report and literature review^ien. CES med 2009 December;23(2):47-53. Case-Control / Case Study
Ref ID: 4112

(1454) Evangelista LS, Doering LV, Lennie T, Moser DK, Hamilton MA, Fonarow GC, Dracup K. Usefulness of a home-based exercise program for overweight and obese patients with advanced heart failure. Am J Cardiol 2006 March 15;97(6):886-90. Study limited to adults
Ref ID: 284

(1455) Evans BW, Claiborne JM. Health Related Physical Fitness: Who, What, Why, and How. 1982 Jan 1. ¬

Review article
Ref ID: 3903

(1456) Evenson KR, Scott MM, Cohen DA, Voorhees CC. Girls' perception of neighborhood factors on physical activity, sedentary behavior, and BMI. Obesity 2007 February;15(2):430-45. Survey or questionnaire
Ref ID: 1276

(1457) Evenson KR, Murray DM, Birnbaum AS, Cohen DA. Examination of perceived neighborhood characteristics and transportation on changes in physical activity and sedentary behavior: The Trial of Activity in Adolescent Girls. Health & Place 2010;16:977-85. Not a randomized controlled trial (RCT)
Ref ID: 4764

(1458) Everitt AV, Hilmer SN, Brand-Miller JC, Jamieson HA, Truswell AS, Sharma AP, Mason RS, Morris BJ, Le Couteur DG. Dietary approaches that delay age-related diseases. [Review] [336 refs]. Clin Interventions Aging 2006;1(1):11-31. Review article
Ref ID: 1297

(1459) Exl BM, Deland U, Wall M, Preysch U, Secretin MC, Shmerling DH. Zug-Frauenfeld nutritional survey ('Zuff Study'): Allergen-reduced nutrition in a normal infant population and its health-related effects: Results at the age of six months. Nutrition Research 1998;18:1443-62. Subjects less than 2 years old
Ref ID: 4765

(1460) Eyler AA, Brownson RC, Doescher MP, Evenson KR, Fesperman CE, Litt JS, Pluto D, Steinman LE, Terpstra JL, Troped PJ, Schmid TL. Policies related to active transport to and from school: a multisite case study. Health Education Research 2008 December;23(6):963-75. Case-Control / Case Study
Ref ID: 860

(1461) Ezendam NP, Oenema A, van de Looij-Jansen PM, Brug J. Design and evaluation protocol of "FATaintPHAT", a computer-tailored intervention to prevent excessive weight gain in adolescents. BMC Public Health 2007 November 12;7:324.:324. Description versus conduct of study
Ref ID: 212

(1462) Ezendam NP, Brug J, Oenema A. Evaluation of the Web-based computer-tailored FATaintPHAT intervention to promote energy balance among adolescents: results from a school cluster randomized trial. Archives of Pediatrics & Adolescent Medicine 2012 March;166(3):248-55. Multiple interventions
Ref ID: 2514

(1463) Fabricatore AN, Wadden TA, Ebbeling CB, Thomas JG, Stallings VA, Schwartz S, Ludwig DS. Targeting dietary fat or glycemic load in the treatment of obesity and type 2 diabetes: a randomized controlled trial. Diabetes Research & Clinical Practice 2011 April;92(1):37-45. Study limited to adults
Ref ID: 1059

(1464) Fabricatore AN, Wadden TA, Higginbotham AJ, Faulconbridge LF, Nguyen AM, Heymsfield SB, Faith MS. Intentional weight loss and changes in symptoms of depression: a systematic review and meta-analysis. [Review]. International Journal of Obesity 2011 November;35(11):1363-76. Review article
Ref ID: 1047

(1465) Fagondes SC, Moreira GA. Apneia obstrutiva do sono em crianças^ipt
Obstructive sleep apnea in children^ien. J bras pneumol 2010 June;36(supl.2):57-61. Review article
Ref ID: 4113

(1466) Fagundes AL, Ribeiro DC, Naspitz L, Garbelini LE, Vieira JK, Silva APd, Lima VdO, Fagundes DJ, Compri PC, Juliano Y. Prevalência de sobrepeso e obesidade em escolares da região de Parelheiros do município de São Paulo^ipt. Rev paul pediatr 2008 September;26(3):212-7. Cross-sectional study
Ref ID: 4114

(1467) Fahrenwald NL, Atwood JR, Walker SN, Johnson DR, Berg K. A randomized pilot test of "Moms on the Move": a physical activity intervention for WIC mothers. Annals of behavioral medicine : a publication of the Society of Behavioral Medicine 2004;27:82-90. Study limited to adults
Ref ID: 4766

(1468) Fahrenwald NL, Atwood JR, Johnson DR. Mediator analysis of Moms on the move. Western journal of nursing research 2005;27:271-91. Study limited to adults
Ref ID: 4767

(1469) Faigenbaum AD, Westcott WL, Loud RL, Long C. The effects of different resistance training protocols on muscular strength and endurance development in children. Pediatrics 1999;104:e5. Not All Participants were Overweight and/or Obese
Ref ID: 900

(1470) Faigenbaum AD, Loud RL, O'Connell J, Glover S, O'Connell J, Westcott WL. Effects of different resistance training protocols on upper-body strength and endurance development in children. Journal of strength and conditioning research / National Strength & Conditioning Association 2001;15:459-65. Not a randomized controlled trial (RCT)
Ref ID: 795

(1471) Faigenbaum AD, Milliken LA, Loud RL, Burak BT, Doherty CL, Westcott WL. Comparison of 1 and 2 days per week of strength training in children. Research quarterly for exercise and sport 2002;73:416-24. Not a randomized controlled trial (RCT)
Ref ID: 4768

(1472) Faigenbaum AD, Farrell A, Fabiano M, Radler T, Naclerio F, Ratamess NA, Kang J, Myer GD. Effects of integrative neuromuscular training on fitness performance in children. Pediatric Exercise Science 2011 November;23(4):573-84. Not All Participants were Overweight and/or Obese
Ref ID: 1034

(1473) Faintuch J, Souza SAF, Valezi AC, Sant'Anna AF, Gama-Rodrigues JJ. Pulmonary function and aerobic capacity in asymptomatic bariatric candidates with very severe morbid obesity. Rev Hosp Clin Fac Med Univ São Paulo 2004 August;59(4):181-6. Prospective Study
Ref ID: 4115

(1474) Fairley JA, Sejdic E, Chau T. The effect of treadmill walking on the stride interval dynamics of children. Human Movement Science 2010;29(6):987-98. Acute study
Ref ID: 5261

(1475) Faith MS, Berman N, Heo M, Pietrobelli A, Gallagher D, Epstein LH, Eiden MT, Allison DB. Effects of contingent television on physical activity and television viewing in obese children. Pediatrics 2001 May;107(5):1043-8. Behavior Modification Intervention, No comparative control group
Ref ID: 397

(1476) Faith MS, Van HL, Appel LJ, Burke LE, Carson JA, Franch HA, Jakicic JM, Kral TV, Odoms-Young A, Wansink B, Wylie-Rosett J, American Heart Association Nutrition and Obesity Committees of the Council on Nutrition, Physical Activity and Metabolism, Council on Clinical Cardiology, Council on Cardiovascular Disease in the Young, Council on Cardiovascular Nursing, Council on Epidemiology and Prevention aCotKiCD. Evaluating parents and adult caregivers as "agents of change" for treating obese children: evidence for parent behavior change strategies and research gaps: a scientific statement from the American Heart Association. Circulation 2012 March 6;125(9):1186-207. Review article
Ref ID: 2517

(1477) Falvo DR, Parker RM. Ethics in Rehabilitation Education and Research. Rehabilitation Counseling Bulletin 2000;43(4):197-214. Review article
Ref ID: 3579

(1478) Fan H, Zhang XQ, Li J. [Effects of life style intervention on obesity-related vascular dysfunction in children]. Zhonghua Liu Xing Bing Xue Za Zhi 2008 July;29(7):672-5. No exercise only group, No comparative control group
Ref ID: 148

(1479) Fan JG, Zhu J, Li XJ, Chen L, Li L, Dai F, Li F, Chen SY. Prevalence of and risk factors for fatty liver in a general population of Shanghai, China. Journal of Hepatology 2005 September;43(3):508-14. Study limited to adults
Ref ID: 1487

(1480) Fan YF, Fan YB, Li ZY, Lv CS, Luo DL. Natural Gaits of the Non-Pathological Flat Foot and High-Arched Foot. PLoS ONE 2011;6(3). Not an exercise intervention study
Ref ID: 5262

(1481) Fang PC, Kuo HK, Huang CB, Ko TY, Chen CC, Chung MY. The effect of supplementation of docosahexaenoic acid and arachidonic acid on visual acuity and neurodevelopment in larger preterm infants. Chang Gung medical journal 2005;28:708-15. Subjects less than 2 years old
Ref ID: 4769

(1482) Fantino M. Is the energy supplied by caloric sweetened beverages regulated? Sciences des Aliments 2007;27(4-5):301-10. Review article
Ref ID: 5263

(1483) Faria AMC, Weiner HL. Oral tolerance. Immunological Reviews 2005 August;206(1):232-59. Review article
Ref ID: 3580

(1484) Faria ERd, Franceschini SdCC, Peluzio MdCG, Priore SE. Síndrome Metabólica em adolescentes: uma atualização^ipt
Metabolic Syndrome in adolescents: an update^ien. Nutrire Rev Soc Bras Aliment Nutr 2009 August;34(2):179-94. Review article
Ref ID: 4116

(1485) Farias Júnior JCd, Mendes JKF, Barbosa DBM, Lopes AdS. Fatores de risco cardiovascular em adolescentes: prevalência e associação com fatores sociodemográficos^ipt
Cardiovascular risk factors for adolescents: prevalence and association with sociodemographic factors^ien. Rev bras epidemiol 2011 March;14(1):50-62. Not a randomized controlled trial (RCT)
Ref ID: 4117

(1486) Farias Júnior JCd, Lopes AdS, Mota J, Hallal PC. Prática de atividade física e fatores associados em adolescentes no Nordeste do Brasil^ipt
Physical activity practice and associated factors in adolescents in Northeastern Brazil^ien
Práctica de actividad física y factores asociados en adolescentes en el Noreste de Brasil^ies. Rev saúde pública 2012 June;46(3):505-15. Survey or questionnaire
Ref ID: 4118

(1487) Farias ES, Paula F, Carvalho WR, Goncalves EM, Baldin AD, Guerra-Junior G. Influence of programmed physical activity on body composition among adolescent students. Jornal de Pediatria 2009 January;85(1):28-34. Not a randomized controlled trial (RCT)
Ref ID: 800

(1488) Farias EdS, Petroski EL. Estado nutricional e atividade física de escolares da cidade de Porto Velho, RO^ipt. Rev bras cineantropom desempenho hum 2003;5(1). Survey or questionnaire
Ref ID: 4119

(1489) Faude O, Meyer T, Scharhag J, Weins F, Urhausen A, Kindermann W. Volume vs. intensity in the training of competitive swimmers. International Journal of Sports Medicine 2008 November;29(11):906-12. Not All Participants were Overweight and/or Obese
Ref ID: 873

(1490) Faude O, Kerper O, Multhaupt M, Winter C, Beziel K, Junge A, Meyer T. Football to tackle overweight in children. Scand J Med Sci Sports 2010 April;20 Suppl 1:103-10. Epub;%2010 Feb 2.:103-10. No comparative control group
Ref ID: 62

(1491) Faulkner RA, Forwood MR, Beck TJ, Mafukidze JC, Russell K, Wallace W. Strength indices of the proximal femur and shaft in prepubertal female gymnasts. Medicine & Science in Sports & Exercise 2003 March;35(3):513-8. Cross-sectional study
Ref ID: 1770

(1492) Favier R, Caceres E, Koubi H, Sempore B, Sauvain M, Spielvogel H. Effects of coca chewing on hormonal and metabolic responses during prolonged submaximal exercise. Journal of Applied Physiology 1996 February;80(2):650-5. Not an exercise intervention study
Ref ID: 2155

(1493) Fechner H, Pinkert S, Geisler A, Poller W, Kurreck J. Pharmacological and Biological Antiviral Therapeutics for Cardiac Coxsackievirus Infections. Molecules 2011 October;16(10):8475-503. Review article
Ref ID: 3581

(1494) Fehily AM, Coles RJ, Evans WD, Elwood PC. Factors affecting bone density in young adults. American Journal of Clinical Nutrition 56(3):579-86, 1992 Sep 1992;(3):579-86. Follow-up Study
Ref ID: 2961

(1495) Feinberg I, Higgins LM, Khaw WY, Campbell IG. The adolescent decline of NREM delta, an indicator of brain maturation, is linked to age and sex but not to pubertal stage. American journal of physiology Regulatory, integrative and comparative physiology 2006;291:R1724-R1729. Not an exercise intervention study
Ref ID: 4770

(1496) Feinstein S, Rinat C, Becker-Cohen R, Ben-Shalom E, Schwartz SB, Frishberg Y. The outcome of chronic dialysis in infants and toddlers - advantages and drawbacks of haemodialysis. Nephrology Dialysis Transplantation 2008;23(4):1336-45. Not an exercise intervention study
Ref ID: 5264

(1497) Feitosa MF, Rice T, Rosmond R, Borecki IB, An P, Gagnon J, Leon AS, Skinner JS, Wilmore JH, Bouchard C, Rao DC. A genetic study of cortisol measured before and after endurance training: the HERITAGE Family Study. Metabolism: Clinical & Experimental 2002 March;51(3):360-5. Inappropriate Outcomes
Ref ID: 1844

(1498) Feitosa MF, Borecki IB, Rankinen T, Leon AS, Skinner JS, Wilmore JH, Bouchard C, Rao DC. Lack of pleiotropic genetic effects between adiposity and sex hormone-binding globulin concentrations before and after 20 weeks of exercise training: the HERITAGE family study. Metabolism: Clinical & Experimental 2003 January;52(1):35-41. Inappropriate Outcomes
Ref ID: 1778

(1499) Feld JJ, Hoofnagle JH. Mechanism of action of interferon and ribavirin in treatment of hepatitis C. Nature 2005 August 18;436(7053):967-72. Drug intervention study
Ref ID: 3582

(1500) Feldman NT. Narcolepsy. Southern Medical Journal 2003 March;96(3):277. Review article
Ref ID: 3583

(1501) Fenichel GM, Florence JM, Pestronk A, Mendell JR, Moxley RT, Griggs RC, Brooke MH, Miller JP, Robison J, King W. Long-term benefit from prednisone therapy in Duchenne muscular dystrophy. Neurology 1991;41:1874-7. Drug intervention study
Ref ID: 4771

(1502) Fenn P. Assessment and management of abdominal obesity in patients with type 2 diabetes. (Cover story). Nursing Standard 2007 February 28;21(25):37-44. Review article
Ref ID: 3584

(1503) Ferber SG, Kuint J, Weller A, Feldman R, Dollberg S, Arbel E, Kohelet D. Massage therapy by mothers and trained professionals enhances weight gain in preterm infants. Early human development 2002;67:37-45. Subjects less than 2 years old
Ref ID: 770

(1504) Ferguson MA, Gutin B, Le NA, Karp W, Litaker M, Humphries M, Okuyama T, Riggs S, Owens S. Effects of exercise training and its cessation on components of the insulin resistance syndrome in obese children. Int J Obes Relat Metab Disord 1999 August;23(8):889-95. Same subjects as another study already included
Ref ID: 408

(1505) Ferguson MA, Gutin B, Owens S, Barbeau P, Tracy RP, Litaker M. Effects of physical training and its cessation on the hemostatic system of obese children. American Journal of Clinical Nutrition 1999 June;69(6):1130-4. Same subjects as another study already included
Ref ID: 2024

(1506) Ferguson TB, Syrotuik DG. Effects of creatine monohydrate supplementation on body composition and strength indices in experienced resistance trained women. Journal of Strength & Conditioning Research 2006 November;20(4):939-46. Diet Intervention or Supplement Study
Ref ID: 1311

(1507) Fermino RC, Rech CR, Hino AAF, Rodriguez Añez CR, Reis RS. Atividade física e fatores associados em adolescentes do ensino médio de Curitiba, Brasil^ipt
Physical activity and associated factors in high-school adolescents in Southern Brazil^ien
Actividad física y factores asociados en adolescentes de enseñanza secundaria de Curitiba, Sur de Brasil^ies. Rev saúde pública 2010 December;44(6):986-95. Cross-sectional study
Ref ID: 4121

(1508) Fernald LC, Gertler PJ, Neufeld LM. 10-year effect of Oportunidades, Mexico's conditional cash transfer programme, on child growth, cognition, language, and behaviour: a longitudinal follow-up study. Lancet 2009;374:1997-2005. Follow-up Study
Ref ID: 4772

(1509) Fernandes PS, Bernardo CDO, Campos RMMB, De-Vasconcelos FDAG. Evaluating the effect of nutritional education on the prevalence of overweight/obesity and on foods eaten at primary schools. Jornal de Pediatria 2009;85:315-21. Diet Intervention Study
Ref ID: 4773

(1510) Fernandes RA, Codogno JS, Cardoso JR, Ronque ERV, Freitas Júnior IF, Oliveira AR. Fatores associados ao excesso de peso entre adolescentes de diferentes redes de ensino do município de Presidente Prudente, São Paulo^ipt
Factors associated with overweight among adolescents in different school systems in the municipality of Presidente Prudente in the State of São Paulo^ien. Rev bras saúde matern infant 2009 December;9(4):443-9. Cross-sectional study
Ref ID: 4123

(1511) Fernandes RA, Oliveira ARd, Freitas Júnior IF. Correlação entre diferentes indicadores de adiposidade corporal e atividade física habitual em jovens do sexo masculino^ipt. Rev bras cineantropom desempenho hum 2006 December;8(4). Survey or questionnaire
Ref ID: 598

(1512) Fernandes RA, Christofaro DGD, Cucato GG, Agostini L, Oliveira ARd, Freitas Júnior IF. Nutritional status, physical activity level, waist circumference, and flexibility in brazilian boys^ien. Rev bras cineantropom desempenho hum 2007 September;9(4). Cross-sectional study
Ref ID: 4124

(1513) Fernandes RA, Casonatto J, Christofaro DGD, Ronque ERV, Oliveira ARd, Freitas Júnior IF. Riscos para o excesso de peso entre adolescentes de diferentes classes socioeconômicas^ipt. Rev Assoc Med Bras (1992) 2008 August;54(4):334-8. Cross-sectional study
Ref ID: 4125

(1514) Fernandes RA, Casonatto J, Christofaro DGD, Cucato GG, Romanzini M, Ronque ERV. Aptidão cardiorrespiratória, excesso de peso e pressão arterial elevada em adolescentes^ipt
Cardiorespiratory fitness, surplus weight and high blood pressure in adolescents^ien. Rev bras med esporte 2010 December;16(6):404-7. Cross-sectional study
Ref ID: 4126

(1515) Fernandes RA, Casonatto J, Christofaro DGD, Buonani C, Oliveira ARd, Freitas Júnior IF. Influência da atividade e inatividade física na composição corporal e adiposidade central^ipt. Motriz rev educ fís (Impr ) 2010 March;16(1):43-9. Cross-sectional study
Ref ID: 4127

(1516) Fernandes RA, Christofaro DGD, Casonatto J, Codogno JS, Rodrigues E, Cardoso M, Kawaguti S, Zanesco A. Prevalência de dislipidemia em indivíduos fisicamente ativos durante a infância, adolescência e idade adulta^ipt
Prevalence of dyslipidemia in individuals physically active during childhood, adolescence and adult age^ien. Arq bras cardiol 2011 October;97(4):317-23. Cross-sectional study
Ref ID: 4128

(1517) Fernandes TL, Protta TR, Fregni F, Neto RB, Pedrinelli A, Camanho GL, Hernandez AJ. Isokinetic muscle strength and knee function associated with double femoral pin fixation and fixation with interference screw in anterior cruciate ligament reconstruction. Knee Surgery, Sports Traumatology, Arthroscopy 2012 February;20(2):275-80. Not an exercise intervention study
Ref ID: 2518

(1518) Fernandez-de-las-Penas C, Hernandez-Barrera V, Alonso-Blanco C, Palacios-Cena D, Carrasco-Garrido P, Jimenez-Sanchez S, Jimenez-Garcia R. Prevalence of neck and low back pain in community-dwelling adults in Spain: a population-based national study. Spine 2011 February 1;36(3):E213-E219. Study limited to adults
Ref ID: 2519

(1519) Fernandez AC, Cintra IP, Sawaya AL, Fisberg M, Silva AC. Respostas metabólicas e cardiorrespiratórias ao exercício máximo e submáximo em meninas eutróficas e com desnutriçäo pregressa. Rev Assoc Med Bras (1992) 2000 December;46(4):312-9. Not a randomized controlled trial (RCT)
Ref ID: 4129

(1520) Fernandez AC, Mello MTd, Tufik S, Castro PMd, Fisberg M. Influência do treinamento aeróbio e anaeróbio na massa de gordura corporal de adolescentes obesos^ipt
Influence of the aerobic and anaerobic training on the body fat mass in obese adolescents^ien. Rev bras med esporte 2004 June;10(3):152-64. Diet & Exercise intervention
Ref ID: 4130

(1521) Fernandez F, I, Pascual dlP, Investigadores del Pg. [Predictive value of metabolic syndrome in pregnancy for the development of diabetes mellitus and factors of short-term vascular risk for mother and child after birth (gestaMET)]. [Spanish]. Atencion Primaria 2006 May 31;37(9):517-21. Study limited to adults
Ref ID: 1386

(1522) Fernández Ortega JA, González M, Martha Farfán F. Modelo teórico, agentes, estrategias, duración, escenarios para la implementación, grado escolar e indicadores de resultados utilizados en los programas de promoción de la actividad física en el contexto escolar^ies
Theoretical model, agents, strategies, duration, spaces of implementation, school grades and performance indicators, used in programs to promote physical activity in school context^ien. MedUNAB 2011 August;14(2):121-31. Review article
Ref ID: 4131

(1523) Fernhall B, Otterstetter M. Attenuated responses to sympathoexcitation in individuals with Down syndrome. J Appl Physiol 2003 June;94(6):2158-65. Acute study
Ref ID: 370

(1524) Ferns SJ, Wehrmacher WH, Serratto M. Effects of obesity and gender on exercise capacity in urban children.[Erratum appears in Gend Med. 2011 Oct;8(5):342]. Gender Medicine 2011 August;8(4):224-30. Observational study
Ref ID: 2520

(1525) Ferrara A, Ehrlich SF. Strategies for diabetes prevention before and after pregnancy in women with GDM. [Review]. Current Diabetes Reviews 2011 March;7(2):75-83. Review article
Ref ID: 2521

(1526) Ferrari GLd, Silva LJ, Ceschini FL, Oliveira LC, Douglas R, Matsudo VKR. Influência da maturação sexual na aptidão física de escolares do município de Ilhabela: um estudo longitudinal^ipt. Rev bras ativ fís saúde 2008;13(3). Not an exercise intervention study
Ref ID: 4132

(1527) Ferrari TK, Ferrari GLd, Silva Júnior JPd, Silva LJd, Oliveira LC, Matsudo VKR. Modificações da adiposidade em escolares de acordo com o estado nutricional: análise de 20 anos^ipt
Modifications of adiposity in school-age children according to nutritional status: a 20-year analysis^ien. J pediatr (Rio J ) 2012 June;88(3):239-45. Not an exercise intervention study
Ref ID: 4133

(1528) Ferraz ST, Frônio JdS, Neves LAT, Demarchi RS, Vargas ALdA, Ghetti FdF, Filgueiras MST. Programa de follow-up de recém-nascidos de alto risco: relato da experiência de uma equipe interdisciplinar^ipt
Follow-up program of high risk neonates: report of the experience of an interdisciplinary team^ien. Rev APS 2010 March;13(1). Follow-up Study
Ref ID: 4134

(1529) Ferreira AD, César CC, Malta DC, Souza Andrade ACd, Ramos CGC, Proietti FA, Bernal RTI, Caiaffa WT. Validade de estimativas obtidas por inquérito telefônico: comparação entre VIGITEL 2008 e inquérito Saúde em Beagá^ipt
Validity of data collected by telephone survey: a comparison of VIGITEL 2008 and 'Saúde em Beagá' survey^ien. Rev bras epidemiol 2011 September;14(supl.1):16-30. Survey or questionnaire
Ref ID: 4135

(1530) Ferreira AMd, Yonamine CY, Fujisawa DS, Lavado EL. A criança com paralisia cerebral: características clínicas e fisioterapia^ipt. Temas desenvolv 2008 August;16(93):113-7. Retrospective study
Ref ID: 4136

(1531) Ferreira AM, Bergamasco NHP. Análise comportamental de recém-nascidos pré-termos incluídos em um programa de estimulação tátil-cinestésica durante a internação hospitalar^ipt
Behavioral analysis of preterm neonates included in a tactile and kinesthetic stimulation program during hospitalization^ien. Rev bras fisioter 2010 April;14(2):141-8. Subjects less than 2 years old
Ref ID: 4137

(1532) Ferreira PL, Coelho VACC, Cesar MdC, Tolocka RE. Avaliação da saúde, fatores de risco e estado nutricional de crianças e adultos freqüentadores de um programa de natação^ipt. Rev bras ativ fís saúde 2006 December;11(3). Cross-sectional study
Ref ID: 4138

(1533) Ferreira S, Marins JCB, Silva LCd, Lunz W, Pimentel GGdA, Migliorini EM. Determinação de perfil de repetições máximas no exercício de extensão de pernas e supino reto com diferentes percentuais de força^ipt. Rev educ fis 2006 December;17(2):149-59. Not a randomized controlled trial (RCT)
Ref ID: 4139

(1534) Ferretti G, Narici MV, Binzoni T, Gariod L, Lebas JF, Reutenauer H, Cerretelli P. Determinants of Peak Muscle Power - Effects of Age and Physical Conditioning. European Journal of Applied Physiology and Occupational Physiology 1994;68(2):111-5. Not a randomized controlled trial (RCT)
Ref ID: 5265

(1535) Ferriani MdGC, Dechen S, Dias TS, Iossi MA. A percepção de saúde para adolescentes obesos. Rev bras enferm 2000 December;53(4):537-43. Not a randomized controlled trial (RCT)
Ref ID: 4140
[truncated: 934,067 more chars]
