# Supplementary material for: Effects of exercise on BMI z-score in overweight and obese children and adolescents: a systematic review with meta-analysis
Source: BMC Pediatr. 2014 Sep 9;14:225. doi: 10.1186/1471-2431-14-225 (PMC4180550; doi:10.1186/1471-2431-14-225)
Supplement: Supplementary file 4 — Additional file 4: Cochrane risk of bias results for each item from each included study. (DOCX 15 KB) [file 12887_2014_1161_MOESM4_ESM.docx]

Additional File 3. Cochrane risk of bias results for each item from each included study.

|  | Selection Bias | | Performance Bias | | Attrition Bias | Reporting Bias | Other Bias |
| --- | --- | --- | --- | --- | --- | --- | --- |
| Study | Random Sequence Generation | Allocation Concealment | Blinding (Participants & Personnel) | Blinding (Outcome Assessors) | Incomplete Outcome Data | Incomplete Outcome Reporting | Physically Inactive |
| Daley et al. (2006) | Low | Unclear | High | Unclear | Low | Low | Unclear |
| Davis et al. (2012) | Low | Low | High | High | Low | Low | Low |
| Farpour-Lambert et al. (2009) | Low | Low | High | Low | Low | Low | Low |
| Hagstromer et al. (2009) | Low | Unclear | High | Unclear | High | Low | High |
| Kelly et al (2004) | Low | Unclear | High | Unclear | Unclear | Low | Unclear |
| Maddison et al. (2011) | Low | Low | High | High | Low | Low | High |
| Meyer et al. (2006) | Low | Unclear | High | Unclear | High | Low | Low |
| Murphy et al. (2009) | Low | Unclear | High | Unclear | Unclear | Low | Unclear |
| Shaibi et al. (2006) | Low | Unclear | High | Unclear | High | Low | Unclear |
| Weintraub et al. (2008) | Low | Unclear | High | High | Low | Low | Unclear |

Notes: All studies were considered to be at a high risk of bias for blinding of participants and personnel because it’s impossible to blind subjects in exercise intervention studies.
