# Supplementary material for: Effects of exercise on BMI z-score in overweight and obese children and adolescents: a systematic review with meta-analysis
Source: BMC Pediatr. 2014 Sep 9;14:225. doi: 10.1186/1471-2431-14-225 (PMC4180550; doi:10.1186/1471-2431-14-225)
Supplement: Supplementary file 5 — Additional file 5: Table of categorical analyses results for BMI z-score. (DOCX 44 KB) [file 12887_2014_1161_MOESM5_ESM.docx]

Additional File 4. Table of categorical analyses results for BMI z-score.

| Variable | ES (#) | Participants (#) | $\bar{X}$ $\bar{\times}$(95% CI) |  |  | Q_b_(*p*) |
| --- | --- | --- | --- | --- | --- | --- |
| Country  - USA  - Other | 6  5 | 320  515 | -0.051 (-0.085, -0.017)  -0.079 (-0.129, -0.029) |  |  | 0.84 (0.36) |
| Type of Control  - Nonintervention  - Attention control | 7  4 | 541  294 | -0.069 (-0.111, -0.028)  -0.053 (-0.094, -0.012) |  |  | 0.29 (0.59) |
| IPD provided  - Yes  - No | 7  4 | 381  454 | -0.044 (-0.075, -0.013)  -0.098 (-0.154, -0.042) |  |  | 2.74 (0.10) |
| Sequence generation  - Low  - High  - Unclear | 11  --  -- | 835  --  -- | -0.055 (-0.071, -0.039)  --  -- |  |  | -- |
| Allocation concealment  - Low  - High  - Unclear | 4  --  7 | 588  --  247 | -0.069 (-0.096, -0.042)  --  -0.057 (-0.105, -0.010) |  |  | 0.17 (0.68) |
| Blinding (Participants/Personnel)  - Low  - High  - Unclear | --  11  -- | --  835  -- | --  -0.061 (-0.089, -0.034)  -- |  |  | --  -- |
| Blinding (Outcome Assessors)^a^  - Low  - High  - Unclear | 1  4  6 | 44  565  226 | -0.100 (-0.059, -0.041)  -0.064 (-0.088, -0.040)  -0.055 (-0.109, -0.001) |  |  | 1.45 (0.48) |
| Incomplete outcome data  - Low  - High  - Unclear | 6  3  2 | 660  120  55 | -0.060 (-0.088, -0.032)  -0.118 (-0.240, 0.003)  -0.018 (-0.073, 0.038) |  |  | 2.93 (0.23) |
| Selective outcome reporting  - Low  - High  - Unclear | 11  --  -- | 835  --  -- | -0.055 (-0.071, -0.039)  --  -- |  |  | -- |
| Previously Inactive  - Low  - High  - Unclear | 4  2  5 | 333  353  149 | -0.100 (-0.161, -0.040)  -0.065 (-0.092, -0.037)  -0.021 (-0.049, 0.006) |  |  | 7.87(0.02)* |
| Type of analysis  - Per protocol  - Intention to treat | 3  6 | 120  660 | -0.118 (-0.240, 0.003)  -0.060 (-0.088, -0.032) |  |  | 0.11 (0.74) |
| Sample size estimate  - Yes  - No | 6  5 | 660  175 | -0.060 (-0.088, -0.032)  -0.073 (-0.147, 0.001) |  |  | 0.11 (0.74) |
| Funding for study  - Yes  - No | 11  -- | 835  -- | -0.061 (-0.089, -0.034)  -- |  |  | -- |
| Adverse events  - Yes  - No | 1  3 | 149  416 | -0.040 (-0.080, 0.001)  -0.072 (-0.096, -0.047) |  |  | -- |
| Smoking  - Yes  - No  - Some | --  1  -- | --  67 | --  -0.290 (-0.449, -0.131  -- |  |  | -- |
| Alcohol consumption  - Yes  - No  - Some | --  --  -- | --  --  -- | --  --  -- |  |  | -- |
| Hyperlipidemia  - Yes  - No  - Some | --  1  1 | --  20  44 | --  0.000 (-0.052, 0.052)  -0.100 (-0.159, -0.041) |  |  | -- |
| Type 1 diabetes  - Yes  - No  - Some | --  4  -- | --  160  -- | --  -0.047 (-0.121, 0.026)  -- |  |  | -- |
| Type 2 diabetes  - Yes  - No  - Some | --  2  -- | --  109  -- | --  -0.004 (-0.051, 0.043)  -- |  |  | -- |
| Asthma  - Yes  - No  - Some | --  1  1 | --  20  35 | --  0.000 (-0.052, 0.052)  -0.063 (-0.160, 0.034) |  |  | -- |
| Change in exercise  - Increase  - Decrease  - No change | --  1  2 | --  31  95 | --  0.000 (-0.052, 0.052)  -0.074 (-0.147, -0.001) |  |  | -- |
| Type of exercise^a^  - Aerobic  - Strength  - Aerobic + strength | 8  1  2 | 738  22  75 | -0.057 (-0.090, -0.023)  -0.021 (-0.129, 0.087)  -0.096 (-0.141, -0.051) |  |  | 2.63 (0.27) |
| Exercise Intensity  - Moderate  - High | 7  4 | 571  265 | -0.049 (-0.082, -0.003)  -0.064 (-0.009, -0.003) |  |  | 0.002(0.96) |
| Exercise delivery^a^  - Supervised  - Unsupervised  - Supervised + Unsupervised | 8  2  1 | 427  357  51 | -0.073 (-0.112, -0.034)  -0.060 (-0.089, -0.032)  -0.010 (-0.052, 0.032) |  |  | 5.37 (0.07) |
| Exercise Location^a^  - Facility  - Home  - Facility + Home  Exercise Participation^a^  - Group  - Self  - Group + Self | 8  2  1  8  2  1 | 427  357  51  427  357  51 | -0.073 (-0.112, -0.034)  -0.060 (-0.089, -0.032)  -0.010 (-0.052, 0.032)  -0.073 (-0.112, -0.034)  -0.060 (-0.089, -0.032)  -0.010 (-0.052, 0.032) |  |  | 5.37 (0.07)  5.37 (0.07) |

Notes: ES (#), number of effect sizes; Participants (#), total number of participants nested within effect sizes; $\bar{X}$ $\bar{\times}$(95% CI), mean and 95% confidence intervals; Q_b_(*p*), between-group difference (Q_b_) and alpha value (*p*); --, Insufficient data reported; ^a^_,_ Analysis limited to categories with at least 2 ES per category; *, p < 0.05;
