# Supplementary material for: Effects of exercise on BMI z-score in overweight and obese children and adolescents: a systematic review with meta-analysis
Source: BMC Pediatr. 2014 Sep 9;14:225. doi: 10.1186/1471-2431-14-225 (PMC4180550; doi:10.1186/1471-2431-14-225)
Supplement: Supplementary file 6 — Additional file 6: Mixed effects meta-regression results for changes in BMI z-score. (DOCX 36 KB) [file 12887_2014_1161_MOESM6_ESM.docx]

Additional File 5. Mixed effects meta-regression results for changes in BMI z-score.

|  |  | |  | | |  | |  | | |
| --- | --- | --- | --- | --- | --- | --- | --- | --- | --- | --- |
| Variable | | ES  (#) | | Participants (#) | $\beta$_1_ + SE | | CI (95%) | | *Z(p*) |  |
| Year of publication | | 11 | | 835 | -0.006 + 0.005 | | -0.016, 0.004 | | -1.20(0.23) |  |
| Dropouts (%) | | 9 | | 769 | -0.002 + 0.002 | | -0.005, 0.002 | | -1.14(0.25) |  |
| Age (years) | | 11 | | 835 | 0.003 + 0.008 | | -0.013, 0.018 | | 0.37(0.71) |  |
| BMI z-score (Initial) | | 11 | | 835 | 0.012 + 0.027 | | -0.040, 0.065 | | 0.46(0.64) |  |
| Exercise Characteristics  - Length (weeks)  - Frequency (days/week)  - Duration (minutes/session)  - Minutes/week  - Minutes/week (adj.)  - MET minutes/week  - MET minutes/week (adj.)  - Total minutes  - Total minutes (adj.)  - Compliance (%) | | 11  11  11  11  5  11  5  11  5  5 | | 835  835  835  835  309  835  309  835  309  309 | -0.004 + 0.003  0.003 + 0.009  -0.0002 + 0.0007  -0.0002 + 0.0002  -0.0007 + 0.0004  -0.00002 + 0.00008  -0.00004 + 0.00006  -0.00002 + 0.00001  -0.00004 + 0.00003  0.0004 + 0.0011 | | -0.009, 0.002  -0.016, 0.020  -0.002, 0.001  -0.0006, 0.0003  -0.0015, 0.00003  -0.00008, 0.00004  -0.0002, 0.00007  -0.00004, 0.00000  -0.00009, 0.00001  -0.002, 0.003 | | -1.26(0.21) 0.28(0.78)  -0.33(0.74)  -0.66(0.51)  -1.9(0.06)  -0.67(0.50)  -0.73(0.46)  -1.62(0.11)  -1.40(0.16)  0.37(0.71) |  |

Notes: Notes: BMI, body mass index; ES(#), number of effect sizes; Participants (#), number of participants nested within effect sizes; $\beta$_1_ + SE, slope + standard error; CI(95%), 95% confidence intervals; *Z(p*), *z-*score and alpha value; MET, metabolic equivalent; (adj), adjusted for compliance.
